# A comprehensive taxonomic checklist of bark and ambrosia beetle species (Curculionidae, Scolytinae)

**DOI:** 10.3897/zookeys.1271.168928

**Published:** 2026-02-27

**Authors:** Andrew J. Johnson, Thomas H. Atkinson, Roger A. Beaver, Anthony I. Cognato, Bjarte H. Jordal, Miloš Knížek, Michail Yu. Mandelshtam, Robert J. Rabaglia, Sarah M. Smith, Jiri Hulcr

**Affiliations:** 1 School of Forest, Fisheries, and Geomatics Sciences, University of Florida, Gainesville, Florida, USA School of Forest, Fisheries, and Geomatics Sciences, University of Florida Gainesville United States of America; 2 Division of Plant Industries, Florida Department of Agriculture and Consumer Services, Gainesville, Florida, 32609, USA Department of Entomology and Nematology, University of Florida Gainesville United States of America; 3 University of Texas Insect Collection, 3001 Lake Austin Blvd., Suite 1.314 Austin, Texas 78702, USA Saint-Petersburg State Forest Technical University named after S.M. Kirov Saint-Petersburg Russia; 4 161/2 Mu 5, Soi Wat Pranon, T. Donkaew, A. Maerim, Chiangmai 50180, Thailand Department of Forest Protection Service, Forestry and Game Management Research Institute Jíloviště – Strnady Czech Republic; 5 Department of Entomology, Michigan State University 288 Farm Lane, 243 Natural Science Bldg., East Lansing, Michigan 48824, USA University Museum of Bergen, University of Bergen Bergen Norway; 6 Department of Natural History, University Museum of Bergen, University of Bergen, P.O. 7800, NO-5020 Bergen, Norway Division of Plant Industries, Florida Department of Agriculture and Consumer Services Gainesville United States of America; 7 Department of Forest Protection Service, Forestry and Game Management Research Institute, Jíloviště – Strnady, CZ-252 02, Czech Republic Michigan State University 288 Farm Lane East Lansing United States of America; 8 Department of Forest Protection, Wood Science and Game Management, Saint-Petersburg State Forest Technical University named after S.M. Kirov, Institutskii per., 5, Saint-Petersburg, 194021, Russia University of Texas Insect Collection Austin United States of America; 9 Maryland Department of Agriculture, Forest Pest Management, 28577 Mary's Ct, Suite 4, Easton, Maryland 21601, USA Unaffiliated Chiangmai Thailand; 10 Department of Entomology and Nematology, University of Florida, Gainesville, Florida, 32609, USA Maryland Department of Agriculture, Forest Pest Management Easton United States of America

**Keywords:** Bark beetle, inventory, worldwide

## Abstract

A comprehensive list of the currently (as of 17 November 2025) valid taxa of bark and ambrosia beetles of the world (Curculionidae: Scolytinae) is presented, including all tribes, genera, species (6516), and subspecies. The high diversity of scolytine species, their biosecurity significance, and the rapidly advancing understanding of their phylogenetic relationships has resulted in decentralized and often incorrect usage of names. The list represents species names accepted and used in compliance with the International Code of Zoological Nomenclature and a consensus of name usage by the community of active systematists. Species names listed here are bona fide hypotheses of biological species, but not always of confirmed biological entities; instead, the list aims to provide a foundation for the subsequent taxonomic, biological, and phylogenetic work on species resolution, and for biodiversity data management. The list is presented as a formatted checklist, as well as a Darwin-core format enabling interpolation into biodiversity information systems. Two replacement names were needed to resolve homonyms, *Hylastes woodi* Johnson, nom. nov. (= *Hylastes niger* Wood, 1974) and *Taphrorychus krivolutskayae* Johnson, nom. nov. (= *Dryocoetes aceris* Krivolutskaya, 1968).

## Introduction

Classification of species is the foundation of research and management of biological systems. Bark and ambrosia beetles (Coleoptera: Curculionidae: Scolytinae) are an example of a group with a large impact on human interests, combined with high species diversity and a history of challenging systematics. There are more than 9000 names for more than 6500 valid species and subspecies names, described in numerous taxonomic publications, journals, and books. Tracking the taxonomy of Scolytinae was historically difficult. For decades, synthesis was only achieved on narrow regional levels. The only global synthesis is the comprehensive Catalog of species and its supplements (Wood and Bright 1987, 1992; Bright and Skidmore 1997, 2002; Bright 2014, 2021). These publications transformed research and remain useful resources for finding information about species, type repositories, and locating literature.

Since then, however, ambrosia and bark beetle systematics have undergone rapid changes. The community now involves the greatest number of actively working bark beetle systematists at the same time, supporting thousands of bark beetle classification users, from biologists to biosecurity agencies. This expanding science requires a reliable and unified classification. Several revisionary systematics projects coupled with molecular methods resulted in hundreds of taxonomic changes, but only the Palaearctic region has been catalogued comprehensively (Knížek 2011; Alonso-Zarazaga et al. 2017, 2023). Several published higher classifications (e.g., Bright 2014, 2021) are subjective and do not follow contemporary systematics (Jordal et al. 2014). This further complicates the problem of unifying nomenclature.

The biodiversity information ecosystem is also rapidly evolving. Taxonomic information is now widely available via online data repositories, but those depend on interoperable taxonomic information. Taxonomic databases such as NCBI taxonomy (which is used by GenBank) and Global Biodiversity Infrastructure (GBIF) utilize taxonomic information to connect newly generated data but contain unverified or even erroneous information. Bark and ambrosia beetles are an example of a group with few digitally discoverable, interoperable, and accurate taxonomic data sources. The increase of digitized museum collections is a welcome development, but the taxonomy and nomenclature of institutional bark beetle collections has rarely been kept up to date, and so the resulting digital resources are rife with incorrect names. This authoritative global checklist of Scolytinae species aims to address many of these issues.

The checklist is not an attempt to resolve all the taxonomic ambiguities in the scolytine systematics. It is a list of valid taxonomic names, conforming to the ICZN code, and derived from bona fide attempts to define biological entities. However, users of this list should not assume that species included herein have all been biologically validated, or that the process of resolving name usage has been completed, or that the species placement within higher classification (genera, tribes) has been settled, or that the taxonomy reflects phylogeny. Subsequent work on resolving species identity, placement, and name usage is encouraged, and this list should serve as a starting point for further systematic research. In addition, this checklist is formatted for machine-ingestion which will facilitate the uptake of these data by online aggregators and use by researchers and institutions.

## Methods

The original list was created from two unpublished lists, provided by THA and RJR, which were derived from Wood and Bright (1992) and subsequently updated. Discrepancies in the two initial lists were first identified, then resolved by consulting primary literature and/or other taxonomists’ own lists to ensure accurate year of publication and page of description. All authors reviewed the resulting list to identify missing taxonomic changes, especially recent ones.

The bibliographic references were initially taken from Wood and Bright (1987), Alonso-Zarazaga and Lyal (2009), Alonso-Zarazaga et al. (2023), and from the primary literature, especially those within the Biodiversity Heritage Library.

For higher taxonomy and classification, we followed Alonso-Zarazaga and Lyal (2009), amended with subsequent changes found in the recent literature. We included Coptonotini, despite its dubious placement within Scolytinae, given no act has expressly transferred it from Scolytinae, although it has been treated in Platypodinae (Wood 1993) and in its own subfamily within Curculionidae (Smith and Cognato 2016). The monophyly of Scolytinae has also been questioned with the potential exclusion of the tribe Scolytini (Shin et al. 2018), but formal taxonomic changes have not been proposed. The monotypic genus *Cryphyophthorus* Schedl was retained in Hypoborini despite its inconclusive molecular phylogenetic and morphological placement (Jordal 2021a).

We included described synonyms of species and subspecies with a citation of the original description, but not subsequent taxonomic acts, e.g., synonymy. A comprehensive catalog of taxonomic acts is needed but beyond the scope of this project.

Some names required emendation from the original description or most recent usage based on correcting Latin grammar, particularly for names formed from adjectives when matching the gender of the genus. The following are newly emended here: *Beaverium rufonitidus* (Schedl, 1951), *Cyclorhipidion capense* (Eggers, 1944), *Cyclorhipidion conidentatum* Smith, Beaver & Cognato, 2022, *Cyclorhipidion kajangense* (Schedl, 1942), *Ctonoxylon kivuense* Schedl, 1957, *Cyclorhipidion malayense* (Browne, 1981), *Cyclorhipidion obiense* (Browne, 1980), *Euwallacea illustrior* (Schedl, 1939), *Karlsenius ghanaense* (Schedl, 1977), *Liparthrum caymanense* Bright, 2019, *Liparthrum meridense* Wood, 1971, *Liparthrum sabahense* Bright, 1990, *Liparthrum tinianense* Wood, 1988, *Monarthrum boliviense* Wood, 1989, *Monarthrum catarinense* Wood, 2007, *Monarthrum durangoense* Wood, 2007, *Monarthrum gracilius* (Schedl, 1959), *Monarthrum xalapense* Wood, 1987, *Scolytodes peruanus* Wood, 2007, *Styphlosoma brasiliense* (Schedl, 1972), *Truncaudum leverense* (Browne, 1986), *Truncaudum longius* (Eggers, 1923).

We recognize *Hylastes ater* Erichson, 1836 as a valid name for the species widely known as “Hylastes ater (Paykull, 1800)”, made available by Article 11.10, and with reversal of precedence over *Ips niger* Marsham, 1802 based on Article 23.9.

Two unresolved homonyms were discovered during the compilation of this checklist, and we propose the following replacement names. *Hylastes woodi*, Johnson, nom. nov. is proposed as a substitute name for *Hylastes niger* Wood, 1974a, a secondary homonym of *Ips niger* Marsham, 1802, now in *Hylastes* as a synonym of *Hylastes ater* Erichson, 1836. The new name is a noun in the genitive case, and honors the original author of the species, Stephen L. Wood. *Taphrorychus krivolutskayae* Johnson, nom. nov. is proposed as a substitute name for *Taphrorychus aceris* (Krivolutskaya,1968) (originally *Dryocoetes*), which is a primary homonym of *Lymantor aceris aceris* (Lindemann, 1875a), described with the identical name, *Dryocoetes aceris*. The new name is a noun in the genitive case honoring the original author, Gali O. Krivolutskaya.

This list is objective, with taxonomic changes (synonymy, rank changes, and combinations) mostly based on expressed acts or implicitly from listing a name as a synonym or a novel combination. Fossil taxa are excluded. Subspecies are listed at the same level as species, not as a child of their species. Subgenera are not included. Following Recommendation 51B (ICZN code online), authorship names in languages without Latin characters are transliterated to Latin letters. For Russian names, transliteration follows BGN/PCGN romanization of Russian, except in cases where the author provided their own Romanized spelling. When three or more authors described a species, they are listed in full. For authors with multiple family names (e.g., Spanish and some Hispanic countries), we follow the Chicago Manual of Style, where only the first family name is used. When authors share a last name, we include the first initial, which is enough to resolve all authority names in the list.

The names are presented with the following structure: a valid species or subspecies is listed with a bullet point. The authority and citation are then listed, with parentheses if the species was first described in a different genus. Names which were described in an unavailable genus with an identical spelling to its contemporary genus are also listed with parentheses, denoted with an asterisk. If the description authority differs from the publication authorship, the publication is referenced following “in” (following recommendation 51E). Then, the year of publication (meeting ICZN standards of publication, Article 21) and a letter in ambiguous cases, a colon, and then the page where the taxonomic treatment starts. Names emended from the original description are marked with “(E)”, but this does not include emended combinations, i.e., names which have been used in non-original combinations but with incorrect grammar. For these only the correct name is shown, even in cases where this is the first use of the correct spelling. Emendations are only considered for the specific or subspecific epithet, not for the spelling of the genus, which is corrected without comment. If the name was given as a substitute/replacement name, a note is marked with “(RN)”. Then, if the genus name differs from its original description, the original genus is included in parentheses. If the specific or subspecific epithet differs (though gender changes or rank changes), then these are also included. Then, on a new line, synonymous names are included with their original combination only, preceded by “=”, with the authority, publication authors (if different), year, letter (if the citation is ambiguous), a colon and the page number, plus a note on whether the name is emended and/or a replacement name. Only bibliographic references to the description of species and subspecies presented. The references pertaining to the synonymy and other taxonomic acts are not included, nor are those related to descriptions of higher taxonomy. Synonyms are listed by the year of description and then alphabetically, which may not necessarily represent the order of priority nor the order of publications within a year.

The counts of genera, species and subspecies are included for convenience. The total number of species includes those listed as nominate subspecies. The counts of subspecies include the nominate subspecies as well.

## Results

We have identified 6516 species in 270 genera, valid as of 17 November 2025. The list is available in tabular format with Darwin Core headers (following GBIF 2017), and as a formatted checklist, published with xml markup for maximum interoperability.

### SCOLYTINAE Latreille

30 tribes, 270 genera, 6516 spp. (30 spp. with 68 sspp.).

### AMPHISCOLYTINI Mandelshtam & Beaver

1 genus, 1 sp.

#### *Amphiscolytus* Mandelshtam & Beaver

1 sp.

• *Amphiscolytus capensis* (Schedl, 1971d: 6) (*Dacryophthorus*).

### BOTHROSTERNINI Blandford

6 genera, 134 spp.

#### *Akrobothrus* Dole & Cognato

1 sp.

• *Akrobothrus ecuadoriensis* Dole & Cognato, 2007: 321.

#### *Bothrosternus* Eichhoff

11 spp.

• *Bothrosternus affinis* Eggers, 1933f: 14.

• *Bothrosternus artestrigosus* Schedl, 1939h: 722.

• *Bothrosternus brevis* Eggers, 1933f: 15.

• *Bothrosternus definitus* Wood, 1968b: 109.

• *Bothrosternus foveatus* (Blackman, 1943d: 375) (*Cnesinus*).

• *Bothrosternus hirsutus* Wood, 1985: 271.

• *Bothrosternus isolatus* Bright, 1972a: 28.

• *Bothrosternus lucidus* Wood, 1974a: 6.

• *Bothrosternus rudis* Wood, 2007: 104.

• *Bothrosternus subopacus* Schedl, 1963f: 217.

• *Bothrosternus truncatus* Eichhoff, 1868b: 150.

= *Bothrosternus striatus* Eggers, 1933f: 16.

#### *Cnesinus* LeConte

98 spp.

• *Cnesinus acuminatus* Schedl, 1978a: 298.

• *Cnesinus adusticus* Wood, 1967b: 87.

• *Cnesinus adustus* Schedl, 1949b: 266.

= *Cnesinus atrodeclivis* Wood, 1968b: 108.

• *Cnesinus advena* Schedl, 1973b: 169.

• *Cnesinus alienus* Wood, 1974a: 3.

• *Cnesinus ampliatus* Schedl, 1938f: 21.

• *Cnesinus amplipennis* Schedl, 1963f: 219.

• *Cnesinus amplus* Bright, 2019: 23.

• *Cnesinus annectens* Wood, 1967b: 86.

• *Cnesinus atavus* Wood, 1968b: 106.

• *Cnesinus atrocis* Wood, 1982: 226.

• *Cnesinus beaveri* Wood, 1974a: 2.

• *Cnesinus bicinctus* Schedl, 1954b: 29.

• *Cnesinus bicolor* Eggers, 1943d: 376.

• *Cnesinus bicornus* Wood, 1967b: 80.

• *Cnesinus bispinatus* Schedl, 1976: 62.

• *Cnesinus bisulcatus* Schedl, 1949b: 266.

• *Cnesinus bituberculatus* Schedl, 1966d: 102.

• *Cnesinus blackmani* Schedl, 1949b: 268 (RN).

= *Cnesinus nitidus* Blackman, 1943d: 377.

= *Cnesinus mexicanus* Nunberg, 1956c: 207 (RN).

• *Cnesinus brevisetosus* Bright, 2019: 24.

• *Cnesinus brighti* Wood, 1974a: 6.

• *Cnesinus carbonarius* Schedl, 1952e: 354.

• *Cnesinus carinatus* Wood, 1967b: 88.

• *Cnesinus coffeae* Schedl, 1939g: 12.

• *Cnesinus colombianus* Wood, 1967b: 84.

• *Cnesinus coracinus* Wood, 1974a: 6.

• *Cnesinus cornutus* Wood, 1983: 655.

• *Cnesinus costulatus* Blandford, 1896d: 137.

= *Cnesinus similis* Blackman, 1943d: 375..

• *Cnesinus cubensis* Blackman, 1943d: 371..

• *Cnesinus degener* Wood, 1968b: 105.

• *Cnesinus denotatus* Wood, 1968b: 107.

• *Cnesinus deperditus* Wood, 1974a: 4.

• *Cnesinus dividuus* Schedl, 1938f: 22.

= *Cnesinus dryographus* Schedl, 1951c: 78.

= *Cnesinus laevicollis* Schedl, 1951c: 79.

• *Cnesinus electinus* Wood, 1967b: 82.

• *Cnesinus electus* Wood, 1975a: 23.

• *Cnesinus elegans* Blandford, 1896d: 140.

• *Cnesinus elegantis* Wood, 1967b: 79.

= *Cnesinus zapotecus* Bright, 1972c: 1493.

• *Cnesinus equihuai* Wood, 1982: 226.

• *Cnesinus excellens* Wood, 2007: 85.

• *Cnesinus foratus* Wood, 1967b: 81.

• *Cnesinus frontalis* Wood, 1968b: 104.

• *Cnesinus fulgens* Wood, 1974a: 4.

• *Cnesinus fulgidus* Wood, 1974a: 5.

• *Cnesinus garrulus* Schedl, 1940a: 332.

• *Cnesinus gibbosus* Wood, 1968b: 101.

• *Cnesinus gibbulus* Wood, 1968b: 100.

• *Cnesinus gibbus* (Chapuis, 1869: 28) (*Nemophilus*).

• *Cnesinus gracilis* Blandford, 1896d: 141.

= *Cnesinus substrigatus* Blackman, 1943d: 376.

= *Cnesinus laetus* Schedl, 1978a: 299.

• *Cnesinus grandis* Schedl, 1935g: 273.

• *Cnesinus guadeloupensis* Eggers, 1940a: 137.

• *Cnesinus hispidulosus* Wood, 2007: 93.

• *Cnesinus hispidus* Eggers, 1943d: 378.

• *Cnesinus insularis* Eggers, 1940a: 138.

• *Cnesinus intermedius* Schedl, 1936a: 105.

• *Cnesinus lecontei* Blandford, 1896d: 138.

• *Cnesinus longicollis* Eggers, 1940a: 137.

• *Cnesinus lucaris* Wood, 1974a: 5.

• *Cnesinus meris* Wood, 1982: 226.

• *Cnesinus minax* Schedl, 1952e: 352.

• *Cnesinus minitropis* Wood, 1968b: 105.

• *Cnesinus minor* Wood, 1985: 272.

• *Cnesinus minusculus* Schedl, 1952e: 353.

• *Cnesinus myelitis* Wood, 1967b: 84.

• *Cnesinus nebulosus* Wood, 1983: 655.

• *Cnesinus niger* Wood, 1967b: 83.

• *Cnesinus nitidus* Eggers, 1943d: 376.

= *Cnesinus discretus* Wood, 1985: 271.

• *Cnesinus noguerae* Atkinson, 1989a: 57.

• *Cnesinus ocularis* Blandford, 1896d: 140.

• *Cnesinus paleatus* Blandford, 1896d: 138.

• *Cnesinus parvicornis* Wood, 1983: 656.

• *Cnesinus perplexus* Wood, 1968b: 102.

• *Cnesinus pilatus* Wood, 1975a: 24.

• *Cnesinus plaumanni* Schedl, 1963f: 220.

• *Cnesinus porcatus* Blandford, 1896d: 137.

• *Cnesinus prominulus* Wood, 1977a: 212.

• *Cnesinus pulchellus* Wood, 2007: 80.

• *Cnesinus pullus* Blandford, 1896d: 141.

• *Cnesinus punctatus* Blandford, 1896d: 136.

• *Cnesinus pusillus* Schedl, 1949b: 267.

• *Cnesinus quaesitus* Schedl, 1940a: 331.

• *Cnesinus reticulatus* (Chapuis, 1869: 29) (*Hylesinus*).

• *Cnesinus reticulus* Wood, 1974a: 2.

• *Cnesinus retifer* Wood, 1967b: 85.

• *Cnesinus robai* Blackman, 1943d: 374.

• *Cnesinus schoenherri* Schedl, 1976: 63.

• *Cnesinus schulzi* Wood, 2007: 89.

• *Cnesinus setosus* Eggers, 1943d: 377.

• *Cnesinus setulosus* Blandford, 1896d: 139.

= *Cnesinus flavopilosus* Schedl, 1940a: 333.

= *Cnesinus cognatus* Blackman, 1943d: 373.

= *Cnesinus panamensis* Blackman, 1943d: 372.

• *Cnesinus squamifer* Wood, 2007: 91.

• *Cnesinus squamosus* Wood, 1968b: 102.

• *Cnesinus strigicollis* LeConte, 1868: 171.

= *Nemophilus strigillatus* Chapuis, 1869: 27.

• *Cnesinus sulcatus* Eggers, 1931b: 34.

= *Cnesinus novateutonicus* Schedl, 1951c: 77.

• *Cnesinus teres* Blandford, 1896d: 141.

• *Cnesinus teretis* Wood, 1974a: 3.

• *Cnesinus theocallus* Bright, 1972c: 1493.

• *Cnesinus triangularis* Wood, 1974a: 5.

• *Cnesinus vestitus* Eggers, 1933f: 16.

• *Cnesinus vorontsovi* Petrov, 2014: 151.

#### *Eupagiocerus* Blandford

3 spp.

• *Eupagiocerus ater* Eggers, 1931a: 14.

= *Eupagiocerus nevermanni* Schedl, 1952e: 350.

= *Eupagiocerus serratus* Wood, 1961c: 104.

• *Eupagiocerus dentipes* Blandford, 1896d: 133.

= *Eupagiocerus clarus* Wood, 1965: 33.

• *Eupagiocerus vastus* Wood, 1965: 34.

#### *Pagiocerus* Eichhoff

5 spp.

• *Pagiocerus cribricollis* Eichhoff, 1868b: 148.

• *Pagiocerus eggersi* Wood, 2007: 100.

• *Pagiocerus frontalis* (Fabricius, 1801: 389) (*Bostrichus*).

= *Pagiocerus rimosus* Eichhoff, 1868b: 148.

= *Bothrosternus hubbardi* Schwarz, 1886: 54.

= *Hylastinus fiorii* Eggers, 1908a: 215.

= *Pagiocerus chiriquensis* Eggers, 1928a: 92.

= *Pagiocerus zeae* Eggers, 1928a: 92.

= *Pagiocerus nitidus* Eggers, 1930a: 170.

= *Pagiocerus caraibicus* Eggers, 1940a: 136.

• *Pagiocerus luederwaldti* Eggers, 1928a: 93.

= *Pagiocerus major* Schedl, 1976: 64.

• *Pagiocerus punctatus* Eggers, 1928a: 93.

#### *Sternobothrus* Eggers

16 spp.

• *Sternobothrus ater* (Schedl, 1952e: 352) (*Cnesinus*).

• *Sternobothrus bicaudatus* (Blandford, 1896d: 133) (*Bothrosternus*).

= *Sternobothrus tuberculatus* Eggers, 1951: 152.

• *Sternobothrus bicostatus* (Schedl, 1936a: 106) (*Cnesinus*).

• *Sternobothrus cancellatus* (Chapuis, 1869: 25) (*Bothrosternus*).

• *Sternobothrus carinatus* Eggers, 1943d: 373.

• *Sternobothrus costatus* (Chapuis, 1869: 25) (*Bothrosternus*).

= *Bothrosternus lacordairei* Chapuis, 1869: 25.

• *Sternobothrus marginicollis* (Eggers, 1931a: 15) (*Cnesinus*).

• *Sternobothrus opaculus* Schedl, 1963f: 218.

• *Sternobothrus paraguayensis* (Schedl, 1936a: 107) (*Cnesinus*).

• *Sternobothrus pilosus* (Eggers, 1943d: 378) (*Cnesinus*).

• *Sternobothrus pumilus* (Eggers, 1931b: 34) (*Cnesinus*).

• *Sternobothrus rufonitidus* Schedl, 1952e: 351.

• *Sternobothrus sculpturatus* (Blandford, 1896d: 132) (*Bothrosternus*).

• *Sternobothrus suturalis* (Eggers, 1931b: 32) (*Bothrosternus*).

• *Sternobothrus transitus* (Schedl, 1976: 63) (*Cnesinus*).

• *Sternobothrus vexator* (Schedl, 1963f: 220) (*Cnesinus*).

### CARPHODICTICINI Wood

3 genera, 6 spp.

#### *Carphodicticus* Wood

1 sp.

• *Carphodicticus cristatus* Wood, 1971a: 19.

#### *Craniodicticus* Blandford

4 spp.

• *Craniodicticus cinnamomi* Lin & Beaver, 2023: 129.

• *Craniodicticus minor* Eggers, 1936a: 635.

• *Craniodicticus mucronatus* Blandford, 1895: 317.

• *Craniodicticus sabahensis* Beaver, 1999: 202.

#### *Dendrodicticus* Schedl

1 sp.

• *Dendrodicticus argentiniae* Schedl, 1958j: 37.

### CHAETOPHLOEINI Jordal

1 genus, 34 spp.

#### *Chaetophloeus* LeConte

34 spp.

• *Chaetophloeus andinus* Wood, 1971a: 9.

• *Chaetophloeus atlanticus* Bright, 1981b: 158.

• *Chaetophloeus bahamaensis* Bright, 2019: 56.

• *Chaetophloeus beaveri* Petrov, 2021: 383.

• *Chaetophloeus braziliensis* (Blackman, 1940b: 398) (*Renocis*).

• *Chaetophloeus chapini* (Blackman, 1943d: 390) (*Renocis*).

• *Chaetophloeus confinis* Wood, 1983: 652.

• *Chaetophloeus coronatus* (Chapuis, 1869: 39) (*Phloeosinus*).

• *Chaetophloeus cubensis* Bright, 1981b: 159.

• *Chaetophloeus fasciatus* (Blackman, 1940b: 385) (*Renocis*).

• *Chaetophloeus flourensiae* Atkinson, 2024: 152.

• *Chaetophloeus heterodoxus* (Casey, 1886: 258) (*Renocis*).

= *Pseudocryphalus brittaini* Swaine, 1917: 20.

= *Pseudocryphalus criddlei* Swaine, 1917: 21.

= *Renocis brunneus* Blackman, 1940b: 389.

= *Renocis conmixtus* Blackman, 1940b: 392.

= *Renocis fuscus* Blackman, 1940b: 391.

• *Chaetophloeus howdeni* Bright, 1972a: 36.

• *Chaetophloeus hystrix* (LeConte, 1858: 81) (*Hylesinus*).

• *Chaetophloeus insularis* (Blackman, 1940b: 400) (*Renocis*).

• *Chaetophloeus lasius* Wood, 1956c: 251.

• *Chaetophloeus longisetum* Bright, 2019: 60.

• *Chaetophloeus maclayi* (Bruck, 1936: 35) (*Pseudocryphalus*).

• *Chaetophloeus mandibularis* Bright, 1981b: 160.

• *Chaetophloeus mexicanus* (Blackman, 1940b: 397) (*Renocis*).

= *Renocis mexicanus* Eggers, 1951: 149.

= *Renocis blackmani* Nunberg, 1956c: 208 (RN).

= *Renocis eggersi* Wood, 1956c: 253 (RN).

• *Chaetophloeus minimus* Wood, 1967b: 95.

• *Chaetophloeus minutus* Bright, 2019: 62.

• *Chaetophloeus montanus* Bright, 2019: 62.

• *Chaetophloeus parkinsoniae* (Blackman, 1940b: 378) (*Renocis*).

• *Chaetophloeus penicillatus* (Bruck, 1933c: 239) (*Renocis*).

• *Chaetophloeus phoradendri* Wood, 1969a: 8.

• *Chaetophloeus pouteriae* Wood, 1986a: 269.

• *Chaetophloeus pruinosus* (Blackman, 1940b: 383) (*Renocis*).

• *Chaetophloeus psittacanthi* Burgos & Atkinson, 2022: 73.

• *Chaetophloeus struthanthi* Wood, 1967b: 96.

• *Chaetophloeus sulcatus* (Wood, 1956c: 252) (*Renocis*).

• *Chaetophloeus woodi* Burgos & Atkinson, 2022: 77.

• *Chaetophloeus woodruffi* Bright, 2019: 64.

• *Chaetophloeus zapotecanus* Burgos & Atkinson, 2022: 80.

### COPTONOTINI Chapuis

1 genus, 4 spp.

#### *Coptonotus* Chapuis

4 spp.

• *Coptonotus cyclopus* Chapuis, 1869: 11.

• *Coptonotus doppelganger* Smith & Cognato, 2016: 417.

• *Coptonotus striatus* Eggers, 1933f: 3.

• *Coptonotus uteq* Smith & Cognato, 2016: 421.

### CORIACEPHILINI Johnson

1 genus, 5 spp.

#### *Coriacephilus* Schedl

5 spp.

• *Coriacephilus coriaceus* (Eichhoff, 1878b: 494) (*Stephanoderes*).

= *Cryphalus birmanus* Eggers, 1925: 153.

• *Coriacephilus cribripennis* Schedl, 1943: 40.

• *Coriacephilus exiguus* Beaver, 2005b: 57.

• *Coriacephilus proximus* (Eggers, 1925: 156) (*Cryphalus*).

• *Coriacephilus xyloctonoides* Schedl, 1939b: 340.

### CORTHYLINI LeConte

37 genera, 1330 spp. (5 spp. with 11 sspp.).

### CORTHYLINA LeConte

13 genera, 578 spp.

#### *Amphicranus* Erichson

68 spp.

• *Amphicranus acus* Wood, 1974a: 66.

• *Amphicranus apicalis* Wood, 2007: 698.

• *Amphicranus araguensis* Wood, 2007: 707.

• *Amphicranus argutus* Wood, 1976: 349.

• *Amphicranus attenuatus* Wood, 2007: 700.

• *Amphicranus bahiae* Wood, 2007: 700.

• *Amphicranus balteatus* Blandford, 1905: 291.

• *Amphicranus belti* Blandford, 1905: 292.

• *Amphicranus bipunctatus* Eichhoff, 1878b: 469.

• *Amphicranus brasiliensis* Eggers, 1931b: 37.

• *Amphicranus brevior* Wood, 2007: 709.

• *Amphicranus brevipennis* Blandford, 1905: 293.

• *Amphicranus brownei* Schedl, 1979c: 128 (RN).

= *Amphicranus elegantulus* Schedl, 1978a: 304.

= *Amphicranus electus* Wood, 1981: 122 (RN).

• *Amphicranus callosus* (Schedl, 1935f: 351) (*Pterocyclon*).

• *Amphicranus cordatus* (Bright, 1972b: 1380) (*Tricolus*).

• *Amphicranus cracens* Wood, 2007: 701.

• *Amphicranus dohrnii* (Eichhoff, 1878b: 461) (*Steganocranus*).

= *Amphicranus caudatus* Eggers, 1931a: 17.

• *Amphicranus eggersianus* Wood, 2007: 708.

• *Amphicranus eichhoffi* Eggers, 1931a: 17.

= *Steganocranus galeatus* Eggers, 1943d: 384.

• *Amphicranus electilis* Wood, 2007: 700.

• *Amphicranus elegantulus* Schedl, 1963f: 225.

• *Amphicranus explicitus* Wood, 2007: 699.

• *Amphicranus fastigiatus* Blandford, 1905: 296.

• *Amphicranus filiformis* Blandford, 1905: 295.

• *Amphicranus fryi* Blandford, 1905: 291.

• *Amphicranus fulgidus* Wood, 1976: 350.

• *Amphicranus gracilis* Eggers, 1943d: 383.

= *Tricolus gracilipennis* Schedl, 1950c: 170.

• *Amphicranus grouvellei* Blandford, 1905: 294.

• *Amphicranus hispaniolus* Bright, 2019: 396.

• *Amphicranus hybridus* Blandford, 1905: 298.

• *Amphicranus incisus* (Schedl, 1976: 84) (*Tricolus*).

• *Amphicranus laureli* Wood, 2007: 704.

• *Amphicranus lesnei* Hagedorn, 1905a: 550.

• *Amphicranus macellus* Wood, 1974a: 64.

• *Amphicranus melanurus* (Blandford, 1904: 272) (*Pterocyclon melanura*).

= *Pterocyclon opacifrons* Schedl, 1935f: 350.

• *Amphicranus micans* Wood, 1976: 350.

• *Amphicranus micidus* Wood, 2007: 702.

• *Amphicranus minor* (Eggers, 1935d: 332) (*Anchonocerus*).

• *Amphicranus mirandus* Wood, 1974a: 63.

• *Amphicranus mucronatus* Wood, 1974a: 66.

• *Amphicranus parilis* Wood, 1975a: 31.

• *Amphicranus plaumanni* Schedl, 1978a: 305.

• *Amphicranus politus* Eichhoff, 1869: 276.

• *Amphicranus propugnatus* Blandford, 1905: 297.

= *Amphicranus armatus* Schedl, 1934b: 37.

• *Amphicranus quadridens* Wood, 2007: 708.

• *Amphicranus quadrimaculatus* Schedl, 1966d: 127.

• *Amphicranus rameus* Wood, 1967a: 55.

• *Amphicranus rasilis* Schedl, 1950c: 174.

• *Amphicranus schaufussi* Blandford, 1905: 293.

• *Amphicranus sexdentatus* (Eggers, 1935d: 331) (*Anchonocerus*).

= *Monarthrum sexdenticulum* Wood, 1989: 178 (RN).

• *Amphicranus speciosus* (Schedl, 1934b: 38) (*Tricolus*).

• *Amphicranus spectabilis* (Wood, 1967c: 140) (*Tricolus*).

• *Amphicranus spectus* Wood, 1980b: 358.

• *Amphicranus spinachius* (Schedl, 1939d: 584) (*Steganocranus*).

• *Amphicranus spinescens* Wood, 1974a: 65.

• *Amphicranus spinosus* Wood, 1974a: 65.

• *Amphicranus splendens* Wood, 1982: 223.

• *Amphicranus stenodermus* (Schedl, 1963c: 166) (*Pterocyclon*).

• *Amphicranus taino* Bright, 2019: 396.

• *Amphicranus tenuis* Blandford, 1905: 295.

• *Amphicranus terebella* Blandford, 1905: 296.

• *Amphicranus theobroma* Sampson, 1912: 245.

• *Amphicranus thoracicus* Erichson, 1836: 64.

= *Piezorhopalus nitidulus* Guérin-Méneville, 1838: 107.

= *Amphicranus elegans* Eichhoff, 1869: 276.

= *Amphicranus retusus* Eichhoff, 1869: 276.

= *Amphicranus crenatus* Eichhoff, 1878b: 465.

• *Amphicranus thunesi* Wood, 2007: 706.

• *Amphicranus tornatilis* Wood, 1974a: 64.

• *Amphicranus torneutes* Blandford, 1905: 292.

• *Amphicranus ursus* Schedl, 1934b: 37.

• *Amphicranus woytkowskii* Wood, 2007: 708.

#### *Brachyspartus* Ferrari

1 sp.

• *Brachyspartus moritzi* Ferrari, 1867: 68.

= *Corthylus obtusus* Schedl, 1966d: 122.

#### *Corthylocurus* Wood

16 spp.

• *Corthylocurus aguacatensis* (Schedl, 1940a: 357) (*Metacorthylus*).

• *Corthylocurus barbatus* (Blandford, 1904: 265) (*Brachyspartus*).

• *Corthylocurus cincinnatus* Bright, 1972b: 1379.

• *Corthylocurus costaricensis* (Schedl, 1936a: 108) (*Microcorthylus*).

• *Corthylocurus debilis* Wood, 1974b: 150.

• *Corthylocurus medialis* Wood, 2007: 801.

• *Corthylocurus mexicanus* (Schedl, 1950c: 163) (*Brachyspartus*).

= *Corthylus cylindricus* Schedl, 1963c: 164.

= *Corthylus anomalus* Bright, 1972b: 1378.

• *Corthylocurus moritzi* Wood, 2007: 800.

• *Corthylocurus pisinnus* (Bright, 1972a: 103) (*Corthylus*).

• *Corthylocurus pristinus* Wood, 2007: 800.

• *Corthylocurus protuberans* Wood, 2007: 801.

• *Corthylocurus reticulatus* Wood, 2007: 803.

• *Corthylocurus setifer* Wood, 2007: 803.

• *Corthylocurus signatifrons* (Schedl, 1950c: 162) (*Brachyspartus*).

• *Corthylocurus tuberculifer* (Eggers, 1937b: 86) (*Brachyspartus*).

• *Corthylocurus vernaculus* (Schedl, 1939d: 569) (*Brachyspartus*).

#### *Corthyloxiphus* Wood

21 spp.

• *Corthyloxiphus antennatus* Wood, 2007: 794.

• *Corthyloxiphus apicalis* Wood, 2007: 793.

• *Corthyloxiphus araguensis* Wood, 2007: 791.

• *Corthyloxiphus aztecus* (Bright, 1972b: 1374) (*Corthylus*).

• *Corthyloxiphus caliginis* (Wood, 1974b: 148) (*Corthycyclon*).

• *Corthyloxiphus carbonerae* Wood, 2007: 794.

• *Corthyloxiphus colombiae* Wood, 2007: 793.

• *Corthyloxiphus declivis* Wood, 2007: 794.

• *Corthyloxiphus ebeninus* (Blandford, 1904: 265) (*Brachyspartus*).

• *Corthyloxiphus emarginatus* (Eggers, 1943d: 380) (*Corthylus*).

• *Corthyloxiphus frontalis* Wood, 2007: 792.

• *Corthyloxiphus furvus* (Wood, 1974b: 148) (*Corthycyclon furvum*).

• *Corthyloxiphus morulus* (Wood, 1974b: 149) (*Corthycyclon*).

• *Corthyloxiphus obesus* Wood, 2007: 794.

• *Corthyloxiphus punctatus* Wood, 2007: 792.

• *Corthyloxiphus reticulatus* Wood, 2007: 794.

• *Corthyloxiphus simplicus* Wood, 2007: 794.

• *Corthyloxiphus tardus* (Wood, 1974b: 149) (*Corthycyclon tardum*).

• *Corthyloxiphus truncatus* Wood, 2007: 791.

• *Corthyloxiphus usticus* Wood, 2007: 793.

• *Corthyloxiphus willei* Wood, 2007: 791.

#### *Corthylus* Erichson

170 spp.

• *Corthylus abbreviatus* Eichhoff, 1869: 279.

= *Corthylus dentatus* Eggers, 1943d: 382.

• *Corthylus abruptedeclivis* Schedl, 1966d: 121.

• *Corthylus additus* Wood, 1974c: 199.

• *Corthylus alienus* Schedl, 1966d: 119.

• *Corthylus alpestris* Bright, 2019: 400.

• *Corthylus andinus* Wood, 2007: 852.

• *Corthylus annexus* Wood, 2007: 835.

• *Corthylus antennarius* Schedl, 1966d: 120.

• *Corthylus araguensis* Wood, 2007: 843.

• *Corthylus ater* Schedl, 1952e: 347 (RN).

= *Corthylus columbianus* Schedl, 1950c: 158.

• *Corthylus atomus* Wood, 2007: 847.

• *Corthylus attenuatus* Wood, 2007: 857.

• *Corthylus bellus* Wood, 2007: 861.

• *Corthylus bifurcus* Schedl, 1935f: 345.

• *Corthylus bolivianus* Eggers, 1943d: 379.

• *Corthylus brunnescens* Wood, 1981: 122 (RN).

= *Corthylus brunneus* Wood, 1974c: 188.

• *Corthylus burgosi* Atkinson, 2020: 19.

• *Corthylus calamarius* Wood, 1974c: 185.

• *Corthylus callidus* Schedl, 1973b: 172.

• *Corthylus calmicolens* Wood, 1974c: 189.

• *Corthylus cannularius* Wood, 1974c: 186.

• *Corthylus castaneus* (Ferrari, 1867: 55) (*Pseudocorthylus*).

• *Corthylus cecropicolens* Wood, 2007: 825.

• *Corthylus cecropii* Wood, 1975a: 31.

• *Corthylus chiriquensis* Wood, 2007: 835.

• *Corthylus cirrifer* Wood, 2007: 856.

• *Corthylus cirritus* Wood, 1974c: 200.

• *Corthylus coffeae* Wood, 2007: 829.

• *Corthylus collaris* Blandford, 1904: 261.

= *Corthylus splendens* Wood, 1967c: 138.

• *Corthylus columbianus* Hopkins, 1895: 104.

• *Corthylus comatus* Blandford, 1904: 258.

= *Corthylus splendidus* Bright, 1972b: 1371.

• *Corthylus comitabilis* Wood, 2007: 845.

• *Corthylus comosus* Wood, 1974c: 186.

• *Corthylus compressicornis* (Fabricius, 1801: 388) (*Bostrichus*).

• *Corthylus concavus* Bright, 1972b: 1376.

• *Corthylus concisus* Wood, 1974c: 201.

• *Corthylus confertus* Wood, 2007: 841.

• *Corthylus confusus* Wood, 2007: 833.

• *Corthylus consimilis* Wood, 1974c: 188.

• *Corthylus convexicauda* Eggers, 1931b: 40.

= *Corthylus bituberculatus* Nunberg, 1962: 229.

• *Corthylus convexifrons* Wood, 1986a: 270.

• *Corthylus coronatus* Eggers, 1933f: 21.

• *Corthylus costulatus* Wood, 2007: 828.

• *Corthylus crassus* Wood, 2007: 834.

• *Corthylus cristatulus* Atkinson, 2020: 9.

• *Corthylus cristatus* Atkinson, 2020: 6.

• *Corthylus curiosus* Bright, 1972a: 104.

• *Corthylus detrimentosus* Schedl, 1940a: 355.

• *Corthylus diligens* Wood, 1974c: 190.

• *Corthylus discoideus* Blandford, 1904: 262.

• *Corthylus donaticus* Wood, 1974c: 198.

• *Corthylus dubiosus* (Schedl, 1976: 81) (*Metacorthylus*).

• *Corthylus eichhoffi* Schedl, 1933a: 34.

• *Corthylus electinus* Wood, 2007: 833.

• *Corthylus epistomalis* Wood, 2007: 846.

• *Corthylus equihuai* Wood, 2007: 830.

• *Corthylus excisus* (Ferrari, 1867: 71) (*Morizus*).

• *Corthylus exiguus* Wood, 1984a: 113.

• *Corthylus flagellifer* Blandford, 1904: 255.

= *Corthylus cirrus* Schedl, 1940a: 351.

= *Corthylus nudiusculus* Schedl, 1950c: 156.

• *Corthylus frontalis* Wood, 2007: 863.

• *Corthylus fuscus* Blandford, 1904: 262.

• *Corthylus garai* Wood, 2007: 826.

• *Corthylus gracilens* Wood, 2007: 860.

• *Corthylus gracilior* Wood, 2007: 860.

• *Corthylus gracilis* (Schedl, 1972f: 74) (*Corthylomimus*).

• *Corthylus granulatus* Schedl, 1935f: 345.

• *Corthylus granulifer* Wood, 1974c: 181.

• *Corthylus granulocristatus* Atkinson, 2020: 10.

• *Corthylus granulosus* Atkinson, 2020: 20.

• *Corthylus ibarrai* Atkinson, 2020: 14.

• *Corthylus ingaensis* (Schedl, 1939g: 14) (*Metacorthylus*).

• *Corthylus insignis* Wood, 1974c: 200.

• *Corthylus insularis* Bright & Torres, 2006: 424.

• *Corthylus latisetosus* Atkinson, 2020: 16.

• *Corthylus letzneri* (Ferrari, 1867: 59) (*Pseudocorthylus*).

= *Corthylus strigillatus* Eggers, 1933f: 20.

• *Corthylus luridus* Blandford, 1904: 256.

= *Corthylus biseriatus* Eggers, 1943a: 247.

• *Corthylus lustratus* Wood, 1984a: 113.

• *Corthylus macrocerus* Eichhoff, 1869: 279.

• *Corthylus merkli* Wood, 2007: 866.

• *Corthylus mexicanus* Schedl, 1950c: 159.

= *Corthylus glabinus* Bright, 1972b: 1372.

• *Corthylus micacirrus* Wood, 1984a: 114.

• *Corthylus microcorthyloides* Atkinson, 2020: 22.

• *Corthylus minimus* Wood, 1974c: 192.

• *Corthylus minulus* Wood, 2007: 827.

• *Corthylus minutissimus* Schedl, 1940a: 353.

• *Corthylus minutus* Bright, 1972b: 1373.

• *Corthylus mirabilis* Nunberg, 1962: 226.

• *Corthylus montanus* Wood, 2007: 833.

• *Corthylus monticellus* Bright, 2019: 402.

• *Corthylus nanus* Wood, 1979: 138.

• *Corthylus nevermanni* (Schedl, 1939d: 568) (*Thylurcos*).

• *Corthylus niger* (Schedl, 1954b: 43) (*Metacorthylus*).

• *Corthylus nigrescens* Wood, 2007: 836.

• *Corthylus nigricans* Wood, 2007: 839.

• *Corthylus noguerai* Wood, 2007: 837.

• *Corthylus nolenae* Wood, 1974c: 194.

• *Corthylus nudus* Schedl, 1940a: 354.

• *Corthylus oculatus* Wood, 1974c: 184.

• *Corthylus panamensis* Blandford, 1904: 259.

• *Corthylus papuellus* Wood, 2007: 855.

• *Corthylus papulans* Eichhoff, 1869: 280.

= *Corthylus spinifer* Schwarz, 1891: 114.

= *Metacorthylus affinis* Fonseca, 1925: 3.

= *Corthylus guayanensis* Eggers, 1933f: 22.

= *Corthylus tomentosus* Schedl, 1940a: 350.

= *Corthylus brunneus* Nunberg, 1972: 191.

• *Corthylus parvicirrus* Wood, 2007: 844.

• *Corthylus parvulus* Blandford, 1904: 261.

= *Corthylus uniseptis* Schedl, 1961h: 229.

= *Corthylus reburrus* Bright, 1972b: 1375.

• *Corthylus peruanus* Schedl, 1950c: 155.

• *Corthylus petilus* Wood, 1967a: 56.

• *Corthylus pharax* Schedl, 1976: 80.

• *Corthylus pilifer* Wood, 2007: 831.

• *Corthylus pinguis* Wood, 2007: 826.

• *Corthylus poblanus* Atkinson, 2020: 12.

• *Corthylus praealtus* Schedl, 1976: 80.

• *Corthylus praeustus* Schedl, 1950c: 153.

• *Corthylus procerus* Bright, 1972b: 1376.

• *Corthylus pseudoandinus* Wood, 2007: 852.

• *Corthylus pseudoexcisus* Wood, 2007: 853.

• *Corthylus pseudovillus* Wood, 2007: 831.

• *Corthylus ptyocerus* Blandford, 1904: 257.

• *Corthylus pumilus* Wood, 1974c: 192.

• *Corthylus punctatissimus* (Zimmermann, 1868: 144) (*Crypturgus*).

• *Corthylus punctatus* Eggers, 1943d: 382.

= *Corthylus nudipennis* Schedl, 1950c: 155.

= *Corthylus cavifrons* Nunberg, 1962: 230.

= *Corthylus oliveirai* Schedl, 1976: 80.

= *Corthycyclon tardus* Schedl, 1976: 85.

= *Corthycyclon neotardus* Schedl, 1980c: 120 (RN).

= *Corthycyclon tardulus* Wood, 1981: 122 (RN).

• *Corthylus punctifrons* Wood, 2007: 848.

• *Corthylus pusillus* Eggers, 1943d: 380.

• *Corthylus pygmaeus* Wood, 1974c: 195.

• *Corthylus redtenbacheri* (Ferrari, 1867: 55) (*Pseudocorthylus*).

• *Corthylus reticulatus* Bright, 2019: 404.

• *Corthylus retusifer* Wood, 1974c: 183.

• *Corthylus retusus* Wood, 1974c: 182.

• *Corthylus robustus* Schedl, 1936a: 108.

• *Corthylus rubricollis* Blandford, 1904: 260.

• *Corthylus rufopilosus* Eggers, 1931b: 39.

• *Corthylus sanguineus* Schedl, 1935f: 346.

• *Corthylus schaufussi* Schedl, 1937a: 69.

• *Corthylus schulzi* Wood, 2007: 865.

• *Corthylus senticosus* Wood, 1986a: 271.

• *Corthylus sentosus* Wood, 1986a: 271.

• *Corthylus sentus* Wood, 1974c: 195.

• *Corthylus serratus* Wood, 1974c: 197.

• *Corthylus serrulatus* Eggers, 1934a: 82.

= *Corthylus argentinensis* Schedl, 1950c: 157.

• *Corthylus simillimus* Schedl, 1966d: 118.

• *Corthylus simplex* Wood, 1974c: 187.

• *Corthylus simplicis* Wood, 2007: 832.

• *Corthylus sobrinus* Wood, 1974c: 196.

• *Corthylus spinipennis* Wood, 2007: 850.

• *Corthylus spinosus* Wood, 1974c: 194.

• *Corthylus spinulosus* Atkinson, 2020: 2.

• *Corthylus splendidulus* Wood, 2007: 845.

• *Corthylus strigilis* Wood, 1974c: 189.

• *Corthylus subasperulus* Eggers, 1940a: 141.

• *Corthylus subserratus* Wood, 1974c: 197.

• *Corthylus subsulcatus* Schedl, 1961h: 230.

• *Corthylus suturalis* Eggers, 1931b: 41.

= *Corthylus obliquus* Schedl, 1976: 79.

• *Corthylus suturifer* Schedl, 1963c: 165.

• *Corthylus theobromae* Nunberg, 1971: 59.

• *Corthylus transversus* Eichhoff, 1869: 279.

• *Corthylus trucis* Wood, 1974c: 193.

• *Corthylus truncatiformus* Wood, 2007: 847.

• *Corthylus truncatus* Wood, 1985: 272.

• *Corthylus trunculus* Wood, 1974c: 191.

• *Corthylus tuberculatus* Eggers, 1940a: 140.

• *Corthylus tuberculifer* Wood, 2007: 849.

• *Corthylus tuberosus* Wood, 2007: 849.

• *Corthylus tulcanus* Hagedorn, 1910: 6.

• *Corthylus ustus* (Schedl, 1951c: 128) (*Corthycyclon ustum*).

• *Corthylus venustus* (Schedl, 1951c: 127) (*Thylurcos*).

• *Corthylus versicolor* Bright, 2019: 407.

• *Corthylus villifer* Wood, 1974c: 183.

• *Corthylus villosus* Eggers, 1943d: 381.

• *Corthylus villus* Bright, 1972b: 1371.

• *Corthylus vochysiae* Wood, 2007: 847.

• *Corthylus zelus* Wood, 1974c: 190.

• *Corthylus zulmae* Wood, 2007: 842.

#### *Glochinocerus* Blandford

2 spp.

• *Glochinocerus gemellus* Blandford, 1904: 267.

• *Glochinocerus retusipennis* Blandford, 1904: 266.

#### *Gnatharus* Wood & Yin

1 sp.

• *Gnatharus tibetensis* Wood & Yin, 1986: 463.

#### *Gnathotrichus* Eichhoff

17 spp.

• *Gnathotrichus alniphagus* Wood, 1984a: 115.

• *Gnathotrichus consentaneus* Blandford, 1904: 247.

• *Gnathotrichus deleoni* Blackman, 1942b: 227.

• *Gnathotrichus dentatus* Wood, 1967a: 45.

• *Gnathotrichus denticulatus* Blackman, 1931b: 270.

• *Gnathotrichus hispaniolus* Bright, 2019: 408.

• *Gnathotrichus imitans* Wood, 1967a: 48.

• *Gnathotrichus materiarius* (Fitch, 1858: 726) (*Tomicus*).

= *Gnathotrichus corthyloides* Eichhoff, 1869: 275.

= *Xyleborus duprezi* Hoffmann, 1936: 43.

• *Gnathotrichus nimifrons* Wood, 1967a: 47.

• *Gnathotrichus nitidifrons* Hopkins, 1906: 72.

• *Gnathotrichus obscurus* Wood, 1974a: 55.

• *Gnathotrichus omissus* Wood, 1974a: 56.

• *Gnathotrichus perniciosus* Wood, 1967a: 47.

• *Gnathotrichus pilosus* (LeConte, 1868: 156) (*Cryphalus*).

= *Ancyloderes saltoni* Blackman, 1938c: 206.

• *Gnathotrichus primus* (Bright, 1972d: 1678) (*Prognathotrichus*).

• *Gnathotrichus retusus* (LeConte, 1868: 155) (*Cryphalus*).

= *Gnathotrichus alni* Blackman, 1931b: 271.

• *Gnathotrichus sulcatus* (LeConte, 1868: 155) (*Cryphalus*).

= *Gnathotrichus aciculatus* Blackman, 1931b: 272.

#### *Gnathotrupes* Schedl

28 spp.

• *Gnathotrupes barbifer* (Schedl, 1967d: 13) (*Gnathotrichus*).

= *Gnathotrupes similis* Schedl, 1975k: 10.

• *Gnathotrupes bituberculatus* (Blandford, 1904: 248) (*Gnathotrichus*).

= *Gnathotrichus impressus* Schedl, 1977d: 44.

• *Gnathotrupes bolivianus* Schedl, 1951c: 126.

• *Gnathotrupes caliculus* (Schedl, 1975k: 12) (*Gnathocortus*).

• *Gnathotrupes cirratus* Schedl, 1975k: 5.

• *Gnathotrupes colaphus* Wood, 1989: 181.

• *Gnathotrupes consobrinus* (Eichhoff, 1878b: 409) (*Gnathotrichus*).

= *Gnathotrichus obnixus* Schedl, 1939a: 47.

= *Gnathotrichus quadrituberculatus* Schedl, 1951c: 122.

= *Gnathotrichus sextuberculatus* Schedl, 1951c: 118.

= *Gnathotrichus corthyliodes* Schedl, 1952i: 20.

• *Gnathotrupes crecentus* Wood, 1974a: 56.

• *Gnathotrupes dilutus* Wood, 1974a: 56.

• *Gnathotrupes electus* (Wood, 1968a: 10) (*Gnathotrypanus*).

• *Gnathotrupes emarginatus* Wood, 2007: 661.

• *Gnathotrupes fimbriatus* (Schedl, 1955h: 259) (*Gnathotrichus*).

= *Gnathotrichus frontalis* Schedl, 1972g: 146.

• *Gnathotrupes herbertfranzi* (Schedl, 1972g: 147) (*Gnathotrichus*).

• *Gnathotrupes impressus* (Schedl, 1975k: 17) (*Gnathoglochinus*).

= *Gnathotrupes pauciconvacus* Schedl, 1975k: 7.

• *Gnathotrupes kirkendalli* Wood, 2007: 668.

• *Gnathotrupes longicollis* (Schedl, 1951c: 120) (*Gnathotrichus*).

• *Gnathotrupes longipennis* (Blanchard, 1851: 429) (*Tomicus*).

= *Gnathotrichus corthyloides* Schedl, 1952i: 21.

= *Gnathotrichus corthyliformis* Schedl, 1964g: 312 (RN).

= *Gnathotrupes constrictus* Schedl, 1975k: 6.

• *Gnathotrupes longiusculus* (Schedl, 1951c: 121) (*Gnathotrichus*).

= *Gnathotrupes ciliatus* Schedl, 1975k: 4.

• *Gnathotrupes moraviae* Wood, 2007: 662.

• *Gnathotrupes nanus* (Eichhoff, 1878b: 410) (*Gnathotrichus*).

= *Gnathotrichus nanulus* Schedl, 1972g: 149.

• *Gnathotrupes naumanni* (Schedl, 1975k: 15) (*Gnathomimus*).

• *Gnathotrupes nectandrae* Wood, 1989: 181.

• *Gnathotrupes nothofagi* (Schedl, 1975k: 13) (*Gnathomimus*).

• *Gnathotrupes pustulatus* Schedl, 1975k: 9.

• *Gnathotrupes sericeus* (Schedl, 1980a: 160) (*Eidognathus*).

• *Gnathotrupes terebratus* (Wood, 1968a: 9) (*Gnathotrypanus*).

• *Gnathotrupes vafer* (Schedl, 1975k: 3) (*Gnathotrichus*).

• *Gnathotrupes velatus* Schedl, 1975k: 10.

= *Gnathotrupes solidus* Schedl, 1975k: 10.

#### *Metacorthylus* Blandford

13 spp.

• *Metacorthylus concisus* (Wood, 1974a: 67) (*Paracorthylus*).

• *Metacorthylus costatulus* Wood, 2007: 771.

• *Metacorthylus granosus* Wood, 2007: 769.

• *Metacorthylus mutilus* (Wood, 1974a: 66) (*Paracorthylus*).

• *Metacorthylus nigripennis* Blandford, 1904: 263.

• *Metacorthylus obscuriceps* Wood, 2007: 769.

• *Metacorthylus subcostulatus* Wood, 2007: 771.

• *Metacorthylus subpronum* (Schedl, 1976: 83) (*Pterocyclon*).

• *Metacorthylus subtruncatus* (Schedl, 1978a: 303) (*Pterocyclon subtruncatum*).

• *Metacorthylus truncatorus* (Schedl, 1950c: 175) (*Amphicranus*).

• *Metacorthylus velutinus* (Wood, 1968a: 7) (*Paracorthylus*).

• *Metacorthylus vicinus* (Schedl, 1970e: 100) (*Pterocyclon vicinum*).

• *Metacorthylus volvulus* (Eichhoff, 1869: 279) (*Pterocyclon volvulum*).

#### *Microcorthylus* Ferrari

40 spp.

• *Microcorthylus absonus* Wood, 2007: 783.

• *Microcorthylus bicolor* Eggers, 1935c: 154.

• *Microcorthylus brevior* Wood, 2007: 781.

• *Microcorthylus brevis* Eggers, 1935c: 155.

• *Microcorthylus concisus* Wood, 1973: 270.

• *Microcorthylus curtus* Wood, 1973: 274.

• *Microcorthylus debilis* Wood, 1973: 265.

• *Microcorthylus declivis* Wood, 2007: 781.

• *Microcorthylus degener* Wood, 2007: 776.

• *Microcorthylus demissus* Wood, 1973: 266.

• *Microcorthylus dilutus* Wood, 1973: 271.

• *Microcorthylus diversus* Wood, 1973: 272.

= *Microcorthylus hostilis* Wood, 1973: 272.

• *Microcorthylus glabratus* (Ferrari, 1867: 60) (*Pseudocorthylus*).

= *Microcorthylus pallidus* Schedl, 1939d: 571.

= *Microcorthylus subopacus* Schedl, 1939d: 573.

• *Microcorthylus grandiclavatus* Eggers, 1935c: 156.

• *Microcorthylus inops* Wood, 2007: 780.

• *Microcorthylus insularis* Bright, 2019: 413.

• *Microcorthylus invalidus* Wood, 1973: 268.

• *Microcorthylus lassus* Wood, 1973: 270.

• *Microcorthylus macer* Wood, 2007: 780.

• *Microcorthylus mexicanus* Eggers, 1934a: 81.

• *Microcorthylus minimus* Schedl, 1950c: 160.

• *Microcorthylus minutissimus* Schedl, 1952e: 361.

• *Microcorthylus nebulosus* Wood, 2007: 779.

• *Microcorthylus obscuriceps* Wood, 2007: 778.

• *Microcorthylus obscurus* Eggers, 1935c: 155.

• *Microcorthylus ocularis* Wood, 1973: 266.

• *Microcorthylus parvulus* Ferrari, 1867: 58.

= *Pterocyclon exile* Eichhoff, 1878b: 451.

= *Microcorthylus inermis* Wood, 1973: 267.

• *Microcorthylus parvus* Wood, 2007: 784.

• *Microcorthylus puerulus* Schedl, 1939d: 571.

= *Microcorthylus castaneus* Schedl, 1939d: 572.

= *Microcorthylus porrectus* Schedl, 1951a: 292.

= *Microcorthylus contractus* Wood, 1973: 274.

• *Microcorthylus pumilus* Wood, 1973: 268.

• *Microcorthylus pusillus* Wood, 1973: 269.

• *Microcorthylus quadridens* Wood, 2007: 782.

• *Microcorthylus rufotestaceus* Schedl, 1939d: 572.

• *Microcorthylus simulans* Wood, 2007: 782.

• *Microcorthylus suggrandis* Schedl, 1939d: 574.

• *Microcorthylus tuberculifer* Wood, 2007: 777.

• *Microcorthylus umbratus* Wood, 1973: 273.

• *Microcorthylus vescus* Wood, 1973: 271.

• *Microcorthylus vicinus* Wood, 1977a: 213.

• *Microcorthylus vietus* Wood, 2007: 777.

#### *Monarthrum* Kirsch

146 spp.

• *Monarthrum aberrans* Schedl, 1977d: 46.

• *Monarthrum abruptum* (Nunberg, 1962: 233) (*Microcorthylus abruptus*).

• *Monarthrum ambiguum* Bright, 2019: 416.

• *Monarthrum amparae* Wood, 2007: 746 (RN).

= *Mimips brasiliensis* Schedl, 1976: 72.

• *Monarthrum amphicranoides* (Schedl, 1963f: 225) (*Pterocyclon*).

• *Monarthrum annulatum* Wood, 2007: 763.

• *Monarthrum antillicum* Bright, 2019: 416.

• *Monarthrum aztecum* Wood, 2007: 745.

• *Monarthrum bicallosum* (Schedl, 1939d: 577) (*Pterocyclon*).

• *Monarthrum bicavum* Wood, 1967a: 51.

• *Monarthrum bicolor* (Ferrari, 1867: 56) (*Corthylus*).

= *Corthylus signatus* Ferrari, 1867: 56.

= *Phthorius edentatus* Hagedorn, 1905a: 549.

= *Monarthrum sexdentatum* Eggers, 1935b: 83.

• *Monarthrum bicoloratum* Wood, 1974d: 284 (RN).

= *Monarthrum bicolor* Wood, 1968a: 4.

• *Monarthrum bidens* (Blandford, 1904: 277) (*Pterocyclon*).

• *Monarthrum bidentatum* Wood, 1974b: 142.

• *Monarthrum bifoveatum* Wood, 1974b: 137.

• *Monarthrum bispinum* (Blandford, 1905: 281) (*Pterocyclon*).

• *Monarthrum bituberculatum* Wood, 2007: 760.

• *Monarthrum boliviense* Wood, 1989: 178 (RN) (E).

= *Cosmocorynus bolivianus* Schedl, 1970e: 103.

• *Monarthrum brittoni* (Schedl, 1970e: 101) (*Pterocyclon*).

• *Monarthrum brunneum* (Eichhoff, 1869: 278) (*Pterocyclon*).

= *Corthylus plagiatus* Eichhoff, 1869: 279.

• *Monarthrum bullatum* Bright, 2019: 418.

• *Monarthrum canalis* Wood, 2007: 759.

• *Monarthrum carinatum* Wood, 1974b: 139.

• *Monarthrum carinifrons* Wood, 2007: 761.

• *Monarthrum carinulum* Wood, 1974b: 144.

• *Monarthrum catarinense* Wood, 2007: 762 (E).

• *Monarthrum chapuisi* Kirsch, 1866: 213.

= *Monarthrum bolivianum* Eggers, 1935b: 80.

• *Monarthrum cincinnatum* (Eichhoff, 1878b: 435) (*Trypocranus cincinnatus*).

• *Monarthrum collinum* Bright, 2019: 419.

• *Monarthrum connexum* Wood, 2007: 751.

• *Monarthrum consimile* (Blandford, 1904: 275) (*Pterocyclon*).

= *Pterocyclon pseudosulcatum* Schedl, 1935f: 348.

• *Monarthrum conversum* Wood, 1974b: 138.

• *Monarthrum corculum* Wood, 1974b: 146.

• *Monarthrum cordatum* (Blandford, 1904: 279) (*Pterocyclon*).

• *Monarthrum cordicticum* Wood, 1974b: 135.

• *Monarthrum costatum* (Eggers, 1937b: 85) (*Brachyspartus costatus*).

• *Monarthrum cristatum* (Ferrari, 1867: 64) (*Cosmocorynus cristatus*).

• *Monarthrum dentatulum* Wood, 1989: 178 (RN).

= *Monarthrum dentatum* Eggers, 1935b: 84.

• *Monarthrum dentatum* (Eggers, 1931a: 19) (*Amphicranus dentatus*).

• *Monarthrum denticulatum* Wood, 1981: 122 (RN).

= *Pterocyclon dentatum* Eggers, 1941a: 101.

• *Monarthrum dentifrons* Wood, 2007: 758.

• *Monarthrum dentigerum* (LeConte, 1868: 154) (*Cryphalus dentiger*).

• *Monarthrum desum* (Wood, 1967a: 52) (*Microcorthylus desus*).

• *Monarthrum difficile* (Blandford, 1904: 276) (*Pterocyclon*).

• *Monarthrum diligens* Wood, 2007: 764.

• *Monarthrum dimidiatum* (Ferrari, 1867: 57) (*Corthylus*).

= *Pterocyclon moritzi* Schedl, 1939h: 727.

• *Monarthrum discordum* Bright, 2019: 419.

• *Monarthrum dolosum* Wood, 2007: 731.

• *Monarthrum dubiosum* (Schedl, 1976: 82) (*Pterocyclon*).

• *Monarthrum duplocordatum* Eggers, 1935b: 86.

• *Monarthrum durangoense* Wood, 2007: 757 (E).

• *Monarthrum egenum* (Blandford, 1904: 280) (*Pterocyclon*).

= *Brachyspartus bisetosus* Schedl, 1954b: 38.

• *Monarthrum eggersi* Wood, 2007: 743.

• *Monarthrum elegans* (Eichhoff, 1869: 277) (*Pterocyclon*).

• *Monarthrum exornatum* (Schedl, 1939d: 575) (*Pterocyclon*).

= *Pterocyclon gracilicornum* Schedl, 1939d: 576.

• *Monarthrum fasciatum* (Say, 1826: 255) (*Bostrichus fasciatus*).

= *Pterocyclon simile* Eichhoff, 1869: 277.

= *Pterocyclon gracile* Eichhoff, 1878b: 444.

• *Monarthrum fastigiorum* Wood, 1974b: 141.

• *Monarthrum fenestratum* Eggers, 1935b: 85.

• *Monarthrum ferrarii* (Blandford, 1905: 284) (*Pterocyclon*).

• *Monarthrum ferrugineum* Bright, 2019: 420.

• *Monarthrum fimbriaticorne* (Blandford, 1905: 285) (*Pterocyclon*).

= *Pterocyclon turbinatum* Schedl, 1961h: 230.

= *Pterocyclon obliquum* Schedl, 1970e: 99.

• *Monarthrum flohri* (Schedl, 1950c: 168) (*Pterocyclon*).

• *Monarthrum fulgens* Schedl, 1971f: 153.

• *Monarthrum furnissi* Wood, 2007: 756.

• *Monarthrum glabrifrons* (Blandford, 1904: 278) (*Pterocyclon*).

• *Monarthrum gnarum* (Schedl, 1950c: 169) (*Pterocyclon*).

= *Amphicranus spinatus* Bright, 1972b: 1383.

• *Monarthrum gracilentum* (Schedl, 1972f: 75) (*Pterocyclon*).

• *Monarthrum gracilius* (Schedl, 1959b: 553) (*Pterocyclon gracilior*).

= *Pterocyclon pennatum* Schedl, 1963c: 167.

= *Pterocyclon glabriculum* Schedl, 1976: 82.

• *Monarthrum granosum* Wood, 2007: 764.

• *Monarthrum granulatum* Bright, 1972b: 1382.

• *Monarthrum granulifer* Wood, 2007: 756.

• *Monarthrum granulosum* Wood, 2007: 761.

• *Monarthrum hagedorni* (Schedl, 1939h: 727) (*Pterocyclon*) (RN).

= *Pterocyclon dimidiatum* Hagedorn, 1905a: 550.

• *Monarthrum hoegei* (Blandford, 1904: 274) (*Pterocyclon*).

= *Monarthrum oaxacaensis* Bright, 1972b: 1382.

• *Monarthrum hondurense* Wood, 2007: 756 (E).

• *Monarthrum huachucae* Wood, 1959b: 61.

• *Monarthrum infradentatum* Wood, 1974b: 145.

• *Monarthrum ingens* (Eichhoff, 1869: 278) (*Pterocyclon*).

= *Anchonocerus rufipes* Eichhoff, 1878b: 431.

= *Anchonocerus excavatus* Eggers, 1935d: 330.

= *Pterocyclon assequens* Schedl, 1978a: 302.

• *Monarthrum insidiosum* Wood, 2007: 730.

• *Monarthrum insignatum* Wood, 1974b: 141.

• *Monarthrum insolitum* (Schedl, 1976: 83) (*Pterocyclon*).

• *Monarthrum intermedium* Schedl, 1970e: 105.

• *Monarthrum laevigatum* (Eichhoff, 1869: 278) (*Pterocyclon*).

= *Pterocyclon adjunctum* Schedl, 1967d: 15.

• *Monarthrum laterale* (Eichhoff, 1869: 278) (*Pterocyclon*).

= *Cosmocorynus trifasciatus* Schedl, 1950c: 173.

= *Pterocyclon durum* Schedl, 1972f: 74.

• *Monarthrum limulum* Wood, 1974b: 143.

• *Monarthrum lobatum* (Ferrari, 1867: 57) (*Corthylus*).

• *Monarthrum lobellum* Wood, 2007: 737.

• *Monarthrum longus* (Schedl, 1950c: 172) (*Anchonocerus*).

• *Monarthrum luctuosum* (Blandford, 1904: 276) (*Pterocyclon*).

• *Monarthrum mali* (Fitch, 1856: 326) (*Tomicus*).

= *Pterocyclon longulum* Eichhoff, 1869: 278.

• *Monarthrum marcidium* (Schedl, 1970e: 98) (*Pterocyclon*).

• *Monarthrum marginatum* Wood, 2007: 760.

• *Monarthrum meuseli* (Reitter, 1905: 249) (*Xyleborus*).

• *Monarthrum minutissimum* (Schedl, 1954b: 40) (*Pterocyclon*).

= *Microcorthylus schedli* Bright, 1972a: 102 (RN).

• *Monarthrum minutum* (Schedl, 1939d: 577) (*Pterocyclon*).

= *Pterocyclon gibber* Schedl, 1952a: 460.

= *Pterocyclon vernaculum* Schedl, 1952a: 460.

= *Pterocyclon distans* Schedl, 1970e: 97.

• *Monarthrum morsum* Wood, 1974b: 139.

• *Monarthrum nevermanni* (Schedl, 1935f: 348) (*Pterocyclon*).

• *Monarthrum notatum* Wood, 1974b: 143.

• *Monarthrum nudum* (Schedl, 1939d: 574) (*Pterocyclon*).

= *Pterocyclon appendicinum* Schedl, 1967d: 14.

• *Monarthrum obesum* (Schedl, 1970e: 99) (*Pterocyclon*).

• *Monarthrum obscuriceps* Wood, 2007: 757.

• *Monarthrum obscurum* Wood, 2007: 757.

• *Monarthrum obtusum* (Eggers, 1935d: 334) (*Anchonocerus*).

= *Anchonocerus quadridentatus* Eggers, 1935d: 333.

• *Monarthrum parvum* (Eggers, 1933f: 22) (*Anchonocerus parvus*).

= *Monarthrum preclarum* Wood, 1968a: 6.

• *Monarthrum penicillatum* (Eichhoff, 1878b: 457) (*Pterocyclon*).

• *Monarthrum peruanum* (Schedl, 1950c: 168) (*Pterocyclon*).

• *Monarthrum peruvianum* Wood, 1981: 122 (RN).

= *Monarthrum peruanum* Schedl, 1978a: 306.

• *Monarthrum plaumanni* (Schedl, 1937a: 68) (*Pterocyclon*).

• *Monarthrum posticum* Wood, 1974b: 146.

• *Monarthrum praeruptum* (Blandford, 1904: 273) (*Pterocyclon*).

• *Monarthrum praeustum* (Eggers, 1941a: 100) (*Pterocyclon*).

= *Pterocyclon opacifrons* Schedl, 1950c: 167.

= *Pterocyclon omissum* Schedl, 1952e: 347 (RN).

= *Monarthrum omissum* Schedl, 1952e: 347 (RN).

• *Monarthrum proprium* Wood, 1974b: 136.

• *Monarthrum proximum* Wood, 1974b: 147.

• *Monarthrum pseudoparvum* Wood, 2007: 744.

• *Monarthrum pseudoscutellare* (Schedl, 1935f: 349) (*Pterocyclon*).

= *Monarthrum adustum* Wood, 1974b: 140.

• *Monarthrum pumilio* (Eichhoff, 1878b: 445) (*Pterocyclon*).

• *Monarthrum punctifrons* (Blandford, 1904: 278) (*Pterocyclon*).

• *Monarthrum quadridens* (Eichhoff, 1869: 277) (*Pterocyclon*).

= *Pterocyclon dubium* Eichhoff, 1869: 277.

= *Anchonocerus brasiliensis* Schedl, 1936a: 107.

= *Pterocyclon eumerum* Schedl, 1952a: 459.

• *Monarthrum quercicolens* Wood, 1967a: 49.

• *Monarthrum quercivorum* Schedl, 1977d: 46.

• *Monarthrum quercum* (Wood, 1967a: 53) (*Amphicranus quercus*).

• *Monarthrum querneum* Wood, 1967a: 50.

= *Monarthrum bifidus* Bright, 1972b: 1381.

• *Monarthrum robustum* (Schedl, 1966d: 123) (*Pterocyclon*).

• *Monarthrum scrobiceps* (Eichhoff, 1878b: 458) (*Pterocyclon*).

= *Eupteroxylon comatum* Eggers, 1936d: 393.

= *Cosmocorynus latum* Schedl, 1966d: 126.

= *Cosmocorynus vagabundus* Schedl, 1966d: 124.

• *Monarthrum scutellare* (LeConte, 1857: 59) (*Corthylus scutellaris*).

= *Cryphalus cavus* LeConte, 1868: 153.

= *Pterocyclon obliquecaudatum* Schedl, 1935f: 351.

• *Monarthrum semipallens* (Schedl, 1954b: 41) (*Pterocyclon*).

• *Monarthrum semitruncatum* Wood, 2007: 763.

• *Monarthrum septulosum* Wood, 2007: 750.

• *Monarthrum subcarinatum* Wood, 2007: 758.

• *Monarthrum subductum* (Schedl, 1978a: 303) (*Pterocyclon*).

• *Monarthrum subgranulatum* Wood, 1974b: 144.

• *Monarthrum subimpressum* Wood, 2007: 748.

• *Monarthrum sulcatum* (Blandford, 1905: 284) (*Pterocyclon*).

• *Monarthrum sulcipenne* (Schedl, 1978a: 304) (*Pterocyclon*).

• *Monarthrum surinamense* Wood, 2007: 762 (E).

• *Monarthrum terminalis* Wood, 2007: 764.

• *Monarthrum terminatum* (Blandford, 1904: 280) (*Pterocyclon*).

• *Monarthrum tetradontium* Wood, 1974b: 137.

• *Monarthrum tomicoides* (Blandford, 1904: 273) (*Pterocyclon*).

• *Monarthrum tridentatum* (Schedl, 1971f: 152) (*Cosmocorynus*).

• *Monarthrum tuberculatum* Wood, 2007: 747.

• *Monarthrum umbrinum* (Blandford, 1904: 275) (*Pterocyclon*).

• *Monarthrum unifasciatum* (Schedl, 1970e: 104) (*Cosmocorynus unifasciatus*).

• *Monarthrum validum* (Ferrari, 1867: 55) (*Corthylus*).

= *Amphicranus mexicanus* Eggers, 1931a: 18.

= *Pterocyclon jalapae* Schedl, 1939d: 584.

• *Monarthrum vittatum* (Blandford, 1905: 282) (*Pterocyclon*).

• *Monarthrum xalapense* Wood, 1987: 548 (E).

#### *Tricolus* Blandford

55 spp.

• *Tricolus abacis* Wood, 2007: 686.

• *Tricolus abruptus* Schedl, 1976: 83.

• *Tricolus aciculatus* Wood, 1974a: 62.

• *Tricolus affinis* Eggers, 1931b: 38.

• *Tricolus amplus* Wood, 1974a: 63.

• *Tricolus angustatus* Wood, 2007: 679.

• *Tricolus animatus* Bright, 2019: 423.

• *Tricolus ardis* Wood, 1974a: 58.

• *Tricolus badius* Wood, 1974a: 60.

• *Tricolus bicavus* Wood, 2007: 683.

• *Tricolus bicolor* Wood, 1974a: 63.

• *Tricolus bifidus* Schedl, 1939d: 579.

• *Tricolus brasilianus* Wood, 2007: 687.

• *Tricolus capitalis* Wood, 1974a: 61.

• *Tricolus cecropii* Wood, 1974a: 58.

• *Tricolus collaris* (Blandford, 1905: 294) (*Amphicranus*).

• *Tricolus coloreus* Wood, 2007: 688.

• *Tricolus cuahuitli* Burgos, Hernández & Burgos, 2024: 530

• *Tricolus difodinus* Bright, 1972b: 1380.

• *Tricolus endemos* Bright, 2019: 424.

• *Tricolus fenoris* Wood, 1974a: 60.

• *Tricolus frontalis* Wood, 1974a: 61.

• *Tricolus gracilis* Eggers, 1937b: 87.

• *Tricolus inaffectus* Wood, 1974a: 57.

• *Tricolus incomptus* Bright, 2019: 426.

• *Tricolus inornatus* Wood, 1974a: 57.

• *Tricolus intrusus* Wood, 1974a: 58.

• *Tricolus minutissimus* Schedl, 1976: 84.

• *Tricolus myrti* Wood, 2007: 687.

• *Tricolus mystacinus* Wood, 2007: 682.

• *Tricolus naevus* Wood, 1974a: 61.

• *Tricolus nayaritensis* Wood, 2007: 688.

• *Tricolus nodifer* Blandford, 1905: 287.

= *Tricolus triarmatus* Schedl, 1939d: 578.

• *Tricolus ovicollis* Blandford, 1905: 287.

• *Tricolus parsus* Wood, 1974a: 59.

• *Tricolus partilis* Wood, 1974a: 60.

• *Tricolus parvus* Wood, 2007: 685.

• *Tricolus peltatus* Wood, 1974a: 62.

• *Tricolus perdiligens* Schedl, 1950c: 171.

= *Tricolus ignotus* Bright, 1972a: 99.

• *Tricolus pernanulus* Schedl, 1939d: 581.

• *Tricolus plaumanni* Schedl, 1954b: 40.

= *Pterocyclonoides octodentatus* Schedl, 1970e: 102.

• *Tricolus pumilio* Eggers, 1937b: 87.

• *Tricolus ruficollis* (Fabricius, 1801: 388) (*Bostrichus*).

• *Tricolus rufithorax* Wood, 1974a: 59.

• *Tricolus rufodorsalis* Wood, 2007: 685.

• *Tricolus saundersi* Wood, 1967c: 139.

• *Tricolus scitulus* Wood, 1974a: 62.

• *Tricolus senex* Schedl, 1939d: 580.

• *Tricolus simplicis* Wood, 1974a: 57.

• *Tricolus spheniscus* Schedl, 1939d: 581.

• *Tricolus subincisuralis* Schedl, 1939h: 726.

• *Tricolus subopacus* Wood, 2007: 681.

• *Tricolus subrufus* Wood, 2007: 681.

• *Tricolus undulatus* Wood, 2007: 689.

• *Tricolus unidentatus* Bright, 1972a: 98.

### PITYOPHTHORINA Eichhoff

23 genera, 751 spp. (5 spp. with 11 sspp.).

#### *Acorthylus* Brèthes

6 spp.

• *Acorthylus asperatus* Brèthes, 1922: 305.

• *Acorthylus bosqi* (Schedl, 1938f: 24) (*Phacrylus*).

= *Ernoporus squamulosus* Eggers, 1943d: 356.

• *Acorthylus frontalis* Wood, 2007: 485.

• *Acorthylus gracilis* (Schedl, 1972f: 58) (*Phacrylus*).

• *Acorthylus pruni* (Wood, 1971a: 33) (*Phacrylus*).

• *Acorthylus robustus* (Schedl, 1952a: 453) (*Phacrylus*).

#### *Araptus* Eichhoff

187 spp.

• *Araptus accinctus* Wood, 1974a: 44.

• *Araptus adustus* Bright, 2019: 317.

• *Araptus amazonicus* (Eggers, 1936d: 391) (*Neodryocoetes*).

= *Neodryocoetes sparsepunctatus* Schedl, 1938f: 26.

• *Araptus andinus* Wood, 2007: 609.

• *Araptus araguensis* Wood, 2007: 601.

• *Araptus araucariae* (Schedl, 1966d: 109) (*Conophthocranulus*).

• *Araptus araujiae* (Brèthes, 1921: 165) (*Xyleborus*).

= *Neodryocoetes longicollis* Schedl, 1938b: 179.

• *Araptus argentiniae* (Schedl, 1958j: 44) (*Breviophthorus*).

• *Araptus attenuatus* Wood, 1975a: 30.

• *Araptus barinensis* Wood, 2007: 594.

• *Araptus beaveri* Wood, 2007: 590.

• *Araptus beckeri* Bright, 2019: 318.

• *Araptus bituberculatus* Bright, 2019: 319.

• *Araptus blanditus* Wood, 1974a: 47.

• *Araptus bolivianus* (Schedl, 1951c: 110) (*Neodryocoetes*).

• *Araptus brasiliensis* (Schedl, 1938b: 178) (*Neodryocoetes*).

• *Araptus brevisetosus* (Eggers, 1933f: 3) (*Pityophthorus*).

• *Araptus caperatus* Bright, 2019: 319.

• *Araptus caribaeus* (Blackman, 1942b: 185) (*Neodryocoetes*).

• *Araptus carinifrons* (Blandford, 1904: 244) (*Pityophthorus*).

• *Araptus celatus* (Schedl, 1967d: 9) (*Breviophthorus*).

• *Araptus chilensis* (Schedl, 1955h: 258) (*Conophthocranulus*).

• *Araptus ciseruditus* Bright, 2019: 320.

• *Araptus clematicolens* Wood, 2007: 608.

• *Araptus clusiae* Wood, 2007: 582.

• *Araptus columbianus* (Schedl, 1938b: 178) (*Neodryocoetes*).

• *Araptus concentralis* (Schedl, 1951c: 107) (*Neodryocoetes*).

• *Araptus conditus* Wood, 1974a: 48.

• *Araptus confinis* (Blandford, 1904: 241) (*Pityophthorus*).

= *Neopityophthorus glabricollis* Schedl, 1938b: 181.

• *Araptus confluens* (Schedl, 1964g: 311) (*Neodryocoetes*) (RN).

= *Ctenyophthorus concentralis* Schedl, 1963f: 222.

• *Araptus consobrinus* Wood, 1975b: 394.

• *Araptus convexifrons* Wood, 2007: 580.

• *Araptus corpulentus* (Schedl, 1973a: 371) (*Neodryocoetes*).

• *Araptus costaricensis* Schedl, 1938b: 180 (*Neodryocoetes insularis costaricensis*).

= *Neodryocoetes nitidulus* Schedl, 1967d: 11.

• *Araptus coumacomis* Wood, 2007: 589.

• *Araptus cracens* Wood, 2007: 604.

• *Araptus crassulus* Wood, 1981: 122 (RN).

= *Araptus crassus* Wood, 1977a: 211.

• *Araptus crassus* (Schedl, 1966d: 108) (*Thamnophthorus*).

• *Araptus cribricollis* (Schedl, 1954b: 36) (*Neodryocoetes*).

= *Breviophthorus sulcatus* Schedl, 1959b: 552.

• *Araptus cribripennis* (Schedl, 1976: 70) (*Neodryocoetes*).

• *Araptus cubensis* (Blackman, 1942b: 191) (*Neodryocoetes*).

• *Araptus culmenifrons* Bright, 2019: 322.

• *Araptus declivis* Wood, 2007: 596.

• *Araptus decorulus* Wood, 1974d: 278 (RN).

= *Araptus decorus* Wood, 1974a: 47.

• *Araptus decorus* (Bright, 1972a: 96) (*Neodryocoetes*).

• *Araptus delicatus* Wood, 1974a: 44.

• *Araptus dentifrons* Wood, 1974a: 45.

= *Breviophthorus mexicanus* Schedl, 1963c: 161.

• *Araptus deyrollei* (Blandford, 1904: 245) (*Pityophthorus*).

= *Araptus insinuatus* Wood, 1974a: 43.

• *Araptus dubius* (Schedl, 1966d: 105) (*Neodryocoetes*).

• *Araptus eggersi* (Schedl, 1951c: 115) (*Brachydendrulus*).

• *Araptus elongatus* (Schedl, 1961h: 226) (*Thamnophthorus*).

• *Araptus epistomalis* Wood, 2007: 585.

• *Araptus equihuai* Wood, 2007: 595.

• *Araptus eruditus* (Schedl, 1938b: 182) (*Neopityophthorus*).

= *Neodryocoetes buscki* Blackman, 1942b: 192.

• *Araptus eusimplicis* Wood, 2007: 608.

• *Araptus excavatus* Wood, 2007: 571.

• *Araptus exigialis* Wood, 1974a: 50.

• *Araptus expers* Wood, 2007: 612.

• *Araptus facetus* Wood, 1974a: 46.

• *Araptus falaciosus* Wood, 2007: 586.

• *Araptus ferrugineus* Bright, 2019: 324.

• *Araptus fossifrons* Wood, 1975a: 30.

• *Araptus foveifrons* (Schedl, 1963c: 161) (*Thamnophthorus*).

= *Araptus interjectus* Wood, 1974a: 44.

• *Araptus franzi* Bright & Peck, 1998: 247.

• *Araptus frenatus* (Schedl, 1939f: 411) (*Thamnophthorus*).

• *Araptus frontalis* (Schedl, 1967d: 8) (*Breviophthorus*).

• *Araptus frontis* Wood, 1989: 177 (RN).

= *Gnathocranus frontalis* Schedl, 1978a: 302.

• *Araptus frugalis* Wood, 1974a: 49.

• *Araptus furvescens* Wood, 1974a: 53.

• *Araptus furvus* Wood, 1974a: 53.

• *Araptus fuscus* Bright, 2019: 324.

• *Araptus genialis* Wood, 1974a: 44.

• *Araptus gloriosus* Wood, 2007: 590.

• *Araptus gracilens* Wood, 1976: 364.

• *Araptus gracilentus* (Schedl, 1972f: 61) (*Neodryocoetes*).

= *Breviophthorus brasiliensis* Schedl, 1938b: 177.

• *Araptus gracilis* (Schedl, 1966d: 106) (*Neodryocoetes*).

• *Araptus grandis* (Schedl, 1954b: 35) (*Neodryocoetes*).

• *Araptus granulipennis* (Schedl, 1967d: 12) (*Neodryocoetes*).

• *Araptus granulosus* (Schedl, 1972f: 60) (*Breviophthorus*).

• *Araptus grenadaensis* Bright, 2019: 325.

• *Araptus guadeloupanus* Wood, 1989: 177 (RN).

= *Brachydendrulus guadeloupensis* Schedl, 1970e: 91.

• *Araptus guadeloupensis* (Schedl, 1951c: 73) (*Neodryocoetes*) (RN).

= *Neopityophthorus insularis* Eggers, 1940a: 130.

• *Araptus guyanae* Wood, 2007: 602.

• *Araptus guyanensis* Wood, 2007: 578.

• *Araptus hostilis* (Blackman, 1942b: 189) (*Neodryocoetes*).

• *Araptus howdeni* Bright, 2019: 328.

• *Araptus hymenaeae* (Eggers, 1933f: 9) (*Neodryocoetes*).

= *Neodryocoetes insularis* Eggers, 1940a: 128.

= *Neodryocoetes guianae* Blackman, 1942b: 186.

= *Neodryocoetes hoodi* Blackman, 1942b: 187.

= *Neodryocoetes humilis* Blackman, 1942b: 188.

• *Araptus imitatrix* (Schedl, 1972f: 62) (*Neodryocoetes*).

• *Araptus impensus* (Wood, 1961a: 6) (*Thamnophthorus*).

• *Araptus incolus* Bright, 2019: 330.

• *Araptus incommodus* (Blandford, 1904: 245) (*Pityophthorus*).

• *Araptus incompositus* (Blandford, 1904: 243) (*Pityophthorus*).

• *Araptus ineditus* Bright, 2019: 331.

• *Araptus insulanus* Bright, 2019: 332.

• *Araptus jaliscoensis* Wood, 2007: 580.

• *Araptus kirkendalli* Wood, 2007: 578.

• *Araptus laevigatus* (Eggers, 1933f: 6) (*Pityophthorus*).

• *Araptus laudatus* Wood, 1974a: 49.

• *Araptus lepidus* Wood, 1974a: 49.

• *Araptus leptus* (Bright, 1972d: 1666) (*Neodryocoetes*).

• *Araptus liminaris* Wood, 2007: 577.

• *Araptus linearis* (Schedl, 1938b: 174) (*Thamnophthorus*).

• *Araptus macer* (Bright, 1972d: 1666) (*Neodryocoetes*).

= *Neodryocoetes tuberculatus* Bright, 1972d: 1665.

• *Araptus medialis* Wood, 1974a: 48.

• *Araptus melanurus* Bright, 2019: 333.

• *Araptus mendicus* Wood, 1974a: 54.

• *Araptus micaceus* Wood, 1975a: 29.

• *Araptus micarius* Wood, 2007: 603.

• *Araptus micrographus* (Schedl, 1972f: 60) (*Breviophthorus*).

= *Pityophthorus micrograptinus* Wood, 1989: 179 (RN).

• *Araptus micropilosus* Wood, 1982: 224.

• *Araptus minulus* Wood, 2007: 602.

• *Araptus minutissimus* (Schedl, 1954b: 35) (*Brachydendrulus*).

• *Araptus mirabilis* Wood, 2007: 591.

• *Araptus mirus* Wood, 2007: 591.

• *Araptus montanus* (Bright, 1972a: 93) (*Neodryocoetes*).

• *Araptus morigerus* Wood, 1982: 224.

• *Araptus mucunae* (Blackman, 1942b: 181) (*Neodryocoetes*).

• *Araptus mucunavorus* Wood, 2007: 572.

• *Araptus muticus* Wood, 2007: 611.

• *Araptus nanulus* Wood, 1974a: 54.

• *Araptus niger* (Bright, 1972a: 93) (*Neodryocoetes*).

• *Araptus nigrellus* Wood, 1974a: 52.

• *Araptus nigriculus* Bright, 2019: 335.

• *Araptus nitens* Wood, 2007: 607 (RN).

= *Breviophthorus nitidipennis* Schedl, 1967d: 10.

• *Araptus nitidipennis* (Schedl, 1963e: 57) (*Neodryocoetes*).

• *Araptus novateutonicus* (Schedl, 1951c: 116) (*Gnathocranus*).

• *Araptus nudus* (Schedl, 1938b: 176) (*Thamnophthorus*).

= *Thamnophthorus dubiosus* Schedl, 1964j: 206.

• *Araptus obesus* Wood, 1977a: 212.

• *Araptus obscurus* (Eggers, 1936d: 390) (*Neodryocoetes*).

• *Araptus obsoletus* (Blandford, 1904: 242) (*Pityophthorus*).

• *Araptus ocularis* Wood, 2007: 601.

• *Araptus pallidus* (Blackman, 1942b: 193) (*Neodryocoetes*).

= *Neodryocoetes portoricensis* Schedl, 1951c: 109.

= *Neodryocoetes devius* Schedl, 1972f: 61.

• *Araptus paranae* (Schedl, 1954b: 34) (*Brachydendrulus*).

• *Araptus partilis* Wood, 2007: 612.

• *Araptus parvistriatus* Wood, 2007: 606.

• *Araptus parvulus* Wood, 2007: 611.

• *Araptus pernicialis* Wood, 2007: 573.

• *Araptus placetulus* Wood, 1982: 225.

• *Araptus plaumanni* (Schedl, 1970e: 102) (*Breviophthorus*).

• *Araptus plaumannianus* Wood, 2007: 575 (RN).

= *Brachydendrulus plaumanni* Schedl, 1976: 70.

• *Araptus playonensis* Wood, 2007: 584.

• *Araptus plicatus* Wood, 2007: 573.

• *Araptus politus* (Blandford, 1904: 244) (*Pityophthorus*).

= *Neodryocoetes mexicanus* Eggers, 1936d: 391.

= *Neodryocoetes hubbardi* Blackman, 1942b: 182.

• *Araptus poricollis* (Blandford, 1904: 238) (*Pityophthorus*).

• *Araptus praevius* Wood, 2007: 589 (RN).

= *Araptus frontalis* Wood, 1974a: 52.

• *Araptus pseudosimilis* Wood, 2007: 599.

• *Araptus pubescens* (Schedl, 1951a: 291) (*Neodryocoetes*).

• *Araptus pumilus* Wood, 2007: 594.

• *Araptus punctatissimus* (Schedl, 1938b: 179) (*Neodryocoetes*).

• *Araptus refertus* Wood, 1974a: 51.

• *Araptus reticulatus* Wood, 2007: 605.

• *Araptus robustus* (Schedl, 1964j: 207) (*Thamnophthorus*).

• *Araptus roupalae* Wood, 2007: 610.

• *Araptus rufopalliatus* Eichhoff, 1872a: 136.

• *Araptus schedli* (Blackman, 1942b: 195) (*Neodryocoetes*).

= *Neodryocoetes lenis* Blackman, 1942b: 198.

• *Araptus schedlianus* Wood, 2007: 614.

• *Araptus schwarzi* (Blackman, 1942b: 178) (*Thamnophthorus*).

• *Araptus semisulcatus* Wood, 2007: 608 (RN).

= *Neodryocoetes sulcatus* Nunberg, 1964: 435.

• *Araptus simplicis* Wood, 2007: 607.

• *Araptus sobrinus* Wood, 1974d: 287.

• *Araptus speciosus* Wood, 1980b: 357.

• *Araptus spiculatus* Wood, 2007: 588.

• *Araptus splendidulus* (Schedl, 1966d: 107) (*Neodryocoetes*).

• *Araptus squamosus* Bright, 2019: 337.

• *Araptus subaciculatus* Wood, 2007: 574.

• *Araptus subconcentralis* Wood, 2007: 597.

• *Araptus subemarginatus* Wood, 2007: 613.

• *Araptus subsimilis* (Schedl, 1966d: 104) (*Breviophthorus*).

= *Pityophthorus subsimilans* Wood, 1989: 179 (RN).

• *Araptus subsulcatus* (Schedl, 1963f: 223) (*Breviophthorus*).

• *Araptus tabogae* (Blackman, 1942b: 184) (*Neodryocoetes*).

= *Neodryocoetes vinealis* Bright, 1972d: 1667.

• *Araptus tenuis* (Blackman, 1942b: 197) (*Neodryocoetes*).

= *Araptus placatus* Wood, 1974a: 46.

• *Araptus teres* (Blackman, 1942b: 190) (*Neodryocoetes*).

• *Araptus trepidus* Wood, 1974a: 51.

• *Araptus turnbowi* Bright, 2019: 338.

• *Araptus umbraticus* (Schedl, 1966d: 108) (*Neodryocoetes*).

• *Araptus uruguayensis* Wood, 2007: 574.

• *Araptus ustulatus* Bright, 2019: 339.

• *Araptus varius* Wood, 2007: 581.

• *Araptus veritus* Wood, 2007: 597.

• *Araptus vesculus* Wood, 1974a: 50.

• *Araptus vinnulus* Wood, 1974a: 53.

• *Araptus virolae* Wood, 2007: 593.

• *Araptus virolavorus* Wood, 2007: 601.

• *Araptus virtus* (Schedl, 1938b: 186) (*Pityophthorus*).

• *Araptus volastos* (Schedl, 1938b: 175) (*Thamnophthorus*).

• *Araptus wintoni* Bright, 2019: 340.

• *Araptus xylotrupes* (Eichhoff, 1872a: 135) (*Pityophthorus*).

#### *Conophthorus* Hopkins

14 spp.

• *Conophthorus apachecae* Hopkins, 1915a: 432.

• *Conophthorus conicolens* Wood, 1977a: 212.

• *Conophthorus coniperda* (Schwarz, 1895: 144) (*Pityophthorus*).

= *Conophthorus clunicus* Hopkins, 1915a: 432.

= *Conophthorus taedae* Hopkins, 1915a: 431.

• *Conophthorus echinatae* Wood, 1978: 398.

• *Conophthorus edulis* Hopkins, 1915a: 430.

= *Conophthorus cembroides* Wood, 1971b: 74.

• *Conophthorus insulatus* Bright, 2019: 340.

• *Conophthorus mexicanus* Wood, 1962: 79.

• *Conophthorus michoacanae* Wood, 1980b: 354.

• *Conophthorus monophyllae* Hopkins, 1915a: 433.

• *Conophthorus ponderosae* Hopkins, 1915a: 431.

= *Conophthorus contortae* Hopkins, 1915a: 432.

= *Conophthorus flexilis* Hopkins, 1915a: 433.

= *Conophthorus lambertianae* Hopkins, 1915a: 433.

= *Conophthorus monticolae* Hopkins, 1915a: 432.

= *Conophthorus scopulorum* Hopkins, 1915a: 431.

• *Conophthorus radiatae* Hopkins, 1915a: 432.

• *Conophthorus resinosae* Hopkins, 1915a: 431.

= *Conophthorus virginianae* Hopkins, 1915a: 431.

= *Conophthorus banksianae* McPherson in: McPherson et al. 1970: 1020.

• *Conophthorus teocotum* Wood, 1980b: 354.

• *Conophthorus terminalis* Flores & Bright, 1987: 181.

#### *Cryptocarenus* Eggers

17 spp.

• *Cryptocarenus amazonicus* Wood, 2007: 492.

• *Cryptocarenus barinensis* Wood, 2007: 496.

• *Cryptocarenus beaveri* Wood, 2007: 495.

• *Cryptocarenus brevicollis* Eggers, 1937b: 81.

= *Cryptocarenus coronatus* Wood, 1971a: 36.

• *Cryptocarenus diadematus* Eggers, 1937b: 80.

• *Cryptocarenus frontalis* Wood, 2007: 495.

• *Cryptocarenus harringtoni* (Blackman, 1943a: 38) (*Tachyderes*).

• *Cryptocarenus heveae* (Hagedorn, 1912c: 338) (*Stephanoderes*).

= *Cryptocarenus caraibicus* Eggers, 1937b: 82.

= *Tachyderes parvus* Blackman, 1943a: 36.

= *Miocryphalus brasiliensis* Schedl, 1951c: 96.

= *Cryptocarenus porosus* Wood, 1954b: 1014.

= *Cryptocarenus acaciae* Schedl, 1958j: 45.

• *Cryptocarenus laevigatus* (Blandford, 1904: 230) (*Hypothenemus*).

• *Cryptocarenus lepidus* Wood, 1971a: 36.

• *Cryptocarenus pilosus* Eggers, 1937b: 81.

• *Cryptocarenus pubescens* Wood, 1986a: 271.

• *Cryptocarenus punctifrons* Schedl, 1939f: 410.

= *Miocryphalus brasiliensis* Schedl, 1951c: 96.

• *Cryptocarenus pygmaeus* Schedl, 1966f: 370.

• *Cryptocarenus seriatus* (Eggers, 1933f: 10) (Cryptocarenus*).

= *Cryptocarenus adustus* Eggers, 1933f: 11.

= *Cryptocarenus bolivianus* Eggers, 1943d: 356.

= *Tachyderes floridensis* Blackman, 1943a: 36.

• *Cryptocarenus spatulatus* Wood, 1986a: 272.

• *Cryptocarenus tropicalis* Wood, 2007: 491.

#### *Dacnophthorus* Wood

5 spp.

• *Dacnophthorus artus* (Wood, 1974a: 27) (*Gnathophthorus*).

• *Dacnophthorus clematis* (Wood, 1971a: 51) (*Gnathophthorus*).

• *Dacnophthorus cracens* (Wood, 1971a: 53) (*Gnathophthorus*).

• *Dacnophthorus pertusus* (Wood, 1971a: 53) (*Gnathophthorus*).

• *Dacnophthorus rallus* (Wood, 1971a: 53) (*Gnathophthorus*).

#### *Dendroterus* Blandford

15 spp.

• *Dendroterus cognatus* Wood, 1971a: 46.

• *Dendroterus decipiens* Wood, 1959a: 5.

• *Dendroterus defectus* Wood, 1971a: 45.

• *Dendroterus eximius* Wood, 1971a: 44.

• *Dendroterus fossifrons* Wood, 1984a: 114.

• *Dendroterus luteolus* (Schedl, 1951c: 111) (*Plesiophthorus*).

= *Dendroterus mundus* Wood, 1959a: 3.

• *Dendroterus mexicanus* Blandford, 1904: 233.

= *Conophthocranulus umbratus* Schedl, 1937b: 168.

= *Dendroterus confinis* Wood, 1959a: 6.

• *Dendroterus modicus* Wood, 1984a: 115.

• *Dendroterus parilis* Wood, 1971a: 44.

• *Dendroterus perspectus* (Schedl, 1940a: 343) (*Plesiophthorus*).

• *Dendroterus resolutus* Wood, 1971a: 45.

• *Dendroterus sallei* Blandford, 1904: 233.

= *Xylochilus insularis* Schedl, 1956a: 31.

• *Dendroterus sodalis* Wood, 1971a: 44.

• *Dendroterus striatus* (LeConte, 1868: 156) (*Cryphalus*).

= *Plesiophthorus californicus* Schedl, 1952j: 123.

• *Dendroterus texanus* Wood, 1959a: 4.

#### *Gnatholeptus* Blackman

7 spp.

• *Gnatholeptus concinnus* Bright, 2019: 343.

• *Gnatholeptus hispanicus* Bright, 2019: 344.

• *Gnatholeptus insularis* Bright, 2019: 345.

• *Gnatholeptus panamensis* Blackman, 1943a: 35.

= *Pityophthorus epistomalis* Schedl, 1961h: 224.

• *Gnatholeptus semiermis* (Nunberg, 1963: 98) (*Pityophthorus*).

• *Gnatholeptus shannoni* (Blackman, 1942b: 224) (*Pityophthorus*).

= *Gnatholeptus mandibularis* Blackman, 1943a: 34.

= *Pityophthorus gentilis* Schedl, 1961h: 225.

• *Gnatholeptus tenellus* (Schedl, 1951c: 109) (*Neodryocoetes*).

= *Ctenyophthorus mexicanus* Schedl, 1963c: 162.

= *Neodryocoetes granulatus* Schedl, 1964g: 311 (RN).

= *Araptus cuspidis* Wood, 1974a: 46.

#### *Gnathoraptus* Bright

1 sp.

• *Gnathoraptus mandibularis* Bright, 2019: 346.

#### *Mimiocurus* Schedl

14 spp.

• *Mimiocurus acuminatus* Schedl, 1957b: 72.

• *Mimiocurus beesoni* Wood, 1989: 182.

• *Mimiocurus camerunus* (Hagedorn, 1909: 743) (*Araptus*).

• *Mimiocurus congonus* (Schedl, 1952b: 14) (*Brachydendrulus*).

• *Mimiocurus kikusae* (Schedl, 1957b: 77) (*Mimiophthorus*).

= *Mimiophthorus kikusae occidentalis* Schedl, 1961d: 83.

• *Mimiocurus montanus* (Schedl, 1957b: 71) (*Micracidendron montanum*).

• *Mimiocurus monticulus* Wood, 1989: 178 (RN).

= *Mimiocurus montanus* Schedl, 1957b: 73.

• *Mimiocurus oleanderi* (Schedl, 1961c: 187) (*Neodryocoetes*).

• *Mimiocurus orientalis* (Schedl, 1972j: 51) (*Mimiophthorus*).

• *Mimiocurus rosseli* (Schedl, 1973d: 88) (*Taphroterus*).

• *Mimiocurus rugicollis* (Schedl, 1957b: 58) (*Mimiophthorus*).

• *Mimiocurus ruwenzoriensis* (Schedl, 1963a: 30) (*Mimiophthorus*).

• *Mimiocurus umbratus* (Schedl, 1972a: 290) (*Mimiophthorus*).

• *Mimiocurus uniseriatus* (Schedl, 1979b: 451) (*Mimiophthorus*).

#### *Neocryphus* Nunberg

1 sp.

• *Neocryphus argentinensis* Nunberg, 1956a: 141.

= *Phacrylus cristatus* Schedl, 1979a: 61.

#### *Phelloterus* Wood

3 spp.

• *Phelloterus anaxeus* Wood, 1971a: 47.

• *Phelloterus atrocis* Wood, 1971a: 48.

• *Phelloterus tersus* Wood, 1971a: 47.

#### *Phloeoterus* Wood

1 sp.

• *Phloeoterus burserae* Wood, 1984a: 117.

#### *Pityoborus* Blackman

7 spp.

• *Pityoborus comatus* (Zimmermann, 1868: 143) (*Crypturgus*).

= *Pityophthorus seriatus* LeConte, 1878a: 433.

• *Pityoborus frontalis* Wood, 1971a: 49.

= *Pityoborus severus* Bright, 1972d: 1676.

• *Pityoborus hirtellus* Wood, 1958: 50.

• *Pityoborus hondurensis* Wood, 1971a: 49.

• *Pityoborus rubentis* Wood, 1958: 51.

• *Pityoborus secundus* Blackman, 1928a: 146.

= *Pityoborus tertius* Blackman, 1942b: 202.

= *Pityoborus intonsus* Wood, 1958: 54.

= *Pityoborus immitus* Bright, 1972d: 1674.

= *Pityoborus ramosus* Bright, 1972d: 1677.

• *Pityoborus velutinus* Wood, 1958: 48.

#### *Pityodendron* Schedl

1 sp.

• *Pityodendron madagascariense* Schedl, 1953c: 93 (E).

#### *Pityophthorus* Eichhoff

421 spp. (5 spp. with 11 sspp.).

• *Pityophthorus abiegnus* Wood, 1964: 67.

• *Pityophthorus abietinus* Wood, 1989: 178 (RN).

= *Pityophthorus abietis* Kurentsov, 1941: 179.

= *Pityophthorus sibiricus* Nunberg, 1956c: 208 (RN).

= *Pityophthorus kurentzovi* Krivolutskaya, 1996: 370 (RN).

• *Pityophthorus ablusus* Bright, 1985c: 476.

• *Pityophthorus abnormalis* Bright, 1972a: 88.

• *Pityophthorus absonus* Blackman, 1928a: 35.

= *Pityophthorus demissus* Blackman, 1928a: 74.

= *Pityophthorus inyoensis* Bright, 1971: 65.

• *Pityophthorus abstrusus* Bright, 1976: 427.

• *Pityophthorus aciculatus* Bright, 1977: 519.

• *Pityophthorus acolus* Bright, 2019: 356.

• *Pityophthorus acuminatus* (Schedl, 1940a: 346) (*Neopityophthorus*).

• *Pityophthorus acutus* Blackman, 1928a: 134.

• *Pityophthorus africanulus* Wood, 1992: 79 (RN).

= *Pityophthorus africanus* Schedl, 1965g: 1079.

• *Pityophthorus africanus* Eggers, 1927c: 184.

• *Pityophthorus alienus* Eichhoff, 1872a: 135.

• *Pityophthorus alni* Blackman, 1942b: 209.

• *Pityophthorus alnicolens* Wood, 1977a: 213.

• *Pityophthorus alpinensis* Hopping, 1960: 865.

• *Pityophthorus alvarengai* Schedl, 1972f: 63.

• *Pityophthorus amiculus* Wood, 1975b: 398.

• *Pityophthorus amoenus* Blandford, 1904: 237.

• *Pityophthorus amplus* (Blackman, 1928a: 18) (*Myeloborus*).

• *Pityophthorus anacardii* Wood, 2007: 656.

• *Pityophthorus angustus* Blackman, 1928a: 83.

• *Pityophthorus annectens* LeConte, 1878b: 622.

= *Pityophthorus citus* Blackman, 1928a: 137.

• *Pityophthorus anthracinus* Bright, 1976: 428.

• *Pityophthorus anticus* Schedl, 1976: 66.

• *Pityophthorus antillicus* Bright, 1981b: 162.

• *Pityophthorus apachae* Bright, 1977: 520.

• *Pityophthorus apicipennis* Schedl, 1976: 67.

• *Pityophthorus apiculatus* Schedl, 1937b: 167.

• *Pityophthorus aquilus* Blackman, 1928a: 33.

= *Pityophthorus caelator* Blackman, 1928a: 78.

= *Pityophthorus aristatae* Bright, 1964: 166.

• *Pityophthorus arakii* (Sawamoto, 1942: 167) (*Cladoborus*).

• *Pityophthorus arcanus* Bright, 1976: 429.

• *Pityophthorus arceuthobii* Wood, 1971a: 48.

• *Pityophthorus argentinensis* Eggers, 1951: 150.

• *Pityophthorus ascendens* Schedl, 1972f: 64.

• *Pityophthorus ashanti* Schedl, 1972a: 291.

• *Pityophthorus assitus* Wood, 1977a: 214.

• *Pityophthorus astringens* Bright, 2019: 358.

• *Pityophthorus aterrimus* Eggers, 1931b: 31.

• *Pityophthorus atkinsoni* Bright, 1985b: 467.

• *Pityophthorus atomus* Wood, 1964: 61.

• *Pityophthorus attenuatus* Blackman, 1942b: 222.

= *Pityophthorus pusillus* Wood, 1964: 62.

• *Pityophthorus auspicatus* Bright, 2019: 358.

• *Pityophthorus aztecus* Bright, 1977: 520.

• *Pityophthorus bahiae* Wood, 2007: 654.

• *Pityophthorus balcanicus* Pfeffer, 1940a: 123.

• *Pityophthorus balsameus* Blackman, 1922b: 119.

= *Pityophthorus patchi* Blackman, 1922b: 120.

• *Pityophthorus barberi* Blackman, 1928a: 112.

• *Pityophthorus barbifer* Schedl, 1964h: 46.

• *Pityophthorus barbosai* Wood, 2007: 652.

• *Pityophthorus bassetti* Blackman, 1920b: 1.

• *Pityophthorus bigranulatus* Bright, 2019: 359.

• *Pityophthorus biovalis* Blackman, 1922b: 122.

• *Pityophthorus blackmani* (Schedl, 1935f: 344) (*Conophthocranulus*).

• *Pityophthorus blandulus* Schedl, 1955i: 19.

• *Pityophthorus blandus* Blackman, 1928a: 107.

= *Pityophthorus singularis* Bright, 1966: 300.

• *Pityophthorus bolivianus* Eggers, 1943d: 359.

• *Pityophthorus borrichiae* Wood, 1964: 60.

• *Pityophthorus boycei* Swaine, 1925b: 192.

= *Myeloborus catulus* Blackman, 1928a: 21.

= *Myeloborus iniquus* Blackman, 1928a: 27.

= *Pityophthorus siouxensis* Bright, 1976: 439.

• *Pityophthorus bravoi* Bright, 1986b: 679.

• *Pityophthorus brevicomatus* Bright, 1976: 430.

• *Pityophthorus brevis* Blackman, 1928a: 81.

• *Pityophthorus brighti* Wood, 1989: 179 (RN).

= *Pityophthorus blackmani* Bright, 1977: 521.

• *Pityophthorus briscoei* Blackman, 1922b: 123.

= *Pityophthorus mundus* Blackman, 1928a: 86.

• *Pityophthorus burserae* Wood, 1976: 362.

• *Pityophthorus busseae* Schedl, 1962g: 69.

• *Pityophthorus buyssoni angeri* Pfeffer, 1927: 111 (*Pityophthorus angeri*).

• *Pityophthorus buyssoni buyssoni* Reitter, 1901a: 101 (*Pityophthorus buyssoni*).

• *Pityophthorus cacuminatus* Blandford, 1904: 237.

• *Pityophthorus californicus* Bright, 1976: 427 (RN).

= *Pityophthorus deleoni* Bright, 1966: 302.

• *Pityophthorus camerunus* Eggers, 1920b: 35.

• *Pityophthorus capillosus* Bright, 2019: 360.

• *Pityophthorus carinatus carinatus* Bright, 1978: 73.

• *Pityophthorus carinatus monticolae* Bright, 1978: 74.

• *Pityophthorus cariniceps* LeConte, 1876: 353.

= *Pityophthorus canadensis* Swaine, 1917: 24.

= *Pityophthorus cognatus* Blackman, 1928a: 69.

• *Pityophthorus carinulatus* Swaine, 1925b: 193.

= *Pityophthorus opimus* Blackman, 1928a: 80.

• *Pityophthorus carmeli* Swaine, 1918: 100.

= *Pityophthorus torreyanae* Swaine, 1918: 101.

• *Pityophthorus carniolicus* Wichmann, 1910: 145.

• *Pityophthorus cascoensis* Blackman, 1928a: 99.

= *Pityophthorus pilifer* Schedl, 1931c: 166.

• *Pityophthorus cavatus* Bright, 1978: 74.

• *Pityophthorus cedri* Wood, 1989: 182.

• *Pityophthorus cephalonicae* Pfeffer, 1940a: 119.

= *Pityophthorus polonicus* Karpiński, 1949: 125.

• *Pityophthorus chalcoensis* Hopkins, 1906: 73 (E).

= *Pityophthorus herrerai* Hopkins, 1906: 74.

• *Pityophthorus chilgoza* Wood, 1989: 183.

• *Pityophthorus ciliatus* Blackman, 1942b: 211.

• *Pityophthorus clarus* Blackman, 1928a: 130.

• *Pityophthorus clivus* Bright, 1977: 522.

• *Pityophthorus collaris* Schedl, 1965f: 67.

• *Pityophthorus comosus* Blackman, 1928a: 65.

= *Pityophthorus foratus* Wood, 1967a: 40.

• *Pityophthorus concavus* Blackman, 1928a: 85.

= *Pityophthorus hesperius* Bright, 1978: 76.

• *Pityophthorus concentralis* Eichhoff, 1878b: 188.

= *Pityophthorus lateralis* Swaine, 1917: 27.

• *Pityophthorus concinnus* Wood, 1977a: 214.

• *Pityophthorus confertus* Swaine, 1917: 27.

= *Pityophthorus agnatus* Blackman, 1928a: 125.

= *Pityophthorus burkei* Blackman, 1928a: 129.

= *Pityophthorus comptus* Blackman, 1928a: 127.

• *Pityophthorus confinis* LeConte, 1876: 354.

• *Pityophthorus confractus* Bright, 1985a: 179.

• *Pityophthorus confusus bellus* Blackman, 1928a: 123 (*Pityophthorus bellus*).

• *Pityophthorus confusus confusus* Blandford, 1904: 237 (*Pityophthorus confusus*).

• *Pityophthorus confusus sequestus* Bright, 2019: 364.

• *Pityophthorus congonus* Eggers, 1927c: 185.

• *Pityophthorus congruus* Bright, 2019: 364.

• *Pityophthorus conscriptus* Bright, 1986b: 680.

• *Pityophthorus consimilis* LeConte, 1878b: 622.

= *Pityophthorus granulatus* Swaine, 1917: 28.

= *Pityophthorus nudus* Swaine, 1917: 30.

• *Pityophthorus conspectus* Wood, 1976: 360.

• *Pityophthorus convexicollis* Bright & Torres, 2006: 422.

• *Pityophthorus convexus* Bright, 2019: 366.

• *Pityophthorus coronarius* Blackman, 1942b: 220.

• *Pityophthorus corruptus* Wood, 1976: 363.

• *Pityophthorus cortezi* Bright, 1977: 523.

• *Pityophthorus corticalis* Eichhoff, 1872a: 135.

• *Pityophthorus costabilis* Wood, 1976: 352.

• *Pityophthorus costalimai* Blackman, 1942b: 223.

• *Pityophthorus costatulus* Wood, 1976: 351.

• *Pityophthorus costatus* Wood, 1975b: 395.

• *Pityophthorus costifera* Bright, 1985c: 477.

• *Pityophthorus cracentis* Bright, 1985c: 477.

• *Pityophthorus crassus* Blackman, 1928a: 67.

• *Pityophthorus crinalis* Blackman, 1928a: 41.

• *Pityophthorus cristatus* Wood, 1964: 68.

• *Pityophthorus crotonis* Wood, 1977c: 517.

• *Pityophthorus culminicolae* Bright, 1977: 524.

• *Pityophthorus cuspidatus* Blackman, 1942b: 217.

• *Pityophthorus debilis* Wood, 1976: 354.

• *Pityophthorus declivisetosus* Bright, 1977: 525.

• *Pityophthorus degener* Wood, 1975b: 397.

• *Pityophthorus deleoni* (Blackman, 1942b: 201) (*Myeloborus*).

• *Pityophthorus deletus* LeConte, 1879: 519.

= *Pityophthorus inquietus* Blackman, 1928a: 48.

= *Pityophthorus monophyllae* Blackman, 1928a: 47.

= *Pityophthorus socius* Blackman, 1928a: 48.

= *Pityophthorus piceus* Bright, 1966: 297.

= *Pityophthorus prealtus* Bright, 1966: 303.

= *Pityophthorus brucki* Bright, 1971: 63.

• *Pityophthorus delicatus* Wood, 1978: 399.

• *Pityophthorus denticulatus* (Wichmann, 1915a: 106) (*Trigonogenius*).

• *Pityophthorus dentifrons* Blackman, 1922b: 125.

• *Pityophthorus deodara* (Stebbing, 1903: 274) (*Cryphalus*).

= *Cryphalus himalayensis* Stebbing, 1914: 540.

= *Cryphalus sampsoni* Stebbing, 1914: 551.

• *Pityophthorus deprecator* Schaufuss, 1891: 15.

• *Pityophthorus desultorius* Bright, 1985c: 478.

• *Pityophthorus detectus* Schedl, 1972f: 66.

• *Pityophthorus detentus* Wood, 1976: 352.

• *Pityophthorus digestus* (LeConte, 1874: 71) (*Cryphalus*).

= *Pityophthorus hopkinsi* Blackman, 1928a: 57.

= *Pityophthorus idoneus* Blackman, 1928a: 55.

= *Pityophthorus ponderosae* Blackman, 1928a: 57.

= *Pityophthorus aplanatus* Schedl, 1930: 195.

• *Pityophthorus diglyphus* Blandford, 1904: 240.

• *Pityophthorus diligens* Wood, 1976: 363.

• *Pityophthorus dimidiatus* Blackman, 1942b: 221.

• *Pityophthorus diminutivus* Bright, 1985b: 468.

• *Pityophthorus dimorphus* Schedl, 1959b: 551.

= *Neomips brasiliensis* Schedl, 1954b: 38.

• *Pityophthorus discretus* Wood, 1977b: 394.

• *Pityophthorus dispar* Bright, 1976: 431.

• *Pityophthorus dissidens* Bright, 2019: 366.

• *Pityophthorus dissolutus* Wood, 1975b: 398.

• *Pityophthorus diversus* Bright, 1972a: 87.

• *Pityophthorus djuguensis* Eggers, 1940d: 103.

= *Pityophthorus ituriensis* Eggers, 1940d: 104.

• *Pityophthorus dolus* Wood, 1964: 65.

• *Pityophthorus donbrighti* Petrov, 2015: 320.

• *Pityophthorus dorsalis* Schedl, 1953c: 94.

• *Pityophthorus durus* Blackman, 1928a: 70.

• *Pityophthorus eccentricus* Bright, 2019: 368.

• *Pityophthorus eggersi* Schedl, 1952e: 347 (RN).

= *Trigonogenius similis* Eggers, 1920c: 34.

• *Pityophthorus eggersianus* Schedl, 1958i: 144 (RN).

= *Pityophthorus denticulatus* Eggers, 1940a: 129.

= *Pityophthorus guadeloupensis* Nunberg, 1956c: 208 (RN).

• *Pityophthorus elatinus* Wood, 1964: 66.

• *Pityophthorus electus* Blackman, 1928a: 140.

• *Pityophthorus elegans* Schedl, 1938b: 184.

• *Pityophthorus elongatulus* Schedl, 1976: 67.

• *Pityophthorus equihuai* Bright, 1985b: 469.

• *Pityophthorus erraticus* Schedl, 1976: 68.

• *Pityophthorus espinosai* Brèthes, 1925: 202.

• *Pityophthorus eucracens* Wood, 2007: 644.

• *Pityophthorus euterpes* Bright, 1978: 75.

• *Pityophthorus excellens* Schedl, 1972f: 66.

• *Pityophthorus eximius* Schedl, 1938b: 184.

• *Pityophthorus explicitus* Wood, 1975b: 399.

• *Pityophthorus exquisitus* (Blackman, 1942b: 196) (*Neodryocoetes*).

= *Pityophthorus inceptis* Wood, 1975b: 396.

• *Pityophthorus exsculptus* (Ratzeburg, 1837: 162) (*Bostrichus*).

= *Pityophthorus macrographus* Eichhoff, 1881: 200.

• *Pityophthorus exsectus* Schedl, 1972f: 67.

= *Pityophthorus abbreviatus* Schedl, 1972f: 67.

• *Pityophthorus fallax* (Hagedorn, 1912a: 354) (*Trigonogenius*).

• *Pityophthorus favorabilis* Bright, 2019: 370.

• *Pityophthorus festus* Wood, 1967a: 39.

• *Pityophthorus flavimaculatus* Murayama, 1963b: 392.

• *Pityophthorus foleyi* Bright, 2019: 371.

• *Pityophthorus franseriae* Wood, 1971b: 75.

• *Pityophthorus fulgens* Schedl, 1965f: 68.

• *Pityophthorus furnissi* Bright, 1976: 433.

• *Pityophthorus fuscus* Blackman, 1928a: 32.

= *Pityophthorus smithi* Schedl, 1931c: 163.

• *Pityophthorus galeritus* Wood, 1976: 355.

• *Pityophthorus germanus* Bright, 1976: 434.

• *Pityophthorus ghanaensis* Schedl, 1972a: 292.

• *Pityophthorus gimmeli* Bright, 2019: 371.

• *Pityophthorus glabratulus* (Schedl, 1955i: 26) (*Ctenyophthorus*).

• *Pityophthorus glabratus* Eichhoff, 1878b: 179.

• *Pityophthorus glutae* Wood, 1989: 183.

• *Pityophthorus grandis* Blackman, 1928a: 119.

• *Pityophthorus granulipennis* Schedl, 1965f: 69.

• *Pityophthorus gratus* Bright, 2019: 372.

• *Pityophthorus grenadacolens* Bright, 2019: 372.

• *Pityophthorus guatemalensis* Blandford, 1904: 239.

• *Pityophthorus henscheli* Seitner, 1887: 44.

= *Pityophthorus senex* Wichmann, 1913b: 143.

• *Pityophthorus hermosus* Wood, 1976: 356.

• *Pityophthorus hintzi* Schedl, 1938d: 459.

= *Pityophthorus acuminatus* Browne, 1965: 192.

• *Pityophthorus hylocuroides* Wood, 1964: 69.

• *Pityophthorus icicae* Wood, 2007: 642.

• *Pityophthorus ignotus* Schedl, 1961g: 143.

• *Pityophthorus ikelaensis* Nunberg, 1967: 322.

• *Pityophthorus illuminus* Bright, 2019: 373.

• *Pityophthorus imbellis* Wood, 2007: 642.

• *Pityophthorus imitans* (Eggers, 1920c: 34) (*Trigonogenius*).

• *Pityophthorus immanis* Blackman, 1928a: 98.

= *Pityophthorus sulcatus* Bright, 1977: 528.

• *Pityophthorus impexus* Bright, 1978: 76.

• *Pityophthorus inaequidens* Schedl, 1976: 68.

• *Pityophthorus indefessus* Bright, 1986a: 641.

• *Pityophthorus indigens* Wood, 1976: 361.

• *Pityophthorus indigus* Wood, 1978: 398 (RN).

= *Pityophthorus indigens* Wood, 1977a: 214.

= *Pityophthorus irritans* Schedl, 1979c: 127 (RN).

• *Pityophthorus ineditus* Bright, 1976: 434.

• *Pityophthorus infimus* Schedl, 1972f: 68.

• *Pityophthorus infulatus* Blackman, 1928a: 103.

= *Pityophthorus hubbardi* Blackman, 1928a: 105.

= *Pityophthorus mollis* Blackman, 1928a: 104.

= *Pityophthorus novellus* Blackman, 1928a: 96.

• *Pityophthorus ingens* Blackman, 1928a: 124.

• *Pityophthorus inhabilis* Bright, 1986a: 642.

• *Pityophthorus inops* Wood, 1976: 353.

• *Pityophthorus insuetus* Bright, 1985c: 479.

• *Pityophthorus insulatus* Bright, 2019: 374.

• *Pityophthorus intentus* Bright, 1978: 77.

• *Pityophthorus intextus* Swaine, 1917: 29.

= *Pityophthorus shepardi* Blackman, 1922b: 124.

= *Pityophthorus tonsus* Blackman, 1928a: 101.

• *Pityophthorus inusitatus* Bright, 2019: 374.

• *Pityophthorus invisibilis* Petrov, 2015: 322.

• *Pityophthorus irregularis* Eggers, 1931b: 31.

• *Pityophthorus jeffreyi* Blackman, 1928a: 113.

• *Pityophthorus joveri* Schedl, 1954a: 881.

• *Pityophthorus jucundus* Blandford, 1894c: 87.

• *Pityophthorus juglandis* Blackman, 1928a: 42.

• *Pityophthorus kataevi* Petrov & Mandelshtam, 2023: 332.

• *Pityophthorus keeni* (Blackman, 1928a: 19) (*Myeloborus*).

• *Pityophthorus kenyae* Schedl, 1955b: 217.

• *Pityophthorus kirgisicus* Pyatnitskiy, 1931: 167.

• *Pityophthorus kivuensis* Schedl, 1957b: 61.

• *Pityophthorus knoteki* Reitter, 1898: 356.

= *Pityophthorus lichtensteinii robustus* Pfeffer, 1940a: 118.

• *Pityophthorus kuscheli* Schedl, 1952i: 19.

• *Pityophthorus laetus* Wood, 1976: 358.

• *Pityophthorus laevis* (Schedl, 1938b: 181) (*Neopityophthorus*).

= *Pityophthoroides pudens* Blackman, 1942b: 199.

= *Pityophthorus formosus* Bright, 1972a: 88.

• *Pityophthorus languidus* Eichhoff, 1878b: 186.

• *Pityophthorus lapponicus* Kurentsov, 1941: 177.

= *Pityophthorus lapponicus* Stark, 1952: 356.

• *Pityophthorus laticeps* Bright, 1978: 78.

• *Pityophthorus lautus* Eichhoff, 1872a: 135.

= *Pityophthorus rhois* Swaine, 1917: 26.

= *Pityophthorus natalis* Blackman, 1921: 8.

• *Pityophthorus lecontei* Bright, 1977: 525.

• *Pityophthorus leechi* Wood, 1977a: 215.

• *Pityophthorus leiophyllae* Blackman, 1942b: 205.

= *Pityophthorus auctor* Blackman, 1942b: 214.

• *Pityophthorus lenis* Wood, 1976: 348.

• *Pityophthorus lepidus* Bright, 1977: 526.

• *Pityophthorus levis* Wood, 1986a: 273.

• *Pityophthorus lichtensteinii* (Ratzeburg, 1837: 162) (*Bostrichus*).

= *Pityophthorus rossicus* Eggers, 1915a: 13.

• *Pityophthorus liquidambarus* Blackman, 1921: 14.

• *Pityophthorus litos* Bright, 1976: 435.

• *Pityophthorus longipilus* Schedl, 1951c: 112.

• *Pityophthorus madagascariensis* Schedl, 1951e: 21.

• *Pityophthorus malleatus* Bright, 1978: 79.

• *Pityophthorus mandibularis* Schedl, 1951c: 113.

• *Pityophthorus maroantsetrae* Schedl, 1965f: 69.

• *Pityophthorus maslovi* Petrov, 2015: 323.

• *Pityophthorus masneri* Bright, 2019: 378.

• *Pityophthorus mauretanicus* Peyerimhoff, 1930: 259.

• *Pityophthorus medialis* Wood, 1976: 361.

• *Pityophthorus megas* Bright, 1976: 436.

• *Pityophthorus melanurus* Wood, 1976: 364.

• *Pityophthorus mendosus* Wood, 1975b: 397.

• *Pityophthorus mesembria* Bright, 1978: 80.

• *Pityophthorus mexicanus* Blackman, 1928a: 121.

• *Pityophthorus micans* Bright, 1981a: 188.

• *Pityophthorus micrographus micrographus* (Linnaeus, 1758: 355) (*Dermestes micrographus*).

= *Tomicus fabricii* Billberg, 1820: 46 (RN).

= *Pityophthorus fennicus* Eggers, 1914b: 183.

= *Pityophthorus micrographus curtulus* Sokanovskiy, 1954: 19.

= *Pityophthorus micrographus longulus* Sokanovskiy, 1954: 19.

• *Pityophthorus micrographus sibiricus* Stark, 1952: 344.

• *Pityophthorus miniatus* Bright, 1981a: 280.

• *Pityophthorus minimus* Wood, 2007: 637.

• *Pityophthorus minus* Bright, 1976: 437.

• *Pityophthorus minutalis* Wood, 1976: 357.

• *Pityophthorus minutissimus* Bright, 2019: 379.

• *Pityophthorus minutus* Schedl, 1963f: 221.

• *Pityophthorus modicus* Blackman, 1928a: 94.

= *Pityophthorus navus* Blackman, 1928a: 95.

• *Pityophthorus molestus* Wood, 1976: 361.

• *Pityophthorus montezumae* Bright, 1978: 81.

• *Pityophthorus montivagus* Bright, 1977: 527.

• *Pityophthorus moritzi* Wood, 2007: 655.

• *Pityophthorus mormon* Bright, 1977: 528.

• *Pityophthorus morosovi* Spessivtsev, 1926a: 48.

• *Pityophthorus morosus* Wood, 1976: 362.

• *Pityophthorus mpossae* Schedl, 1957b: 63.

• *Pityophthorus muluensis* Bright, 2000: 49.

• *Pityophthorus mulungensis* Schedl, 1957b: 60.

• *Pityophthorus murrayanae* Blackman, 1922c: 138.

= *Pityophthorus cutleri* Swaine, 1925b: 195.

= *Pityophthorus elongatus* Swaine, 1925b: 194.

= *Pityophthorus exilis* Swaine, 1925b: 196.

= *Pityophthorus gracilis* Swaine, 1925b: 195.

= *Pityophthorus tenuis* Swaine, 1925b: 196.

= *Pityophthorus depygis* Blackman, 1928a: 128.

= *Pityophthorus watsoni* Schedl, 1930: 197.

= *Pityophthorus aurulentus* Bright, 1966: 301.

= *Pityophthorus acceptus* Bright, 1981a: 315.

• *Pityophthorus nanus* Wood, 1964: 64.

• *Pityophthorus nebulosus* Wood, 1976: 355.

• *Pityophthorus nectandrae* Wood, 2007: 638.

• *Pityophthorus nemoralis* Wood, 1976: 351.

• *Pityophthorus nesocolus* Bright, 2019: 379.

• *Pityophthorus niger* Schedl, 1938b: 187.

• *Pityophthorus nigricans* Blandford, 1904: 236.

= *Pityophthorus chiapensis* Bright, 1977: 522.

• *Pityophthorus nigriceps* Wood, 2007: 633.

• *Pityophthorus nitellus* Browne, 1973: 291.

• *Pityophthorus nitidulus* (Mannerheim, 1843: 298) (*Bostrichus*).

= *Cryphalus atratulus* LeConte, 1868: 156.

= *Cryphalus puncticollis* LeConte, 1874: 71.

• *Pityophthorus nitidus* Swaine, 1917: 25.

= *Pityophthorus borealis* Swaine, 1925b: 195.

= *Pityophthorus anceps* Blackman, 1928a: 31.

= *Pityophthorus varians* Schedl, 1930: 196.

= *Pityophthorus aquilonius* Bright, 1968a: 604.

• *Pityophthorus nocturnus* Schedl, 1938b: 185.

= *Pityophthorus hidalgoensis* Blackman, 1942b: 215.

• *Pityophthorus novateutonicus* Schedl, 1964j: 205.

• *Pityophthorus nugalis* Wood, 1976: 356.

• *Pityophthorus obtusipennis* Blandford, 1904: 240.

• *Pityophthorus obtusus* Schaufuss, 1891: 17.

• *Pityophthorus occidentalis* Blackman, 1920b: 4.

• *Pityophthorus occlusus* Bright, 1976: 437.

• *Pityophthorus olivierai* Schedl, 1972f: 68.

• *Pityophthorus opacifrons* Wood, 2007: 643.

• *Pityophthorus opaculus* LeConte, 1878b: 623.

= *Pityophthorus abietis* Blackman, 1928a: 49.

= *Pityophthorus albertensis* Blackman, 1928a: 50.

= *Pityophthorus exiguus* Blackman, 1928a: 51.

= *Pityophthorus pygmaeus* Schedl, 1931c: 165.

• *Pityophthorus orarius* Bright, 1968a: 607.

• *Pityophthorus ornatus* Blackman, 1928a: 102.

= *Pityophthorus kenti* Blackman, 1928a: 141.

= *Pityophthorus limatus* Wood, 1964: 65.

• *Pityophthorus ostryacolens* Bright, 1986b: 681.

• *Pityophthorus pampasae* Schedl, 1970e: 92.

• *Pityophthorus parfentievi* Pyatnitskiy, 1931: 169.

= *Pityophthorus schrenkianae* Pyatnitskiy, 1931: 171.

• *Pityophthorus parilis* Wood, 1976: 359.

• *Pityophthorus pauculus* Bright, 2019: 380.

• *Pityophthorus paulus* Wood, 1964: 63.

• *Pityophthorus pecki* Atkinson, 1993b: 613.

• *Pityophthorus pellitus* Schedl, 1955i: 23.

• *Pityophthorus pentaclethrae* Schedl, 1957b: 62.

• *Pityophthorus peregrinus* Eichhoff, 1878b: 193.

• *Pityophthorus perexiguus* Wood, 1976: 355.

• *Pityophthorus perotei* Blackman, 1942b: 218.

• *Pityophthorus philippinensis* Schedl, 1936d: 59.

• *Pityophthorus pinavorus* Bright, 1985a: 182.

• *Pityophthorus pinguis* (Blackman, 1928a: 17) (*Myeloborus*).

• *Pityophthorus pini* Kurentsov, 1941: 176.

• *Pityophthorus pinsapo* Pfeffer, 1982: 154.

• *Pityophthorus pityographus cribratus* Pfeffer, 1940a: 116.

= *Pityophthorus pityographus maritimus* Stark, 1952: 344.

• *Pityophthorus pityographus pityographus* (Ratzeburg, 1837: 162) (*Bostrichus pityographus*).

= *Pityophthorus pityographus bibractensis* Balachowsky, 1949b: 236.

• *Pityophthorus podocarpi* Wood, 2007: 641.

• *Pityophthorus procerus* Bright, 2019: 382.

• *Pityophthorus pseudotsugae* Swaine, 1918: 99.

= *Pityophthorus thatcheri* Bright, 1976: 442.

• *Pityophthorus puberulus* (LeConte, 1868: 157) (*Cryphalus*).

= *Pityophthorus infans* Eichhoff, 1872a: 135.

• *Pityophthorus pubifrons* Bright, 1981a: 195.

• *Pityophthorus pudicus* Blackman, 1942b: 208.

• *Pityophthorus pulchellus* Eichhoff, 1869: 275.

= *Pityophthorus hirticeps* LeConte, 1878b: 623.

= *Pityophthorus pusio* LeConte, 1878b: 623.

• *Pityophthorus pulicarius* (Zimmermann, 1868: 144) (*Crypturgus*).

= *Scolytus strobi* Peck, 1817: 207.

= *Pityophthorus cubensis* Schedl, 1972f: 65.

• *Pityophthorus pullus* (Zimmermann, 1868: 143) (*Crypturgus*).

= *Pityophthorus bisulcatus* Eichhoff, 1869: 274.

= *Pityophthorus cribripennis* Eichhoff, 1869: 274.

• *Pityophthorus punctatus* Eggers, 1940a: 130.

• *Pityophthorus punctifrons* Bright, 1966: 298.

• *Pityophthorus punctiger* (Schedl, 1951c: 108) (*Neodryocoetes*).

• *Pityophthorus pygmaeolus* Schedl, 1970e: 93.

• *Pityophthorus quadrispinatus* Schedl, 1966d: 110.

= *Pityophthorus gunneri* Schedl, 1970c: 583.

= *Pityophthorus roppae* Schedl, 1976: 69.

• *Pityophthorus quercinus* Wood, 1967a: 40.

• *Pityophthorus ramiperda* Swaine, 1917: 28.

= *Myeloborus fivasi* Blackman, 1928a: 23.

• *Pityophthorus ramulorum* (Perris, 1856: 191) (*Tomicus*).

= *Ips pubescens* Marsham, 1802: 58.

• *Pityophthorus recens* Bright, 1976: 438.

• *Pityophthorus regularis* Blackman, 1942b: 206.

• *Pityophthorus reticulatus* Wood, 2007: 646.

• *Pityophthorus retifrons* Wood, 2007: 644.

• *Pityophthorus robai* (Blackman, 1942b: 200) (*Pityophthoroides*).

• *Pityophthorus rogueti* Bright, 2019: 384.

• *Pityophthorus rubidus* Wood, 1978: 400.

• *Pityophthorus rudis* Blackman, 1942b: 212.

• *Pityophthorus sachalinensis* Krivolutskaya, 1956b: 838.

• *Pityophthorus sambuci* Blackman, 1942b: 207.

• *Pityophthorus sapineus* Bright, 1981a: 194.

• *Pityophthorus scabridus* Schedl, 1955i: 24.

• *Pityophthorus scalptor* Blackman, 1928a: 30.

• *Pityophthorus scalptus* Bright, 1978: 82.

• *Pityophthorus schwarzi* Blackman, 1928a: 71.

• *Pityophthorus schwerdtfegeri* (Schedl, 1955i: 28) (*Conophthorus*).

= *Pityophthorus islasi* Wood, 1962: 80.

= *Conophthocranulus islasi* Schedl, 1963c: 163.

• *Pityophthorus scitulus* Wood, 1976: 359.

• *Pityophthorus scriptor* Blackman, 1921: 7.

• *Pityophthorus segnis* Blackman, 1928a: 52.

• *Pityophthorus seiryuensis* Murayama, 1963b: 393.

• *Pityophthorus senticosus* Bright, 2019: 386.

• *Pityophthorus separatus* Bright, 1977: 529.

• *Pityophthorus sepositus* Bright, 2019: 386.

• *Pityophthorus serratus* Swaine, 1918: 103.

• *Pityophthorus setifer* Browne, 1965: 193.

• *Pityophthorus setosus* Blackman, 1928a: 77.

• *Pityophthorus sextuberculatus* Eggers, 1933f: 6.

• *Pityophthorus sichotensis* Kurentsov, 1941: 175.

• *Pityophthorus sierrensis* Bright, 1971: 64.

• *Pityophthorus signatifrons* Browne, 1975: 758.

• *Pityophthorus similaris* Wood, 2007: 635.

• *Pityophthorus similis* Eichhoff, 1869: 275.

• *Pityophthorus simplicis* Wood, 2007: 635.

• *Pityophthorus sinopae* Schedl, 1976: 69.

• *Pityophthorus sobrinus* Wood, 1976: 357.

• *Pityophthorus solatus* Wood, 1977a: 215.

• *Pityophthorus solers* Blackman, 1928a: 138.

• *Pityophthorus solus* Blackman, 1928a: 64.

= *Pityophthorus cribratus* Blackman, 1942b: 216.

• *Pityophthorus spadix* Blackman, 1942b: 219.

• *Pityophthorus sparsepilosus* (Schedl, 1935f: 343) (*Gnathophorus*).

• *Pityophthorus speciosus* Wood, 1977a: 215.

• *Pityophthorus speculum* Bright, 1976: 440.

• *Pityophthorus splendens* Wood, 2007: 638.

• *Pityophthorus strictus* Wood, 1976: 354.

• *Pityophthorus subconcentralis* Schedl, 1938b: 183.

= *Pityophthorus hispaniolus* Bright, 1985a: 181.

• *Pityophthorus subcribratus* Schedl, 1937b: 168.

= *Pityophthorus zeteki* Blackman, 1942b: 226.

• *Pityophthorus subopacus* Blackman, 1942b: 210.

= *Pityophthorus elimatus* Bright, 1976: 432.

• *Pityophthorus subsimilis* Schedl, 1955i: 25.

= *Pityophthorus subimpressus* Bright, 1976: 441.

• *Pityophthorus subtilus* Bright, 2019: 388.

• *Pityophthorus surinamensis* Schedl, 1961h: 226.

• *Pityophthorus suspicious* Bright, 1972a: 89.

• *Pityophthorus suturalis* Eggers, 1932c: 291.

• *Pityophthorus tenax* Wood, 1976: 354.

• *Pityophthorus terebrans* Schedl, 1970a: 223 (RN).

= *Pityophthorus granulipennis* Schedl, 1966d: 111.

= *Pityophthorus apicenotatus* Schedl, 1976: 66.

= *Pityophthorus granulicauda* Schedl, 1979f: 112 (RN).

• *Pityophthorus thamnus* Bright, 1985b: 470.

• *Pityophthorus thomasi* Bright, 1976: 443.

• *Pityophthorus timidulus* Wood, 1975b: 396.

• *Pityophthorus timidus* Blandford, 1904: 241.

• *Pityophthorus tishechkini* Petrov, 2015: 324.

• *Pityophthorus togonus* Eggers, 1920c: 35.

• *Pityophthorus tomentosus* Bright, 2019: 389.

• *Pityophthorus toralis* Wood, 1964: 59.

= *Myeloborus confusus* Bright, 1966: 295.

= *Pityophthorus collinus* Bright, 1968a: 605.

• *Pityophthorus torresi* Bright, 2019: 390.

• *Pityophthorus torridus* Wood, 1971b: 76.

• *Pityophthorus tragardhi* Spessivtsev, 1921: 219.

• *Pityophthorus treculiae* Schedl, 1965g: 1080.

• *Pityophthorus trepidus* Bright, 1978: 83.

• *Pityophthorus trunculus* Bright, 1985b: 470.

• *Pityophthorus tuberculatus* Eichhoff, 1878b: 498.

= *Pityophthorus rugicollis* Swaine, 1925b: 193.

= *Pityophthorus tuberculatus australis* Blackman, 1928a: 94.

• *Pityophthorus tucumanensis* Wood, 2007: 645.

• *Pityophthorus tumidus* Blackman, 1928a: 58.

• *Pityophthorus turbiculus* Schedl, 1938b: 187.

• *Pityophthorus tutulus* Bright, 1986a: 643.

• *Pityophthorus vegrandis* Bright, 1986a: 643.

• *Pityophthorus venezuelensis* Schedl, 1935c: 91.

• *Pityophthorus venustus* Blackman, 1928a: 75.

= *Pityophthorus artifex* Blackman, 1928a: 76.

• *Pityophthorus vesculus* Wood, 1978: 401.

• *Pityophthorus vescus* Wood, 2007: 654.

• *Pityophthorus vespertinus* Bright, 1978: 83.

• *Pityophthorus vilcabambensis* Petrov, 2015: 327.

• *Pityophthorus viminalis* Bright, 1977: 530.

• *Pityophthorus virilis* Blackman, 1928a: 143.

= *Pityophthorus fortis* Blackman, 1928a: 142.

• *Pityophthorus volvulus* Schedl, 1965e: 10.

• *Pityophthorus vrydaghi* Nunberg, 1973: 13.

• *Pityophthorus vulgaris* Bright, 2019: 391.

• *Pityophthorus woodi* Bright, 1977: 531.

• *Pityophthorus youngi* Bright, 2019: 391.

• *Pityophthorus zexmenivora* Bright, 1985b: 471.

• *Pityophthorus zonalis* Bright, 1976: 443.

#### *Pityotrichus* Wood

3 spp.

• *Pityotrichus barbatus* (Blackman, 1928a: 147) (*Pityophilus*).

• *Pityotrichus hesperius* Bright, 1971: 69.

• *Pityotrichus turkmenicus* Mandelshtam & Petrov in: Mandelshtam et al. 2006: 214.

#### *Pseudopityophthorus* Swaine

28 spp.

• *Pseudopityophthorus absitus* Bright, 2019: 392.

• *Pseudopityophthorus agrifoliae* Blackman, 1931a: 230.

• *Pseudopityophthorus asperulus* (LeConte, 1868: 155) (*Cryphalus*).

= *Pseudopityophthorus gracilis* Blackman, 1921: 6.

• *Pseudopityophthorus cincinnatus* (Blandford, 1904: 242) (*Pityophthorus*).

• *Pseudopityophthorus colombianus* Wood, 1971a: 51.

• *Pseudopityophthorus comosus* Bright, 1972d: 1670.

• *Pseudopityophthorus declivis* Wood, 1971a: 50.

= *Pseudopityophthorus curtus* Bright, 1972d: 1674.

= *Pseudopityophthorus truncatus* Bright, 1972d: 1673.

• *Pseudopityophthorus denticulus* Wood, 1977a: 216.

• *Pseudopityophthorus durangoensis* Wood, 1987: 549.

• *Pseudopityophthorus fagi* Blackman, 1931a: 228.

• *Pseudopityophthorus festivus* Wood, 1974a: 55.

• *Pseudopityophthorus granulatus* Blackman, 1931a: 230.

• *Pseudopityophthorus granulifer* Wood, 1967a: 42.

• *Pseudopityophthorus hispidus* Eggers, 1930a: 170.

• *Pseudopityophthorus hondurensis* Wood, 1967a: 42.

= *Pseudopityophthorus montanus* Bright, 1972d: 1667.

• *Pseudopityophthorus limbatus* Eggers, 1930a: 169.

= *Pseudopityophthorus micans* Wood, 1967a: 44.

• *Pseudopityophthorus minutissimus* (Zimmermann, 1868: 143) (*Crypturgus*).

• *Pseudopityophthorus opacicollis* Blackman, 1931a: 235.

= *Pseudopityophthorus aesculinus* Bright, 1972d: 1672.

• *Pseudopityophthorus peregrinus* Wood & Yin, 1986: 462.

• *Pseudopityophthorus pruinosus* (Eichhoff, 1878b: 198) (*Pityophthorus*).

= *Pityophthorus tomentosus* Eichhoff, 1878b: 201.

= *Pityophthorus querciperda* Schwarz, 1888: 56.

= *Pseudopityophthorus pulvereus* Blackman, 1931a: 232.

= *Pseudopityophthorus tropicalis* Wood, 1967a: 43.

= *Pseudopityophthorus convexus* Bright, 1972d: 1672.

• *Pseudopityophthorus pubescens* Blackman, 1931a: 229.

• *Pseudopityophthorus pubipennis* (LeConte, 1857: 59) (*Bostrichus*).

• *Pseudopityophthorus singularis* Wood, 1971a: 50.

= *Pseudopityophthorus acuminatus* Bright, 1972d: 1671.

• *Pseudopityophthorus squamosus* Bright, 1972d: 1670.

• *Pseudopityophthorus tenuis* Wood, 1959a: 1.

= *Pseudopityophthorus hirsutus* Bright, 1972d: 1668.

• *Pseudopityophthorus virilis* Wood, 1971a: 50.

• *Pseudopityophthorus xalapae* Wood, 1987: 549.

• *Pseudopityophthorus yavapaii* Blackman, 1931a: 233.

#### *Sauroptilius* Browne

1 sp.

• *Sauroptilius sauropterus* (Schedl, 1953c: 101) (*Xyleborus*).

= *Sauroptilius sauropteroides* Schedl, 1970b: 237.

#### *Spermophthorus* Costa Lima

2 spp.

• *Spermophthorus aberrans* Wood, 1968a: 11.

• *Spermophthorus apuleiae* Costa Lima, 1929: 111.

= *Conophthocranulus vianai* Schedl, 1938f: 27.

= *Spermophthorus caesalpiniae* Blackman, 1942b: 204.

#### *Sphenoceros* Bright

3 spp.

• *Sphenoceros antillicus* Bright, 2019: 394.

• *Sphenoceros aztecus* (Wood, 1971a: 54) (Sphenoceros*).

• *Sphenoceros limax* (Schedl, 1939d: 566) (Sphenoceros*).

#### *Stegomerus* Wood

9 spp.

• *Stegomerus chiriquensis* Wood, 1967c: 131.

• *Stegomerus diversus* Bright, 2019: 181.

• *Stegomerus longipennis* Wood, 2007: 482.

• *Stegomerus mexicanus* Wood, 1967c: 133.

• *Stegomerus mirandus* Wood, 1971a: 32.

• *Stegomerus montanus* Wood, 1967c: 132.

• *Stegomerus parvatis* (Wood, 1974a: 17) (*Cryphalomorphus*).

• *Stegomerus pygmaeus* Wood, 1967c: 130.

• *Stegomerus vulgaris* Wood, 1967c: 134.

#### *Styphlosoma* Blandford

4 spp.

• *Styphlosoma boliviae* (Schedl, 1961h: 224) (*Stephanopodius*).

• *Styphlosoma brasiliense* (Schedl, 1972f: 58) (*Stephanopodius brasiliensis*).

• *Styphlosoma granulatum* Blandford, 1904: 232.

• *Styphlosoma subulatum* Wood, 1971a: 43.

#### *Trypolepis* Bright

1 sp.

• *Trypolepis antillicus* Bright, 2019: 182.

### UDZUNGWANINA Jordal

1 genus, 1 sp.

#### *Udzungwana* Jordal

1 sp.

• *Udzungwana bispinosa* Jordal, 2025a: 2.

### CRYPHALINI Lindemann

1 genus, 256 spp.

#### *Cryphalus* Erichson

256 spp.

• *Cryphalus abbreviatus* Schedl, 1943: 35.

• *Cryphalus abietis* (Ratzeburg, 1837: 163) (*Bostrichus*).

• *Cryphalus aciculatus* (Schedl, 1939b: 341) (*Hypocryphalus*).

• *Cryphalus afiamalus* (Schedl, 1951f: 148) (*Hypocryphalus*).

• *Cryphalus amplicollis* Johnson in: Johnson et al. 2020a: 20 (RN).

= *Cryphalus laticollis* Browne, 1984b: 288.

• *Cryphalus angustior* Eggers, 1927e: 395.

• *Cryphalus aquilonius* (Nobuchi, 1975: 52) (*Taenioglyptes*).

• *Cryphalus araucariae* Schedl, 1970h: 214.

• *Cryphalus armatus* Schedl, 1974a: 459.

• *Cryphalus artocarpus* (Schedl, 1939b: 342) (*Ericryphalus*).

= *Cryphalus artocarpus* Schedl, 1958h: 498.

= *Cryphalus brownei* Wood, 1992: 79 (RN).

• *Cryphalus asper* (Broun, 1881: 742) (*Tomicus*).

• *Cryphalus asperulus* Schedl, 1948b: 26.

• *Cryphalus ater* Browne, 1984e: 91.

• *Cryphalus babai* Murayama, 1961: 29.

• *Cryphalus bakeri* (Eggers, 1927b: 77) (*Stephanoderes*).

• *Cryphalus balanopselaphus* Eggers, 1920b: 121.

• *Cryphalus basihirtus* Beeson, 1929: 227.

• *Cryphalus bellus* Schedl, 1957b: 47.

• *Cryphalus bicarinatus* (Nobuchi, 1975: 53) (*Taenioglyptes*).

• *Cryphalus bicolor* (Browne, 1983c: 69) (*Cryphalomorphus*).

• *Cryphalus bidentatus* (Browne, 1980b: 383) (*Hypocryphalus*).

• *Cryphalus boettcheri* Schedl, 1943: 38.

• *Cryphalus borneensis* Schedl, 1943: 38.

• *Cryphalus brasiliensis* Schedl, 1976: 65.

• *Cryphalus brevior* (Schedl, 1943: 40) (*Hypocryphalus*).

• *Cryphalus brevipilosus* Schedl, 1942c: 173.

• *Cryphalus brimblecombei* Schedl, 1948b: 26.

• *Cryphalus brownei* (Beaver, 1991: 53) (*Hypothenemus*) (RN).

= *Cryphalomorphus striatulus* Browne in: Beaver and Browne 1979: 584.

• *Cryphalus brunneus* Browne, 1984e: 93.

• *Cryphalus buloloensis* Browne, 1983c: 67.

• *Cryphalus capucinicollis* Schedl, 1950e: 47.

• *Cryphalus capucinoides* Eggers, 1939b: 4.

• *Cryphalus capucinomorphus* Schedl, 1950e: 48.

• *Cryphalus capucinus* Schedl, 1938e: 425.

• *Cryphalus carinatus* (Browne, 1980b: 382) (*Margadillius*).

• *Cryphalus carpini* Berger, 1917: 234.

• *Cryphalus carpinivorus* Murayama, 1930: 14.

• *Cryphalus chamaecipariae* Niisima, 1910: 10.

• *Cryphalus chinlingensis* Tsai & Li, 1963: 605.

• *Cryphalus ciliatipes* Blandford, 1896a: 242.

• *Cryphalus cinereotestaceus* (Motschulsky, 1866: 403) (*Hypoborus*).

• *Cryphalus compactus* Lea, 1910: 139.

• *Cryphalus confusus* (Hopkins, 1915d: 38) (*Margadillius*).

• *Cryphalus constrictus* Schedl, 1942c: 174.

= *Hypocryphalus constrictus* Schedl, 1942d: 22.

= *Cryphalus perminimus* Schedl, 1942d: 13.

= *Hypocryphalus froggatti* Nunberg, 1961a: 611.

• *Cryphalus corpulentus* (Schedl, 1942d: 22) (*Hypocryphalus*).

• *Cryphalus coryli* Stark, 1936: 144.

• *Cryphalus cryptomeriae* Niisima, 1908: 91.

• *Cryphalus cylindricus* Browne, 1980b: 384.

• *Cryphalus cylindripennis* (Schedl, 1959i: 483) (*Hypocryphalus*).

• *Cryphalus cylindrus* (Browne, 1950: 647) (*Dacryphalus*).

• *Cryphalus densepilosus* (Schedl, 1942d: 21) (*Hypocryphalus*).

• *Cryphalus dilutus* Eichhoff, 1878a: 384.

= *Cryphalus dilutus* Eichhoff, 1878b: 490.

• *Cryphalus dipterocarpi* Wood, 1989: 179.

• *Cryphalus discrepans* (Schedl, 1965f: 58) (*Hypocryphalus*).

• *Cryphalus discretus* Eichhoff, 1878b: 490.

= *Cryphalus scabricollis* Eichhoff, 1878b: 491.

= *Cryphalus brevisetosus* Schedl, 1943: 36.

• *Cryphalus dissimilis* (Nobuchi, 1975: 57) (*Taenioglyptes*).

• *Cryphalus diversicolor* Browne, 1984e: 90.

• *Cryphalus dorsalis* (Motschulsky, 1866: 403) (*Hypoborus*).

= *Hypoborus nebulosus* Motschulsky, 1866: 403.

= *Hylesinus sericeus* Motschulsky, 1866: 402.

= *Cryphalus indicus* Eichhoff, 1878a: 384.

= *Cryphalus indicus* Eichhoff, 1878b: 489.

• *Cryphalus dubiosus* Schedl, 1963d: 66.

• *Cryphalus duplosquamosus* Schedl, 1942d: 15.

• *Cryphalus eggersi* Johnson in: Johnson et al. 2020a: 21 (RN).

= *Cryphalus confusus* Eggers, 1927e: 395.

• *Cryphalus elaboratus* Schedl, 1950e: 51.

• *Cryphalus elongatus* Schedl, 1962d: 105.

• *Cryphalus eriobotryae* Johnson in: Zheng et al. 2019: 3.

• *Cryphalus erraticus* Schedl, 1979e: 96 (E).

• *Cryphalus erythrinae* (Hopkins, 1915d: 38) (*Margadillius*).

• *Cryphalus exiguus* Blandford, 1894c: 82.

= *Cryphalus pilosus* Sasaki, 1899: 238.

• *Cryphalus felis* Wood, 1989: 180.

• *Cryphalus fici* (Browne, 1986a: 90) (*Hypocryphalus*).

• *Cryphalus ficivorus* Murayama, 1958: 933.

• *Cryphalus formosanus* Schedl, 1942b: 175.

• *Cryphalus fugax* Schedl, 1973d: 87.

• *Cryphalus fuliginosus* Blandford, 1895: 319.

• *Cryphalus fulmineus* Wood, 1989: 180.

• *Cryphalus fulvus* Niisima, 1908: 92.

= *Cryphalus pini* Eggers, 1921: 39.

• *Cryphalus furukawai* Murayama, 1934b: 59.

• *Cryphalus fuscus* Johnson in: Johnson et al. 2020a: 21 (RN).

= *Cryphalus cylindrus* Browne, 1984e: 92.

• *Cryphalus garambaensis* Nunberg, 1965: 25.

• *Cryphalus garciniae* Nobuchi, 1959b: 24.

• *Cryphalus gigas* Schedl, 1975h: 218.

• *Cryphalus glabratus* (Schedl, 1959i: 484) (*Hypocryphalus*).

• *Cryphalus gnetivorus* Johnson in: Johnson et al. 2020b: 34.

• *Cryphalus gracilis* Johnson in: Johnson et al. 2020a: 22 (RN).

= *Cryphalus laevis* Browne, 1984b: 288.

• *Cryphalus granulatus* (Schedl, 1942b: 176) (*Hypocryphalus*).

• *Cryphalus helopioides* Schedl, 1953f: 295.

• *Cryphalus himalayensis* Buhroo & Zubair, 2024: 12.

• *Cryphalus hirsutus* (Nobuchi, 1975: 52) (*Taenioglyptes*).

• *Cryphalus horridus* Eichhoff, 1878a: 384.

= *Cryphalus horridus* Eichhoff, 1878b: 488.

• *Cryphalus imitans* (Schedl, 1951f: 148) (*Hypocryphalus*).

• *Cryphalus infimus* Schedl, 1972a: 287.

• *Cryphalus intermedius* Ferrari, 1867: 79.

• *Cryphalus interponens* (Schedl, 1953d: 126) (*Hypocryphalus*).

• *Cryphalus itinerans* Johnson in: Johnson et al. 2020b: 37.

• *Cryphalus jeholensis* Murayama, 1939: 143.

• *Cryphalus jezoensis* Inouye & Nobuchi, 1957: 49.

• *Cryphalus jugransi* Niisima, 1913: 3.

• *Cryphalus kagoshimensis* (Nobuchi, 1975: 56) (*Taenioglyptes*).

• *Cryphalus kalambanganus* (Schedl, 1943: 39) (*Hypocryphalus*).

• *Cryphalus kivuensis* Schedl, 1957b: 48.

• *Cryphalus kolbei* (Hagedorn, 1912a: 355) (*Allarthrum*).

• *Cryphalus kurenzovi* Stark, 1936: 142.

= *Cryphalus punctulatus* Eggers, 1942a: 29.

= *Cryphalus ussuriensis* Eggers, 1942a: 29.

• *Cryphalus kyotoensis* Nobuchi, 1966: 53.

• *Cryphalus laevis* (Browne, 1980a: 374) (*Hypocryphalus*).

• *Cryphalus laricis* Niisima, 1909: 142.

• *Cryphalus laticollis* (Browne, 1974a: 67) (*Hypocryphalus*).

• *Cryphalus latus* Eggers, 1929c: 10.

= *Cryphalus premayaensis* Murayama, 1943: 97.

• *Cryphalus lepocrinus* Tsai & Li, 1963: 606.

• *Cryphalus lipingensis* Tsai & Li, 1959: 90.

= *Cryphalus kesiyae* Browne in: Beaver and Browne 1975: 288.

• *Cryphalus longior* Browne, 1984e: 91.

• *Cryphalus longipennis* (Browne, 1970: 552) (*Taenioglyptes*).

• *Cryphalus longipilis* (Browne, 1981a: 128) (*Hypocryphalus*).

• *Cryphalus longipilus* Schedl, 1943: 34.

• *Cryphalus longisetosus* (Nobuchi, 1975: 57) (*Taenioglyptes*).

• *Cryphalus longus* (Eggers, 1926a: 136) (*Ernoporus*).

= *Cryphalus alni* Krivolutskaya, 1958: 145.

• *Cryphalus luteus* Johnson in: Johnson et al. 2020a: 22 (RN).

= *Margadillius fulvus* Browne, 1984b: 289.

• *Cryphalus magnus* (Browne, 1984e: 88) (*Margadillius*).

• *Cryphalus major* Stebbing, 1903: 270.

= *Cryphalus morinda* Stebbing, 1903: 265.

• *Cryphalus malayensis* (Schedl, 1942b: 176) (*Hypocryphalus*).

• *Cryphalus malloti* Schedl, 1943: 37.

• *Cryphalus malus* Niisima, 1909: 144.

= *Cryphalus padi* Krivolutskaya, 1958: 144.

• *Cryphalus mandschuricus* Eggers, 1929c: 10.

• *Cryphalus mangiferae* Stebbing, 1914: 542.

= *Cryphalus inops* Eichhoff, 1872a: 131.

= *Hypothenemus griseus* Blackburn in: Blackburn and Sharp 1885: 194.

= *Hypocryphalus mangiferae* Eggers, 1928a: 85.

= *Hypocryphalus mangiferae* Beeson, 1929: 226.

= *Cryphalus mimicus* Schedl, 1942d: 17.

= *Hypocryphalus opacus* Schedl, 1942d: 20.

= *Cryphalus subcylindricus* Schedl, 1942d: 16.

= *Taenioglyptes artestriatus* Browne, 1970: 553.

• *Cryphalus margadilaonis* (Hopkins, 1915d: 38) (*Margadillius*).

• *Cryphalus markangensis* Tsai & Li, 1963: 606.

• *Cryphalus massonianus* Tsai & Li, 1963: 605.

• *Cryphalus meridionalis* (Nobuchi, 1975: 55) (*Taenioglyptes*).

• *Cryphalus mindoroensis* (Schedl, 1943: 39) (*Hypocryphalus*).

• *Cryphalus minimus* Eggers, 1927b: 76.

• *Cryphalus minor* (Schedl, 1943: 40) (*Hypocryphalus*).

• *Cryphalus minusculus* Johnson in: Johnson et al. 2020a: 22 (RN).

= *Hypocryphalus minutus* Browne, 1980d: 495.

• *Cryphalus minutus* (Hopkins, 1915d: 37) (*Margadillius*).

• *Cryphalus miyalopiceus* Tsai & Li, 1963: 602.

• *Cryphalus montanus* Nobuchi, 1964: 129.

• *Cryphalus moorei* (Schedl, 1964e: 247) (*Hypocryphalus*).

• *Cryphalus morivorus* Johnson in: Johnson et al. 2020b: 51.

• *Cryphalus nataliyae* Mandelshtam & Petrov, 2022: 160.

• *Cryphalus neglectus* Schedl, 1962d: 106.

• *Cryphalus negrosensis* Browne, 1979: 85.

• *Cryphalus niger* Schedl, 1942c: 172.

• *Cryphalus nigericus* Browne, 1973: 289.

• *Cryphalus nigricans* Schedl, 1943: 35.

• *Cryphalus nigrosetosus* (Schedl, 1948b: 27) (*Hypocryphalus*).

• *Cryphalus niponensis* Inouye & Nobuchi, 1957: 51.

• *Cryphalus nitens* Browne, 1980d: 494.

• *Cryphalus nitidicollis* (Schedl, 1975h: 219) (*Hypocryphalus*).

• *Cryphalus nitidipennis* Browne, 1984e: 89.

• *Cryphalus nothofagi* Browne, 1983c: 68.

• *Cryphalus numidicus* Eichhoff, 1878a: 385.

= *Cryphalus numidicus* Eichhoff, 1878b: 487.

• *Cryphalus nyalubombeae* Schedl, 1957b: 49.

• *Cryphalus obesus* (Hopkins, 1915d: 42) (*Dacryphalus*).

• *Cryphalus oblongus* Niisima, 1910: 9.

• *Cryphalus obscurus* (Hopkins, 1915d: 42) (*Hypocryphalus*).

• *Cryphalus ovalicollis* (Schedl, 1942c: 177) (*Hypocryphalus*).

• *Cryphalus ozopemoides* Johnson in: Johnson et al. 2020a: 23 (RN).

= *Hypocryphalus montanus* Schedl, 1974a: 460.

• *Cryphalus paganus* Eichhoff, 1878b: 474.

• *Cryphalus palawanus* Schedl, 1942b: 174.

• *Cryphalus pallidus* Eichhoff, 1872a: 131.

• *Cryphalus papuanus* (Schedl, 1973d: 87) (*Margadillius*).

= *Ernoporus antennarius* Schedl, 1974a: 461.

• *Cryphalus paramangiferae* Johnson in: Johnson et al. 2020b: 54.

• *Cryphalus parvulus* Niisima, 1910: 8.

• *Cryphalus pellicius* Johnson in: Johnson et al. 2020a: 23 (RN).

= *Hypocryphalus pilifer* Schedl, 1979e: 98.

• *Cryphalus pexus* Schedl, 1979g: 104.

• *Cryphalus piceae* (Ratzeburg, 1837: 163) (*Bostrichus*).

= *Cryphalus piceae orientalis* Eggers, 1911b: 122.

= *Cryphalus hattori* Kôno, 1938: 67.

= *Cryphalus subdepressus* Eggers, 1940g: 37.

• *Cryphalus piceus* Eggers, 1926a: 136.

• *Cryphalus pilifer* Eggers, 1927e: 394.

• *Cryphalus piliger* (Schedl, 1975h: 219) (*Hypocryphalus*).

• *Cryphalus pilosellus* Erichson, 1842: 212.

• *Cryphalus pilosulus* Browne, 1980b: 385.

• *Cryphalus pilosus* Tsai & Li, 1963: 606.

• *Cryphalus pini* (Hopkins, 1915d: 39) (*Piperius*).

• *Cryphalus planicollis* Schedl, 1978b: 74.

• *Cryphalus polynesiae* (Schedl, 1979e: 105) (*Hypocryphalus*).

• *Cryphalus procerus* Schedl, 1953f: 296.

• *Cryphalus pruni* Eggers, 1929c: 11.

• *Cryphalus pseudochinlingensis* Tsai & Li, 1963: 610.

• *Cryphalus pseudotabulaeformis* Tsai & Li, 1963: 613.

• *Cryphalus puberulus* Schedl, 1942c: 171.

• *Cryphalus pubescens* Hopkins, 1915d: 40.

= *Cryphalus subconcentralis* Hopkins, 1915d: 40.

• *Cryphalus pulchellus* (Nobuchi, 1975: 54) (*Taenioglyptes*).

• *Cryphalus punctatostriatus* Schedl, 1942b: 175.

• *Cryphalus punctatus* Browne, 1983c: 66.

• *Cryphalus punctipennis* Schedl, 1942c: 169.

• *Cryphalus punctistriatulus* Johnson in: Johnson et al. 2020a: 23 (RN).

= *Cryphalus striatulus* Browne, 1981a: 127.

• *Cryphalus pusillimus* Schedl, 1942c: 171.

• *Cryphalus pusillus* Schedl, 1943: 38.

• *Cryphalus quadrituberculatus* (Schedl, 1963d: 64) (*Margadillius*).

• *Cryphalus redikorzevi* Berger, 1917: 232.

• *Cryphalus reflexus* (Browne, 1980b: 383) (*Hypocryphalus*).

• *Cryphalus resiniferi* Schedl, 1943: 36.

• *Cryphalus rhusii* Niisima, 1909: 145.

= *Cryphalus kurilensis* Krivolutskaya, 1968: 53.

• *Cryphalus robustus* Eichhoff, 1872a: 131.

• *Cryphalus rotundus* (Hopkins, 1915d: 41) (*Hypocryphalus*).

• *Cryphalus rubentis* Hopkins, 1915d: 40.

• *Cryphalus rufopilosus* Schedl, 1942b: 174.

• *Cryphalus rugosus* (Schedl, 1958e: 102) (*Ericryphalus*).

• *Cryphalus saltuarius* Weise in: Heyden et al. 1891: 336.

= *Bostrichus asperatus* Gyllenhal, 1813: 383.

= *Cryphalus scriba* Gozis, 1886: 31 (RN).

• *Cryphalus samoensis* Beeson, 1929: 224.

• *Cryphalus sandakanensis* Schedl, 1937f: 548.

= *Hypocryphalus maculatus* Browne, 1962b: 303.

• *Cryphalus sarawakensis* Schedl, 1972d: 199.

• *Cryphalus sawadai* Nobuchi & Takahashi, 1965: 3.

• *Cryphalus scabripennis* Schedl, 1972d: 199.

• *Cryphalus schedli* Johnson in: Johnson et al. 2020a: 23 (RN).

= *Hypocryphalus formosanus* Schedl, 1952h: 62.

• *Cryphalus scopiger* Berger, 1917: 228.

• *Cryphalus securus* (Schedl, 1940b: 436) (*Ericryphalus*).

• *Cryphalus sejugatus* Schedl, 1965f: 54.

• *Cryphalus sichotensis* Kurentsov, 1941: 152.

• *Cryphalus sidneyanus* (Nördlinger, 1856: 75) (*Bostrichus*).

• *Cryphalus silvanus* Schedl, 1951f: 145.

• *Cryphalus similis* (Eggers, 1922c: 168) (*Stephanoderes*).

• *Cryphalus simplex* (Schedl, 1939b: 341) (*Ericryphalus*).

• *Cryphalus sinoabietis* Tsai & Li, 1963: 606.

= *Cryphalus sinoabietis opiensis* Tsai & Li, 1963: 607.

• *Cryphalus solomonensis* Johnson in: Johnson et al. 2020a: 24 (RN).

= *Margadillius terminaliae* Browne, 1984c: 450.

• *Cryphalus sordidus* (Nobuchi, 1975: 51) (*Taenioglyptes*).

• *Cryphalus sparsepilosus* Schedl, 1942c: 172.

• *Cryphalus spathulatus* (Schedl, 1938g: 49) (*Hypocryphalus*).

• *Cryphalus spissepilosus* Johnson in: Johnson et al. 2020a: 24 (RN).

= *Cryphalus densepilosus* Schedl, 1943: 36.

• *Cryphalus squameus* Schedl, 1943: 38.

• *Cryphalus squamulosus* Strohmeyer, 1911b: 20.

• *Cryphalus storckiellae* Johnson in: Johnson et al. 2020a: 24 (RN).

= *Cryphalus striatus* Browne, 1974a: 66.

• *Cryphalus striatulus* Mannerheim, 1853: 235.

= *Cryphalus approximatus* Hopkins, 1915d: 42.

= *Cryphalus balsameus* Hopkins, 1915d: 41.

= *Cryphalus fraseri* Hopkins, 1915d: 40.

= *Cryphalus ruficollis* Hopkins, 1915d: 40.

= *Cryphalus ruficollis ruficollis* Hopkins, 1915d: 40.

= *Cryphalus amabilis* Chamberlin, 1917: 321.

= *Cryphalus grandis* Chamberlin, 1917: 323.

= *Cryphalus canadensis* Chamberlin, in: Swaine 1918: 88.

= *Cryphalus mainensis* Blackman, 1922b: 126.

= *Taenioglyptes ruficollis coloradensis* Wood, 1954b: 1008.

• *Cryphalus striatus* (Hopkins, 1915d: 42) (*Hypocryphalus*).

• *Cryphalus strigilatus* Wichmann, 1914a: 137.

• *Cryphalus strigipennis* Schedl, 1950e: 50.

• *Cryphalus strohmeyeri* Stebbing, 1914: 538 (RN).

= *Cryphalus indicus* Stebbing, 1906: 403.

• *Cryphalus subcompactus* Lea, 1910: 140.

• *Cryphalus subgranulatus* Schedl, 1943: 37.

• *Cryphalus submuricatus* Eichhoff, 1878a: 385.

= *Cryphalus submuricatus* Eichhoff, 1878b: 492.

= *Cryphalus tuberculatus* Schedl, 1943: 37.

• *Cryphalus substriatus* Schedl, 1942b: 174.

• *Cryphalus subtuberculatus* Schedl, 1942c: 168.

• *Cryphalus subvestitus* Schedl, 1959i: 482.

• *Cryphalus sumatranus* (Schedl, 1939j: 38) (*Dacryphalus*).

• *Cryphalus sundaensis* Schedl, 1942d: 14.

• *Cryphalus swezeyi* Schedl, 1942a: 147.

• *Cryphalus sylvicola* (Perkins, 1900: 181) (*Hypothenemus*).

= *Ericryphalus henshawi* Hopkins, 1915d: 38.

= *Cryphalus dimorphus* Schedl, 1950e: 38.

= *Cryphalus sylvicola obliquus* Schedl, 1950e: 48.

• *Cryphalus szechuanensis* Tsai & Li, 1963: 614.

= *Cryphalus szechuanensis tehchangensis* Tsai & Li, 1963: 615.

• *Cryphalus tabulaeformis* Tsai & Li, 1963: 609.

= *Cryphalus tabulaeformis chienzhuangensis* Tsai & Li, 1963: 610.

• *Cryphalus taiwanus* Schedl, 1970f: 359.

• *Cryphalus takahashii* Johnson in: Johnson et al. 2020a: 24 (RN).

= *Euptilius exiguus* Browne, 1984c: 451.

• *Cryphalus tenuis* Schedl, 1942d: 16.

• *Cryphalus terminaliae* Browne, 1980b: 384.

• *Cryphalus tetricus* (Schedl, 1940b: 437) (*Ericryphalus*).

= *Cryphalus papuanus* Schedl, 1942c: 170.

= *Cryphalus grayi* Schedl, 1968c: 265.

= *Ptilopodius brevis* Browne, 1970: 556.

• *Cryphalus theobromae* Nunberg, 1956c: 208 (RN).

= *Cryphalus horridus* Graham, 1908: 113.

• *Cryphalus triangularis* (Schedl, 1975a: 351) (*Hypocryphalus*).

• *Cryphalus trypanoides* Beeson, 1935a: 106.

= *Cryphalus mollis* Schedl, 1955e: 288.

= *Hypocryphalus tongaensis* Schedl, 1979e: 104.

• *Cryphalus trypanus* Sampson, 1914: 383.

• *Cryphalus tutuilaensis* (Schedl, 1951f: 147) (*Hypocryphalus*).

• *Cryphalus uapouensis* (Beeson, 1935a: 107) (*Ericryphalus*).

• *Cryphalus upoluensis* Schedl, 1951f: 144.

• *Cryphalus variolosus* Schedl, 1950e: 49.

• *Cryphalus vestitus* Blandford, 1895: 318.

• *Cryphalus viburni* Stark, 1936: 143.

= *Cryphalus viburni* Eggers, 1942a: 30.

• *Cryphalus vitiensis* Browne, 1974a: 67.

• *Cryphalus walkeri* (Blandford, 1896e: 200) (*Cryptarthrum*).

= *Coccotrypes hagedorni* Eggers, 1908a: 216.

= *Cryphalus javanus* Schedl, 1954c: 148.

• *Cryphalus wapleri* Eichhoff, 1872a: 131.

= *Cryphalus mekeoi* Schedl, 1972k: 61.

• *Cryphalus yamaguchii* Inouye & Nobuchi, 1957: 55.

• *Cryphalus zimmermani* Schedl, 1950e: 51.

### CRYPTURGINI LeConte

5 genera, 50 spp. (5 spp. with 12 sspp.).

#### *Aphanarthrum* Wollaston

25 spp. (5 spp. with 12 sspp.).

• *Aphanarthrum aeonii* Lindberg, 1953: 18.

• *Aphanarthrum affine* Wollaston, 1860a: 166.

• *Aphanarthrum alluaudi* Peyerimhoff, 1923: 52.

= *Coleobothrus jandiacus* Enderlein, 1929: 144.

• *Aphanarthrum armatum* Wollaston, 1862b: 167.

= *Aphanarthrum monodi* Paulian & Villiers, 1941: 102.

• *Aphanarthrum bicinctum bicinctum* Wollaston, 1860a: 165 (*Aphanarthrum bicinctum*).

• *Aphanarthrum bicinctum obsitum* Wollaston, 1865: 43.

• *Aphanarthrum bicinctum vestitum* Wollaston, 1865: 43.

• *Aphanarthrum bicolor* Wollaston, 1860a: 165.

• *Aphanarthrum canariense canariense* Wollaston, 1860a: 164 (*Aphanarthrum canariense*).

• *Aphanarthrum canariense neglectum* Schedl, 1964c: 99 (*Aphanarthrum neglec­tum*).

= *Aphanarthrum canariense longipes* Israelson, 1972: 256.

• *Aphanarthrum canescens canescens* Wollaston, 1865: 41 (*Aphanarthrum canescens*).

• *Aphanarthrum canescens polyspiniger* Israelson, 1972: 256.

• *Aphanarthrum canescens simplex* Wollaston, 1865: 41 (*Aphanarthrum simplex*).

• *Aphanarthrum capense* Jordal, 2009a: 55.

• *Aphanarthrum euphorbiae* Wollaston, 1854: 293.

• *Aphanarthrum germeauxi* (Menier, 1973: 207) (*Coleobothrus*).

• *Aphanarthrum glabrum glabrum* Wollaston, 1860a: 167 (*Aphanarthrum glabrum*).

• *Aphanarthrum glabrum nudum* Israelson, 1972: 251.

• *Aphanarthrum hesperidum* Wollaston, 1868: 117.

• *Aphanarthrum indicum* Wood, 1988b: 190.

• *Aphanarthrum jubae jubae* Wollaston, 1860a: 164 (*Aphanarthrum jubae*).

• *Aphanarthrum jubae tuberculatum* Wollaston, 1865: 40 (*Aphanarthrum tuberculatum*).

• *Aphanarthrum luridum* Wollaston, 1860a: 163.

= *Coleobothrus luridus annulicollis* Enderlein, 1929: 146.

= *Coleobothrus luridus flavicollis* Enderlein, 1929: 146.

• *Aphanarthrum maculatum* Jordal, 2009a: 57.

• *Aphanarthrum mairei* Peyerimhoff, 1923: 53.

= *Aphanarthrum saturatum* Peyerimhoff, 1925: 12.

= *Aphanarthrum goniomma* Enderlein, 1929: 142.

= *Aphanarthrum duongi* Villiers, 1946: 140.

• *Aphanarthrum orientale* Schedl, 1971d: 12 (E).

• *Aphanarthrum piscatorium* Wollaston, 1860a: 166.

• *Aphanarthrum pygmaeum* Wollaston, 1865: 42.

= *Aphanarthrum pygmaeum laticollis* Wollaston, 1865: 42.

• *Aphanarthrum reticulatum* Wood, 1988b: 191.

• *Aphanarthrum royaleanum* Wood, 1988b: 191.

• *Aphanarthrum subglabrum* Israelson, 1972: 251.

• *Aphanarthrum wollastoni* Israelson, 1972: 251.

#### *Cisurgus* Reitter

7 spp.

• *Cisurgus ferulae* Pfeffer, 1983: 294.

• *Cisurgus filum* (Reitter, 1889c: 126) (*Crypturgus*).

• *Cisurgus hystrix* (Abeille de Perrin, 1894: 94) (*Crypturgus*).

= *Cisurgus ragusae* Reitter, 1906a: 241.

• *Cisurgus maurus* Eggers, 1910a: 559.

= *Cisurgus karamani* Reitter, 1913: 64.

• *Cisurgus occidentalis* Peyerimhoff, 1923: 53.

= *Cisurgus occidentalis resiniferae* Peyerimhoff, 1925: 13.

• *Cisurgus pusillus* (Wollaston, 1860a: 167) (*Aphanarthrum*).

= *Crypturgus wollastonii* Eichhoff, 1878b: 77 (RN).

• *Cisurgus seselii* Coffin & Téocchi, 1991: 157.

#### *Crypturgus* Erichson

16 spp.

• *Crypturgus alutaceus* Schwarz, 1894a: 17.

• *Crypturgus beesoni* Eggers, 1936a: 627.

• *Crypturgus borealis* Swaine, 1917: 7.

= *Crypturgus corrugatus* Swaine, 1917: 8.

• *Crypturgus cedri* Eichhoff, 1868a: 403.

• *Crypturgus cinereus* (Herbst, 1793: 116) (*Bostrichus*).

= *Hylesinus tenerrimus* Sahlberg, 1836b: 140.

= *Crypturgus atticus* Eggers, 1911b: 120.

= *Crypturgus corsicus* Eggers, 1923a: 135.

= *Crypturgus apfelbecki* Eggers, 1940g: 36.

• *Crypturgus concolor* (Wollaston, 1864: 263) (*Aphanarthrum*).

• *Crypturgus cribrellus* Reitter, 1895: 64.

• *Crypturgus cylindricollis* Eggers, 1940g: 37.

• *Crypturgus dubius* Eichhoff, 1872b: 139.

• *Crypturgus hispidulus* Thomson, 1870: 338.

= *Crypturgus maulei* Roubal, 1910: 203.

• *Crypturgus mediterraneus* Eichhoff in: Puton 1869: 41.

= *Crypturgus mediterraneus* Eichhoff, 1872b: 139.

• *Crypturgus numidicus* Ferrari, 1867: 6.

= *Crypturgus numidicus abbreviatus* Eggers, 1911b: 123.

= *Crypturgus brevipennis* Reitter, 1913: 63.

= *Crypturgus barbeyi* Strohmeyer, 1929: 182.

• *Crypturgus parallelocollis* Eichhoff, 1878b: 73 (*Crypturgus pusillus parallelocollis*).

= *Crypturgus gaunersdorferi* Reitter, 1885: 389.

• *Crypturgus pusillus* (Gyllenhal, 1813: 371) (*Bostrichus*).

= *Bostrichus aphodiodes* Villa & Villa, 1833: 36.

= *Crypturgus atomus* LeConte, 1868: 152.

= *Polygraphus minimus* Stebbing, 1903: 252.

= *Crypturgus danicus* Eggers, 1932a: 80.

• *Crypturgus subcribrosus* Eggers, 1933a: 5.

• *Crypturgus tuberosus* Niisima, 1909: 139.

#### *Deropria* Enderlein

1 sp.

• *Deropria elongata* (Eggers, 1927d: 39) (*Aphanarthrum elongatum*).

#### *Dolurgus* Eichhoff

1 sp.

• *Dolurgus pumilus* (Mannerheim, 1843: 297) (*Hylastes*).

### DIAMERINI Hagedorn

7 genera, 136 spp.

#### *Acacicis* Lea

14 spp.

• *Acacicis aphananthe* Lin & Beaver, 2021: 1.

• *Acacicis atomarius* (Chapuis, 1869: 29) (*Hylesinus*).

= *Acacicis abundans* Lea, 1910: 149.

• *Acacicis bicornis* Wood, 1988a: 34.

• *Acacicis borneensis* Browne, 1962a: 203.

• *Acacicis carphoboroides* (Nunberg, 1960: 287) (*Trogloditica*).

• *Acacicis globosus* (Eggers, 1935e: 298) (*Trogloditica globosa*).

• *Acacicis granulicollis* (Schedl, 1971b: 282) (*Neodiamerus*).

• *Acacicis kivuensis* (Nunberg, 1967: 314) (*Trogloditica*).

• *Acacicis malayanus* Browne, 1962a: 202.

• *Acacicis minor* Schedl, 1936e: 525.

• *Acacicis squamosus* (Eggers, 1933e: 20) (*Trogloditica squamosa*).

• *Acacicis syrutscheki* (Wichmann, 1913c: 143) (*Dendrosinus*).

• *Acacicis trahax* (Sampson, 1922b: 148) (*Trogloditica*).

• *Acacicis zeylanicus* Wood, 1988a: 34.

#### *Bothrosternoides* Schedl

1 sp.

• *Bothrosternoides malayensis* Schedl, 1969a: 211.

#### *Diamerus* Erichson

34 spp.

• *Diamerus ater* Hagedorn, 1909: 735.

• *Diamerus batoensis* Eggers, 1927e: 392.

• *Diamerus brevicollis* Eggers, 1923b: 131.

• *Diamerus caesius* Hagedorn, 1909: 735.

• *Diamerus cherenus* Eggers, 1920a: 230.

• *Diamerus cinerascens* Fairmaire, 1897: 195.

• *Diamerus curvifer* (Walker, 1859: 261) (*Hylesinus*).

= *Acanthurus spinipennis* Eichhoff, 1886: 24.

= *Diamerus dissimilis* Hagedorn, 1909: 735.

• *Diamerus eggersi* Schedl, 1937e: 397.

• *Diamerus fici* Blandford, 1898b: 426.

• *Diamerus granifer* Browne, 1984e: 87.

• *Diamerus granulatus* Eggers, 1923b: 131.

• *Diamerus griseopubescens* Schedl, 1951i: 52.

• *Diamerus hispidus* (Klug, 1833: 202) (*Hylesinus*).

• *Diamerus impar* Chapuis, 1869: 50.

= *Diamerus impar nanus* Hagedorn, 1909: 734.

• *Diamerus imperfectus* Eggers, 1940e: 227.

• *Diamerus inermis* Eggers, 1940e: 228.

• *Diamerus interstitialis* (Lea, 1910: 145) (*Hylesinus*).

= *Diamerus subsulcatus* Eggers, 1923b: 130.

• *Diamerus lignivorus* Browne, 1955: 343.

• *Diamerus luteus* Hagedorn, 1909: 735.

• *Diamerus mangiferae* Browne, 1955: 344.

• *Diamerus matangi* Sampson, 1919: 108.

• *Diamerus merinjaki* Sampson, 1919: 107.

• *Diamerus minor* Eggers, 1936e: 79.

• *Diamerus nigrisetosus* Eggers, 1936e: 79.

• *Diamerus obtusus* Eggers, 1936e: 78.

• *Diamerus opacus* Eggers, 1923b: 132.

• *Diamerus parvus* Eggers, 1943c: 71.

• *Diamerus pimelioides* (Schaufuss, 1905e: 71) (*Lissoclastus*).

= *Diamerus tuberculatus* Hagedorn, 1909: 734.

• *Diamerus propinquus* Nunberg, 1969: 399.

• *Diamerus pulverulentus* Gerstäcker, 1873: 249.

• *Diamerus puncticollis* Eggers, 1927b: 68.

• *Diamerus ritsemae* (Eichhoff, 1886: 25) (*Acanthurus*).

= *Diamerus nigrescens* Eggers, 1936e: 77.

• *Diamerus striatus* Eggers, 1927b: 67.

• *Diamerus variegatus* Schedl, 1959f: 41.

#### *Peronophorus* Strohmeyer

5 spp.

• *Peronophorus abhorrens* Eggers, 1943c: 71.

• *Peronophorus armatus* Eggers, 1933e: 21.

• *Peronophorus brevicollis* (Strohmeyer, 1910a: 70) (*Acanthophorus*).

• *Peronophorus obscurus* Eggers, 1940e: 230.

• *Peronophorus pondoanus* Eggers, 1933e: 22.

#### *Pseudodiamerus* Eggers

3 spp.

• *Pseudodiamerus aspericollis* Eggers, 1936c: 33.

• *Pseudodiamerus obscurus* Eggers, 1943c: 72.

= *Phloeoditica obscura* Schedl, 1962f: 189.

= *Phloeoditica obscura* Schedl, 1963b: 261.

• *Pseudodiamerus striatus* Eggers, 1933e: 18.

#### *Sphaerotrypes* Blandford

48 spp.

• *Sphaerotrypes bangensis* Eggers, 1927b: 73.

• *Sphaerotrypes barbatus* Hagedorn, 1909: 739.

• *Sphaerotrypes bengalensis* Wood, 1988a: 35.

• *Sphaerotrypes bicolor* Eggers, 1927b: 70.

• *Sphaerotrypes biseriatus* Schedl, 1964i: 243.

• *Sphaerotrypes blandfordi* Schaufuss, 1897a: 102.

• *Sphaerotrypes boettcheri* Eggers, 1927b: 72.

• *Sphaerotrypes brevisetosus* Browne in: Beaver and Browne 1979: 599.

• *Sphaerotrypes brunneus* Heymons, 1920: 98.

• *Sphaerotrypes bryanti* Browne, 1962b: 300.

• *Sphaerotrypes carinatus* Eggers, 1927b: 71.

• *Sphaerotrypes carpini* Eggers, 1926a: 134.

• *Sphaerotrypes coimbatorensis* Stebbing, 1906: 395.

• *Sphaerotrypes congonus* Eggers, 1932b: 27.

• *Sphaerotrypes costatus* Wood, 1988a: 35.

• *Sphaerotrypes cristatus* Wood, 1988a: 36.

• *Sphaerotrypes expressus* Schedl, 1937e: 398.

• *Sphaerotrypes globulus* Blandford, 1894c: 63.

• *Sphaerotrypes grandis* Schedl, 1957b: 31.

• *Sphaerotrypes hagedorni* Eggers, 1920b: 122.

• *Sphaerotrypes helferi* Eggers, 1925: 152.

• *Sphaerotrypes inermis* Browne, 1981a: 127.

• *Sphaerotrypes insularis* Eggers, 1927b: 74.

• *Sphaerotrypes juglansi* Tsai & Yin, 1966: 236.

• *Sphaerotrypes juglansis* Krivolutskaya, 1970: 210.

• *Sphaerotrypes limbatus* Eggers, 1943a: 244.

• *Sphaerotrypes magnus* Tsai & Yin, 1966: 234.

• *Sphaerotrypes minutus* Browne, 1962b: 302.

• *Sphaerotrypes montanus* Buhroo & Knížek, 2020: 145.

• *Sphaerotrypes moseri* Eggers, 1927b: 73.

• *Sphaerotrypes palawanus* Eggers, 1927b: 74.

• *Sphaerotrypes pentacme* Wood, 1988a: 36.

• *Sphaerotrypes philippinensis* Strohmeyer, 1911b: 18.

• *Sphaerotrypes pila* Blandford, 1894c: 62.

= *Sphaerotrypes imitans* Eggers, 1926a: 134.

• *Sphaerotrypes pygeumi* Schedl, 1955b: 214.

• *Sphaerotrypes pyri* Tsai & Yin, 1966: 240.

• *Sphaerotrypes quadrituberculatus* Sampson, 1922b: 150.

• *Sphaerotrypes querci* Stebbing, 1908: 5.

= *Chramesus globulus* Stebbing, 1909: 21.

= *Sphaerotrypes tectus* Beeson, 1921: 514.

• *Sphaerotrypes ranasinghei* Wood, 1988a: 37.

• *Sphaerotrypes rufopalliatus* Schedl, 1939b: 338.

• *Sphaerotrypes siwalikensis* Stebbing, 1906: 389.

= *Sphaerotrypes assamensis* Stebbing, 1907: 23.

• *Sphaerotrypes subtectus* Browne, 1970: 545.

• *Sphaerotrypes sulcatus* Browne, 1986a: 90.

• *Sphaerotrypes tanganus* Schaufuss, 1897a: 101.

• *Sphaerotrypes tsugae* Tsai & Yin, 1966: 240.

• *Sphaerotrypes ulmi* Tsai & Yin, 1966: 240.

• *Sphaerotrypes variegatus* Eggers, 1932b: 26.

• *Sphaerotrypes yunnanensis* Tsai & Yin, 1966: 240.

#### *Strombophorus* Hagedorn

31 spp.

• *Strombophorus camerunus* Hagedorn, 1909: 742.

• *Strombophorus capensis* Schedl, 1965d: 113.

• *Strombophorus celtis* Schedl, 1955b: 214.

• *Strombophorus cordatus* Hagedorn, 1909: 741.

• *Strombophorus crenatus* Hagedorn, 1909: 740.

= *Strombophorus nudus* Nunberg, 1961b: 332.

• *Strombophorus dialiumi* Schedl, 1957b: 20.

• *Strombophorus elongatus* Eggers, 1920a: 231.

• *Strombophorus ericius* (Schaufuss, 1897b: 217) (*Diamerus*).

= *Strombophorus variegatus* Eggers, 1940e: 231.

• *Strombophorus flavipubens* Schedl, 1959a: 706.

• *Strombophorus granulifer* Eggers, 1932b: 23.

• *Strombophorus hispidus* Eggers, 1932b: 26.

• *Strombophorus intermedius* Eggers, 1920b: 117.

• *Strombophorus interstitialis* Browne, 1963a: 241.

• *Strombophorus kaszabi* Schedl, 1967c: 223.

• *Strombophorus laevis* Eggers, 1920a: 232.

• *Strombophorus latus* Eggers, 1920a: 233.

• *Strombophorus lukengeae* Schedl, 1957b: 20.

• *Strombophorus major* Schedl, 1941c: 385.

• *Strombophorus millettiae* Schedl, 1957b: 21.

• *Strombophorus minor* Eggers, 1920a: 233.

• *Strombophorus minutissimus* Schedl, 1936f: 136.

• *Strombophorus movoliae* Schedl, 1957b: 22.

• *Strombophorus nigrescens* Schedl, 1941c: 387.

• *Strombophorus occidentalis* Schedl, 1941c: 385.

• *Strombophorus pilifer* (Eggers, 1927c: 172) (*Hylesinus*).

• *Strombophorus pusillus* Eggers, 1932b: 24.

= *Strombophorus spinosus* Eggers, 1932b: 25.

• *Strombophorus setulosus* Schedl, 1941c: 386.

• *Strombophorus spathulatus* Schedl, 1941c: 386.

• *Strombophorus testudo* Eggers, 1944c: 92.

• *Strombophorus vagans* Schedl, 1957b: 23.

• *Strombophorus vittatus* Eggers, 1935e: 297.

= *Strombophorus pseudomoviliae* Nunberg, 1961b: 336.

### DRYOCOETINI Lindemann

21 genera, 485 spp. (2 spp. with 4 sspp.).

#### *Chiloxylon* Schedl

1 sp.

• *Chiloxylon rufulum* Schedl, 1959b: 550 (E).

#### *Coccotrypes* Eichhoff

129 spp.

• *Coccotrypes abruptulus* (Schedl, 1975e: 347) (*Poecilips*).

• *Coccotrypes abruptus* (Schedl, 1979e: 99) (*Poecilips*).

• *Coccotrypes aciculatus* Schedl, 1952e: 360.

• *Coccotrypes advena* Blandford, 1894c: 100.

= *Thamnurgides persicae* Hopkins, 1915d: 45.

= *Dendrurgus minor* Eggers, 1923b: 150.

= *Dendrurgus philippinensis* Eggers, 1923b: 145.

= *Dendrurgus ternatensis* Eggers, 1923b: 146.

= *Thamnurgides setosus* Beeson, 1929: 228.

= *Coccotrypes philippinensis* Schedl, 1933b: 104.

= *Thamnurgides cubanus* Eggers, 1934a: 79.

= *Poecilips nuciferus* Schedl, 1938c: 10.

= *Poecilips niger* Schedl, 1939b: 345.

= *Thamnurgides vicarius* Beeson, 1939: 285.

= *Poecilips subnitidus* Schedl, 1954c: 147.

• *Coccotrypes amplipunctatus* Bright, 2000: 55.

• *Coccotrypes asper* (Schedl, 1961a: 351) (*Poecilips*).

• *Coccotrypes aspericollis* (Beeson, 1939: 295) (*Thamnurgides*).

• *Coccotrypes babai* (Murayama, 1961: 29) (*Thamnurgus*).

• *Coccotrypes barbatus* (Schedl, 1934a: 90) (*Thamnurgides*).

= *Thamnurgides ater* Eggers, 1936e: 84.

= *Thamnurgides bambusae* Beeson, 1939: 289.

= *Thamnurgides dipterocarpi* Beeson, 1939: 288.

• *Coccotrypes bicolor* Sampson, 1914: 390.

• *Coccotrypes birmanus* Eggers, 1939b: 8.

• *Coccotrypes borneensis* (Eggers, 1923b: 146) (*Dendrurgus*).

• *Coccotrypes brunneus* (Nunberg, 1961a: 616) (*Poecilips*).

• *Coccotrypes brunnipes* Wood, 1989: 178 (RN).

= *Coccotrypes brunneus* Nunberg, 1973: 23.

• *Coccotrypes campnospermae* (Browne in: Beaver and Browne 1979: 593) (*Poecilips*).

• *Coccotrypes cardamomi* Schaufuss, 1905a: 8.

• *Coccotrypes carinensis* (Eggers, 1923b: 147) (*Dendrurgus*).

• *Coccotrypes carpophagus* (Hornung, 1842: 116) (*Bostrichus*).

= *Coccotrypes integer* Eichhoff, 1878b: 311.

= *Coccotrypes integer* Eichhoff, 1878a: 391.

= *Coccotrypes pygmaeus* Eichhoff, 1878b: 310.

= *Coccotrypes pygmaeus* Eichhoff, 1878a: 391.

= *Cryphaloides donisthorpei* Formánek, 1908: 91.

= *Coccotrypes anonae* Hopkins, 1915d: 46.

= *Coccotrypes bakeri* Hopkins, 1915d: 46.

= *Coccotrypes hubbardi* Hopkins, 1915d: 46.

= *Coccotrypes liberiensis* Hopkins, 1915d: 47.

= *Coccotrypes rolliniae* Hopkins, 1915d: 47.

= *Coccotrypes thrinacis* Hopkins, 1915d: 46.

= *Coccotrypes nanus* Eggers, 1920c: 33.

= *Coccotrypes canariensis* Eggers, 1928c: 117.

= *Coccotrypes phoenicola* Beeson, 1939: 281.

= *Coccotrypes trevori* Beeson, 1939: 282.

= *Coccotrypes ceylonicus* Schedl, 1948e: 119.

= *Coccotrypes pilosulus* Schedl, 1948e: 118.

= *Coccotrypes pubescens* Schedl, 1948e: 119.

= *Coccotrypes punctulatus* Eggers, 1951: 151.

= *Coccotrypes grisseopuberulus* Schedl, 1972f: 59.

= *Coccotrypes exasperatus* Schedl, 1975j: 455.

• *Coccotrypes chimbui* (Schedl, 1975a: 354) (*Poecilips*).

• *Coccotrypes cinnamomi* (Eggers, 1936a: 630) (*Thamnurgides*).

• *Coccotrypes circumdatus* Fonseca, 1930: 88.

• *Coccotrypes collaris* (Schedl, 1965f: 59) (*Poecilips*).

• *Coccotrypes confertus* (Schedl, 1942d: 24) (*Poecilips*).

• *Coccotrypes congonus* Eggers, 1924: 104.

• *Coccotrypes corpulentus* Browne, 1970: 565.

• *Coccotrypes crassiventris* (Schedl, 1961a: 351) (*Poecilips*).

• *Coccotrypes creber* (Schedl, 1955e: 290) (*Poecilips*).

• *Coccotrypes cylindricus* (Eggers, 1927c: 187) (*Poecilips*).

• *Coccotrypes cyperi* (Beeson, 1929: 230) (*Thamnurgides*).

= *Xyleborus conspiciens* Schedl, 1936a: 110.

= *Thamnurgides indicus* Eggers, 1936a: 631.

= *Dryocoetes insularis* Eggers, 1940a: 127.

= *Coccotrypes insularis* Eggers, 1940a: 129.

= *Dryocoetes subimpresus* Eggers, 1940a: 127.

= *Poecilips subaplanatus* Schedl, 1942d: 23.

= *Poecilips caraibicus* Schedl, 1952e: 345 (RN).

= *Poecilips eggersi* Schedl, 1952e: 347 (RN).

= *Poecilips pilifrons* Browne, 1970: 568.

• *Coccotrypes dactyliperda* (Fabricius, 1801: 387) (*Bostrichus*).

= *Bostrichus palmicola* Hornung, 1842: 116.

= *Coccotrypes tropicus* Eichhoff, 1878b: 312.

= *Coccotrypes tropicus* Eichhoff, 1878a: 391.

= *Coccotrypes laboulbenei* Decaux, 1890: 953.

= *Coccotrypes eggersi* Hagedorn, 1904a: 449.

= *Coccotrypes bassiaevorus* Hopkins, 1915d: 47.

= *Coccotrypes moreirai* Eggers, 1928a: 86.

= *Coccotrypes tanganus* Eggers, 1935e: 307.

= *Coccotrypes borassi* Beeson, 1939: 283.

= *Coccotrypes elaeocarpi* Beeson, 1939: 284.

• *Coccotrypes declivis* Sampson, 1914: 390.

• *Coccotrypes depressus* (Eggers, 1927b: 82) (*Thamnurgides*).

• *Coccotrypes distinctus* (Motschulsky, 1866: 401) (*Anodius*).

= *Coccotrypes floridensis* Schedl, 1948e: 117.

• *Coccotrypes duplopilosus* (Browne, 1970: 567) (*Thamnurgides*).

= *Thamnurgides uniseriatus* Eggers, 1936e: 85.

• *Coccotrypes elongatulus* (Schedl, 1975j: 455) (*Poecilips*).

• *Coccotrypes elongatus* (Eggers, 1923b: 150) (*Dendrurgus*).

• *Coccotrypes excavatus* Schedl, 1948e: 114.

• *Coccotrypes fagi* (Nobuchi, 1959a: 12) (*Poecilips*).

• *Coccotrypes fallax* (Eggers, 1927e: 399) (*Poecilips*).

• *Coccotrypes flavicornis* (Blandford, 1895: 320) (*Dryocoetes*).

• *Coccotrypes fulgens* (Schedl, 1979e: 99) (*Poecilips*).

• *Coccotrypes furvus* (Sampson, 1914: 389) (*Dryocoetes*).

• *Coccotrypes gedeanus* (Eggers, 1936e: 86) (*Thamnurgides*).

= *Xyleborus grossopunctatus* Schedl, 1942d: 36.

= *Ozopemon sulcipennis* Schedl, 1942b: 178.

= *Xyleborus despectus* Schedl, 1975i: 294.

• *Coccotrypes ghesquierei* Eggers, 1927c: 179.

= *Poecilips imitans* Eggers, 1932c: 293.

• *Coccotrypes grandis* (Eggers, 1927c: 188) (*Poecilips*).

• *Coccotrypes graniceps* (Eichhoff, 1877a: 120) (*Dryocoetes*).

= *Coccotrypes graniceps* Eichhoff, 1878b: 314.

• *Coccotrypes impressostriatus* Bright, 2000: 58.

• *Coccotrypes impressus* Eggers, 1936a: 632.

• *Coccotrypes incertus* Bright, 2019: 236.

• *Coccotrypes incognitus* (Schedl, 1962e: 89) (*Poecilips*).

• *Coccotrypes jacobsoni* (Eggers, 1923b: 149) (*Dendrurgus*).

• *Coccotrypes japonicus* (Eggers, 1926b: 145) (*Poecilips*).

• *Coccotrypes klapperichi* (Schedl, 1953a: 26) (*Poecilips*).

• *Coccotrypes laevicollis* (Schedl, 1957b: 67) (*Poecilips*).

• *Coccotrypes laticollis* (Browne, 1980e: 775) (*Poecilips*).

• *Coccotrypes leveri* Browne, 1970: 564.

• *Coccotrypes limbatideclivis* Bright, 2000: 60.

• *Coccotrypes litoralis* (Beeson, 1939: 291) (*Thamnurgides*).

• *Coccotrypes longicollis* (Eggers, 1927e: 397) (*Thamnurgides*).

• *Coccotrypes longior* (Eggers, 1927b: 83) (*Poecilips*).

= *Poecilips oblongus* Eggers, 1927b: 83.

= *Poecilips linearis* Eggers, 1941c: 222.

= *Poecilips nitidipennis* Schedl, 1950a: 896.

= *Dryocoetes naidaijinensis* Murayama, 1957b: 605.

= *Poecilips apicatus* Schedl, 1971e: 372.

• *Coccotrypes luzonicus* (Schedl, 1943: 41) (*Dryocoetes*).

• *Coccotrypes magnus* Beeson, 1939: 283.

• *Coccotrypes malgasicus* (Schedl, 1961g: 136) (*Poecilips*).

• *Coccotrypes marginatus* (Browne in: Beaver and Browne 1979: 595) (*Poecilips*).

• *Coccotrypes medius* (Eggers, 1927b: 84) (*Poecilips*).

• *Coccotrypes minimus* (Schedl, 1955e: 291) (*Poecilips*).

• *Coccotrypes minutissimus* (Schedl, 1955e: 292) (*Poecilips*).

• *Coccotrypes minutus* Schedl, 1975e: 346.

• *Coccotrypes mjoebergi* (Schedl, 1971e: 373) (*Poecilips*).

• *Coccotrypes monoceros* (Beeson, 1939: 291) (*Thamnurgides*).

• *Coccotrypes morokensis* (Eggers, 1923b: 151) (*Dendrurgus*).

• *Coccotrypes myristicae* (Roepke, 1919: 23) (*Thamnurgides*).

= *Dendrurgus sundaensis* Eggers, 1923b: 145.

= *Thamnurgides calapanus* Eggers, 1927b: 81.

= *Thamnurgides curtus* Eggers, 1927b: 80.

= *Thamnurgides masoni* Beeson, 1939: 292.

• *Coccotrypes niger* Eggers, 1927c: 180.

• *Coccotrypes nigripes* Eggers, 1924: 105.

• *Coccotrypes nigronitens* (Schedl, 1975g: 455) (*Poecilips*).

• *Coccotrypes nitidus* (Eggers, 1923b: 147) (*Dendrurgus*).

= *Thamnurgides insularis* Eggers, 1939c: 223.

= *Poecilips aterrimus* Schedl, 1953f: 298.

• *Coccotrypes norimasanus* (Murayama, 1954: 192) (*Dryocoetes*).

• *Coccotrypes nubilus* (Blandford, 1894c: 95) (*Dryocoetes*).

= *Thamnurgides brevipilosus* Beeson, 1939: 298.

= *Thamnurgides corticis* Beeson, 1939: 298.

= *Thamnurgides himalayensis* Beeson, 1939: 299.

= *Thamnurgides parvus* Beeson, 1939: 297.

= *Poecilips mauritianus* Browne, 1970: 569.

• *Coccotrypes obtusicollis* (Schedl, 1975g: 455) (*Poecilips*).

• *Coccotrypes omissus* Schedl, 1979e: 101.

• *Coccotrypes palmarus* Eggers, 1933f: 8.

• *Coccotrypes papuanus* (Eggers, 1923b: 148) (*Dendrurgus*).

= *Thamnurgides glandis* Beeson, 1939: 287.

= *Thamnurgides rubidus* Beeson, 1939: 290.

= *Poecilips decipiens* Browne, 1972: 21.

• *Coccotrypes parvus* Sampson, 1914: 391.

• *Coccotrypes perditor* Blandford, 1894c: 99.

• *Coccotrypes petioli* (Browne in: Beaver and Browne 1979: 596) (*Poecilips*).

• *Coccotrypes politus* (Browne, 1970: 568) (*Poecilips*).

• *Coccotrypes precarius* Bright, 2019: 237.

• *Coccotrypes priesneri* Schedl, 1950b: 25.

• *Coccotrypes pteridophytae* (Schedl, 1968c: 266) (*Poecilips*).

• *Coccotrypes punctatus* (Eggers, 1927b: 82) (*Thamnurgides*).

• *Coccotrypes queenslandi* (Schedl, 1942c: 180) (*Poecilips*).

• *Coccotrypes recticollis* (Schedl, 1970b: 235) (*Poecilips*).

• *Coccotrypes regularis* (Schedl, 1955e: 292) (*Poecilips*).

• *Coccotrypes rhizophorae* (Hopkins, 1915d: 48) (*Spermatoplex*).

= *Dendrurgus rhizophorae* Eggers, 1923b: 149.

= *Thamnurgides nephelii* Eggers, 1936e: 84.

= *Thamnurgides shanorum* Beeson, 1939: 296.

• *Coccotrypes robustulus* Wood, 1989: 178 (RN).

= *Poecilips robustus* Schedl, 1972h: 227.

• *Coccotrypes robustus* Eichhoff, 1878b: 313.

= *Coccotryes cylindricus* Schedl, 1948e: 116.

• *Coccotrypes rotundicollis* (Eggers, 1927c: 186) (*Poecilips*).

= *Poecilips congonus* Eggers, 1927c: 187.

= *Poecilips rugulosus* Eggers, 1932c: 294.

= *Poecilips intermedius* Eggers, 1940d: 107.

= *Poecilips latior* Eggers, 1940d: 105.

= *Poecilips subtuberculatus* Eggers, 1940d: 105.

• *Coccotrypes rugicollis* (Eggers, 1925: 157) (*Thamnurgides*).

• *Coccotrypes rutschuruensis* Eggers, 1940d: 103.

• *Coccotrypes salakensis* (Schedl, 1939j: 38) (*Poecilips*).

= *Poecilips punctatus* Eggers, 1927b: 85.

= *Thamnurgides opacifrons* Beeson, 1939: 294.

= *Poecilips acuminatus* Schedl, 1966c: 34 (RN).

• *Coccotrypes sannio* (Schaufuss, 1897a: 110) (*Poecilips*).

= *Poecilips bambesanus* Eggers, 1940d: 107.

• *Coccotrypes sculptilis* (Schedl, 1965f: 60) (*Poecilips*).

• *Coccotrypes sierraleonensis* (Eggers, 1932c: 292) (*Poecilips*).

= *Poecilips confusus* Eggers, 1940d: 106.

• *Coccotrypes similis* (Eggers, 1923b: 148) (*Dendrurgus*).

• *Coccotrypes solomonicus* (Browne, 1968: 112) (*Poecilips*).

• *Coccotrypes sparsepilosus* (Eggers, 1940d: 106) (*Poecilips*).

• *Coccotrypes sparserugosus* (Schedl, 1972k: 62) (*Poecilips*).

• *Coccotrypes spinipennis* (Schedl, 1955e: 293) (*Poecilips*).

• *Coccotrypes squamifer* (Schedl, 1975a: 355) (*Poecilips*).

• *Coccotrypes striatus* Eggers, 1920c: 33.

• *Coccotrypes strigicollis* Schedl, 1972h: 228.

• *Coccotrypes suaui* (Schedl, 1973c: 70) (*Poecilips*).

• *Coccotrypes subacuminatus* (Eggers, 1927e: 399) (*Poecilips*).

• *Coccotrypes subcribrosus* (Blandford, 1896e: 224) (*Xyleborus*).

= *Xyleborus infans* Hagedorn, 1910: 7.

• *Coccotrypes subcylindricus* (Schedl, 1942d: 24) (*Poecilips*).

• *Coccotrypes subdepressus* Schedl, 1948e: 115.

• *Coccotrypes subovalis* Eggers, 1932c: 291.

• *Coccotrypes subsulcatus* Schedl, 1965g: 1077.

• *Coccotrypes subvulgaris* (Browne, 1966: 246) (*Poecilips*).

• *Coccotrypes surinamensis* Schedl, 1948e: 116 (RN).

= *Coccotrypes brevipilosus* Eggers, 1951: 150.

• *Coccotrypes tahitensis* (Beeson, 1935b: 117) (*Thamnurgides*).

= *Thamnurgides striatus* Eggers, 1927b: 82.

= *Coccotryes striatulus* Wood, 1989: 178 (RN).

• *Coccotrypes taprobanus* (Blandford, 1896e: 203) (*Dryocoetes*).

• *Coccotrypes theae* Eggers, 1929b: 112.

• *Coccotrypes tunggali* (Schedl, 1942b: 178) (*Poecilips*).

• *Coccotrypes tutuilensis* (Beeson, 1929: 229) (*Thamnurgides*).

= *Poecilips fijianus* Schedl, 1942c: 179.

= *Poecilips tapatapoanus* Schedl, 1951f: 149.

• *Coccotrypes uniseriatus* Eggers, 1927e: 398.

• *Coccotrypes variabilis* (Beeson, 1939: 286) (*Thamnurgides*).

• *Coccotrypes vateriae* (Beeson, 1939: 293) (*Thamnurgides*).

• *Coccotrypes vulgaris* (Eggers, 1923b: 151) (*Dendrurgus*).

= *Poecilips brevior* Eggers, 1927b: 84.

#### *Cynanchophagus* Aksent’ev

1 sp.

• *Cynanchophagus cornutus* Aksent’ev, 1988: 150.

#### *Cyrtogenius* Strohmeyer

105 spp.

• *Cyrtogenius abruptodeclivis* Bright, 1994: 258.

• *Cyrtogenius acuminatus* (Schedl, 1975h: 221) (*Eidophelus*).

• *Cyrtogenius aequalis* (Schedl, 1979e: 100) (*Ozodendron*).

• *Cyrtogenius aethiopicus* (Eggers, 1927c: 178) (*Dryocoetes*).

• *Cyrtogenius affinis* (Schedl, 1940b: 439) (*Pelicerus*).

• *Cyrtogenius africanus* (Schreiner, 1882: 246) (*Dryocoetes*).

• *Cyrtogenius africus* Wood, 1988c: 196 (RN).

= *Metahylastes africanus* Eggers, 1922c: 165.

• *Cyrtogenius agathis* (Browne, 1980c: 486) (*Ozodendron*).

• *Cyrtogenius alternantes* Schedl, 1975a: 353.

• *Cyrtogenius alternatus* (Eggers, 1927e: 398) (*Thamnurgides*).

• *Cyrtogenius anisopterae* (Browne, 1985b: 291) (*Ozodendron*).

• *Cyrtogenius aries* (Schedl, 1969c: 158) (*Artepityophthorus*).

• *Cyrtogenius bicolor* Strohmeyer, 1910b: 127.

= *Tiarophorus kivuensis* Nunberg, 1961b: 344.

• *Cyrtogenius borneanus* Beaver, 2011: 280 (RN).

= *Eidophelus borneensis* Browne, 1984a: 153.

• *Cyrtogenius borneensis* Schedl, 1967b: 129.

• *Cyrtogenius brevior* (Eggers, 1927b: 86) (*Pelicerus*).

• *Cyrtogenius cavifrons* Bright, 1994: 259.

• *Cyrtogenius chirindaensis* (Schedl, 1948a: 666) (*Dryocoetes*).

• *Cyrtogenius chlorophorae* (Schedl, 1957b: 66) (*Dryocoetes*).

• *Cyrtogenius congonus* (Eggers, 1924: 105) (*Dryocoetes*).

• *Cyrtogenius cribricollis* Schedl, 1958a: 875.

= *Cyrtogenius dryocoetoides* Nunberg, 1961b: 338.

• *Cyrtogenius cribripennis* Schedl, 1962g: 68.

= *Cyrtogenius cribripennis* Schedl, 1964h: 43.

• *Cyrtogenius curtus* (Schedl, 1973c: 71) (*Ozodendron*).

• *Cyrtogenius cyclopus* (Schedl, 1940b: 439) (*Pelicerus*).

• *Cyrtogenius declivis* (Schedl, 1955e: 294) (*Carposinus*).

• *Cyrtogenius depressus* (Browne, 1963a: 243) (*Mimidendrulus*).

• *Cyrtogenius detectus* Schedl, 1972a: 289.

• *Cyrtogenius dimorphus* (Schedl, 1936e: 527) (*Dryocoetes*).

• *Cyrtogenius dubius* (Eggers, 1924: 105) (*Dryocoetes*).

• *Cyrtogenius elongatulus* Wood, 1988c: 197 (RN).

= *Eidophelus elongatus* Schedl, 1979e: 102.

• *Cyrtogenius elongatus* (Eggers, 1927b: 85) (*Pelicerus*).

• *Cyrtogenius festivus* Schedl, 1975h: 220.

• *Cyrtogenius fijianus* (Schedl, 1951f: 150) (*Ozopemon*).

= *Dryocoetes noumeanus* Browne, 1970: 563.

• *Cyrtogenius frigidus* (Schedl, 1942b: 178) (*Orosiotes*).

• *Cyrtogenius glaber* (Schedl, 1939b: 344) (*Eulepiops*).

• *Cyrtogenius glabrata* Bright, 1994: 259.

• *Cyrtogenius gracilior* Beaver, 2011: 280 (RN).

= *Eidophelus gracilis* Browne, 1984a: 152.

= *Eidophelus levis* Johnson in: Johnson et al. 2020a: 54 (RN).

• *Cyrtogenius gracilis* (Schedl, 1974a: 462) (*Ozodendron*).

• *Cyrtogenius gracillimus* Wood, 1988c: 197 (RN).

= *Cyrtogenius gracilis* Browne, 1984e: 95.

• *Cyrtogenius grandis* (Beeson, 1929: 232) (*Pelicerus*).

• *Cyrtogenius granistriatus* (Schedl, 1942c: 180) (*Orosiotes*).

• *Cyrtogenius grossepunctatus* (Schedl, 1939b: 346) (*Pelicerus*).

• *Cyrtogenius hirtellus* Schedl, 1953c: 89.

• *Cyrtogenius hirtus* (Eggers, 1923b: 162) (*Dryocoetes*).

• *Cyrtogenius hornus* (Schedl, 1975a: 356) (*Eidophelus*).

• *Cyrtogenius impar* Schedl, 1953c: 88.

• *Cyrtogenius inermis* Browne, 1983c: 72.

• *Cyrtogenius kadazanus* Bright, 1994: 260.

• *Cyrtogenius kumamotoensis* (Niisima, 1917: 1) (*Orosiotes*).

= *Lymantor kabei* Murayama, 1955: 87.

• *Cyrtogenius laevis* (Browne, 1984b: 289) (*Ozodendron*).

• *Cyrtogenius lineatopunctatus* (Eggers, 1927e: 401) (*Xyleborus*).

• *Cyrtogenius longipennis* Browne, 1965: 196.

= *Ozodendron elongatus* Schedl, 1964i: 244.

= *Cyrtogenius elongatissimus* Wood, 1988c: 196 (RN).

• *Cyrtogenius lowi* Bright, 1994: 261.

• *Cyrtogenius luteus* (Blandford, 1894c: 94) (*Dryocoetes*).

= *Carposinus pini* Hopkins, 1915d: 47.

= *Orosiotes formosanus* Schedl, 1952h: 63.

• *Cyrtogenius madagascariensis* Schedl, 1965f: 61.

• *Cyrtogenius major* Strohmeyer, 1911a: 16.

• *Cyrtogenius malayensis* (Schedl, 1977c: 501) (*Dryocoetes*).

• *Cyrtogenius mandibularis* Bright, 1994: 261.

• *Cyrtogenius mayumbensis* (Schedl, 1966f: 369) (*Dryocoetes*).

• *Cyrtogenius mediosetosus* Bright, 1994: 262.

• *Cyrtogenius milletiae* (Schedl, 1952d: 9) (*Dryocoetes*).

• *Cyrtogenius minor* (Eggers, 1923b: 218) (*Pelicerus*).

= *Pelicerus minor robusts* Schedl, 1940b: 438.

• *Cyrtogenius movoliae* (Schedl, 1957b: 68) (*Mimidendrulus*).

• *Cyrtogenius mulungensis* (Schedl, 1952d: 10) (*Dryocoetes*).

• *Cyrtogenius nitidus* (Hagedorn, 1910: 1) (*Lepicerus*).

= *Pelicerus nitidus orientalis* Eggers, 1923b: 217.

• *Cyrtogenius nodulosus* Bright, 1994: 263.

• *Cyrtogenius obesus* Bright, 1994: 264.

• *Cyrtogenius papuae* Wood, 1988c: 197 (RN).

= *Pelicerus papuanus* Eggers, 1923b: 217.

• *Cyrtogenius papuanus* (Eggers, 1923b: 162) (*Dryocoetes*).

• *Cyrtogenius papuensis* Wood, 1988c: 197 (RN).

= *Eidophelus papuanus* Schedl, 1973c: 71.

• *Cyrtogenius parinarii* (Browne, 1983b: 556) (*Ozodendron*).

• *Cyrtogenius parvus* Browne, 1980d: 495.

• *Cyrtogenius perakensis* (Schedl, 1936b: 9) (*Dryocoetes*).

= *Dryocoetes castaneus* Browne, 1949: 896.

• *Cyrtogenius peregrinus* Schedl, 1972j: 50.

• *Cyrtogenius philippinensis* (Eggers, 1927b: 87) (*Pelicerus*).

• *Cyrtogenius piceus* Bright, 1994: 264.

• *Cyrtogenius polyphagus* (Schedl, 1952b: 12) (*Dryocoetes*).

• *Cyrtogenius preparvus* Browne, 1986b: 664.

• *Cyrtogenius prinavorus* Bright, 1994: 265.

• *Cyrtogenius pygmaeus* (Eggers, 1923b: 218) (*Pelicerus*).

= *Protopityophthorus durus* Schedl, 1973c: 73.

• *Cyrtogenius quercicolens* Bright, 1994: 265.

• *Cyrtogenius rugicollis* (Eggers, 1940f: 132) (*Orosiotes*).

• *Cyrtogenius ruginosus* Wood, 1988c: 197 (RN).

= *Mimidendrulus rugicollis* Browne, 1965: 194.

• *Cyrtogenius rugipennis* (Schedl, 1958i: 143) (*Carpophloeus*).

• *Cyrtogenius samoanus* (Eggers, 1928b: 174) (*Dryocoetes*).

= *Pelicerus granulifer* Beeson, 1929: 231.

• *Cyrtogenius sarawakensis* (Schedl, 1954e: 155) (*Dryocoetes*).

• *Cyrtogenius scabricollis* Browne, 1980b: 385.

• *Cyrtogenius scotti* Eggers, 1936b: 31.

• *Cyrtogenius sedlaceki* (Schedl, 1975a: 353) (*Ozodendron*).

• *Cyrtogenius silvaniae* Schedl, 1982: 285.

• *Cyrtogenius siporanus* (Eggers, 1923b: 163) (*Dryocoetes*).

• *Cyrtogenius smetanai* Bright, 1994: 266.

• *Cyrtogenius subacuminatus* (Schedl, 1939b: 347) (*Pelicerus*).

• *Cyrtogenius subgranosus* (Schedl, 1973c: 72) (*Eidophelus*).

= *Eidophelus subgranosus affinis* Schedl, 1973c: 73.

• *Cyrtogenius subsulcatus* (Schedl, 1979e: 102) (*Eidophelus*).

• *Cyrtogenius sulcatus* (Schedl, 1979e: 103) (*Eidophelus*).

• *Cyrtogenius suturalis* Browne, 1970: 564.

• *Cyrtogenius tanae* Bright, 1994: 267.

• *Cyrtogenius tenuis* (Schedl, 1958e: 102) (*Carposinus*).

• *Cyrtogenius tikaludus* Bright, 1994: 268.

• *Cyrtogenius tuberculatus* (Schedl, 1973c: 72) (*Eidophelus*).

• *Cyrtogenius tuberculifer* Schedl, 1950d: 109.

• *Cyrtogenius unicus* (Schedl, 1975a: 355) (*Eidophelus*).

• *Cyrtogenius variipennis* (Schedl, 1971e: 386) (*Xyleborus*).

• *Cyrtogenius vaticae* (Nunberg, 1961a: 617) (*Taphroborus*).

#### *Dactylotrypes* Eggers

1 sp.

• *Dactylotrypes longicollis* (Wollaston, 1864: 256) (*Xyloterus*).

= *Dactylotrypes uyttenboogaarti* Eggers, 1927d: 38.

= *Dactylotrypes draconis* Enderlein, 1929: 148.

#### *Dendrocranulus* Schedl

51 spp.

• *Dendrocranulus acutus* Wood, 1979: 139 (E).

• *Dendrocranulus ambiguus* Bright, 2019: 241.

• *Dendrocranulus barbatulus* Bright, 2019: 242.

• *Dendrocranulus barbatus* Schedl, 1939e: 172.

• *Dendrocranulus brasiliensis* (Schedl, 1963f: 224) (*Xylocleptes*).

• *Dendrocranulus carbonarius* (Ferrari, 1867: 41) (*Xylocleptes*).

= *Xylocleptes anonae* Hopkins, 1915d: 43.

= *Xylocleptes floridensis* Hopkins, 1915d: 43.

= *Xylocleptes guatemalensis* Hopkins, 1915d: 44.

= *Dendrocranulus parallelus* Schedl, 1938b: 172.

• *Dendrocranulus caymanensis* Bright, 2019: 244.

• *Dendrocranulus columbianus* (Schedl, 1937b: 167) (Dendrocranulus*).

= *Dendrocranulus limatus* Wood, 1974a: 22.

• *Dendrocranulus conditus* Wood, 1974a: 23.

• *Dendrocranulus confinis* Wood, 1974a: 26.

• *Dendrocranulus consimilis* Wood, 1974a: 23.

• *Dendrocranulus convexus* Bright, 2019: 244.

• *Dendrocranulus costalimai* Schedl, 1938b: 169.

= *Dendrocranulus sechii* Nunberg, 1972: 193.

• *Dendrocranulus costaricensis* Schedl, 1938b: 171.

• *Dendrocranulus cucurbitae* (LeConte, 1879: 519) (*Xylocleptes*).

= *Xylocleptes californicus* Hopkins, 1915d: 44.

= *Xylocleptes punctatus* Hopkins, 1915d: 44.

= *Xylocleptes venturina* Hopkins, 1915d: 44.

• *Dendrocranulus declivis* (Schedl, 1937b: 166) (Dendrocranulus*).

• *Dendrocranulus dervish* Petrov & Mandelshtam, 2016: 282.

• *Dendrocranulus diversus* Wood, 1971a: 37.

• *Dendrocranulus fulgens* Bright, 2019: 245.

• *Dendrocranulus fulgidus* Wood, 1974a: 25.

• *Dendrocranulus gracilis* Wood, 1982: 227.

• *Dendrocranulus hispaniolus* Bright, 2019: 246.

• *Dendrocranulus knausi* (Hopkins, 1915d: 44) (*Xylocleptes*).

• *Dendrocranulus knizeki* Petrov & Mandelshtam, 2016: 281.

• *Dendrocranulus limbatus* (Blandford, 1898a: 190) (*Dryocoetes*).

• *Dendrocranulus limbellus* Wood, 1979: 139.

• *Dendrocranulus limitaris* Wood, 1979: 140.

• *Dendrocranulus limus* Wood, 1971a: 38.

• *Dendrocranulus macilentus* (Blandford, 1898a: 190) (*Dryocoetes*).

= *Dendrocranulus grossopunctatus* Schedl, 1937b: 166.

• *Dendrocranulus major* Schedl, 1938b: 170.

• *Dendrocranulus maurus* (Blandford, 1898a: 191) (*Dryocoetes*).

= *Dendrocranulus huehuetanus* Schedl, 1940a: 344.

• *Dendrocranulus melaenus* (Eichhoff, 1872a: 136) (*Dryocoetes*).

• *Dendrocranulus mexicanus* Wood, 1986a: 272.

• *Dendrocranulus modus* Wood, 1979: 140.

• *Dendrocranulus pilosus* Eggers, 1943d: 357.

• *Dendrocranulus pinguis* Wood, 1979: 140.

• *Dendrocranulus pumilus* Wood, 1971a: 38.

• *Dendrocranulus reditus* Wood, 1974a: 23.

• *Dendrocranulus rubripes* (Eggers, 1943d: 358) (*Pityophthorus*).

• *Dendrocranulus rudis* Wood, 1974a: 26.

• *Dendrocranulus satipensis* Petrov & Mandelshtam, 2016: 286.

• *Dendrocranulus schedli* Wood, 1966: 23 (RN).

= *Dendrocranulus cucurbitae* Schedl, 1939a: 45.

• *Dendrocranulus securus* Wood, 1974a: 25.

• *Dendrocranulus sobrinus* Wood, 1984a: 114.

• *Dendrocranulus tardulus* Wood, 1971a: 37.

• *Dendrocranulus tardus* (Schedl, 1937b: 165) (Dendrocranulus*).

• *Dendrocranulus tayuyaensis* Schedl, 1939e: 173.

• *Dendrocranulus uncinatus* (Eichhoff, 1872a: 134) (*Xylocleptes*).

• *Dendrocranulus vicinalis* Wood, 1974a: 24.

• *Dendrocranulus vicinus* Wood, 1974a: 25.

• *Dendrocranulus vinealis* Wood, 1974a: 24.

#### *Dryocoetes* Eichhoff

40 spp. (1 sp. with 2 sspp.).

• *Dryocoetes affaber* (Mannerheim, 1852: 359) (*Bostrichus*).

= *Dryocoetes pubescens* Swaine, 1912: 350.

= *Dryocoetes piceae* Hopkins, 1915d: 51.

• *Dryocoetes affinis* Blandford, 1894c: 93.

• *Dryocoetes alni* (Georg, 1856: 59) (*Bostrichus*).

= *Tomicus marshami* Rye, 1868: 188.

= *Dryocoetes similis* Eggers, 1911b: 121.

= *Dryocoetes leonhardi* Eggers, 1912b: 49.

• *Dryocoetes asperatus* Buhroo & Zubair, 2024: 23.

• *Dryocoetes ater* Eggers, 1925: 157.

• *Dryocoetes autographus* (Ratzeburg, 1837: 160) (*Bostrichus*).

= *Bostrichus villosus* Herbst, 1793: 121.

= *Bostrichus septentrionis* Mannerheim, 1843: 298.

= *Bostrichus semicastaneus* Mannerheim, 1852: 298.

= *Bostrichus victoris* Mulsant & Rey, 1853a: 205.

= *Bostrichus victoris* Mulsant & Rey, 1853b: 91.

= *Dryocoetes americanus* Hopkins, 1915d: 51.

= *Dryocoetes pseudotsugae* Swaine, 1915b: 360.

= *Dryocoetes suecicus* Eggers, 1923a: 139.

= *Dryocoetes alternans* Eggers, 1931b: 30.

= *Dryocoetes brasiliensis* Schedl, 1940c: 207.

= *Dryocoetes artepunctatus* Eggers, 1941d: 122.

= *Dryocoetes longicollis* Eggers, 1941d: 121.

= *Dryocoetes autographus sachalinensis* Sokanovskiy, 1960: 675.

• *Dryocoetes baikalicus* Reitter, 1899: 286.

• *Dryocoetes betulae* Hopkins, 1915d: 50.

= *Dryocoetes eichhoffi* Hopkins, 1894: 279.

= *Dryocoetes liquidambaris* Hopkins, 1915d: 51.

• *Dryocoetes brevipilosus* Murayama, 1957b: 604.

• *Dryocoetes brownei* Mandelshtam & Petrov, 2010a: 180.

• *Dryocoetes carpinivorus* Choo & Woo, 1989: 58.

• *Dryocoetes caryi* Hopkins, 1915d: 50.

• *Dryocoetes cerasi* Eggers, 1942a: 32.

= *Dryocoetes cerasi* Stark, 1950: 229.

• *Dryocoetes confusus* Swaine, 1912: 351.

= *Dryocoetes abietis* Hopkins, 1915d: 52.

• *Dryocoetes conspicuus* Bright, 1994: 268.

• *Dryocoetes cornivorus* (Murayama, 1950a: 63) (*Xyleborus*).

• *Dryocoetes cristatus* Inouye & Nobuchi, 1959: 122.

• *Dryocoetes eichhoffi* Ferrari, 1867: 29.

• *Dryocoetes granicollis* (LeConte, 1868: 162) (*Xyleborus*).

• *Dryocoetes hectographus* Reitter, 1913: 76.

= *Ozopemon ater* Eggers, 1933d: 101.

= *Dryocoetes formosanus* Nobuchi, 1967: 18.

• *Dryocoetes himalayensis* Strohmeyer, 1908c: 161.

• *Dryocoetes indicus* Stebbing, 1914: 549.

• *Dryocoetes infuscatus* Murayama, 1937: 370.

= *Dryocoetes orientalis* Kurentsov, 1941: 169.

= *Dryocoetes orientalis pilosiusculus* Kurentsov, 1948: 51.

• *Dryocoetes italus* Eggers, 1940b: 44.

• *Dryocoetes karamatsu* Sawamoto, 1940: 97.

• *Dryocoetes krivolutzkajae* Mandelshtam, 2001: 11.

• *Dryocoetes longipilus* (Eggers, 1926b: 146) (*Xyleborus*).

• *Dryocoetes niijimai* Nobuchi, 1959a: 11.

• *Dryocoetes padi* Kurentsov, 1941: 170.

= *Dryocoetes padi* Stark, 1952: 335.

• *Dryocoetes pilosus* Blandford, 1894c: 92.

• *Dryocoetes pini* Niisima, 1909: 152.

• *Dryocoetes pumilio* Eichhoff, 1878b: 295.

• *Dryocoetes quadrisulcatus* Strohmeyer, 1908b: 72.

• *Dryocoetes rotundicollis* Eggers, 1928b: 174.

• *Dryocoetes rugicollis* Eggers, 1926a: 137.

• *Dryocoetes sechelti* Swaine, 1915b: 359.

• *Dryocoetes striatus* Eggers, 1933a: 8.

= *Dryocoetes abietinus* Kôno & Tamanuki, 1939: 90.

• *Dryocoetes uniseriatus* Eggers, 1926b: 145.

• *Dryocoetes ussuriensis* Eggers, 1933a: 8.

= *Dryocoetes ussuriensis rugulosus* Eggers, 1933a: 9.

• *Dryocoetes villosus minor* Eggers, 1908b: 122.

= *Dryocoetes sardus* Strohmeyer, 1912a: 57.

• *Dryocoetes villosus villosus* (Fabricius, 1792: 367) (*Bostrichus villosus*).

= *Tomicus micrographus* Billberg, 1820: 46 (RN).

= *Bostrichus histerinus* Dufour, 1843: 91.

= *Dryocoetes eichhoffi* Ferrari, 1867: 29.

= *Bostrichus villosus starhoni* Reitter, 1913: 77.

#### *Dryocoetiops* Schedl

11 spp.

• *Dryocoetiops apatoides* (Eichhoff in: Chapuis and Eichhoff 1876: 201) (*Dryocoe­tes*).

• *Dryocoetiops bicolor* Schedl, 1970f: 360.

• *Dryocoetiops inopinatus* (Schedl, 1955e: 294) (*Dryocoetes*).

• *Dryocoetiops krivetsae* Kerchev, Mandelshtam, Bykov & Ilinsky in: Kerchev et al. 2023: 269.

• *Dryocoetiops laevis* (Strohmeyer, 1911b: 22) (*Ozopemon*).

= *Ozopemon dipterocarpi* Hopkins, 1915d: 49.

= *Ozopemon diversicolor* Eggers, 1923b: 160.

= *Xyleborus orbus* Browne, 1966: 249.

= *Poecilips loebli* Schedl, 1972h: 227.

• *Dryocoetiops moestus* (Blandford, 1894c: 96) (*Dryocoetes*).

= *Dryocoetes dinoderoides* Blandford, 1894c: 97.

= *Dryocoetes coffeae* Eggers, 1923b: 161.

= *Dryocoetes javanus* Eggers, 1936e: 87.

= *Dryocoetes hirsutus* Schedl, 1939b: 348.

= *Dryocoetes australis* Schedl, 1942c: 181.

= *Dryocoetes eugeniae* Schedl, 1942b: 180.

= *Dryocoetes malaccensis* Schedl, 1942b: 180.

= *Dryocoetes tonkinensis* Schedl, 1942b: 179.

= *Pseudopoecilips taradakensis* Murayama, 1957b: 618.

• *Dryocoetiops nitidus* (Schedl, 1942b: 179) (*Dryocoetes*).

• *Dryocoetiops pasohensis* Beaver, Smith & Sanguansub, 2019b: 240.

• *Dryocoetiops salebrosus* Beaver, Smith & Sanguansub, 2019b: 241.

• *Dryocoetiops schultzei* (Schedl, 1962e: 88) (*Dryocoetes*).

• *Dryocoetiops semigranulatus* (Schedl, 1936b: 10) (*Dryocoetes*).

= *Dryocoetes kepongi* Schedl, 1953f: 296.

#### *Lymantor* Løvendal

4 spp. (1 sp. with 2 sspp.).

• *Lymantor aceris aceris* (Lindemann, 1875a: 140) (*Dryocoetes aceris*).

• *Lymantor aceris schabliovskii* Stark, 1936: 153 (*Lymantor schabliovskii*).

• *Lymantor alaskanus* Wood, 1978: 399.

• *Lymantor coryli* (Perris, 1855: lxxviii) (*Tomicus*).

= *Lymantor sepicola* Løvendal, 1889a: 25.

• *Lymantor decipiens* (LeConte, 1878b: 624) (*Xylocleptes*).

#### *Minyotrypetes* Bright

1 sp.

• *Minyotrypetes primus* Bright, 2019: 247.

#### *Neocultus* Bright

1 sp.

• *Neocultus thomasi* Bright, 2019: 248.

#### *Ozopemon* Hagedorn

21 spp.

• *Ozopemon aplanatus* Schedl, 1942b: 177.

• *Ozopemon augustae* Eggers, 1923b: 159.

= *Coccotrypes kuscheli* Schedl, 1979g: 104.

• *Ozopemon borneensis* Eggers, 1923b: 154.

• *Ozopemon brevis* Eggers, 1923b: 155.

• *Ozopemon brownei* Schedl, 1953f: 297.

• *Ozopemon fuscicollis* Hagedorn, 1910: 3.

• *Ozopemon giganteus* Schedl, 1934b: 38.

• *Ozopemon granulatus* Schedl, 1936e: 526.

• *Ozopemon gravidus* (Blandford, 1896e: 206) (*Dryocoetes*).

• *Ozopemon grossepunctatus* Eggers, 1923b: 155.

• *Ozopemon latus* Eggers, 1923b: 156.

• *Ozopemon obanus* Hagedorn, 1910: 3.

= *Ozopemon cylindricus* Eggers, 1923b: 156.

• *Ozopemon papuanus* Eggers, 1923b: 157.

• *Ozopemon parinarii* Hopkins, 1915d: 48.

• *Ozopemon perfacilis* Eggers, 1939b: 8.

• *Ozopemon regius* Hagedorn, 1908: 382.

• *Ozopemon rugatus* (Blandford, 1896e: 204) (*Dryocoetes*).

• *Ozopemon similis* Eggers, 1927b: 87.

• *Ozopemon sumatranus* (Blandford, 1896e: 205) (*Dryocoetes*).

• *Ozopemon theklae* Hagedorn, 1910: 2.

= *Ozopemon singalangicus* Eggers, 1923b: 153.

• *Ozopemon uniseriatus* Eggers, 1923b: 158.

#### *Peridryocoetes* Wood

7 spp.

• *Peridryocoetes crassus* (Eggers, 1927e: 397) (*Dryocoetes*).

• *Peridryocoetes minutissimus* (Schedl, 1979f: 127) (*Dryocoetes*).

• *Peridryocoetes nitens* (Schedl, 1964i: 243) (*Ozodendron*).

• *Peridryocoetes peliciformis* (Schedl, 1936c: 29) (*Xyleborus*).

• *Peridryocoetes pinguis* (Browne, 1983b: 557) (*Xyleborus*).

• *Peridryocoetes queenslandi* (Schedl, 1972e: 146) (Peridryocoetes*).

• *Peridryocoetes squamipennis* (Schedl, 1979e: 101) (Peridryocoetes*).

#### *Pseudothamnurgus* Eggers

5 spp.

• *Pseudothamnurgus mediterraneus* (Eggers, 1910a: 560) (*Dryocoetes*).

• *Pseudothamnurgus nitidicollis* (Reitter, 1887: 197) (*Dryocoetes*).

• *Pseudothamnurgus normandi* (Eggers, 1910b: 37) (*Thamnurgus*).

= *Pseudothamnurgus elegans* Wichmann, 1913a: 118.

• *Pseudothamnurgus orientalis* (Schedl, 1978c: 38) (*Thamnurgus*).

• *Pseudothamnurgus scrutator* (Pandellé in: Eichhoff 1885: 136) (*Thamnurgus*).

#### *Taphronurgus* Reitter

1 sp.

• *Taphronurgus exul* (Reitter, 1891b: 199) (*Thamnurgus*).

#### *Taphrorychus* Eichhoff

20 spp.

• *Taphrorychus alni* Pfeffer, 1940b: 53.

• *Taphrorychus betulae* Schedl, 1974c: 87.

• *Taphrorychus bicolor* (Herbst, 1793: 116) (*Bostrichus*).

= *Ips fuscus* Marsham, 1802: 53.

• *Taphrorychus carpini* (Kurentsov, 1941: 167) (*Dryocoetes*).

= *Dryocoetes carpini* Eggers, 1942a: 32.

= *Dryocoetes carpini* Stark, 1952: 329.

• *Taphrorychus ceratoniae* Peyerimhoff, 1926: 388.

= *Taphrorychus cribripennis* Eggers, 1944a: 142.

• *Taphrorychus coronatus* Eggers, 1944a: 141.

• *Taphrorychus hewetti* (Stebbing, 1908: 11) (*Dryocoetes*).

• *Taphrorychus hirtellus* Eichhoff, 1878b: 208.

= *Taphrorychus mecedanus* Reitter, 1913: 95.

• *Taphrorychus krivolutskayae* Johnson **nom. nov**. (RN).

= *Dryocoetes aceris* Krivolutskaya, 1968: 58.

• *Taphrorychus lenkoranus* Reitter, 1913: 96.

• *Taphrorychus machnovskii* (Sokanovskiy, 1954: 17) (*Hypothenemus*).

• *Taphrorychus mikuniyamensis* (Murayama, 1957b: 616) (*Pseudopoecilips*).

• *Taphrorychus minor* Eggers, 1923a: 137.

• *Taphrorychus picipennis* (Eggers, 1926a: 138) (*Dryocoetes*).

• *Taphrorychus pilosus* (Murayama, 1957b: 619) (*Pseudopoecilips*).

= *Pseudopoecilips pilosus brevipilosus* Murayama, 1957b: 619.

• *Taphrorychus primitus* (Schedl, 1965a: 341) (*Taphroterus*).

• *Taphrorychus ramicola* (Reitter, 1895: 94) (*Dryocoetes*).

= *Dryocoetes pusillus* Eggers, 1933a: 7.

• *Taphrorychus siculus* Eggers, 1908b: 121.

• *Taphrorychus striatus* Nobuchi, 1966: 52.

• *Taphrorychus villifrons* (Dufour, 1843: 91) (*Bostrichus*).

= *Bostrichus bulmerincqii* Kolenati, 1846: 39.

= *Dryocoetes capronatus* Perris, 1866: 193.

= *Taphrorychus bulmerincqii roubali* Pfeffer, 1940b: 53.

= *Taphrorychus schimitscheki* Eggers, 1940g: 38.

= *Taphrorychus splendens* Sokanovskiy, 1958: 39.

#### *Thamnurgus* Eichhoff

32 spp.

• *Thamnurgus africanus* Eggers, 1924: 108.

• *Thamnurgus armeniacus* Reitter, 1897: 244.

= *Thamnurgus posticepunctatus* Eggers, 1937a: 334.

• *Thamnurgus brylinskyi* Reitter, 1889a: 40.

• *Thamnurgus capensis* Schedl, 1977a: 396.

• *Thamnurgus caucasicus* Reitter, 1887: 195.

• *Thamnurgus characiae* Rosenhauer, 1878: 162.

= *Thamnurgus sardus* Eggers, 1912a: 114.

• *Thamnurgus concavifrons* (Schedl, 1950g: 207) (*Xylocleptes*).

• *Thamnurgus cylindricus* (Eggers, 1927c: 181) (*Xylocleptes*).

• *Thamnurgus delphinii* (Rosenhauer, 1856: 302) (*Bostrichus*).

= *Thamnurgus semirufus* Reitter, 1906b: 36.

= *Thamnurgus holtzi* Strohmeyer, 1907: 6.

= *Thamnurgus robustus* Eggers, 1908b: 122.

• *Thamnurgus elegans* Schedl, 1957b: 64.

• *Thamnurgus elongatus* Schedl, 1969b: 14.

• *Thamnurgus euphorbiae* (Küster, 1845: 39) (*Bostrichus*).

= *Thamnurgus siculus* Eggers, 1912a: 115.

• *Thamnurgus euryopsis* Schedl, 1955b: 218.

• *Thamnurgus granulicollis* Schedl, 1957b: 65.

• *Thamnurgus grossepunctatus* Schedl, 1977a: 397.

• *Thamnurgus interpunctatus* Schedl, 1950d: 105.

• *Thamnurgus joliveti* Nunberg, 1973: 18.

• *Thamnurgus kaltenbachi* (Bach, 1849: 199) (*Bostrichus*).

= *Thamnurgus declivis* Reitter, 1897: 244.

= *Thamnurgus csikii* Endrödi, 1957: 307.

• *Thamnurgus kenyae* Lepesme, 1942b: 269.

= *Thamnurgus wittei* Eggers, 1943e: 64.

• *Thamnurgus lobeliae* Eggers, 1933e: 23.

= *Thamnurgus latus* Lepesme, 1942b: 268.

• *Thamnurgus longipilus* Schedl, 1938a: 456.

• *Thamnurgus mairei* Peyerimhoff, 1949: 302.

• *Thamnurgus mandibularis* Schedl, 1953c: 90.

• *Thamnurgus nitellus* Schedl, 1953c: 92.

• *Thamnurgus nitidulus* Schedl, 1953c: 91.

• *Thamnurgus nitidus* Browne, 1980e: 773.

• *Thamnurgus pegani* Eggers, 1933a: 6.

• *Thamnurgus petzi* Reitter, 1901b: 182.

= *Thamnurgus rossicus* Alekseev, 1957: 161.

• *Thamnurgus punctatissimus* Eggers, 1944c: 95.

• *Thamnurgus senecionis* Schedl, 1938a: 456.

= *Thamnurgus jeanneli* Lepesme, 1942b: 268.

= *Thamnurgus kinangopensis* Lepesme, 1942b: 270.

= *Thamnurgus ugandensis* Nunberg, 1961a: 614.

• *Thamnurgus varipes* Eichhoff, 1878b: 212.

• *Thamnurgus zukwalae* Scott, 1935: 282.

#### *Tiarophorus* Schreiner

8 spp.

• *Tiarophorus camerunus* (Hagedorn, 1908: 374) (*Hypaspistes*).

• *Tiarophorus capucinus* Schedl, 1966f: 369.

• *Tiarophorus decellei* Browne, 1973: 285.

• *Tiarophorus elongatus* Schreiner, 1882: 247.

• *Tiarophorus gardneri* Schedl, 1958a: 874.

• *Tiarophorus hyaspistis* Schedl, 1941c: 399.

• *Tiarophorus intermedius* Schedl, 1954d: 77.

• *Tiarophorus praeruptus* (Eggers, 1925: 158) (*Pseudothamnurgus*).

#### *Triotemnus* Wollaston

16 spp.

• *Triotemnus aethiopicus* Eggers, 1936b: 31.

• *Triotemnus antoinei* Peyerimhoff, 1949: 299.

• *Triotemnus batelkai* Knížek, 2010: 197.

• *Triotemnus grangeri* (Peyerimhoff, 1949: 253) (*Lymantor*).

• *Triotemnus kabateki* Knížek, 2010: 201.

• *Triotemnus lepineyi* Balachowsky, 1949a: 98.

• *Triotemnus longicollis* Peyerimhoff, 1925: 11.

• *Triotemnus pilicornis* Wood, 1992: 87.

• *Triotemnus pseudolepineyi* Knížek, 2010: 194.

• *Triotemnus scrofa* (Schedl, 1975j: 454) (*Cladoctoporcus*).

• *Triotemnus socotraensis* Knížek, 2010: 199.

• *Triotemnus striatus* Eggers, 1936c: 37.

• *Triotemnus subretusus* Wollaston, 1864: 265.

• *Triotemnus ulianai* Gatti & Pennacchio, 2004: 103.

• *Triotemnus villiersi* Schedl, 1958c: 240.

• *Triotemnus yemenensis* Knížek, 2010: 203.

#### *Urocorthylus* Petrov, Mandelshtam & Beaver

2 spp.

• *Urocorthylus fanii* Wang, Beaver & Hulcr, 2017: 54.

• *Urocorthylus hirtellus* Petrov, Mandelshtam & Beaver, 2007: 251.

#### *Xylocleptes* Ferrari

28 spp.

• *Xylocleptes abruptus* Nunberg, 1973: 20.

• *Xylocleptes abyssinicus* (Schedl, 1966f: 364) (*Hoplitontus*).

• *Xylocleptes adeniae* (Schedl, 1952d: 11) (*Dryocoetes*).

• *Xylocleptes ater* Schedl, 1965g: 1075.

• *Xylocleptes baikiae* Schedl, 1958a: 879.

• *Xylocleptes bicuspis* Reitter, 1887: 196.

• *Xylocleptes bispinus* (Duftschmid, 1825: 92) (*Bostrichus*).

= *Scolytus retusus* Olivier, 1800a: 10.

• *Xylocleptes bituberculatus* Hagedorn, 1910: 1.

• *Xylocleptes biuncus* Reitter, 1894a: 45.

• *Xylocleptes brownei* Schedl, 1964g: 309 (RN).

= *Xylocleptes punctatus* Browne, 1963a: 243.

= *Xylocleptes immunis* Nunberg, 1969: 382 (RN).

• *Xylocleptes brunneus* (Nunberg, 1973: 16) (*Hylonius*).

• *Xylocleptes camerunus* (Schedl, 1941c: 400) (*Dendrocranulus*).

• *Xylocleptes castaneus* Browne, 1963a: 244.

• *Xylocleptes congonus* Hagedorn, 1908: 375.

• *Xylocleptes cribratus* Browne, 1973: 286.

• *Xylocleptes densepunctatus* Schedl, 1965g: 1076.

• *Xylocleptes granulipennis* Schedl, 1967c: 228.

• *Xylocleptes indicus* Schedl, 1971e: 374.

• *Xylocleptes irretitus* Browne, 1965: 196.

• *Xylocleptes jemeniae* (Schedl, 1975f: 353) (*Thamnurgus*).

• *Xylocleptes linnavuorii* Schedl, 1968b: 145.

• *Xylocleptes marginatus* (Hagedorn, 1912a: 353) (*Xestips*).

= *Xylocleptes meruensis* Nunberg, 1960: 291.

• *Xylocleptes robustus* (Schedl, 1971c: 198) (*Mimidendrulus*).

• *Xylocleptes rugulosus* (Schedl, 1972a: 290) (*Mimidendrulus*).

• *Xylocleptes sidanus* (Schedl, 1952b: 13) (*Dryocoetes*).

• *Xylocleptes sparsipunctatus* (Eggers, 1935e: 308) (*Cyrtogenius*).

• *Xylocleptes usambaricus* Schedl, 1950g: 208.

• *Xylocleptes villiersi* (Lepesme, 1942b: 270) (*Thamnurgus*).

### ERNOPORINI Nüsslin

4 genera, 175 spp.

#### *Eidophelus* Eichhoff

149 spp.

• *Eidophelus absonus* (Schedl, 1975a: 344) (*Cryphalomorphus*).

• *Eidophelus afer* (Schedl, 1970f: 359) (*Cryphalophilus*).

• *Eidophelus africanus* (Schedl, 1977a: 396) (*Stephanorhopalus*).

• *Eidophelus agnatus* (Schedl, 1942c: 168) (*Miocryphalus*).

• *Eidophelus aitutakii* (Beaver & Maddison, 1990: 1367) (*Ptilopodius*).

• *Eidophelus alniphagus* (Nobuchi, 1975: 43) (*Ernoporicus*).

• *Eidophelus alstoniae* Johnson in: Johnson et al. 2020a: 52 (RN).

= *Chiloxylon sumatranus* Schedl, 1970f: 358.

• *Eidophelus alternans* (Schedl, 1975a: 345) (*Cryphalomorphus*).

• *Eidophelus amanicus* (Eggers, 1920a: 239) (*Cryphalus*).

• *Eidophelus ankius* (Schedl, 1979e: 96) (*Cryphalomorphus*).

• *Eidophelus apicalis* (Schedl, 1971d: 11) (*Cryphalomorphus*).

• *Eidophelus approximatus* (Schedl, 1975a: 346) (*Cryphalomorphus*).

• *Eidophelus aspericollis* (Eichhoff, 1878a: 388) (*Lepicerus*).

= *Lepicerus aspericollis* Eichhoff, 1878b: 501.

= *Cryphalus stierlini* Eggers, 1911b: 121.

• *Eidophelus ater* (Eggers, 1923b: 142) (*Negritus*).

• *Eidophelus australis* (Schedl, 1942c: 175) (*Lepicerinus*).

• *Eidophelus badius* (Nobuchi, 1975: 44) (*Cryphalomorphus*).

• *Eidophelus bambusae* (Browne, 1983c: 69) (*Ptilopodius*).

• *Eidophelus bangensis* (Eggers, 1927b: 75) (*Cryphalomorphus*).

• *Eidophelus basilaris* (Wood, 1960a: 30) (*Cryphalomorphus*).

• *Eidophelus birosimensis* (Murayama, 1958: 935) (*Ernocryphalus*).

• *Eidophelus braderi* (Browne, 1965: 191) (*Cryphalomorphus*).

= *Cryphalomorphus orientalis* Schedl, 1971d: 11.

• *Eidophelus brighti* Johnson in: Johnson et al. 2020a: 52 (RN).

= *Hemicryphalus minutus* Bright, 1992: 187.

• *Eidophelus brimblecombei* (Schedl, 1972e: 146) (*Cryphalomorphus*).

• *Eidophelus brownei* Johnson in: Johnson et al. 2020a: 52 (RN).

= *Euptilius papuanus* Browne, 1983c: 70.

• *Eidophelus buruensis* (Eggers, 1926c: 300) (*Cryphalomorphus*).

• *Eidophelus camelliae* (Nobuchi, 1975: 45) (*Cryphalomorphus*).

• *Eidophelus candidus* (Nobuchi, 1975: 47) (*Cryphalomorphus*).

• *Eidophelus capucinus* (Schedl, 1971b: 283) (*Cryphalops*).

• *Eidophelus caucasicus* (Lindemann, 1877b: 373) (*Ernoporus*).

= *Cryphalus schreineri* Eichhoff, 1881: 185.

• *Eidophelus ceylonicus* (Schedl, 1959i: 475) (*Ptilopodius*).

• *Eidophelus cicatricosus* (Schedl, 1942c: 176) (*Lepicerinus*).

• *Eidophelus ciliatipennis* (Schedl, 1979g: 105) (*Miocryphalus*).

• *Eidophelus coccotrypanoides* (Schedl, 1939b: 343) (*Lepicerinus*).

• *Eidophelus communis* (Schaufuss, 1891: 12) (*Cryphalomorphus*).

• *Eidophelus concentralis* (Schedl, 1975a: 342) (*Cryphalophilus*).

= *Margadillius concentralis* Schedl, 1975a: 344.

• *Eidophelus confragosus* (Sampson, 1914: 386) (*Cryphalomorphus*).

• *Eidophelus corni* (Kurentsov, 1941: 162) (*Hypothenemus*).

• *Eidophelus corpulentus* (Schedl, 1965f: 54) (*Cryphalomorphus*).

• *Eidophelus corrugatus* (Schedl, 1950b: 19) (*Stephanorhopalus*).

• *Eidophelus creber* (Schedl, 1975a: 346) (*Cryphalomorphus*).

• *Eidophelus crenatus* (Sampson, 1914: 385) (*Cryphalomorphus*).

• *Eidophelus cylindricus* (Schedl, 1959i: 476) (*Cryphalomorphus*).

• *Eidophelus darwini* (Eichhoff, 1878a: 387) (*Scolytogenes*).

= *Scolytogenes darwinii* Eichhoff, 1878b: 497.

= *Negritus similis* Eggers, 1923b: 142.

= *Negritus major* Eggers, 1927b: 69.

= *Scolytogenes cryptolepis* Schedl, 1951i: 55.

= *Cryphalomorphus scolytomimoides* Nobuchi, 1975: 50.

• *Eidophelus devius* (Schedl, 1975b: 280) (*Cryphalophilus*).

• *Eidophelus dubiosus* (Wood, 1960a: 19) (*Ptilopodius*).

• *Eidophelus eggersi* (Schedl, 1962a: 490) (*Cryphalomorphus*) (RN).

= *Cryphalomorphus minor* Eggers, 1927b: 76.

• *Eidophelus euphorbiae* (Wood, 1980a: 91) (*Cryphalogenes*).

• *Eidophelus excellens* (Schedl, 1979e: 97) (*Cryphalomorphus*).

• *Eidophelus exiguus* (Wood, 1980a: 92) (*Cryphalogenes*).

• *Eidophelus exilis* (Yin, 2001: 327) (*Scolytogenes*).

• *Eidophelus eximius* (Schedl, 1942d: 9) (*Erischidias*).

• *Eidophelus fagi* (Fabricius, 1798: 157) (*Apate*).

= *Bostrichus serratus* Panzer, 1795: 288.

= *Ernoporus thomsoni* Ferrari, 1867: 14.

• *Eidophelus fijianus* (Schedl, 1950e: 42) (*Lepicerinus*).

• *Eidophelus formosanus* (Browne, 1981a: 129) (*Ptilopodius*).

• *Eidophelus fugax* (Schedl, 1975a: 347) (*Cryphalomorphus*).

• *Eidophelus fujisanus* (Nobuchi, 1975: 46) (*Cryphalomorphus*).

• *Eidophelus fulgens* (Schedl, 1975a: 348) (*Cryphalomorphus*).

• *Eidophelus fulgidus* (Schedl, 1975a: 348) (*Cryphalomorphus*).

• *Eidophelus fulvipennis* (Nobuchi, 1975: 47) (*Cryphalomorphus*).

• *Eidophelus furvus* Johnson in: Johnson et al. 2020a: 53 (RN).

= *Cryphalophilus ater* Schedl, 1972e: 146.

• *Eidophelus glabratus* (Yin, 2001: 330) (*Scolytogenes*).

• *Eidophelus gracilis* (Schedl, 1950e: 44) (*Lepicerinus*).

• *Eidophelus granulatus* (Wood, 1960a: 31) (*Cryphalomorphus*).

• *Eidophelus grobleri* (Schedl, 1962g: 68) (*Cryphalomorphus*).

• *Eidophelus hirtus* (Wood, 1974a: 18) (*Cryphalomorphus*).

• *Eidophelus hobohmi* (Schedl, 1955b: 215) (*Cryphalomorphus*).

• *Eidophelus hylesinopsis* (Schedl, 1975a: 349) (*Cryphalomorphus*).

• *Eidophelus imitans* Eichhoff in: Chapuis and Eichhoff 1876: 201.

= *Phellodendrophagus elegans* Krivolutskaya, 1956a: 56.

= *Ptilopodius nitidus* Schedl, 1959i: 475.

• *Eidophelus incultus* (Yin, 2001: 325) (*Scolytogenes*).

• *Eidophelus indicus* (Wood, 1989: 184) (*Scolytogenes*).

• *Eidophelus inermis* (Browne, 1984e: 94) (*Hypothenemus*).

• *Eidophelus insignis* (Browne, 1984a: 151) (*Hypothenemus*).

• *Eidophelus insularis* (Nobuchi, 1975: 48) (*Cryphalomorphus*).

• *Eidophelus insularum* (Krivolutskaya, 1968: 56) (*Hypothenemus insularus*).

= *Ericryphalus elongatus* Nobuchi, 1975: 42.

= *Hypothenemus krivolutskayae* Wood, 1992: 79 (RN).

• *Eidophelus jalappae* (Letzner, 1849: 99) (*Bostrichus*).

= *Ernoporides floridensis* Hopkins, 1915d: 34.

= *Ernoporides knabi* Hopkins, 1915d: 34.

= *Hypothenemus ritchiei* Sampson, 1918: 295.

= *Cryphalomorphus carabaicus* Schedl, 1951c: 96.

= *Cryphalomorphus minutissimus* Schedl, 1951c: 97.

= *Cryphalomorphus subtriatus* Schedl, 1952e: 360.

= *Cryphalomorphus alienus* Schedl, 1976: 65.

• *Eidophelus javanus* (Schedl, 1942d: 10) (*Ptilopodius*).

• *Eidophelus kanawhae* (Hopkins, 1915d: 35) (*Ernoporus*).

• *Eidophelus kinabaluensis* (Bright, 1992: 186) (*Hemicryphalus*).

• *Eidophelus landolphiae* (Schedl, 1961g: 133) (*Cryphalomorphus*).

• *Eidophelus leprosulus* (Browne, 1974a: 66) (*Cryphalomorphus*).

• *Eidophelus longipennis* (Eggers, 1936b: 30) (*Cryphalomorphus*).

• *Eidophelus lucidus* Johnson in: Johnson et al. 2020a: 54 (RN).

= *Lepicerinus pacificus* Schedl, 1942c: 175.

• *Eidophelus magnocularis* (Yin, 2001: 326) (*Scolytogenes*).

• *Eidophelus marquesanus* (Beeson, 1935a: 101) (*Ptilopodius*).

= *Ptilopodius zimmermanni* Schedl, 1951f: 143.

• *Eidophelus mauritianus* (Schedl, 1965f: 56) (*Cryphalomorphus*).

• *Eidophelus micans* (Eggers, 1927e: 396) (*Stephanoderes*).

• *Eidophelus minor* (Eggers, 1927b: 69) (*Negritus*).

• *Eidophelus minusculus* Johnson in: Johnson et al. 2020a: 54 (RN).

= *Eidophelus minutissimus* Schedl, 1962f: 191.

• *Eidophelus minutissimus* (Schedl, 1943: 34) (*Ptilopodius*).

• *Eidophelus minutus* Blandford, 1894c: 88.

• *Eidophelus mus* (Schedl, 1975a: 349) (*Cryphalomorphus*).

• *Eidophelus nanulus* (Wood, 1960a: 29) (*Cryphalomorphus*).

• *Eidophelus nigellatus* (Schedl, 1950e: 44) (*Lepicerinus*).

• *Eidophelus niger* Johnson in: Johnson et al. 2020a: 54 (RN).

= *Ernoporicus ater* Nobuchi, 1975: 54.

• *Eidophelus nubilus* (Wood, 1960a: 30) (*Cryphalomorphus*).

• *Eidophelus ocularis* (Schedl, 1966f: 368) (*Cryphalomorphus*).

• *Eidophelus onyanganus* (Schedl, 1941c: 391) (*Letzernella*).

= *Cryphalomorphus similaris* Schedl, 1965e: 8.

• *Eidophelus opacus* (Schedl, 1959i: 477) (*Cryphalomorphus*).

• *Eidophelus pacificus* (Schedl, 1941b: 111) (*Ptilopodius*).

• *Eidophelus papuanus* (Schedl, 1974a: 459) (*Cryphalomorphus*).

• *Eidophelus papuensis* (Wood, 1989: 179) (*Scolytogenes*) (RN).

= *Xylocryptus papuanus* Schedl, 1975a: 352.

• *Eidophelus paradoxus* (Wood, 1992: 80) (*Scolytogenes*) (RN).

= *Cryphalomorphus papuanus* Schedl, 1979e: 97.

• *Eidophelus parvulus* Johnson in: Johnson et al. 2020a: 54 (RN).

= *Cryphalus parvus* Browne, 1984a: 152.

• *Eidophelus parvus* (Hopkins, 1915d: 11) (*Hypothenoides*).

• *Eidophelus philippinensis* (Schedl, 1967b: 126) (*Erioschidias*).

• *Eidophelus pityophthorinus* (Schedl, 1943: 39) (*Lepicerinus*).

• *Eidophelus pleiocarpae* (Schedl, 1957b: 51) (*Cryphalomorphus*).

• *Eidophelus podocarpi* (Bright, 1992: 188) (*Hemicryphalus*).

• *Eidophelus polisquamosus* (Yin, 2001: 329) (*Scolytogenes*).

• *Eidophelus praeda* (Browne in: Beaver and Browne 1979: 589) (*Ptilopodius*).

• *Eidophelus puerarae* (Choo & Woo, 1989: 58) (*Scolytogenes*).

• *Eidophelus pumilionides* (Schedl, 1977c: 500) (*Cryphalomorphus*).

• *Eidophelus pumilus* (Wood, 1960a: 29) (*Cryphalomorphus*).

• *Eidophelus punctatulus* (Nobuchi, 1976: 72) (*Scolytogenes*) (RN).

= *Cryphalomorphus punctatus* Nobuchi, 1975: 49.

= *Cryphalomorphus nobuchii* Schedl, 1980c: 118 (RN).

• *Eidophelus punctatus* (Schedl, 1951i: 55) (*Cryphalomorphus*).

• *Eidophelus puncticollis* (Schedl, 1950e: 43) (*Lepicerinus*).

= *Cryphalomorphus grossepunctatus* Browne, 1974a: 66.

• *Eidophelus pygmaeolus* (Schedl, 1971d: 12) (*Cryphalomorphus*).

• *Eidophelus quadridens* (Browne, 1983c: 71) (*Hypothenemus*).

• *Eidophelus ramosus* (Beeson, 1935b: 115) (*Ptilopodius*).

• *Eidophelus rhododendri* Johnson in: Johnson et al. 2020a: 55 (RN).

= *Hemicryphalus squamosus* Bright, 1992: 189.

• *Eidophelus robustus* (Schedl, 1955c: 32) (*Cryphalomorphus*).

• *Eidophelus rugosus* (Schedl, 1943: 34) (*Ptilopodius*).

• *Eidophelus rusticus* (Wood, 1974a: 18) (*Cryphalomorphus*).

• *Eidophelus samoanus* Schedl, 1972i: 268.

• *Eidophelus schedli* Johnson in: Johnson et al. 2020a: 55 (RN).

= *Cryphalomorphus ceylonicus* Schedl, 1959i: 477.

• *Eidophelus semenovi* (Kurentsov, 1941: 231) (*Eocryphalus*).

• *Eidophelus separandus* (Schedl, 1965f: 56) (*Cryphalomorphus*).

• *Eidophelus setifer* (Wood, 1974a: 18) (*Cryphalomorphus*).

• *Eidophelus sodalis* (Schedl, 1965f: 55) (*Cryphalomorphus*).

• *Eidophelus spessivtzevi* (Berger, 1917: 243) (*Ernoporicus*).

• *Eidophelus spirostachius* (Schedl, 1958b: 557) (*Ptilopodius*).

• *Eidophelus splendens* (Schedl, 1975a: 350) (*Cryphalomorphus*).

• *Eidophelus squamatilis* (Schedl, 1977c: 500) (*Cryphalomorphus*).

• *Eidophelus squamosus* (Schedl, 1942c: 175) (*Lepicerinus*).

• *Eidophelus squamulosus* (Eggers, 1936a: 633) (*Cylindrotomicus*).

• *Eidophelus stephegynis* (Hopkins, 1915d: 11) (*Ptilopodius*).

• *Eidophelus sumatranus* Schedl, 1961f: 72.

• *Eidophelus takahashii* (Nobuchi, 1975: 43) (*Ernoporicus*).

• *Eidophelus tarawai* (Beaver, 1990b: 149) (*Ptilopodius*).

• *Eidophelus tonsus* (Schedl, 1969d: 49) (*Cryphalomorphus*).

• *Eidophelus tricolor* (Lea, 1910: 141) (*Cryphalus*).

• *Eidophelus trucis* (Wood, 1974a: 19) (*Cryphalomorphus*).

• *Eidophelus uncatus* (Schedl, 1971e: 373) (*Cryphalophilus*).

• *Eidophelus usagaricus* (Eggers, 1922c: 169) (*Neocryphalus*).

• *Eidophelus varius* (Schedl, 1975a: 350) (*Cryphalomorphus*).

• *Eidophelus venustus* (Schedl, 1953c: 78) (*Ptilopodius*).

• *Eidophelus yinae* Johnson in: Johnson et al. 2020a: 55 (RN).

= *Scolytogenes venustus* Yin, 2001: 328.

• *Eidophelus yunnanensis* (Yin, 2001: 324) (*Scolytogenes*).

• *Eidophelus zachvatkini* (Krivolutskaya, 1958: 149) (*Eocryphalus*).

#### *Ernoporus* Thomson

19 spp.

• *Ernoporus acanthopanaxi* (Niisima, 1913: 4) (*Cryphalus*).

• *Ernoporus armatus* (Browne, 1981a: 129) (*Euptilius*).

• *Ernoporus concentralis* Eggers, 1936a: 629.

• *Ernoporus corpulentus* (Sampson, 1919: 113) (*Cryphalus*).

= *Margadillius corpulentus sundri* Schedl, 1969d: 48.

• *Ernoporus dispar* (Schedl, 1972j: 49) (*Cryphalops*).

• *Ernoporus eggersi* Kurentsov, 1941: 142.

• *Ernoporus guibourtiae* (Schedl, 1957b: 53) (*Miocryphalus*).

• *Ernoporus imitatrix* (Schedl, 1977c: 499) (*Erioschidias*).

• *Ernoporus inermis* (Schedl, 1939b: 343) (*Stephanorhopalus*).

• *Ernoporus japonicus* Nobuchi, 1966: 52.

• *Ernoporus melodori* (Hopkins, 1915d: 36) (*Stephanorhopalus*).

• *Ernoporus minor* (Schedl, 1942b: 176) (*Margadillius*).

• *Ernoporus parvulus* (Eggers, 1943c: 75) (*Margadillius*).

= *Allothenemus minutus* Bright & Torres, 2006: 401.

= *Allothenemus exquisitus* Bright, 2019: 105.

• *Ernoporus quadridens* (Schedl, 1971b: 284) (*Cryphalops*).

• *Ernoporus shimanensis* Murayama, 1953b: 36.

• *Ernoporus squamosus* Buhroo & Zubair, 2024: 32.

• *Ernoporus thailandicus* (Schedl, 1967b: 127) (*Euptilius*).

• *Ernoporus tiliae* (Panzer, 1793: 14) (*Apate*).

= *Cryphalus ratzeburgi* Ferrari, 1867: 11.

= *Cryphalus lederi* Reitter, 1889d: 93.

= *Cryphalus starki* Eggers, 1942a: 31.

• *Ernoporus tuberculatus* (Browne, 1981a: 128) (*Euptilius*).

#### *Hemicryphalus* Schedl

3 spp.

• *Hemicryphalus argutus* (Wood, 1960a: 33) (*Eidophelus*).

• *Hemicryphalus atomus* (Wood, 1960a: 34) (*Eidophelus*).

• *Hemicryphalus incomptus* (Wood, 1960a: 32) (*Eidophelus*).

#### *Procryphalus* Hopkins

4 spp.

• *Procryphalus fraxini* (Berger, 1917: 238) (*Ernoporus*).

• *Procryphalus mucronatus* (LeConte, 1879: 518) (*Cryphalus*).

= *Procryphalus idahoensis* Hopkins, 1915d: 34.

= *Procryphalus populi* Hopkins, 1915d: 34.

• *Procryphalus petioli* (Beaver, 1990a: 281) (*Dryocoetiops*).

• *Procryphalus utahensis* Hopkins, 1915d: 33.

= *Procryphalus aceris* Hopkins, 1915d: 33.

= *Procryphalus salicis* Hopkins, 1915d: 33.

### HEXACOLINI Eichhoff

5 genera, 332 spp.

#### *Gymnochilus* Eichhoff

12 spp.

• *Gymnochilus alni* Wood, 1971a: 14.

• *Gymnochilus alternatus* (Eggers, 1943d: 345) (*Problechilus*).

• *Gymnochilus brevis* (Eggers, 1932d: 231) (*Problechilus*).

• *Gymnochilus consocius* (Blandford, 1897: 171) (*Problechilus*).

= *Problechilus trimaculatus* Schedl, 1935c: 91.

= *Problechilus novateutonicus* Schedl, 1936a: 105.

• *Gymnochilus glaber* (Schedl, 1951c: 87) (*Problechilus*).

= *Scolytodes schoenmanni* Wood, 2007: 297 (RN).

• *Gymnochilus insularis* (Eggers, 1932d: 230) (*Problechilus*).

• *Gymnochilus laevicollis* (Eggers, 1932d: 230) (*Problechilus*).

• *Gymnochilus minor* (Blandford, 1897: 172) (*Problechilus*).

= *Problechilus varius* Schedl, 1951c: 86.

• *Gymnochilus pilifer* (Eggers, 1932d: 229) (*Problechilus*).

• *Gymnochilus reitteri* Eichhoff, 1878b: 169.

= *Problechilus bicolor* Eggers, 1932d: 228.

= *Problechilus striatus* Eggers, 1932d: 227.

• *Gymnochilus vestitus* (Eggers, 1932d: 233) (*Problechilus*).

• *Gymnochilus zonatus* Eichhoff, 1868c: 399.

= *Meringopalpus fallax* Hagedorn, 1905a: 548.

= *Problechilus freyi* Schedl, 1966d: 103.

#### *Microborus* Blandford

14 spp.

• *Microborus aberrans* Wichmann, 1914c: 143.

= *Microborus setulosus* Eggers, 1933f: 19.

= *Microborus imitans* Eggers, 1940a: 131.

• *Microborus ambitus* Wood, 1969a: 13.

• *Microborus angustus* Jordal, 2017: 35.

• *Microborus bicolor* Eggers, 1933f: 19.

• *Microborus boops* Blandford, 1897: 175.

• *Microborus brevisetosus* Jordal, 2017: 37.

• *Microborus camerunus* (Eggers, 1920a: 236) (*Pseudocrypturgus*).

• *Microborus caymanensis* Bright, 2019: 187.

• *Microborus iviei* Bright, 2019: 187.

• *Microborus lautus* Wood, 1961c: 101.

• *Microborus lectus* Wood, 1971a: 17.

• *Microborus limatus* Wood, 1969a: 13.

• *Microborus mexicanus* Wood, 1981: 123.

• *Microborus rawlinsi* Bright, 2019: 189.

#### *Pseudohexacolus* Bright

1 sp.

• *Pseudohexacolus singularis* Bright, 2019: 190.

#### *Pycnarthrum* Eichhoff

18 spp.

• *Pycnarthrum amersum* Wood, 1983: 658.

• *Pycnarthrum bahiae* Wood, 2007: 245.

• *Pycnarthrum brosmii* Wood, 1971a: 14 (E).

• *Pycnarthrum carinatum* Wood, 1971a: 13.

• *Pycnarthrum fulgidum* Wood, 1977a: 217.

• *Pycnarthrum funerium* Wood, 1971a: 11.

• *Pycnarthrum hispidum* (Ferrari, 1867: 19) (*Hypoborus hispidus*).

= *Nemobius lambottei* Chapuis, 1869: 42.

= *Pycnarthrum gracile* Eichhoff, 1878b: 104.

= *Pycnarthrum quadraticolle* Eichhoff, 1878b: 106.

= *Pycnarthrum transversum* Blandford, 1897: 177.

= *Pycnarthrum reimoseri* Schedl, 1934c: 208.

• *Pycnarthrum inornatum* Wood, 1971a: 11.

• *Pycnarthrum insulare* Blair, 1933: 487.

• *Pycnarthrum kleinei* Eggers, 1951: 151.

• *Pycnarthrum lucidum* Wood, 1971a: 12.

• *Pycnarthrum pallidum* (Chapuis, 1869: 41) (*Nemobius pallidus*).

= *Pycnarthrum reticulatum* Schedl, 1940a: 355.

= *Pycnarthrum fici* Wood, 1971a: 11.

• *Pycnarthrum perditum* Wood, 1971a: 12.

• *Pycnarthrum pseudoinsulare* Kirkendall & Jordal, 2006: 731.

• *Pycnarthrum setulosum* Waterhouse, 1890: 553.

• *Pycnarthrum subcarinatum* Wood, 1971a: 13.

• *Pycnarthrum tuberculiferum* Wood, 2007: 243.

• *Pycnarthrum uniseriatum* Schedl, 1973a: 370.

#### *Scolytodes* Ferrari

287 spp.

• *Scolytodes abbreviatus* Jordal & Smith, 2020: 27.

• *Scolytodes acares* Wood, 1969a: 15.

• *Scolytodes acuminatus* Wood, 1969a: 17 (*Scolytodes cecropiavorus acuminatus*).

• *Scolytodes adustus* (Eggers, 1943d: 368) (*Hylocurosoma adustum*).

• *Scolytodes ageratinae* Wood, 2007: 273.

• *Scolytodes alni* Wood, 1969a: 20.

• *Scolytodes amictus* Jordal & Smith, 2020: 48.

• *Scolytodes amoenus* Wood, 1967c: 128.

• *Scolytodes anceps* Wood, 1981: 126.

• *Scolytodes angulus* Jordal & Kirkendall, 2019: 4.

• *Scolytodes animus* Jordal & Smith, 2020: 32.

• *Scolytodes anthracinus* Bright, 2019: 198.

• *Scolytodes aquilus* Bright, 2019: 198.

• *Scolytodes arcuatus* Jordal & Smith, 2020: 55.

• *Scolytodes aridus* Bright, 2019: 199.

• *Scolytodes asperatus* Jordal, 2018b: 89.

• *Scolytodes ater* (Eggers, 1943d: 365) (*Prionoscelis*).

= *Hylocurosoma elongatum* Eggers, 1943d: 369.

= *Scolytodes elongatissimus* Wood, 1988a: 32 (RN).

• *Scolytodes aterrimus* (Wood, 1988a: 32) (*Hylocurosoma*) (RN).

= *Hylocurosoma atrum* Eggers, 1943d: 371.

• *Scolytodes atlanticus* Bright & Torres, 2006: 396.

• *Scolytodes atomus* Bright, 2019: 200.

• *Scolytodes atratus* (Blandford, 1897: 178) (*Prionosceles*).

= *Prionosceles panamensis* Wood, 1961c: 103.

• *Scolytodes atrotibialis* (Eggers, 1943d: 365) (*Prionosceles*).

• *Scolytodes aureifrons* Jordal, 2018b: 84.

• *Scolytodes banosus* (Hagedorn, 1909: 743) (*Hexacolus*).

• *Scolytodes bellus* Bright, 2019: 201.

• *Scolytodes bicarinatus* Jordal, 2018b: 97.

• *Scolytodes bicolor* (Eggers, 1931b: 33) (*Hexacolus*).

• *Scolytodes bipilosus* Jordal, 2018b: 89.

• *Scolytodes bisetosus* Jordal & Smith, 2020: 35.

• *Scolytodes blandfordi* (Schedl, 1936a: 104) (*Hexacolus*).

• *Scolytodes boliviae* (Schedl, 1951c: 80) (*Hexacolus*).

• *Scolytodes bolivianus* Eggers, 1928a: 86.

= *Scolytodes aequipunctatus* Eggers, 1943d: 361.

• *Scolytodes bombycinus* Jordal & Smith, 2020: 33.

• *Scolytodes borealis* Jordal, 1998a: 56.

• *Scolytodes brasilianus* Wood, 1988a: 32 (RN).

= *Hexacolus brasiliensis* Schedl, 1935g: 274.

• *Scolytodes brasiliensis* (Eggers, 1928a: 89) (*Prionosceles*).

• *Scolytodes bruchi* (Hagedorn, 1909: 743) (*Hexacolus*).

• *Scolytodes callegarii* Petrov & Mandelshtam, 2007: 451.

• *Scolytodes callosus* Jordal & Kirkendall, 2019: 24.

• *Scolytodes canaliculus* Wood, 1977c: 517.

• *Scolytodes canalis* Wood, 1974a: 13.

= *Scolytodes amabilis* Wood, 1975a: 26.

• *Scolytodes cancellatus* Jordal & Smith, 2020: 25.

• *Scolytodes capillus* Jordal, 2018b: 92.

• *Scolytodes catinus* Jordal & Kirkendall, 2019: 16.

• *Scolytodes caudatus* Jordal, 1998a: 58.

• *Scolytodes cavus* Jordal, 2018b: 77.

• *Scolytodes cecropiavorus* Wood, 1969a: 17.

• *Scolytodes cecropicolens* Wood, 1969a: 16.

• *Scolytodes cecropii* (Schedl, 1937a: 66) (*Hexacolus*).

• *Scolytodes cedrelae* Wood, 1969a: 19.

• *Scolytodes cenchros* Jordal, 2013b: 534.

• *Scolytodes chaplini* Jordal & Smith, 2020: 20.

• *Scolytodes chapuisi* Wood, 1977a: 210 (RN).

= *Ctenophorus laevigatus* Chapuis, 1869: 49.

= *Hexacolus levis* Blackman, 1943c: 342.

• *Scolytodes chrysifrons* Jordal & Smith, 2020: 47.

• *Scolytodes circumsetosus* Jordal, 1998b: 412.

• *Scolytodes clusiacolens* Wood, 1967c: 121.

• *Scolytodes clusiae* Wood, 1969a: 14.

• *Scolytodes clusiaphilus* Jordal, 2013b: 533.

• *Scolytodes clusiapraelatus* Jordal, 2013b: 532.

• *Scolytodes clusiavorus* Wood, 1967c: 121.

• *Scolytodes cnesinoides* Jordal & Smith, 2020: 49.

• *Scolytodes columbianus* (Schedl, 1967a: 160) (*Hexacolus*).

• *Scolytodes comitabilis* Wood, 1978: 401.

• *Scolytodes comosus* Jordal & Kirkendall, 2019: 11.

• *Scolytodes concavifrons* Jordal, 2013b: 536.

• *Scolytodes concavus* Jordal, 1998b: 410.

• *Scolytodes confusus* (Eggers, 1943d: 370) (*Hylocurosoma confusum*).

• *Scolytodes conpunctus* Jordal, 2013b: 543.

• *Scolytodes constrictus* Wood, 1977c: 518.

• *Scolytodes contractus* Wood, 1977c: 518.

• *Scolytodes coronatus* Jordal & Smith, 2020: 60.

• *Scolytodes costabilis* Wood, 1974a: 13.

= *Scolytodes obesus* Wood, 1975a: 26.

• *Scolytodes crassus* Wood, 1971a: 16.

• *Scolytodes crinalis* Wood, 1978: 402.

• *Scolytodes criniger* Jordal & Smith, 2020: 37.

• *Scolytodes crinitus* Wood, 1978: 402.

• *Scolytodes cubensis* (Schedl, 1972f: 56) (*Hexacolus*).

• *Scolytodes culcitatus* (Blandford, 1897: 179) (*Epomadius*).

• *Scolytodes curvicostatus* Jordal, 2018b: 85.

• *Scolytodes declivistriatus* Schedl, 1967b: 126.

• *Scolytodes decorus* Wood, 1978: 402.

• *Scolytodes discedens* Eggers, 1943d: 360.

= *Scolytodes discriminatus* Wood, 1988a: 32 (RN).

• *Scolytodes dissimilis* Schedl, 1967d: 5.

• *Scolytodes echinus* Jordal & Smith, 2020: 53.

• *Scolytodes eggersi* Schedl, 1952e: 346 (RN).

= *Scolytodes bicolor* Eggers, 1943d: 363.

• *Scolytodes elongatus* Schedl, 1935g: 273.

• *Scolytodes erineophilus* Wood, 1969a: 20.

• *Scolytodes excavatus* Jordal, 2018b: 78.

• *Scolytodes exiguus* Wood, 1969a: 22.

• *Scolytodes facetus* Wood, 1967c: 126.

• *Scolytodes festus* Wood, 1977c: 519.

• *Scolytodes ficicolens* Wood, 1981: 126.

• *Scolytodes ficivorus* Wood, 1967c: 125.

• *Scolytodes fimbriatus* Jordal & Kirkendall, 2019: 18.

• *Scolytodes flavifrons* Jordal, 2018b: 90.

• *Scolytodes fortis* Jordal & Smith, 2020: 44.

• *Scolytodes fraterniatratus* Jordal, 2013b: 545.

• *Scolytodes frontocarinatus* Jordal, 2013b: 537.

• *Scolytodes frontoglabratus* (Schedl, 1935g: 274) (*Hexacolus*).

• *Scolytodes fulmineus* Wood, 1977a: 217.

• *Scolytodes fulvus* Jordal, 2013b: 547.

• *Scolytodes genialis* Wood, 1975a: 27.

• *Scolytodes gennaeus* Wood, 1988a: 33 (RN).

= *Scolytodes genialis* Wood, 1978: 403.

• *Scolytodes glaber* (Eichhoff, 1868c: 400) (*Hexacolus*).

= *Erineophilus schwarzi* Hopkins, 1902a: 36.

• *Scolytodes glabratus* (Schedl, 1954b: 23) (*Hexacolus*).

• *Scolytodes glabrellus* (Schedl, 1954b: 21) (*Hexacolus*).

• *Scolytodes glabrescens* Wood, 1971c: 152 (RN).

= *Prionosceles glaber* Wood, 1961c: 102.

• *Scolytodes gracilis* Schedl, 1976: 61.

= *Hexacolus laevigatus* Schedl, 1962d: 98.

= *Scolytodes laevigatulus* Wood, 1988a: 33 (RN).

• *Scolytodes grandis* (Schedl, 1962d: 100) (*Hexacolus*).

= *Scolytodes glaber* Eggers, 1943d: 360.

= *Scolytodes glaberrimus* Wood, 1971c: 152 (RN).

• *Scolytodes granulatus* Jordal, 2018b: 88.

• *Scolytodes grossepunctatus* Jordal, 2013b: 541.

• *Scolytodes gunnerae* Wood, 2007: 282.

• *Scolytodes guyanaensis* (Schedl, 1937d: 13) (*Erineophilus*).

= *Hexacolus nardus* Schedl, 1976: 62.

• *Scolytodes habilis* Wood, 1978: 403.

• *Scolytodes hagedorni* (Schedl, 1962d: 101) (*Prionosceles*).

• *Scolytodes hirsutus* Wood, 1971a: 16.

• *Scolytodes hondurensis* Jordal, 1998a: 44.

• *Scolytodes horridus* Jordal & Smith, 2020: 36.

• *Scolytodes immanis* Wood, 1969a: 19.

• *Scolytodes impressus* Wood, 1969a: 22.

• *Scolytodes ingae* (Blackman, 1943d: 383) (*Prionosceles*).

= *Scolytodes trigonus* Jordal, 2013b: 546.

• *Scolytodes ingavorus* Wood, 1967c: 126.

• *Scolytodes inordinatus* Jordal & Smith, 2020: 23.

• *Scolytodes interpunctatus* (Eggers, 1943d: 366) (*Prionosceles*).

• *Scolytodes inusitatus* Jordal, 2013b: 540.

• *Scolytodes irazuensis* Wood, 1969a: 15.

• *Scolytodes iviei* Bright, 2019: 204.

• *Scolytodes johnsoni* Jordal, 2018b: 93.

• *Scolytodes jubatus* Jordal & Smith, 2020: 27.

• *Scolytodes jucundus* Wood, 1977c: 519.

• *Scolytodes laevicorpus* Wood, 1988a: 33 (RN).

= *Hylocurosoma laevae* Eggers, 1943d: 367.

• *Scolytodes laevigatus* Ferrari, 1867: 77.

• *Scolytodes laevis* (Eggers, 1928a: 88) (*Prionosceles*).

• *Scolytodes lapillus* Jordal & Smith, 2020: 58.

• *Scolytodes latipes* Jordal & Smith, 2020: 16.

• *Scolytodes lepidus* Wood, 1975a: 27.

• *Scolytodes libidus* Wood, 1978: 404.

• *Scolytodes limbatus* (Eggers, 1943d: 370) (*Hylocurosoma limbatum*).

• *Scolytodes longipennis* Eggers, 1943d: 363.

• *Scolytodes longipilus* Jordal, 2018b: 95.

• *Scolytodes longisetum* Bright, 2019: 206.

• *Scolytodes lubricus* Jordal & Smith, 2020: 22.

• *Scolytodes maestus* Jordal & Smith, 2020: 51.

• *Scolytodes major* (Eggers, 1928a: 86) (*Prionosceles*).

= *Prionosceles argentinensis* Eggers, 1928a: 87.

= *Prionosceles bolivianus* Eggers, 1928a: 88.

= *Prionosceles similis* Eggers, 1928a: 86.

= *Scolytodes boliviensis* Wood, 1988a: 32 (RN).

• *Scolytodes majulus* Wood, 2007: 273 (RN) (E).

= *Scolytodes major* Eggers, 1943d: 361.

= *Scolytodes majus* Wood, 1988a: 33 (RN).

• *Scolytodes marginatus* Wood, 1969a: 21.

• *Scolytodes maurus* (Blandford, 1897: 178) (*Prionosceles*).

= *Prionosceles medius* Eggers, 1928a: 89.

= *Hexacolus ellipticus* Eggers, 1934a: 80.

• *Scolytodes medialis* Wood, 1988a: 33 (RN).

= *Scolytodes medius* Eggers, 1943d: 359.

• *Scolytodes melanocephalus* (Blandford, 1897: 181) (*Hexacolus*).

• *Scolytodes micidus* Wood, 1971a: 17.

• *Scolytodes minimus* Jordal, 2018b: 80.

• *Scolytodes minor* (Eggers, 1928a: 89) (*Prionosceles*).

• *Scolytodes minutissimus* Schedl, 1952e: 355.

= *Hexacolinus minutissimus* Schedl, 1963f: 217.

• *Scolytodes minutus* Wood, 1981: 122 (RN).

= *Hexacolus minutissimus* Schedl, 1978a: 297.

• *Scolytodes montanus* Jordal, 1998b: 413.

• *Scolytodes monticola* Jordal, 2018b: 83.

• *Scolytodes morulus* (Schedl, 1952e: 356) (*Hexacolus*).

• *Scolytodes multistriatus* (Wood, 1961c: 97) (*Hexacolus*).

• *Scolytodes mundus* Jordal & Kirkendall, 2019: 23.

• *Scolytodes naevius* Wood, 1981: 127.

• *Scolytodes nanellus* Wood, 1967c: 124.

• *Scolytodes nayaritensis* Wood, 2007: 291.

• *Scolytodes niger* Jordal & Kirkendall, 2019: 6.

• *Scolytodes nitellus* (Schedl, 1954b: 22) (*Hexacolus*).

• *Scolytodes nitens* (Schedl, 1954b: 23) (*Hexacolus*).

• *Scolytodes nitidissimus* (Eggers, 1940a: 135) (*Hexacolus*).

= *Prionosceles imitans* Eggers, 1940a: 136.

• *Scolytodes nitidus* (Eggers, 1928a: 88) (*Prionosceles*).

• *Scolytodes notatus* (Eggers, 1940a: 133) (*Hexacolus*).

= *Hexacolus discedens* Eggers, 1940a: 133.

= *Hexacolus insularis* Schedl, 1952e: 357.

• *Scolytodes nudifrons* Jordal, 1998b: 414.

• *Scolytodes oblongus* (Eggers, 1940a: 134) (*Hexacolus*).

• *Scolytodes obovatus* Jordal, 2013b: 539.

• *Scolytodes obscurus* (Wood, 1961c: 100) (*Hexacolus*).

• *Scolytodes obtusiceps* Bright, 2019: 210.

• *Scolytodes ochromae* Wood, 1969a: 19.

• *Scolytodes ommateus* Wood, 1971a: 15.

• *Scolytodes opacicollis* (Eggers, 1928a: 90) (*Prionosceles*).

• *Scolytodes opacus* Wood, 1977c: 520.

= *Scolytodes opimus* Wood, 1977c: 520.

• *Scolytodes otongae* Jordal & Smith, 2020: 19.

• *Scolytodes pacificus* Jordal, 1998a: 62.

• *Scolytodes pannuceus* Wood, 1971a: 16.

• *Scolytodes parallelus* (Schedl, 1962d: 99) (*Hexacolus*).

• *Scolytodes parvipilus* Jordal & Kirkendall, 2019: 27.

• *Scolytodes parvulus* Wood, 1969a: 14.

• *Scolytodes parvus* (Eggers, 1943d: 371) (*Hylocurosoma parvum*).

• *Scolytodes pascopomus* Jordal, 2013b: 534.

• *Scolytodes peckorum* Bright, 2019: 211.

• *Scolytodes pelicipennis* (Schedl, 1952e: 356) (*Hexacolus*).

• *Scolytodes peniculus* Jordal & Smith, 2020: 45.

• *Scolytodes perditus* Wood, 1967c: 123.

• *Scolytodes permagnus* (Eggers, 1943d: 364) (*Prionosceles*).

• *Scolytodes perplexus* Schedl, 1972f: 56.

• *Scolytodes perpusillus* Wood, 1978: 404.

• *Scolytodes peruanus* Wood, 2007: 274 (E).

• *Scolytodes phoebeae* Wood, 1969a: 16.

• *Scolytodes piceus* (Blandford, 1897: 183) (*Hexacolus*).

• *Scolytodes pilifer* Wood, 1982: 230.

• *Scolytodes pilifrons* (Schedl, 1962d: 100) (*Hexacolus*).

• *Scolytodes piliscapus* Jordal, 2018b: 84.

• *Scolytodes pilosulus* (Eggers, 1932d: 234) (*Problechilus*).

• *Scolytodes planifrons* Jordal & Kirkendall, 2019: 20.

• *Scolytodes plenus* Jordal & Kirkendall, 2019: 28.

• *Scolytodes plesiopolitus* Bright, 2019: 211.

• *Scolytodes plumeriae* Wood, 1969a: 17.

• *Scolytodes plumericolens* Wood, 1983: 658.

• *Scolytodes politus* Bright, 2019: 212.

• *Scolytodes porosus* Jordal & Kirkendall, 2019: 22.

• *Scolytodes potens* Jordal, 2018b: 79.

• *Scolytodes praeceps* Wood, 1977c: 521.

= *Scolytodes tardus* Wood, 1981: 127.

• *Scolytodes profundus* Jordal & Kirkendall, 2019: 15.

• *Scolytodes projectus* Jordal & Smith, 2020: 22.

• *Scolytodes prolatus* Jordal, 2018b: 95.

• *Scolytodes proximus* Wood, 1967c: 127.

• *Scolytodes pseudoacuminatus* (Schedl, 1935a: 51) (*Hexacolus*).

• *Scolytodes pseudoanimus* Jordal & Smith, 2020: 32.

• *Scolytodes pseudoatratus* Jordal & Smith, 2020: 16.

• *Scolytodes pseudobicolor* (Eggers, 1940a: 132) (*Hexacolus*).

= *Hexacolus ovalis* Eggers, 1940a: 132.

= *Hexacolus subparallelus* Eggers, 1940a: 134.

= *Hexacolus longicollis* Eggers, 1951: 152.

= *Hexacolus pelicerinus* Schedl, 1952e: 358.

• *Scolytodes pseudocrassus* Jordal & Smith, 2020: 39.

• *Scolytodes pseudolepidus* Jordal & Smith, 2020: 41.

• *Scolytodes pseudopiceus* Wood, 1969a: 18.

• *Scolytodes pubescens* Wood, 1969a: 21.

• *Scolytodes puer* (Schedl, 1952e: 359) (*Hexacolinus*).

• *Scolytodes puertoricensis* Bright & Torres, 2006: 398.

• *Scolytodes pumilus* Wood, 1969a: 23.

• *Scolytodes punctatus* (Eggers, 1943d: 368) (*Hylocurosoma punctatum*).

• *Scolytodes punctifer* Wood, 1969a: 18 (*Scolytodes cecropiavorus punctifer*).

• *Scolytodes punctiferus* Wood, 1976: 348 (RN).

= *Scolytodes punctifer* Wood, 1971a: 15.

• *Scolytodes punctifrons* Jordal, 1998b: 415.

• *Scolytodes pusillimus* Wood, 1981: 127.

• *Scolytodes pusillus* (Eggers, 1943d: 367) (*Hylocurosoma pusillum*).

• *Scolytodes radiatus* Wood, 1977a: 218.

• *Scolytodes reticulatus* (Wood, 1961c: 98) (*Hexacolus*).

• *Scolytodes retifer* Wood, 1983: 658.

• *Scolytodes rufifrons* Jordal & Smith, 2020: 54.

• *Scolytodes rufus* Jordal, 2018b: 92.

• *Scolytodes rugicollis* (Schedl, 1940c: 205) (*Hexacolus*).

= *Scolytodes plicatus* Wood, 1969a: 21.

• *Scolytodes rugulosus* Eggers, 1943d: 362.

• *Scolytodes sabaensis* Bright, 2019: 215.

• *Scolytodes sagittarius* Jordal, 2013b: 548.

• *Scolytodes samamae* Jordal & Smith, 2020: 19.

• *Scolytodes semicrassus* Jordal & Smith, 2020: 39.

• *Scolytodes semilepidus* Jordal & Smith, 2020: 42.

• *Scolytodes semipunctatus* Wood, 1978: 405.

• *Scolytodes serenus* Wood, 1977c: 521.

• *Scolytodes seriatus* Jordal & Kirkendall, 2019: 14.

• *Scolytodes setosicauda* Jordal, 2018b: 83.

• *Scolytodes setosus* (Blandford, 1897: 181) (*Hexacolus*).

• *Scolytodes simplex* Jordal & Kirkendall, 2019: 7.

• *Scolytodes sloanae* Jordal & Smith, 2020: 17.

• *Scolytodes solarius* Jordal, 2013b: 543.

• *Scolytodes spadix* (Blackman, 1943d: 384) (*Prionosceles*).

• *Scolytodes sparsepilosus* Wood, 2007: 287 (E).

• *Scolytodes sparsus* Jordal & Smith, 2020: 56.

• *Scolytodes spatulatus* Jordal & Kirkendall, 2019: 12.

• *Scolytodes speculofrons* Jordal, 2018b: 79.

• *Scolytodes squamatifrons* Jordal & Kirkendall, 2019: 10.

• *Scolytodes steineri* Bright, 2019: 215.

• *Scolytodes stramineus* Jordal & Smith, 2020: 29.

• *Scolytodes striatulus* Wood, 1979: 136 (RN).

= *Hylocurosoma striatum* Eggers, 1940a: 139.

• *Scolytodes striatus* (Eggers, 1934a: 79) (*Hexacolus*).

• *Scolytodes subcribrosus* (Eggers, 1933f: 17) (*Hexacolus*).

• *Scolytodes sufflatus* Jordal & Kirkendall, 2019: 8.

• *Scolytodes sulcifrons* Jordal & Kirkendall, 2019: 19.

• *Scolytodes sus* Jordal, 2013b: 539.

• *Scolytodes suspectus* Jordal, 1998a: 60.

• *Scolytodes suturalis* Wood, 1977c: 521.

• *Scolytodes swieteniae* (Blackman, 1943d: 381) (*Hexacolus*).

• *Scolytodes tenuis* (Wood, 1961c: 99) (*Hexacolus*).

• *Scolytodes teres* Jordal & Smith, 2020: 30.

• *Scolytodes tolimanus* (Schedl, 1962d: 102) (*Prionosceles*).

• *Scolytodes torresi* Bright, 2019: 218.

• *Scolytodes triangulus* Jordal, 1998b: 416.

• *Scolytodes trispinosus* Eggers, 1934a: 80.

• *Scolytodes tristis* Jordal & Smith, 2020: 47.

• *Scolytodes tucumani* Wood, 2007: 274.

• *Scolytodes ungulatus* Jordal, 1998b: 417.

• *Scolytodes unipunctatus* (Blandford, 1897: 182) (*Hexacolus*).

= *Scolytodes cylindricus* Schedl, 1978a: 297.

• *Scolytodes uniseriatus* Jordal, 2013b: 530.

• *Scolytodes validus* Jordal & Smith, 2020: 55.

• *Scolytodes varius* Wood, 1977c: 522.

• *Scolytodes vellus* Jordal, 2018b: 88.

• *Scolytodes venustulus* Wood, 1967c: 124.

• *Scolytodes venustus* Wood, 1969a: 18.

• *Scolytodes vesculus* Wood, 1981: 128.

• *Scolytodes vicinus* (Eggers, 1928a: 87) (*Prionosceles*).

= *Prionosceles dubiosus* Schedl, 1972f: 55.

• *Scolytodes vietus* Jordal & Smith, 2020: 51.

• *Scolytodes virgatus* Jordal & Smith, 2020: 36.

• *Scolytodes volcanus* Wood, 1969a: 14.

### HYLASTINI LeConte

4 genera, 55 spp. (1 sp. with 2 sspp.).

#### *Hylastes* Erichson

32 spp. (1 sp. with 2 sspp.).

• *Hylastes ambiguus* Blandford, 1894c: 57.

• *Hylastes angustatus* (Herbst, 1793: 111) (*Bostrichus*).

= *Hylesinus graphus* Duftschmid, 1825: 106.

= *Hylastes scandinavicus* Lekander, 1965: 199.

• *Hylastes asperatus* Wood, 1975a: 24.

• *Hylastes ater* Erichson, 1836: 47.

= *Bostrichus ater* Paykull, 1800: 153.

= *Ips niger* Marsham, 1802: 59.

= *Hylesinus chloropus* Duftschmid, 1825: 102.

= *Tomicus pinicola* Bedel, 1888: 390 (RN).

= *Hylastes angusticollis* Eggers, 1929c: 9.

= *Ipsocossonus anomalus* Oke, 1934: 251.

• *Hylastes attenuatus* Erichson, 1836: 50.

• *Hylastes batnensis anatolicus* Knížek & Pfeffer in: Pfeffer 1995: 74.

• *Hylastes batnensis batnensis* Brisout de Barneville, 1883: 146 (*Hylastes batnensis*).

• *Hylastes brunneus* Erichson, 1836: 48.

= *Hylastes ater rotundicollis* Reitter, 1895: 60.

= *Hylastes aterrimus* Eggers, 1933a: 3.

• *Hylastes cunicularius* Erichson, 1836: 49.

= *Hylurgops starki* Eggers, 1933a: 1.

• *Hylastes exilis* Chapuis, 1869: 20.

• *Hylastes fallax* Wichmann, 1911a: 100.

= *Hylastes gergeri* Eggers, 1911b: 119.

• *Hylastes flohri* (Eggers, 1930a: 166) (*Hylurgops*).

• *Hylastes fulgidus* Blackman, 1941: 18.

• *Hylastes gracilis* LeConte, 1868: 174.

= *Hylastes vastans* Chapuis, 1869: 17.

= *Hylastes longus* LeConte, 1876: 388.

= *Hylastes asper* Swaine, 1917: 19.

= *Hylastes nitidus* Swaine, 1917: 19.

• *Hylastes linearis* Erichson, 1836: 49.

= *Hylastes corticiperda* Erichson, 1836: 50.

= *Hylastes variolosus* Perris, 1852: 181.

= *Hylastes clavus* Wollaston, 1854: 305.

= *Hylastes flavicornis* Lindberg, 1950: 19.

• *Hylastes longicollis* Swaine, 1918: 79.

• *Hylastes lowei* Paiva, 1861: 211.

• *Hylastes macer* LeConte, 1868: 175.

• *Hylastes mexicanus* Wood, 1967a: 36.

• *Hylastes nigrinus* (Mannerheim, 1852: 356) (*Hylurgus*).

= *Hylastes yukonis* Fall, 1926: 207.

• *Hylastes opacus* Erichson, 1836: 51.

= *Hylesinus angustatus* Gyllenhal, 1813: 342.

= *Hylastes simplex* Rey, 1892: 30.

• *Hylastes parallelus* Chapuis in: Chapuis and Eichhoff 1875: 196.

• *Hylastes plumbeus* Blandford, 1894c: 57 (RN).

= *Hylastes obscurus* Chapuis in: Chapuis and Eichhoff 1876: 197.

= *Hylastes septentrionalis* Eggers, 1923a: 135.

= *Hylurgops fushunensis* Murayama, 1940: 235.

• *Hylastes porculus* Erichson, 1836: 49.

= *Hylastes carbonarius* Fitch, 1858: 730.

= *Hylurgus cavernosus* Zimmermann, 1868: 149.

= *Hylastes granosus* Chapuis, 1869: 17.

= *Hylastes scaber* Swaine, 1917: 18.

= *Hylastes swainei* Eggers, 1934b: 25.

= *Hylastes canadensis* Blackman, 1941: 15.

= *Hylastes webbi* Blackman, 1941: 10.

• *Hylastes retifer* Wood, 1982: 227.

• *Hylastes ruber* Swaine, 1915b: 367.

• *Hylastes salebrosus* Eichhoff, 1868b: 146.

= *Hylurgus scabripennis* Zimmermann, 1868: 149.

= *Hylastes scobinosus* Eichhoff, 1868b: 146.

• *Hylastes subopacus* Blackman, 1941: 19.

• *Hylastes substriatus* Strohmeyer, 1914a: 7.

• *Hylastes suspectus* Bright, 1972a: 31.

• *Hylastes techangensis* Tsai & Huang, 1964a: 233.

• *Hylastes tenuis* Eichhoff, 1868b: 147.

= *Hylastes criticus* Eichhoff, 1868b: 147.

= *Hylastes minutus* Blackman, 1941: 25.

= *Hylastes parvus* Blackman, 1941: 24.

= *Hylastes pusillus* Blackman, 1941: 23.

• *Hylastes woodi* Johnson **nom. nov**. (RN).

= *Hylastes niger* Wood, 1974a: 7.

#### *Hylurgops* LeConte

20 spp.

• *Hylurgops bonvouloiri* (Chapuis, 1869: 22) (*Hylastes*).

• *Hylurgops eusulcatus* Tsai & Huang, 1964b: 239.

• *Hylurgops glabratus* (Zetterstedt, 1828: 343) (*Hylurgus*).

= *Hylesinus paykullii* Duftschmid, 1825: 98.

= *Hylesinus decumanus* Erichson, 1836: 51.

= *Hylesinus tenebrosus* Sahlberg, 1836b: 139.

• *Hylurgops incomptus* (Blandford, 1897: 145) (*Hylastes*).

= *Hylurgops grandicollis* Swaine, 1917: 17.

• *Hylurgops inouyei* Nobuchi, 1959a: 10.

• *Hylurgops interstitialis* (Chapuis in: Chapuis and Eichhoff 1876: 196) (*Hylas­tes*).

= *Hylastes imitator* Reitter, 1900: 59.

= *Hylurgops nipponicus* Murayama, 1936: 123.

• *Hylurgops junnanicus* Sokanovskiy, 1959a: 93.

• *Hylurgops knausi* Swaine, 1917: 17.

• *Hylurgops longipennis* (Blandford, 1896d: 143) (*Hylastes*).

• *Hylurgops longipillus* (Reitter, 1895: 63) (*Hylastes*).

= *Hylurgops transbaicalicus* Eggers, 1941d: 119.

= *Hylurgops likiangensis* Tsai & Huang, 1964b: 237.

• *Hylurgops major* Eggers, 1944b: 67.

• *Hylurgops palliatus* (Gyllenhal, 1813: 340) (*Hylesinus*).

= *Ips piceus* Marsham, 1802: 58.

= *Ips rufus* Marsham, 1802: 57.

= *Bostrichus abietiperda* Bechstein, 1818: 187.

= *Hylesinus fuscus* Duftschmid, 1825: 105.

= *Hylesinus marginatus* Duftschmid, 1825: 104.

= *Hylurgus rufescens* Stephens, 1830: 364.

= *Hylurgus helferi* Villa & Villa, 1835: 49.

= *Hylurgops parvus* Eggers, 1933a: 2.

• *Hylurgops pinifex* (Fitch, 1858: 729) (*Hylastes*).

• *Hylurgops planirostris* (Chapuis, 1869: 21) (*Hylastes*).

• *Hylurgops porosus* (LeConte, 1868: 175) (*Hylastes*).

= *Hylurgops lecontei* Swaine, 1917: 16.

• *Hylurgops reticulatus* Wood, 1971b: 71.

• *Hylurgops rugipennis* (Mannerheim, 1843: 297) (*Hylastes*).

• *Hylurgops spessivtsevi* Eggers, 1914b: 187 (E).

= *Hylurgops modestus* Murayama, 1937: 367.

= *Hylurgops squamosus* Murayama, 1942: 56.

• *Hylurgops sulcatus* Eggers, 1933d: 97.

• *Hylurgops tuberculatus* Eggers, 1933d: 98.

#### *Pachysquamus* Mercado & Negron

1 sp.

• *Pachysquamus subcostulatus* (Mannerheim, 1853: 239) (*Hylastes*).

= *Hylastes cristatus* Mannerheim, 1853: 239.

= *Hylastes alternans* Chapuis, 1869: 22.

#### *Scierus* LeConte

2 spp.

• *Scierus annectens* LeConte, 1876: 390.

• *Scierus pubescens* Swaine, 1924d: 287.

### HYLESININI Erichson

16 genera, 161 spp.

#### *Alniphagus* Swaine

3 spp.

• *Alniphagus aspericollis* (LeConte, 1876: 380) (*Hylesinus*).

• *Alniphagus costatus* (Blandford, 1894c: 63) (*Hylesinus*).

= *Hylastes alni* Niisima, 1909: 137.

= *Alniphagus alni imitator* Sokanovskiy, 1958: 38.

• *Alniphagus hirsutus* Schedl, 1948d: 236.

#### *Chilodendron* Schedl

1 sp.

• *Chilodendron planicolle* Schedl, 1953c: 74.

#### *Cryptocurus* Schedl

1 sp.

• *Cryptocurus spinipennis* Schedl, 1958a: 870.

= *Hyloperus bicornis* Browne, 1970: 546.

= *Hyloperus caudatus* Browne, 1970: 547.

#### *Dactylipalpus* Chapuis

11 spp.

• *Dactylipalpus africanus* Eggers, 1920a: 229.

• *Dactylipalpus biseriatus* Browne, 1973: 282.

• *Dactylipalpus camerunus* Hagedorn, 1908: 371.

• *Dactylipalpus cicatricosus* (Blandford, 1896c: 321) (*Ethadopselaphus*).

= *Dactylipalpus marmoratus* Strohmeyer, 1914b: 73.

• *Dactylipalpus floccosus* Hagedorn, 1912a: 351.

• *Dactylipalpus grouvellei* (Blandford, 1896c: 322) (*Ethadopselaphus*).

= *Dactylipalpus similis* Hagedorn, 1908: 370.

• *Dactylipalpus imitans* Eggers, 1922c: 164.

• *Dactylipalpus orientalis* Eggers, 1933e: 16.

• *Dactylipalpus parricida* Eggers, 1920a: 230.

• *Dactylipalpus transversus* Chapuis, 1869: 12.

= *Dactylipalpus quadraticollis* Chapuis, 1869: 12.

• *Dactylipalpus unctus* Wood, 1961b: 8.

= *Dactylipalpus niger* Schedl, 1962e: 87.

#### *Ficicis* Lea

13 spp.

• *Ficicis bakeri* (Sampson, 1921: 27) (*Hylesinus*).

= *Hylesinus sumatranus* Eggers, 1923a: 134.

• *Ficicis brevipilosus* (Schedl, 1942c: 167) (*Hylesinus*).

• *Ficicis despectus* (Walker, 1859: 261) (*Hylesinus*).

= *Hylesinus granulifer* Motschulsky, 1863: 516.

= *Hylesinus scobipennis* Chapuis, 1869: 30.

= *Hylesinus similis* Eggers, 1923b: 136.

= *Hylesinus samoanus* Schedl, 1951f: 142.

• *Ficicis elongatus* (Schedl, 1942c: 167) (*Hylesinus*).

• *Ficicis javanus* (Eggers, 1923b: 135) (*Hylesinus*).

• *Ficicis maculipennis* (Schedl, 1975h: 217) (*Hylesinus*).

• *Ficicis pacificus* (Beeson, 1929: 221) (*Hylesinus*).

= *Hylesinus pellitus* Schedl, 1955e: 288.

• *Ficicis pertinax* (Schedl, 1975h: 217) (*Hylesinus*).

• *Ficicis porcatus* (Chapuis, 1869: 31) (*Hylesinus*).

= *Hylesinus koebeli* Lea, 1910: 148.

= *Hylesinus philippinensis* Eggers, 1923b: 137.

= *Hylesinus subcostatus* Eggers, 1923b: 137.

= *Hylesinus crassus* Beeson, 1929: 220.

= *Hylesinus subopacus* Eggers, 1930c: 10.

= *Hylesinus insularum* Beeson, 1940: 192.

= *Phloeosinus goliathoides* Murayama, 1955: 94.

• *Ficicis robustus* (Eggers, 1939c: 223) (*Hylesinus*).

• *Ficicis sulcinodis* (Schedl, 1974a: 458) (*Hylesinus*).

• *Ficicis varians* Lea, 1910: 147.

• *Ficicis wallacei* (Blandford, 1896e: 197) (*Hylesinus*).

#### *Hapalogenius* Hagedorn

31 spp.

• *Hapalogenius acaciae* Schedl, 1957b: 18.

• *Hapalogenius africanus* (Eggers, 1933e: 19) (*Pseudophloeotribus*).

= *Hapalogenius lesnei* Eggers, 1943c: 73.

= *Metahylesinus brincki* Schedl, 1957a: 323.

= *Xylechinus uniformis* Schedl, 1982: 281.

• *Hapalogenius angolensis* (Schedl, 1982: 284) (*Aridiamerus*).

= *Hylesinopsis angolanus* Wood, 1988a: 32 (RN).

• *Hapalogenius atakorae* (Schedl, 1951b: 1105) (*Rhopalopselion*).

• *Hapalogenius baphiae* (Schedl, 1954d: 75) (*Glochicopterus*).

• *Hapalogenius dimorphus* (Schedl, 1937g: 16) (*Metahylesinus*).

• *Hapalogenius endroedyi* (Schedl, 1967c: 224) (*Hemihylesinus*).

• *Hapalogenius fuscipennis* (Chapuis, 1869: 44) (*Phloeotribus*).

= *Hapalogenius globosus* Hagedorn, 1912a: 352.

= *Hapalogenius bimaculatus* Eggers, 1933f: 22.

= *Hapalogenius congonus* Schedl, 1950g: 206.

= *Glochiphorus alienus* Schedl, 1982: 283.

• *Hapalogenius hispidus* (Eggers, 1935e: 299) (*Metahylesinus*).

• *Hapalogenius horridus* (Eggers, 1924: 99) (*Hylesinus*).

• *Hapalogenius immaturus* Schedl, 1958a: 867.

• *Hapalogenius kenyae* (Wood, 1986a: 268) (*Hylesinopsis*) (RN).

= *Alniphagus africanus* Schedl, 1963b: 259.

= *Hylesinopsis acaciacolens* Wood, 1987: 547 (RN).

• *Hapalogenius lonchocarpae* Schedl, 1959a: 707.

• *Hapalogenius maculatus* Schedl, 1957b: 19.

• *Hapalogenius oblongus* (Eggers, 1935e: 299) (*Pseudophloeotribus*).

= *Metahylesinus striatus* Schedl, 1958a: 865.

• *Hapalogenius occidentalis* Schedl, 1967c: 225.

• *Hapalogenius orientalis* (Eggers, 1943c: 72) (*Metahylesinus*).

• *Hapalogenius primus* Schedl, 1971d: 6.

• *Hapalogenius pusillus* (Gerstäcker, 1855: 639) (*Hylesinus*).

• *Hapalogenius quadrituberculatus* (Schedl, 1957b: 24) (*Metahylesinus*).

• *Hapalogenius rhodesianus* (Eggers, 1933e: 19) (*Pseudophloeotribus*).

• *Hapalogenius rufus* Schedl, 1958a: 866.

• *Hapalogenius senegambiensis* Schedl, 1941c: 382.

• *Hapalogenius seriatus* (Eggers, 1940e: 228) (*Pseudophloeotribus*).

= *Hapalogenius cinchonae* Schedl, 1952d: 8.

• *Hapalogenius squamosus* (Eggers, 1936b: 28) (*Pseudophloeotribus*).

• *Hapalogenius subseriatus* Schedl, 1958a: 866.

• *Hapalogenius sulcatus* (Eggers, 1944c: 93) (*Metahylesinus*).

• *Hapalogenius suturalis* Schedl, 1965e: 6.

• *Hapalogenius togonus* (Eggers, 1920a: 234) (*Pseudohylesinus*).

• *Hapalogenius ugandae* (Wood, 1986a: 268) (*Hylesinopsis*) (RN).

= *Hylesinus africanus* Schedl, 1965e: 4.

= *Hylesinopsis secutus* Wood, 1987: 547 (RN).

• *Hapalogenius variegatus* (Eggers, 1936b: 29) (*Pseudophloeotribus*).

#### *Hylastinus* Bedel

5 spp.

• *Hylastinus achillei* Reitter, 1895: 54.

• *Hylastinus fankhauseri* Reitter, 1895: 54 (*Hylastinus trifolii fankhauseri*).

• *Hylastinus kroaticus* Fuchs, 1912: 39.

• *Hylastinus obscurus* (Marsham, 1802: 57) (*Ips*).

= *Scolytus crenatus* Olivier, 1800a: 12.

= *Dermestes trifolii* Müller, 1803: 47.

= *Hylesinus crenatulus* Duftschmid, 1825: 104.

= *Hylurgus fuscescens* Stephens, 1830: 365.

= *Hylurgus piceus* Stephens, 1830: 365.

= *Hylastinus pilosus* Eggers, 1944a: 140.

• *Hylastinus tiliae* Semenov, 1902: 271.

#### *Hylesinopsis* Eggers

16 spp.

• *Hylesinopsis alluaudi* (Lepesme, 1942c: 207) (*Kissophagus*).

• *Hylesinopsis angolensis* Schedl, 1959h: 24.

• *Hylesinopsis arabiae* Schedl, 1975f: 353.

• *Hylesinopsis ater* (Nunberg, 1967: 315) (*Trypographus*).

• *Hylesinopsis confusa* (Eggers, 1935e: 300) (*Kissophagus confusus*).

• *Hylesinopsis decellei* (Nunberg, 1969: 394) (*Trypographus*).

• *Hylesinopsis dubia* Eggers, 1920c: 40 (E).

• *Hylesinopsis emarginata* (Nunberg, 1973: 6) (*Trypographus emarginatus*).

• *Hylesinopsis fasciata* (Hagedorn, 1909: 737) (*Kissophagus fasciatus*).

= *Kissophagus punctatus* Eggers, 1932b: 28.

• *Hylesinopsis ficus* (Schedl, 1954a: 878) (*Kissophagus*).

• *Hylesinopsis granulata* (Lepesme, 1942c: 206) (*Kissophagus granulatus*).

• *Hylesinopsis hirsuta* (Schedl, 1958a: 869) (*Trypographus hirsutus*).

• *Hylesinopsis joveri* (Schedl, 1950f: 213) (*Trypographus*).

• *Hylesinopsis leprosula* (Browne, 1980e: 774) (*Cryphalus leprosulus*).

• *Hylesinopsis pauliani* (Lepesme, 1942c: 204) (*Kissophagus*).

• *Hylesinopsis saudiarabiae* (Schedl, 1971a: 434) (*Chilodendron*).

#### *Hylesinus* Fabricius

34 spp.

• *Hylesinus aculeatus* Say, 1824: 322.

= *Hylesinus imperialis* Eichhoff, 1868b: 149.

= *Leperisinus cinereus* Swaine, 1917: 15.

• *Hylesinus aesculi* (Kugelann, 1794: 525) (*Bostrichus*).

• *Hylesinus aztecus* Wood, 1980b: 354.

• *Hylesinus bicolor* (Eggers, 1939b: 4) (*Leperisinus*).

• *Hylesinus botscharnikovi* Stark, 1931: 81.

• *Hylesinus californicus* (Swaine, 1916: 190) (*Leperisinus*).

= *Leperisinus hoferi* Blackman, 1943d: 394.

• *Hylesinus caseariae* Wood, 1986a: 272.

• *Hylesinus cholodkovskyi* Berger, 1917: 246.

• *Hylesinus cingulatus* Blandford, 1894c: 67.

• *Hylesinus cordipennis* Lea, 1910: 144.

= *Hylesinus papuanus* Eggers, 1923b: 133.

• *Hylesinus crenatus* (Fabricius, 1787: 37) (*Bostrichus*).

= *Ips sulcatus* Marsham, 1802: 59.

= *Anthribus crenatus* Gravenhorst, 1807: 196.

= *Hylesinus prutenskyi* Sokanovskiy, 1959b: 276.

• *Hylesinus criddlei* (Swaine, 1918: 72) (*Leperisinus*).

• *Hylesinus dolus* Schedl, 1975j: 453.

• *Hylesinus eos* Spessivtsev, 1919: 248.

• *Hylesinus fasciatus* LeConte, 1868: 170.

• *Hylesinus guatemalensis* (Wood, 1967b: 89) (*Leperisinus*).

• *Hylesinus irresolutus* Walker, 1859: 261.

• *Hylesinus laticollis* Blandford, 1894c: 65.

= *Hylesinus pravdini* Stark, 1936: 151.

• *Hylesinus macmahoni* (Stebbing, 1909: 16) (*Sphaerotrypes*).

= *Hylesinus alternans* Schedl, 1959e: 172.

= *Leperisinus fraxinoides* Schedl, 1959f: 39.

• *Hylesinus mandshuricus* Eggers, 1922a: 15.

• *Hylesinus mexicanus* (Wood, 1956c: 249) (*Leperisinus*).

• *Hylesinus nanulus* Schedl, 1940b: 433.

• *Hylesinus nilgirinus* Eggers, 1923b: 133.

= *Hylesinus persimilis* Eggers, 1927b: 70.

= *Trogloditica robustus* Schedl, 1975j: 453.

• *Hylesinus nobilis* Blandford, 1894c: 64.

= *Hylesinus shabliovskyi* Kurentsov, 1941: 133.

• *Hylesinus oregonus* (Blackman, 1943d: 396) (*Leperisinus*).

• *Hylesinus pruinosus* Eichhoff, 1868b: 149.

• *Hylesinus regius* (Schedl, 1942c: 166) (*Leperisinus*).

• *Hylesinus striatus* Eggers, 1933f: 4.

• *Hylesinus taranio* (Danthoine in: Bernard 1788: 270) (*Byrrhus*).

= *Bostrichus oleiperda* Fabricius, 1792: 366.

= *Ips scaber* Marsham, 1802: 56.

= *Hylesinus bicolor* Brullé, 1832: 250.

= *Hylesinus suturalis* Redtenbacher, 1842: 21.

= *Hylesinus esau* Gredler, 1866: 370.

= *Hylesinus antipodus* Schedl, 1952i: 17.

• *Hylesinus tristis* Blandford, 1894c: 66.

= *Hylesinus lubarskii* Stark, 1936: 153.

• *Hylesinus tupolevi* Stark, 1936: 151.

= *Hylesinus tupolevi* Eggers, 1942a: 28.

= *Hylesinus tupolevi denticulosus* Sokanovskiy, 1956: 44.

• *Hylesinus varius* (Fabricius, 1775: 60) (*Bostrichus*).

= *Bostrichus minutus* Fabricius, 1777: 211.

= *Bostrichus melanocephalus* Fabricius, 1792: 368.

= *Anthribus pubescens* Fabricius, 1798: 161.

= *Bostrichus fraxini* Panzer, 1799: 15.

= *Scolytus varius* Olivier, 1800a: 11.

= *Hylesinus varius* Fabricius, 1801: 391.

= *Ips griseus* Marsham, 1802: 55.

= *Ips haemorrhoidalis* Marsham, 1802: 56.

= *Ips rufescens* Marsham, 1802: 55.

= *Bostrichus fraxini* Bechstein in: Bechstein and Scharfenberg 1804: 107.

= *Hylesinus picipennis* Stephens, 1830: 359.

= *Hylesinus henscheli* Knotek, 1892: 234.

• *Hylesinus verae* Petrov, 2002: 121.

• *Hylesinus wachtli* Reitter, 1887: 193.

= *Hylesinus orni* Fuchs, 1906: 51.

#### *Kissophagus* Chapuis

3 spp.

• *Kissophagus erinacellus* Wichmann, 1916: 18.

• *Kissophagus novaki* Reitter, 1894a: 45.

= *Kissophagus binodus* Reitter, 1894a: 45.

• *Kissophagus vicinus* (Comolli, 1837: 36) (*Hylesinus*).

= *Hylesinus hederae* Schmitt, 1843: 108.

= *Kissophagus nuesslini* Reitter, 1913: 44.

#### *Longulus* Krivolutskaya

1 sp.

• *Longulus elatus* (Niisima, 1913: 2) (*Hylesinus*).

#### *Neopteleobius* Nobuchi

1 sp.

• *Neopteleobius scutulatus* (Blandford, 1894c: 67) (*Hylesinus*).

= *Pteleobius trepanatus* Wichmann, 1914a: 137.

#### *Phloeoborus* Erichson

27 spp.

• *Phloeoborus araguensis* Wood, 2007: 43.

• *Phloeoborus asper* Erichson, 1836: 55.

= *Phloeoborus imbricornis* Eichhoff, 1868b: 148.

= *Phloeoborus rugatus* Blandford, 1897: 153.

• *Phloeoborus belti* Blandford, 1897: 151.

• *Phloeoborus cristatus* Chapuis, 1869: 13.

= *Phloeoborus radulosus* Blandford, 1897: 153.

= *Phloeoborus aspericollis* Strohmeyer, 1909: 248.

• *Phloeoborus ellipticus* Chapuis, 1869: 15.

• *Phloeoborus freyi* Schedl, 1955d: 274.

• *Phloeoborus gaujonii* Fairmaire, 1887: xvi.

• *Phloeoborus grandis* (Erichson, 1836: 54) (*Phloeotrupes*).

= *Phloeotrupes punctatus* Schedl, 1976: 64.

• *Phloeoborus granosus* Eichhoff, 1868b: 148.

• *Phloeoborus grossus* Chapuis, 1869: 13.

• *Phloeoborus intermedius* Eggers, 1930a: 165.

• *Phloeoborus irregularis* Eggers, 1942b: 270.

• *Phloeoborus mamillatus* Chapuis, 1869: 14.

• *Phloeoborus marahuaci* Wood, 2007: 40.

• *Phloeoborus minusculus* Wood, 2007: 39.

• *Phloeoborus niger* Wood, 2007: 40.

• *Phloeoborus nitidicollis* Chapuis, 1869: 38.

= *Phloeoborus breviusculus* Chapuis, 1869: 14.

• *Phloeoborus opacithorax* Schedl, 1940c: 205.

• *Phloeoborus orinocensis* Wood, 2007: 42.

• *Phloeoborus ovatus* Chapuis, 1869: 15.

= *Phloeoborus granulatus* Eggers, 1943a: 243.

• *Phloeoborus procerus* (Erichson, 1836: 54) (*Phloeotrupes*).

= *Phloeoborus sipolisii* Fairmaire, 1887: xvi.

• *Phloeoborus punctatorugosus* Chapuis, 1869: 14.

• *Phloeoborus rudis* Erichson, 1836: 55.

= *Phloeoborus elongatus* Chapuis, 1869: 13.

= *Phloeoborus rugipennis* Eggers, 1942b: 271.

• *Phloeoborus scaber* Erichson, 1836: 55.

= *Phloeoborus caelatus* Blanchard in: Blanchard and Brulle 1846: 204.

= *Phloeoborus sericeus* Chapuis, 1869: 13.

= *Phloeoborus lunulatus* Eggers, 1943a: 242.

• *Phloeoborus signatus* Strohmeyer, 1909: 248.

= *Phloeoborus bodei* Eggers, 1930a: 164.

= *Phloeoborus guayanensis* Eggers, 1942b: 268.

= *Phloeoborus similis* Eggers, 1942b: 272.

• *Phloeoborus sulcifrons* Eichhoff, 1868b: 148.

• *Phloeoborus willei* Wood, 2007: 35.

#### *Pteleobius* Bedel

2 spp.

• *Pteleobius kraatzii* (Eichhoff, 1864a: 30) (*Hylesinus*).

= *Hylesinus putonii* Eichhoff, 1868a: 403.

• *Pteleobius vittatus* (Fabricius, 1787: 38) (*Bostrichus*).

= *Ips coadunatus* Marsham, 1802: 55.

= *Ips furcatus* Marsham, 1802: 55.

= *Ips sericeus* Marsham, 1802: 55.

#### *Rhopalopselion* Hagedorn

11 spp.

• *Rhopalopselion atrum* Eggers, 1935e: 295.

• *Rhopalopselion bituberculatum* Hagedorn, 1909: 740.

• *Rhopalopselion confusum* Eggers, 1944c: 93.

• *Rhopalopselion conjungens* Schedl, 1965e: 5.

• *Rhopalopselion dentatum* Nunberg, 1956b: 200.

• *Rhopalopselion grande* Schedl, 1957b: 25.

• *Rhopalopselion immune* Eggers, 1943c: 71.

• *Rhopalopselion intermedium* Schedl, 1957b: 26.

• *Rhopalopselion nitidum* Schedl, 1957b: 27.

• *Rhopalopselion orientale* Schedl, 1957b: 153.

• *Rhopalopselion thompsoni* Schedl, 1954d: 74.

#### *Zygophloeus* Schedl

1 sp.

• *Zygophloeus australis* Schedl, 1958f: 215.

### HYLURGINI Gistel

15 genera, 136 spp. (3 spp. with 6 sspp.).

#### *Chaetoptelius* Fuchs

7 spp.

• *Chaetoptelius bimaculatus* (Schedl, 1936e: 520) (*Leperisinus*).

• *Chaetoptelius impar* (Schedl, 1975h: 216) (*Hylesinus*).

• *Chaetoptelius mundulus* (Broun, 1881: 740) (*Homarus*).

= *Acrantus opacus* Broun, 1895: 417.

• *Chaetoptelius opimus* (Wood, 1985: 270) (*Acrantus*).

• *Chaetoptelius tricolor* (Schedl, 1938g: 34) (*Leperisinus*).

• *Chaetoptelius versicolor* Wood, 1988a: 31 (RN).

= *Acrantus tricolor* Schedl, 1958b: 560.

• *Chaetoptelius vestitus* (Mulsant & Rey, 1861: 340) (*Hylesinus*).

= *Hylesinus vestitus* Mulsant & Rey, 1862: 87.

= *Hylesinus indigenus* Wollaston, 1864: 267.

#### *Dendroctonus* Erichson

21 spp. (1 sp. with 2 sspp.).

• *Dendroctonus adjunctus* Blandford, 1897: 147.

= *Dendroctonus convexifrons* Hopkins, 1909: 87.

• *Dendroctonus approximatus* Dietz, 1890: 28.

• *Dendroctonus armandi* Tsai & Li, 1959: 82.

= *Dendroctonus prosorovi* Kurentsov & Kononov, 1966: 29.

• *Dendroctonus barberi* Hopkins, 1909: 85.

• *Dendroctonus brevicomis* LeConte, 1876: 386.

• *Dendroctonus frontalis* Zimmermann, 1868: 149.

= *Dendroctonus arizonicus* Hopkins, 1909: 95.

• *Dendroctonus jeffreyi* Hopkins, 1909: 114.

• *Dendroctonus mesoamericanus* Armendariz & Sullivan in: Armendariz et al. 2015: 404.

• *Dendroctonus mexicanus* Hopkins, 1906: 80.

• *Dendroctonus micans* (Kugelann, 1794: 523) (*Bostrichus*).

= *Bostrichus abietinus* Fabricius, 1792: 367.

• *Dendroctonus murrayanae* Hopkins, 1909: 140.

• *Dendroctonus parallelocollis* Chapuis, 1869: 36.

= *Dendroctonus aztecus* Wood, 1963: 69.

• *Dendroctonus ponderosae* Hopkins, 1902b: 10.

= *Dendroctonus monticolae* Hopkins, 1905: 11.

• *Dendroctonus pseudotsugae barragani* Furniss, 2001: 21.

• *Dendroctonus pseudotsugae pseudotsugae* Hopkins, 1909: 11 (*Dendroctonus pseudotsugae*).

• *Dendroctonus punctatus* LeConte, 1868: 173.

= *Dendroctonus johanseni* Swaine, 1919: 3.

• *Dendroctonus rhizophagus* Thomas & Bright, 1970: 479.

• *Dendroctonus rufipennis* (Kirby, 1837: 195) (*Hylurgus*).

= *Hylurgus obesus* Mannerheim, 1843: 296.

= *Dendroctonus similis* LeConte, 1857: 59.

= *Dendroctonus piceaperda* Hopkins, 1901: 16.

= *Dendroctonus borealis* Hopkins, 1909: 133.

= *Dendroctonus engelmanni* Hopkins, 1909: 130.

• *Dendroctonus simplex* LeConte, 1868: 173.

• *Dendroctonus terebrans* (Olivier, 1800a: 6) (*Scolytus*).

• *Dendroctonus valens* LeConte, 1857: 59.

= *Dendroctonus beckeri* Thatcher, 1954: 4.

• *Dendroctonus vitei* Wood, 1974d: 289.

#### *Dendrotrupes* Broun

3 spp.

• *Dendrotrupes costiceps* Broun, 1881: 741.

• *Dendrotrupes vestitus* Broun, 1881: 741.

• *Dendrotrupes zealandicus* Wood, 1992: 86.

#### *Hylurdrectonus* Schedl

4 spp.

• *Hylurdrectonus araucariae* Schedl, 1964d: 213.

• *Hylurdrectonus corticinus* Wood, 1980a: 94 (RN).

= *Xylogopinus araucariae* Schedl, 1972k: 64.

• *Hylurdrectonus nanus* Browne, 1984e: 87.

• *Hylurdrectonus piniarius* Schedl, 1938g: 40.

#### *Hylurgonotus* Schedl

4 spp.

• *Hylurgonotus antipodus* (Eggers, 1942c: 14) (*Blastophagus*).

• *Hylurgonotus armaticeps* Schedl, 1955h: 257.

• *Hylurgonotus solidus* (Schedl, 1967d: 6) (*Blastophagus*).

• *Hylurgonotus tuberculatus* (Eggers, 1942c: 13) (*Hylurgus*).

= *Hylurgonotus brunneus* Schedl, 1952a: 448.

#### *Hylurgopinus* Swaine

1 sp.

• *Hylurgopinus rufipes* (Eichhoff, 1868b: 147) (*Hylastes*).

= *Hylesinus opaculus* LeConte, 1868: 170.

#### *Hylurgus* Latreille

4 spp.

• *Hylurgus indicus* Wood, 1985: 273.

• *Hylurgus ligniperda* (Fabricius, 1787: 37) (*Bostrichus*).

= *Bostrichus elongatus* Herbst, 1793: 117.

= *Hylesinus flavipes* Panzer, 1795: 61.

= *Hylurgus longulus* Kolenati, 1846: 38.

• *Hylurgus micklitzi* Wachtl in: Reitter 1881: 227.

• *Hylurgus rufipes* Stephens, 1835: 419.

#### *Pachycotes* Sharp

9 spp.

• *Pachycotes araucariae* Schedl, 1975a: 340.

• *Pachycotes australis* Schedl, 1937c: 38.

• *Pachycotes clavatus* Schedl, 1938g: 39.

• *Pachycotes engelsi* Mecke, 2004b: 346.

• *Pachycotes grandis* Mecke, 2004b: 344.

• *Pachycotes kuscheli* Schedl, 1972i: 267.

• *Pachycotes minor* Wood, 1985: 273.

• *Pachycotes peregrinus* (Chapuis, 1869: 21) (*Hylastes*).

= *Pachycotes ventralis* Sharp, 1877: 10.

• *Pachycotes villosus* Schedl, 1962b: 75.

#### *Pseudohylesinus* Swaine

11 spp. (2 spp. with 4 sspp.).

• *Pseudohylesinus dispar dispar* Blackman, 1942c: 11 (*Pseudohylesinus dispar*).

• *Pseudohylesinus dispar pullatus* Blackman, 1942c: 9 (*Pseudohylesinus pullatus*).

• *Pseudohylesinus granulatus* (LeConte, 1868: 175) (*Hylastes*).

• *Pseudohylesinus maculosus* Blackman, 1942c: 12.

• *Pseudohylesinus magnus* Wood, 1956c: 247.

• *Pseudohylesinus nebulosus nebulosus* (LeConte, 1860: 285) (*Hylesinus nebulosus*).

• *Pseudohylesinus nebulosus serratus* Bruck, 1936: 37 (*Pseudohylesinus serratus*).

• *Pseudohylesinus nobilis* Swaine, 1917: 12.

= *Pseudohylesinus furnissi* Blackman, 1942c: 21.

• *Pseudohylesinus pini* Wood, 1969c: 122.

• *Pseudohylesinus sericeus* (Mannerheim, 1843: 296) (*Hylurgus*).

= *Pseudohylesinus grandis* Swaine, 1917: 13.

= *Pseudohylesinus yasumatsui* Nobuchi, 1971: 160.

• *Pseudohylesinus sitchensis* Swaine, 1917: 17.

• *Pseudohylesinus tsugae* Swaine, 1917: 11.

= *Pseudohylesinus obesus* Swaine, 1917: 15.

= *Pseudohylesinus keeni* Blackman, 1942c: 17.

= *Pseudohylesinus similis* Blackman, 1942c: 18.

• *Pseudohylesinus variegatus* (Blandford, 1897: 145) (*Hylastes*).

= *Pseudohylesinus mexicanus* Blackman, 1942c: 13.

#### *Pseudoxylechinus* Wood & Huang

9 spp.

• *Pseudoxylechinus indicus* Wood, 1986b: 468.

• *Pseudoxylechinus piceae* Chen & Yin, 2001: 336.

• *Pseudoxylechinus rugatus* Wood & Huang, 1986: 467.

• *Pseudoxylechinus setosus* (Eggers, 1939b: 3) (*Kissophagus*).

• *Pseudoxylechinus sinensis* Wood & Huang, 1986: 467.

• *Pseudoxylechinus tibetensis* Wood & Huang, 1986: 466.

• *Pseudoxylechinus tiliae* (Niisima, 1910: 2) (*Kissophagus*).

• *Pseudoxylechinus uniformis* Wood & Huang, 1986: 465.

• *Pseudoxylechinus variegatus* Wood & Huang, 1986: 466.

#### *Sinophloeus* Brèthes

1 sp.

• *Sinophloeus porteri* Brèthes, 1923: 434.

= *Sinophloeus destructor* Eggers, 1942c: 15.

#### *Tomicus* Latreille

9 spp.

• *Tomicus armandii* Li & Zhang in: Li et al. 2010: 60.

• *Tomicus brevipilosus* (Eggers, 1929a: 103) (*Blastophagus*).

= *Blastophagus khasianus* Murayama, 1959: 75.

= *Blastophagus multisetosus* Murayama, 1963a: 37.

• *Tomicus destruens* (Wollaston, 1865: 45) (*Hylurgus*).

• *Tomicus heuksandoensis* Park in: Park et al. 2017: 132.

• *Tomicus minor* (Hartig, 1834: 443) (*Hylesinus*).

= *Myelophilus corsicus* Eggers, 1911a: 75.

• *Tomicus pilifer* (Spessivtsev, 1919: 250) (*Myelophilus*).

• *Tomicus piniperda* (Linnaeus, 1758: 355) (*Dermestes*).

= *Bostrichus testaceus* Fabricius, 1787: 37.

= *Hylurgus analogus* LeConte, 1868: 172.

= *Blastophagus major* Eggers, 1943b: 50.

• *Tomicus puellus* (Reitter, 1895: 53) (*Myelophilus*).

= *Blastophagus starki* Eggers, 1929a: 104.

= *Blastophagus puellus orientalis* Krivolutskaya, 1956b: 828.

• *Tomicus yunnanensis* Kirkendall & Faccoli in: Kirkendall et al. 2008: 32.

#### *Vorontsovia* Petrov

1 sp.

• *Vorontsovia andina* Petrov, 2014: 152.

#### *Xylechinosomus* Schedl

11 spp.

• *Xylechinosomus bicolor* (Philippi in: Philippi and Philippi 1864: 375) (*Hylesinus*).

• *Xylechinosomus brasiliensis* (Schedl, 1951c: 95) (*Pseudohylesinus*).

= *Xylechinosomus araucariae* Schedl, 1963f: 210.

• *Xylechinosomus contractus* (Chapuis, 1869: 23) (*Hylastes*).

= *Xylechinus taunayi* Eggers, 1928a: 84.

• *Xylechinosomus hirsutus* Schedl, 1963f: 211.

• *Xylechinosomus humilis* (Blanchard, 1851: 427) (*Hylesinus*).

• *Xylechinosomus lucianae* Mecke, 2004a: 217.

• *Xylechinosomus minimus* Schedl, 1963f: 212.

• *Xylechinosomus paranaensis* (Schönherr, 1994: 63) (*Pteleobius*).

• *Xylechinosomus pilosus* Wood, 1985: 274.

• *Xylechinosomus sachtlebeni* Schedl, 1963f: 209.

• *Xylechinosomus valdivianus* (Eggers, 1942c: 15) (*Xylechinus*).

#### *Xylechinus* Chapuis

41 spp.

• *Xylechinus aconcaguensis* Wood, 2007: 58.

• *Xylechinus africanus* Browne, 1973: 285.

• *Xylechinus americanus* Blackman, 1922b: 117.

• *Xylechinus araucariae* Mecke, 2004b: 347.

• *Xylechinus arisanus* Eggers, 1939a: 114.

• *Xylechinus australis* Schedl, 1957b: 31.

• *Xylechinus avarus* Wood, 1969a: 1.

• *Xylechinus bergeri* Spessivtsev, 1919: 249.

• *Xylechinus chiliensis* (Nunberg, 1964: 432) (*Squamosinus*).

= *Xylechinus sulcatus* Schedl, 1966d: 100.

• *Xylechinus darjeelingensis* Schedl, 1971e: 371.

• *Xylechinus declivis* Wood, 2007: 59.

• *Xylechinus fatsiae* Lin, 2020: 172.

• *Xylechinus freiburgi* Schedl, 1972f: 57.

• *Xylechinus fuliginosus* Blandford, 1897: 158.

• *Xylechinus gummensis* (Murayama, 1958: 931) (*Pruniphagus*).

• *Xylechinus huapiae* (Schedl, 1979a: 59) (*Phthorophloeus*).

• *Xylechinus imperialis* (Schedl, 1958j: 39) (*Pseudochramesus*).

= *Xylechinus calvus* Schedl, 1979a: 60.

• *Xylechinus irrasus* Blandford, 1897: 157.

• *Xylechinus maculatus* Schedl, 1952i: 18.

• *Xylechinus marmoratus* Blandford, 1897: 159.

• *Xylechinus mexicanus* Wood, 1974a: 7.

• *Xylechinus minor* Eggers, 1928a: 84.

• *Xylechinus montanus* Blackman, 1940a: 123.

• *Xylechinus nahueliae* (Schedl, 1979a: 59) (*Phthorophloeus*).

• *Xylechinus nigrosetosus* Hagedorn, 1909: 737.

• *Xylechinus obscurus* Eggers, 1941c: 222.

• *Xylechinus ougeniae* Wood, 1988a: 37.

• *Xylechinus padus* Wood, 1988a: 38.

• *Xylechinus pilosus* (Ratzeburg, 1837: 178) (*Hylesinus*).

• *Xylechinus porteri* Brèthes, 1925: 202.

= *Xylechinus spathifer* Schedl, 1955h: 256.

= *Pteleobius lomatiae* Schedl, 1975k: 2.

• *Xylechinus roeri* Schedl, 1977a: 395.

• *Xylechinus scabiosus* Blandford, 1897: 158.

• *Xylechinus smithae* Petrov, 2016: 1.

• *Xylechinus solervicensi* Wood, 2007: 60.

• *Xylechinus squamatilis* Wood, 2007: 56.

• *Xylechinus squamiger* Schedl, 1979a: 60.

• *Xylechinus squamosus* (Schedl, 1942c: 165) (*Phloeosinus*).

• *Xylechinus tessellatus* Blandford, 1897: 159.

• *Xylechinus tuberculifer* Wood, 2007: 55.

• *Xylechinus variegatus* (Chapuis, 1869: 40) (*Phloeosinus*).

• *Xylechinus vittatus* Schedl, 1966d: 100.

### HYORRHYNCHINI Hopkins

3 genera, 21 spp. (1 sp. with 2 sspp.).

#### *Hyorrhynchus* Blandford

10 spp.

• *Hyorrhynchus birmanus* Eggers, 1939b: 1.

• *Hyorrhynchus drescheri* Eggers, 1936e: 82.

• *Hyorrhynchus ebianensis* Huang & Yin, 1983: 339.

• *Hyorrhynchus elongatus* Eggers, 1939b: 2.

• *Hyorrhynchus kalimpongensis* Maiti & Saha, 1989: 42.

• *Hyorrhynchus lewisi* Blandford, 1894c: 60.

• *Hyorrhynchus sensarmai* Maiti & Saha, 1989: 44.

• *Hyorrhynchus shiva* Maiti & Saha, 1989: 46.

• *Hyorrhynchus tuberopectus* Huang & Yin, 1983: 340.

• *Hyorrhynchus unicornis* Nobuchi, 1966: 51.

#### *Pseudohyorrhynchus* Murayama

3 spp. (1 sp. with 2 sspp.).

• *Pseudohyorrhynchus blandfordi* (Sampson, 1913: 446) (*Hyorrhynchus*).

• *Pseudohyorrhynchus flavopannus* (Huang & Yin, 1983: 338) (*Hyorrhynchus*).

• *Pseudohyorrhynchus wadai kurilensis* Krivolutskaya, 1996: 331.

• *Pseudohyorrhynchus wadai wadai* Murayama, 1950a: 61 (*Pseudohyorrhynchus wadai*).

#### *Sueus* Murayama

8 spp.

• *Sueus borneensis* Bright, 1994: 271.

• *Sueus chatterjeei* Smith & Cognato in: Cognato et al. 2024: 482.

• *Sueus granulatus* (Eggers, 1936e: 80) (*Hyorrhynchus*).

• *Sueus insulanus* Schiffer, Smith & Cognato in: Cognato et al. 2024: 485.

• *Sueus niisimai* (Eggers, 1926a: 133) (*Hyorrhynchus*).

= *Sphaerotrypes controversae* Murayama, 1950a: 62.

= *Sueus sphaerotrypoides* Murayama, 1951: 2.

• *Sueus obesus* (Browne, 1977b: 369) (*Hyorrhynchus*).

• *Sueus pilosus* (Eggers, 1936e: 81) (*Hyorrhynchus*).

• *Sueus striatulus* (Schedl, 1954c: 145) (*Hyorrhynchus*).

### HYPOBORINI Nüsslin

12 genera, 59 spp. (1 sp. with 2 sspp.).

### HYPOBORINA Nüsslin

9 genera, 51 spp. (1 sp. with 2 sspp.).

#### *Afrotrypetus* Bright

2 spp.

• *Afrotrypetus capensis* Jordal, 2021a: 100.

• *Afrotrypetus euphorbiae* Bright, 1981c: 114.

#### *Corditarsus* Jordal

2 spp.

• *Corditarsus australis* (Schedl, 1975b: 279) (*Liparthrum*).

• *Corditarsus tanganyikaensis* (Schedl, 1972b: 296) (*Hypoborus*).

#### *Cryphyophthorus* Schedl

1 sp.

• *Cryphyophthorus eggersi* Schedl, 1953f: 294.

#### *Dacryostactus* Schaufuss

1 sp.

• *Dacryostactus kolbei* Schaufuss, 1905f: 79.

#### *Hypoborus* Erichson

1 sp.

• *Hypoborus ficus* Erichson, 1836: 62.

= *Hypoborus siculus* Ferrari, 1867: 18.

#### *Liparthrum* Wollaston

38 spp. (1 sp. with 2 sspp.).

• *Liparthrum albosetum* Bright, 1968b: 638.

• *Liparthrum americanum* Wood, 1969a: 8.

• *Liparthrum arizonicum* Wood, 1959b: 57.

• *Liparthrum arnoldi* Semenov, 1902: 272.

= *Liparthrum babadjanidis* Eggers, 1910a: 558.

• *Liparthrum artemisiae* Wollaston, 1854: 299.

• *Liparthrum artocarpus* Wood, 1988b: 193.

• *Liparthrum bartschti* Mühl, 1891: 202.

• *Liparthrum bicaudatum* Wollaston, 1865: 44.

• *Liparthrum bituberculatum* Wollaston, 1854: 297.

• *Liparthrum brincki* (Schedl, 1971b: 281) (*Dacryophthorus*).

• *Liparthrum canum* Israelson, 1990: 6.

• *Liparthrum carapae* Wood, 1971a: 9.

• *Liparthrum caymanense* Bright, 2019: 65 (E).

• *Liparthrum colchicum* Semenov, 1903: 79.

• *Liparthrum corsicum* Eichhoff, 1878a: 383.

= *Liparthrum corsicum* Eichhoff, 1878b: 110.

• *Liparthrum cracentis* Wood, 1969a: 9.

• *Liparthrum curtum* Wollaston, 1854: 298.

= *Liparthrum ciliatum* Eggers, 1928d: 283.

• *Liparthrum genistae genistae* (Aubé, 1862: 388) (*Hypoborus genistae*).

= *Liparthrum peyerimhoffi* Pfeffer, 1941b: 26.

• *Liparthrum genistae georgi* Knotek, 1895: 89.

= *Liparthrum albidum* Wichmann, 1916: 21.

= *Liparthrum cytisi* Eggers, 1927a: 121.

• *Liparthrum hispaniolum* Bright, 1981b: 161.

• *Liparthrum inarmatum* Wollaston, 1860b: 364.

• *Liparthrum laurivorum* Schedl, 1968a: 23 (E).

• *Liparthrum longifolia* (Stebbing, 1903: 267) (*Cryphalus*).

• *Liparthrum loweanum* Wollaston, 1868: 118.

• *Liparthrum lowei* Wollaston, 1862b: 174.

• *Liparthrum mandibulare* Wollaston, 1854: 295.

• *Liparthrum meridense* Wood, 1971a: 10 (E).

• *Liparthrum mexicanum* Wood, 1983: 657.

• *Liparthrum mori* (Aubé, 1862: 387) (*Hypoborus*).

= *Liparthrum dalmatinum* Eggers, 1944a: 140.

= *Liparthrum corsicum* Balachowsky, 1949b: 154.

= *Liparthrum balachowskyi* Pfeffer, 1995: 87 (RN).

• *Liparthrum nigrescens* Wollaston, 1865: 44.

= *Liparthrum degener* Lindberg, 1953: 18.

• *Liparthrum palaquium* (Schedl, 1953f: 292) (*Phloeochilus palaquius*).

• *Liparthrum pruni* Wood, 1983: 657.

• *Liparthrum sabahense* Bright, 1990: 485 (E).

• *Liparthrum semidegener* Israelson, 1990: 7.

• *Liparthrum squamosum* (Blackman, 1920a: 53) (*Erineosinus squamosus*).

• *Liparthrum thevetiae* Wood, 1981: 123.

• *Liparthrum tinianense* Wood, 1988b: 193 (E).

• *Liparthrum tongatapui* (Schedl, 1979g: 106) (*Mimiophthorus*).

• *Liparthrum turnbowi* Bright, 2019: 67.

#### *Phloeotrypetus* Wood

1 sp.

• *Phloeotrypetus palauensis* Wood, 1960a: 17.

#### *Styracoptinus* Wood

3 spp.

• *Styracoptinus cavipennis* (Schedl, 1969b: 13) (*Styracopterus*).

• *Styracoptinus ferreirai* (Schedl, 1969b: 12) (*Styracopterus*).

• *Styracoptinus murex* (Blandford, 1896c: 324) (*Styracopterus*).

#### *Trypanophellos* Bright

2 spp.

• *Trypanophellos minutum* Bright, 2019: 68.

• *Trypanophellos necopinus* Bright, 1982a: 166.

### XERASIBORINA Jordal

3 genera, 8 spp.

#### *Glochiphorus* Strohmeyer

1 sp.

• *Glochiphorus globosus* Strohmeyer, 1910b: 127.

#### *Nisiborus* Jordal

2 spp.

• *Nisiborus hylesiniformis* (Schedl, 1961g: 132) (*Ptilopodius*).

• *Nisiborus schaufussi* Jordal, 2021a: 107.

#### *Xerasiborus* Jordal

5 spp.

• *Xerasiborus asperatus* Jordal, 2021a: 105.

• *Xerasiborus bituberculatus* Jordal, 2021a: 103.

• *Xerasiborus euphorbiae* Jordal, 2021a: 104.

• *Xerasiborus quadrituberculatus* Jordal, 2021a: 102.

• *Xerasiborus zambesianus* Jordal, 2021a: 104.

### IPINI Bedel

8 genera, 231 spp. (6 spp. with 17 sspp.).

#### *Acanthotomicus* Blandford

98 spp.

• *Acanthotomicus acuminatus* (Schedl, 1963g: 71) (*Mimips*).

• *Acanthotomicus africanus* (Browne, 1970: 560) (*Orthotomicus*).

• *Acanthotomicus alternans* (Schedl, 1974d: 262) (*Ips*).

• *Acanthotomicus amatus* Schedl, 1954e: 156.

• *Acanthotomicus analogus* (Wood, 1971a: 40) (*Mimips*).

• *Acanthotomicus angolensis* (Schedl, 1965g: 1086) (*Mimips*).

• *Acanthotomicus angulatus* (Schedl, 1965g: 1086) (*Mimips*).

• *Acanthotomicus angylocalyx* (Schedl, 1957b: 75) (*Mimips*).

• *Acanthotomicus apicalis* (Schedl, 1977c: 501) (*Ips*).

• *Acanthotomicus artocarpi* (Browne, 1955: 347) (*Ips*).

• *Acanthotomicus ashantius* (Schedl, 1977b: 284) (*Mimips*).

• *Acanthotomicus australis* (Schedl, 1972e: 147) (*Ips*).

• *Acanthotomicus bicaudatus* (Eggers, 1927b: 80) (*Ips*).

• *Acanthotomicus biconicus* (Schedl, 1938d: 460) (*Myeloborus*).

= *Mimips confusus* Eggers, 1941b: 179.

= *Mimips cavifrons* Nunberg, 1961b: 342.

• *Acanthotomicus bidens* (Schedl, 1967c: 229) (*Mimips*).

• *Acanthotomicus bidentatus* (Schedl, 1958a: 876) (*Mimips*).

• *Acanthotomicus bidentis* Wood, 1972b: 191 (RN).

= *Mimips bidens* Wood, 1971a: 41.

• *Acanthotomicus bispinosus* (Eggers, 1927b: 78) (*Ips*).

• *Acanthotomicus bolivianus* (Eggers, 1943d: 357) (*Isophthorus*).

• *Acanthotomicus borneensis* Schedl, 1964i: 245.

• *Acanthotomicus caelatus* (Schedl, 1964b: 620) (*Mimips*).

• *Acanthotomicus camerunus* (Eggers, 1944c: 95) (*Mimips*).

• *Acanthotomicus caudatulus* (Schedl, 1962f: 192) (*Ips*).

• *Acanthotomicus caudatus* (Browne, 1970: 559) (*Mimips*).

• *Acanthotomicus celtis* (Schedl, 1955e: 295) (*Ips*).

• *Acanthotomicus chiriquensis* (Blandford, 1898a: 189) (*Xylocleptes*).

• *Acanthotomicus ciliatus* (Hagedorn, 1909: 743) (*Poecilips*).

• *Acanthotomicus cognatoi* Petrov in: Petrov and Atkinson 2018: 42.

• *Acanthotomicus congonus* (Eggers, 1932b: 33) (*Mimips*).

• *Acanthotomicus congruens* (Schedl, 1965g: 1089) (*Mimips*).

• *Acanthotomicus conjunctus* (Schedl, 1962g: 70) (*Mimips*).

• *Acanthotomicus craterigerus* (Nunberg, 1961a: 621) (*Orthotomicus*).

• *Acanthotomicus curvidens* (Schedl, 1955a: 258) (*Brachydendrulus*).

• *Acanthotomicus dentatus* Schedl, 1954c: 149.

• *Acanthotomicus diaboliculus* Cognato & Smith, 2020: 539.

• *Acanthotomicus duplicatus* (Schedl, 1973b: 170) (*Mimips*).

• *Acanthotomicus elongatus* (Schedl, 1961d: 86) (*Mimips*).

• *Acanthotomicus emarginatus* Browne, 1985a: 191.

• *Acanthotomicus enzoi* Cognato & Smith, 2020: 539.

• *Acanthotomicus euphorbiae* (Schedl, 1957b: 76) (*Mimips*).

= *Mimips atratus* Browne, 1965: 197.

• *Acanthotomicus excavatus* (Schedl, 1965g: 1090) (*Mimips*).

• *Acanthotomicus eximius* (Schedl, 1955e: 296) (*Ips*).

• *Acanthotomicus explanatus* Bright, 1994: 257.

• *Acanthotomicus fallaciosus* (Schedl, 1965g: 1091) (*Mimips*).

• *Acanthotomicus fici* Browne, 1984a: 154.

• *Acanthotomicus fortis* (Wood, 1971a: 40) (*Mimips*).

• *Acanthotomicus gracilis* (Schedl, 1966b: 236) (*Mimips*).

• *Acanthotomicus grandis* Browne, 1986b: 664.

• *Acanthotomicus granulatus* (Ferrari, 1867: 40) (*Xylocleptes*).

= *Mimips uncinatus* Wood, 1971a: 41.

• *Acanthotomicus harongae* (Schedl, 1954a: 882) (*Ips*).

• *Acanthotomicus immunitis* (Browne, 1965: 200) (*Mimips*).

• *Acanthotomicus insularis* (Eggers, 1923b: 164) (*Ips*).

= *Ips kepongi* Schedl, 1942b: 181.

= *Ips inclinans* Schedl, 1972j: 51.

• *Acanthotomicus intermedius* (Schedl, 1977b: 283) (*Dendrochilus*).

• *Acanthotomicus ipsiformus* Wood, 1985: 270.

• *Acanthotomicus katangensis* (Eggers, 1932b: 34) (*Mimips*).

• *Acanthotomicus kivuensis* (Schedl, 1957b: 74) (*Mimips*).

• *Acanthotomicus lefevrei* (Browne, 1973: 292) (*Mimips*).

• *Acanthotomicus lepidus* (Wichmann, 1914a: 136) (*Pityogenes*).

• *Acanthotomicus major* (Schedl, 1964b: 621) (*Mimips*).

• *Acanthotomicus medius* (Eggers, 1943c: 75) (*Mimips*).

• *Acanthotomicus mimicus* (Schedl, 1961h: 227) (*Mimips*).

• *Acanthotomicus octodentatus* Browne, 1984a: 153.

• *Acanthotomicus octospinosus* (Schedl, 1958a: 877) (*Mimips*).

• *Acanthotomicus ocularis* (Wood, 1971a: 42) (*Mimips*).

• *Acanthotomicus onerosus* (Schedl, 1942b: 180) (*Ips*).

• *Acanthotomicus pachylobius* (Schedl, 1966f: 371) (*Mimips*).

• *Acanthotomicus parcius* (Schedl, 1977c: 502) (*Ips*).

• *Acanthotomicus peregrinus* (Schedl, 1939b: 350) (*Ips*).

• *Acanthotomicus perexiguus* (Blandford, 1896e: 201) (*Tomicus*).

= *Ips denticulus* Eggers, 1923b: 163.

= *Ips philippinensis* Eggers, 1927b: 78.

• *Acanthotomicus pilosellus* (Browne, 1965: 197) (*Mimips*).

• *Acanthotomicus pilosus* (Eggers, 1924: 106) (*Ips*).

• *Acanthotomicus quadridens* (Schedl, 1965g: 1093) (*Mimips*).

• *Acanthotomicus quadrispinosus* (Eggers, 1920a: 243) (*Xylocleptes*).

• *Acanthotomicus quadrituberculatus* (Schedl, 1938b: 173) (*Isophthorus*).

= *Mimips fallax* Eggers, 1943c: 76.

• *Acanthotomicus robertsi* (Browne, 1965: 198) (*Mimips*).

• *Acanthotomicus sexdentatus* (Eggers, 1944c: 96) (*Mimips*).

• *Acanthotomicus sexspinosus* (Schedl, 1942b: 181) (*Ips*).

• *Acanthotomicus seydeli* (Schedl, 1966f: 372) (*Mimips*).

• *Acanthotomicus similis* Bright, 2000: 52.

• *Acanthotomicus simplex* (Schedl, 1965g: 1094) (*Mimips*).

• *Acanthotomicus sindorae* Browne, 1981b: 600.

• *Acanthotomicus spinidens* (Schedl, 1965g: 1095) (*Mimips*).

• *Acanthotomicus spinosus* Blandford, 1894c: 90.

• *Acanthotomicus subimmunitus* (Schedl, 1964h: 47) (*Mimips*).

• *Acanthotomicus sumatranus* Strohmeyer, 1908a: 69.

= *Ips latedeclivis* Schedl, 1942d: 25.

• *Acanthotomicus suncei* Cognato in: Gao and Cognato 2018: 596.

• *Acanthotomicus suspectus* (Schedl, 1966f: 373) (*Mimips*).

• *Acanthotomicus suturifer* (Schedl, 1965e: 11) (*Mimips*).

• *Acanthotomicus tanganyikaensis* (Schedl, 1957c: 157) (*Ips*).

• *Acanthotomicus tenuis* (Schedl, 1966b: 237) (*Mimips*).

• *Acanthotomicus tridens* (Schedl, 1967c: 230) (*Mimips*).

• *Acanthotomicus tridentatus* (Schedl, 1977b: 284) (*Mimips*).

• *Acanthotomicus tropicus* (Schedl, 1936c: 25) (*Orthotomicus*).

• *Acanthotomicus tuberculatus* (Eggers, 1927b: 79) (*Ips*).

• *Acanthotomicus tuberculifer* Wood, 1988a: 31 (RN).

= *Mimips tuberculatus* Schedl, 1967c: 230.

• *Acanthotomicus uncus* (Schedl, 1942b: 180) (*Ips*).

• *Acanthotomicus uniformis* (Schedl, 1965g: 1096) (*Mimips*).

• *Acanthotomicus variabilis* (Browne, 1963a: 246) (*Mimips*).

#### *Ips* DeGeer

37 spp. (5 spp. with 15 sspp.).

• *Ips acuminatus* (Gyllenhal, 1827: 620) (*Bostrichus*).

= *Bostrichus geminatus* Zetterstedt, 1828: 345.

= *Tomicus heydeni* Eichhoff in: Heyden 1884: 298.

• *Ips amitinus* (Eichhoff, 1872b: 138) (*Tomicus*).

= *Tomicus amitinus montanus* Fuchs, 1913: 80.

• *Ips apache* Lanier, 1991: 1118.

• *Ips avulsus* (Eichhoff, 1868c: 402) (*Tomicus*).

• *Ips bonanseai* (Hopkins, 1906: 76) (*Tomicus*).

• *Ips borealis borealis* Swaine, 1911: 213 (*Ips borealis*).

• *Ips borealis lanieri* Wood, 1974a: 27.

= *Ips borealis* Wood, 1974a: 27.

• *Ips borealis swainei* Hopping, 1939: 169 (*Ips swainei*).

• *Ips borealis thomasi* Hopping, 1965: 193 (*Ips thomasi*).

• *Ips calligraphus calligraphus* (Germar, 1823: 461) (*Tomicus calligraphus*).

= *Bostrichus exesus* Say, 1826: 255.

= *Bostrichus chloroticus* Dejean, 1836: 332.

= *Bostrichus conformis* Dejean, 1836: 332.

= *Tomicus praemorsus* Eichhoff, 1868c: 401.

• *Ips calligraphus interstitialis* Eichhoff, 1869: 273 (*Tomicus interstitialis*).

• *Ips calligraphus ponderosae* Swaine, 1925b: 197 (*Ips ponderosae*).

• *Ips cembrae* (Heer, 1836: 28) (*Bostrichus*).

= *Ips engadinensis* Fuchs, 1913: 82.

= *Ips fallax* Eggers, 1915b: 96.

= *Ips shinanoensis* Yano, 1924: 2.

• *Ips chinensis* Kurentsov & Kononov, 1966: 31.

• *Ips confusus* (LeConte, 1876: 364) (*Tomicus*).

• *Ips cribricollis* (Eichhoff, 1869: 273) (*Tomicus*).

= *Ips cloudcrofti* Swaine, 1924a: 70.

• *Ips duplicatus* (Sahlberg, 1836b: 144) (*Bostrichus*).

= *Bostrichus judeichii* Kirsch, 1870: 388.

= *Tomicus infucatus* Eichhoff, 1877b: 392.

= *Tomicus infucatus* Eichhoff, 1878b: 247.

• *Ips emarginatus* (LeConte, 1876: 364) (*Tomicus*).

• *Ips grandicollis* (Eichhoff, 1868c: 402) (*Tomicus*).

= *Tomicus cacographus* LeConte, 1868: 162.

= *Ips chagnoni* Swaine, 1916: 186.

• *Ips hauseri* Reitter, 1895: 81.

= *Ips ussuriensis* Reitter, 1913: 107.

• *Ips hoppingi* Lanier, 1970a: 1145.

• *Ips hunteri* Swaine, 1917: 31.

• *Ips integer* (Eichhoff, 1869: 273) (*Tomicus*).

• *Ips knausi* Swaine, 1915b: 355.

• *Ips lecontei* Swaine, 1924a: 70.

• *Ips longifolia* (Stebbing, 1909: 26) (*Tomicus*).

• *Ips montanus* (Eichhoff, 1881: 219) (*Tomicus*).

= *Ips vancouveri* Swaine, 1916: 188.

• *Ips nitidus* Eggers, 1933d: 101.

• *Ips paraconfusus* Lanier, 1970a: 1146.

• *Ips perroti* Swaine, 1915b: 356.

• *Ips perturbatus* (Eichhoff, 1869: 274) (*Tomicus*).

= *Tomicus hudsonicus* LeConte, 1876: 366.

= *Tomicus interpunctus* Eichhoff, 1878b: 241.

• *Ips pilifrons pilifrons* Swaine, 1912: 353 (*Ips pilifrons*).

• *Ips pilifrons sulcifrons* Wood, 1960b: 67 (*Ips sulcifrons*).

• *Ips pilifrons thatcheri* Wood, 1975a: 29 (*Ips thatcheri*).

• *Ips pilifrons utahensis* Wood, 1960b: 66 (*Ips utahensis*).

• *Ips pini* (Say, 1826: 257) (*Bostrichus*).

= *Bostrichus dentatus* Sturm, 1826: 76.

= *Bostrichus pallipes* Sturm, 1826: 76.

= *Tomicus praefrictus* Eichhoff, 1868c: 401.

= *Tomicus oregonis* Eichhoff, 1869: 274.

= *Tomicus rectus* LeConte, 1876: 365.

= *Ips laticollis* Swaine, 1918: 116.

• *Ips plastographus maritimus* Lanier, 1970b: 1417.

• *Ips plastographus plastographus* LeConte, 1868: 163 (*Tomicus plastographus*).

• *Ips schmutzenhoferi* Holzschuh, 1988: 482.

• *Ips sexdentatus* (Boerner, 1776: 78) (*Dermestes*).

= *Bostrichus pinastri* Bechstein, 1818: 184.

= *Bostrichus stenographus* Duftschmid, 1825: 88.

= *Ips sexdentatus junnanicus* Sokanovskiy, 1959a: 94.

• *Ips shangrila* Cognato & Sun, 2007: 546.

• *Ips stebbingi* Strohmeyer, 1908a: 69.

= *Tomicus blandfordi* Stebbing, 1909: 27.

= *Tomicus ribbentropi* Stebbing, 1909: 25.

• *Ips subelongatus* (Motschulsky, 1860: 155) (*Tomicus*).

• *Ips tridens engelmanni* Swaine, 1917: 30 (*Ips engelmanni*).

= *Ips dubius* Swaine, 1918: 119.

= *Ips yohoensis* Swaine, 1918: 120.

= *Ips amiskwiensis* Hopping, 1963b: 216.

= *Ips semirostris* Hopping, 1963b: 213.

• *Ips tridens tridens* Mannerheim, 1852: 357 (*Bostrichus tridens*).

= *Bostrichus interruptus* Mannerheim, 1852: 357.

• *Ips typographus* (Linnaeus, 1758: 355) (*Dermestes*).

= *Bostrichus octodentatus* Paykull, 1800: 146.

= *Ips typographus japonicus* Niisima, 1909: 147.

• *Ips woodi* Thatcher, 1965: 493.

#### *Orthotomicus* Ferrari

21 spp.

• *Orthotomicus angulatus* (Eichhoff in: Chapuis and Eichhoff 1876: 200) (*Tomicus*).

• *Orthotomicus caelatus* (Eichhoff, 1868c: 402) (*Tomicus*).

= *Tomicus decretus* Eichhoff, 1868c: 402.

= *Xyleborus vicinus* LeConte, 1874: 72.

= *Xyleborus punctipennis* LeConte, 1878b: 624.

• *Orthotomicus chaokhao* Cognato, 2008: 496.

• *Orthotomicus erosus* (Wollaston, 1857: 95) (*Tomicus*).

= *Tomicus rectangulus* Eichhoff in: Ferrari 1867: 83.

= *Tomicus rectangulus* Eichhoff, 1878b: 260.

• *Orthotomicus golovjankoi* Pyatnitskiy, 1930: 179.

• *Orthotomicus kuniyoshii* (Nobuchi, 1959b: 23) (*Ips*).

• *Orthotomicus laricis* (Fabricius, 1792: 365) (*Bostrichus*).

• *Orthotomicus latidens* (LeConte, 1874: 72) (*Tomicus*).

= *Ips longidens* Swaine, 1911: 214.

= *Ips guildi* Blackman, 1922c: 137.

• *Orthotomicus longicollis* (Gyllenhal, 1827: 621) (*Bostrichus*).

= *Tomicus oblitus* Perris, 1862: 218.

• *Orthotomicus mannsfeldi* (Wachtl, 1880: 51) (*Tomicus*).

• *Orthotomicus multidentatus* (Murayama, 1953b: 34) (*Ips*).

• *Orthotomicus nankinensis* Kurentsov & Kononov, 1966: 32.

• *Orthotomicus nobilis* (Wollaston, 1862a: 441) (*Tomicus*).

• *Orthotomicus pinivorus* Schedl, 1961c: 187 (E).

• *Orthotomicus proximus* (Eichhoff, 1868a: 403) (*Tomicus*).

= *Tomicus omissus* Eichhoff, 1872b: 138.

= *Ips fejferi* Keler, 1925: 191.

• *Orthotomicus robustus* (Knotek, 1899: 296) (*Tomicus*).

= *Ips erosus melanurus* Reitter, 1913: 109.

• *Orthotomicus spinifer* (Eichhoff, 1878b: 499) (*Tomicus*).

= *Orthotomicus sabinianae* Hopping, 1963a: 64.

• *Orthotomicus starki* Spessivtsev, 1926b: 217.

• *Orthotomicus suturalis* (Gyllenhal, 1827: 622) (*Bostrichus*).

= *Bostrichus nigritus* Gyllenhal, 1827: 623.

• *Orthotomicus tosaensis* (Murayama, 1950b: 52) (*Ips*).

• *Orthotomicus tridentatus* (Eggers, 1921: 41) (*Ips*).

#### *Pityogenes* Bedel

24 spp. (1 sp. with 2 sspp.).

• *Pityogenes bidentatus* (Herbst, 1784: 96) (*Bostrichus*).

= *Bostrichus ater* Fabricius, 1792: 368.

= *Bostrichus bidens* Fabricius, 1792: 368.

= *Bostrichus bispinus* Guyon, 1855: 4815.

= *Pityogenes bidentatus carniolica* Fuchs, 1911: 35.

= *Pityogenes obtusus* Eggers, 1932a: 81.

• *Pityogenes bistridentatus* (Eichhoff, 1878b: 282) (*Tomicus*).

= *Pityogenes pilidens* Reitter, 1895: 78.

= *Pityogenes pilidens albanicus* Apfelbeck, 1896: 539.

• *Pityogenes calcaratus* (Eichhoff, 1878b: 280) (*Tomicus*).

= *Tomicus lipperti* Henschel, 1885: 242.

= *Pityogenes opacifrons* Reitter, 1913: 99.

= *Pityogenes herbellae* Strohmeyer, 1929: 181.

• *Pityogenes carinulatus* (LeConte, 1874: 70) (*Cryphalus*).

= *Xyleborus hamatus* LeConte, 1874: 72.

• *Pityogenes chalcographus* (Linnaeus, 1761: 143) (*Dermestes*).

= *Ips spinosus* DeGeer, 1775: 197.

= *Scolytus sexdentatus* Olivier, 1800a: 11.

= *Bostrichus xylographus* Sahlberg, 1836a: 148.

• *Pityogenes conjunctus* (Reitter, 1887: 196) (*Tomicus bistridentatus conjunctus*).

= *Pityogenes baicalicus* Eggers, 1933b: 49.

• *Pityogenes fossifrons* (LeConte, 1876: 353) (*Pityophthorus*).

• *Pityogenes foveolatus* Eggers, 1926a: 137.

• *Pityogenes hopkinsi* Swaine, 1915a: 8.

• *Pityogenes irkutensis irkutensis* Eggers, 1910b: 38 (*Pityogenes irkutensis*).

• *Pityogenes irkutensis monacensis* Fuchs, 1911: 3 (*Pityogenes monacensis*).

= *Pityogenes monacensis bialowiezensis* Karpiński, 1931: 34.

• *Pityogenes japonicus* Nobuchi, 1974: 39.

• *Pityogenes knechteli* Swaine, 1918: 106.

• *Pityogenes meridianus* Blackman, 1921: 15.

• *Pityogenes mexicanus* Wood, 1980b: 356.

• *Pityogenes pennidens* (Reitter, 1889b: 374) (*Tomicus*).

• *Pityogenes plagiatus* (LeConte, 1868: 161) (*Xyleborus*).

= *Pityogenes lecontei* Swaine, 1915a: 10.

• *Pityogenes porifrons* Eggers, 1933b: 50.

• *Pityogenes quadridens* (Hartig, 1834: 109) (*Bostrichus*).

• *Pityogenes rudnevi* Sokanovskiy, 1959b: 278.

• *Pityogenes saalasi* Eggers, 1914b: 188.

= *Pityogenes saalasi niger* Sokanovskiy, 1960: 676.

• *Pityogenes scitus* Blandford, 1893a: 63.

= *Pityophthorus coniferae* Stebbing, 1909: 30.

• *Pityogenes seirindensis* Murayama, 1929: 26.

= *Pityogenes aizawai* Kôno, 1938: 69.

= *Pityogenes nitidus* Eggers, 1941d: 121.

• *Pityogenes spessivtsevi* Lebedev, 1926: 120.

• *Pityogenes trepanatus* (Nördlinger, 1848: 239) (*Bostrichus*).

= *Tomicus austriacus* Wachtl, 1887: 320.

= *Tomicus elongatus* Løvendal, 1889b: 61.

#### *Pityokteines* Fuchs

10 spp.

• *Pityokteines curvidens* (Germar, 1823: 462) (*Tomicus*).

= *Tomicus psilonotus* Germar, 1823: 463.

= *Bostrichus calligraphus* Duftschmid, 1825: 91.

= *Bostrichus orthographus* Duftschmid, 1825: 91.

• *Pityokteines elegans* Swaine, 1916: 182.

• *Pityokteines lasiocarpi* (Swaine, 1916: 183) (*Orthotomicus*).

• *Pityokteines marketae* Knížek, 1998: 189.

• *Pityokteines minutus* (Swaine, 1912: 352) (*Dryocoetes*).

= *Pityokteines jasperi* Swaine, 1916: 181.

• *Pityokteines mystacinus* Wood, 1975a: 29.

• *Pityokteines ornatus* (Swaine, 1916: 185) (*Orthotomicus*).

• *Pityokteines sparsus* (LeConte, 1868: 160) (*Xyleborus*).

= *Tomicus balsameus* LeConte, 1878b: 625.

• *Pityokteines spinidens* (Reitter, 1895: 85) (*Ips*).

= *Tomicus curvidens heterodon* Wachtl, 1895: 15.

• *Pityokteines vorontzowi* (Jacobson in: Jakobson 1895: 521) (*Tomicus*).

#### *Premnobius* Eichhoff

28 spp.

• *Premnobius adjunctus* (Eggers, 1924: 108) (*Xyleborus*).

• *Premnobius ambitiosus* (Schaufuss, 1897a: 109) (*Xylocleptes*).

= *Premnobius cavipennis spinosus* Hagedorn, 1908: 376.

= *Premnobius brasiliensis* Nunberg, 1958: 490.

• *Premnobius amphicranoides* Schedl, 1939c: 172.

• *Premnobius assiduus* (Schedl, 1961h: 228) (*Xyleborus*).

• *Premnobius brownei* Atkinson & Flechtmann in: Atkinson et al. 2023: 74.

• *Premnobius cavipennis* Eichhoff, 1878b: 404.

= *Xyleborus industrius* Sampson, 1912: 249.

= *Xyleborus xylocranellus* Schedl, 1931a: 344.

= *Premnobius bituberculatus* Eggers, 1932b: 35.

= *Premnobius latior* Eggers, 1933f: 9.

• *Premnobius circumspinatus* Eggers, 1924: 107.

= *Premnobius circumdentatus* Schedl, 1938d: 461.

• *Premnobius corruptus* (Schedl, 1963g: 564) (*Xyleborus adjunctus corruptus*).

• *Premnobius corthyloides* Hagedorn, 1910: 1 (*Premnobius cavipennis corthyloides*).

= *Premnobius binopdosus* Nunberg, 1969: 396.

= *Premnobius ivoriensis* Nunberg, 1969: 398.

• *Premnobius declivis* Eggers, 1944c: 97.

= *Xyleborus perdeclivis* Schedl, 1957b: 85 (RN).

• *Premnobius familiaris* (Schedl, 1965f: 72) (*Xyleborus*).

• *Premnobius felix* (Schedl, 1971c: 198) (*Xyleborus*).

• *Premnobius flechtmanni* (Wood, 2007: 337) (*Acanthotomicus*).

• *Premnobius hystrix* (Schedl, 1957b: 108) (*Xyleborus*).

• *Premnobius longus* Eggers, 1932b: 36.

= *Xyleborus artelongus* Schedl, 1957b: 85 (RN).

• *Premnobius marginatus* Eggers, 1932b: 37.

= *Xyleborus marginatulus* Schedl, 1957b: 85 (RN).

• *Premnobius minor* Eggers, 1927c: 183.

= *Xyleborus perminor* Schedl, 1957b: 85 (RN).

• *Premnobius mukunyae* (Schedl, 1957b: 111) (*Xyleborus*).

• *Premnobius neoadjunctus* (Schedl, 1967d: 13) (*Xyleborus*).

• *Premnobius nodulosus* Hagedorn, 1908: 376.

• *Premnobius orientalis* Eggers, 1932b: 36.

= *Xyleborus perorientalis* Schedl, 1957b: 85 (RN).

• *Premnobius perezdelacrucei* Petrov & Atkinson, 2018: 42.

= *Gnathotrupes megapunctatus* Bright, 2019: 410.

• *Premnobius pseudohystrix* (Schedl, 1959h: 26) (*Xyleborus*).

• *Premnobius quadridens* Eggers, 1927c: 183.

= *Xyleborus quadridentatus* Schedl, 1957b: 85 (RN).

• *Premnobius robustulus* (Schedl, 1957b: 112) (*Xyleborus*).

• *Premnobius sexspinosus* Eggers, 1924: 107.

• *Premnobius spinifer* Eggers, 1927c: 182.

= *Xyleborus perspinifer* Schedl, 1957b: 85 (RN).

• *Premnobius subadjunctus* (Schedl, 1950b: 28) (*Xyleborus usagaricus subadjunctus*).

#### *Premnophilus* Browne

8 spp.

• *Premnophilus bertii* Atkinson & Flechtmann in: Atkinson et al. 2023: 80.

• *Premnophilus jordali* Petrov in: Atkinson et al. 2023: 81.

• *Premnophilus maiai* Atkinson & Flechtmann in: Atkinson et al. 2023: 84.

• *Premnophilus pedrosai* Atkinson & Flechtmann in: Atkinson et al. 2023: 84.

• *Premnophilus perspinidens* (Schedl, 1957b: 107) (*Xyleborus*).

• *Premnophilus quadrispinosus* (Schedl, 1938d: 461) (*Premnobius*).

= *Xyleborus joveri* Schedl, 1951h: 41.

= *Xyleborus perquadrispinosus* Schedl, 1957b: 85 (RN).

• *Premnophilus sarahsmithae* Atkinson & Flechtmann in: Atkinson et al. 2023: 86.

• *Premnophilus wilsoni* Atkinson & Flechtmann in: Atkinson et al. 2023: 87.

#### *Pseudips* Cognato

5 spp.

• *Pseudips concinnus* (Mannerheim, 1852: 358) (*Bostrichus*).

= *Tomicus hirsutus* Eichhoff, 1868c: 402.

= *Ips chamberlini* Swaine, 1925b: 196.

• *Pseudips mexicanus* (Hopkins, 1906: 75) (*Tomicus*).

• *Pseudips orientalis* (Wood & Yin, 1986: 461) (*Ips*).

• *Pseudips radiatae* (Hopkins, 1915b: 54) (*Tomicus*).

• *Pseudips yak* Cognato & Smith, 2024: 249.

### MICRACIDINI LeConte

22 genera, 372 spp.

#### *Afromicracis* Schedl

16 spp.

• *Afromicracis brevipilosa* Jordal, 2021e: 82.

• *Afromicracis concava* Jordal, 2021e: 78.

• *Afromicracis congona* (Eggers, 1940d: 102) (*Miocryphalus congonus*).

• *Afromicracis crassa* Jordal, 2021e: 74.

• *Afromicracis crinita* Jordal, 2021e: 74.

• *Afromicracis densisetosa* Jordal, 2021e: 79.

• *Afromicracis depilata* Jordal, 2021e: 85.

• *Afromicracis elongatula* (Schedl, 1977b: 282) (*Dendrochilus elongatulus*).

• *Afromicracis ghanaensis* Jordal, 2021e: 79.

• *Afromicracis jasminiae* (Schedl, 1957b: 81) (*Dendrochilus*).

• *Afromicracis kenyaensis* Schedl, 1959a: 709.

• *Afromicracis longa* (Nunberg, 1964: 434) (*Miocryphalus longus*).

• *Afromicracis mikaniae* (Schedl, 1957b: 82) (*Dendrochilus*).

• *Afromicracis natalensis* (Eggers, 1936c: 36) (*Stephanoderes*).

• *Afromicracis robusta* (Schedl, 1957b: 80) (*Dendrochilus robustus*).

= *Dendrochilus arundinarius* Schedl, 1957b: 80.

= *Dendrochilus filum* Schedl, 1977b: 283.

= *Hypothenemus bambusae* Browne, 1980e: 775.

• *Afromicracis setifera* (Schedl, 1957c: 156) (*Mimiophthorus setifer*).

#### *Cactopinus* Schwarz

22 spp.

• *Cactopinus agavensis* Atkinson, 2010: 24.

• *Cactopinus atkinsoni* Wood, 1983: 651.

• *Cactopinus burjosi* Wood, 1983: 651.

• *Cactopinus cactophthorus* Wood, 1957: 105.

• *Cactopinus carinatus* Wood, 1969b: 50.

• *Cactopinus depressus* Bright, 1967: 921.

• *Cactopinus desertus* Bright, 1967: 923.

• *Cactopinus granulatus* Wood, 1983: 651.

• *Cactopinus granulifer* Wood, 1969b: 48.

• *Cactopinus hubbardi* Schwarz, 1899: 11.

• *Cactopinus koebelei* Blackman, 1938b: 156.

• *Cactopinus mexicanus* Wood, 1967a: 37.

• *Cactopinus microcornis* Wood, 1969b: 45.

• *Cactopinus nasutus* Wood, 1969b: 49.

• *Cactopinus niger* Wood, 1969b: 46.

• *Cactopinus pini* Blackman, 1938b: 153.

• *Cactopinus rhettbutleri* Atkinson, 2016: 196.

• *Cactopinus rhois* Blackman, 1938b: 154.

• *Cactopinus setosus* Wood, 1983: 652.

• *Cactopinus spinatus* Wood, 1957: 106.

• *Cactopinus sulcifrons* Atkinson, 2010: 21.

• *Cactopinus woodi* Atkinson, 2010: 20.

#### *Dendrochilus* Schedl

3 spp.

• *Dendrochilus strombosiopsis* (Schedl, 1957b: 79) (Dendrochilus*).

• *Dendrochilus tener* Jordal, 2021d: 590.

• *Dendrochilus udzungwae* Jordal, 2021d: 592.

#### *Diplotrichus* Jordal

20 spp.

• *Diplotrichus acutior* Jordal, 2021g: 112.

• *Diplotrichus calvifrons* Jordal, 2021g: 108.

• *Diplotrichus catenatus* (Schedl, 1953c: 86) (*Macraciops*).

• *Diplotrichus elongatus* (Schedl, 1950d: 107) (*Landolphianus*).

• *Diplotrichus euphorbiae* (Schedl, 1961g: 137) (*Lanurgus*).

• *Diplotrichus falcatus* Jordal, 2021g: 112.

• *Diplotrichus gracilis* (Schedl, 1958b: 559) (*Lanurgus*).

• *Diplotrichus granulatus* Jordal, 2021g: 118.

• *Diplotrichus ignotus* (Schedl, 1965f: 62) (*Micracis*).

• *Diplotrichus medius* Jordal, 2021g: 110.

• *Diplotrichus minor* (Schedl, 1950d: 108) (*Landolphianus*).

= *Lanurgus frontalis* Schedl, 1953c: 85.

• *Diplotrichus obesus* (Schedl, 1953c: 87) (*Micraciops*).

• *Diplotrichus pilifrons* Jordal, 2021g: 108.

• *Diplotrichus plenus* Jordal, 2021g: 116.

• *Diplotrichus pulchellus* Jordal, 2021g: 114.

• *Diplotrichus robustus* Jordal, 2021g: 120.

• *Diplotrichus rugosipes* (Schedl, 1961g: 138) (*Lanurgus*).

= *Lanurgus pygmaeus* Schedl, 1965f: 66.

• *Diplotrichus subdepressus* (Schedl, 1965f: 66) (*Lanurgus*).

• *Diplotrichus tuberculatus* Jordal, 2021g: 116.

• *Diplotrichus widdringtoniae* (Schedl, 1962g: 69) (*Lanurgus*).

= *Glostatus perplexus* Schedl, 1982: 281.

#### *Hylocurus* Eichhoff

84 spp.

• *Hylocurus aberrans* Wood, 1969a: 44.

• *Hylocurus absonus* Bright, 2019: 73.

• *Hylocurus acutedentatus* Schedl, 1978a: 300.

• *Hylocurus aequalis* Wood, 2007: 327.

• *Hylocurus alienus* Eichhoff, 1878b: 301.

• *Hylocurus alternatus* Eggers, 1951: 153.

• *Hylocurus alternus* Wood, 1969a: 43.

• *Hylocurus anomala* Bright, 2019: 74.

• *Hylocurus antillicus* Bright, 2019: 74.

• *Hylocurus atkinsoni* Wood, 1987: 547.

• *Hylocurus beckeri* Heqvist, 1954: 8.

• *Hylocurus biconcavus* Blackman, 1943c: 344.

• *Hylocurus bicornus* (Blackman, 1920a: 23) (*Micracis*).

• *Hylocurus brasiliensis* Nunberg, 1956c: 208 (RN).

= *Hylocurus simplex* Schedl, 1954b: 33.

= *Hylocurus rectus* Schedl, 1958i: 144 (RN).

• *Hylocurus cancellatus* Blandford, 1898a: 221.

• *Hylocurus carinifrons* Atkinson, 1989b: 331.

• *Hylocurus clarki* Wood, 1979: 141.

• *Hylocurus colombianus* Wood, 2007: 321.

• *Hylocurus crotonis* Wood, 1987: 548.

• *Hylocurus cuspidatus* Eggers, 1951: 153.

• *Hylocurus declivis* Wood, 2007: 326.

• *Hylocurus dilutus* Wood, 1971a: 29.

• *Hylocurus dimorphus* (Schedl, 1939h: 724) (*Micracis*).

= *Hylocurus acuminatus* Schedl, 1950c: 148.

• *Hylocurus discifer* Eichhoff, 1878b: 300.

= *Hylocurus bidentatus* Schedl, 1950c: 149.

• *Hylocurus disparilis* Wood, 1971a: 29.

• *Hylocurus dissidens* Wood, 1971a: 30.

• *Hylocurus dissimilis* Wood, 1984a: 115.

• *Hylocurus effeminatus* Wood, 1956a: 143.

• *Hylocurus egenus* Blandford, 1898a: 222.

• *Hylocurus elegans* Eichhoff, 1872a: 134.

= *Hylocurus minor* Wood, 1961a: 4.

• *Hylocurus equidens* Wood, 1956a: 144.

• *Hylocurus errans* Blandford, 1898a: 224.

= *Hylocurus denticollis* Schedl, 1976: 71.

• *Hylocurus femineus* Wood, 1959b: 59.

• *Hylocurus flagellatus* Wood, 1971a: 32.

• *Hylocurus flaglerensis* Blackman, 1943c: 345.

• *Hylocurus flechtmanni* Wood, 2007: 316.

• *Hylocurus floridensis* Atkinson, 1989b: 333.

• *Hylocurus giganteus* (Schedl, 1950c: 152) (*Micracis*).

= *Hylocurus pseudoimpar* Schedl, 1954b: 30.

= *Hylocurus interruptus* Schedl, 1959b: 548.

• *Hylocurus harnedi* (Blackman, 1920a: 24) (*Micracis*).

• *Hylocurus hirtellus* (LeConte, 1876: 369) (*Micracis*).

= *Hylocurus crinitus* Blackman, 1943c: 347.

• *Hylocurus impar* Schedl, 1939h: 723.

• *Hylocurus inaequalis* Wood, 1956a: 146.

• *Hylocurus inaequidens* Wood, 2007: 326.

• *Hylocurus incognitus* Atkinson, 2024: 158.

• *Hylocurus incomptus* Wood, 1969a: 44.

• *Hylocurus interpositus* Schedl, 1976: 71.

• *Hylocurus langstoni* (Blackman, 1920a: 26) (*Micracis*).

• *Hylocurus longipennis* Wood, 1979: 141.

• *Hylocurus longulus* (Nunberg, 1956a: 135) (*Micracis longula*).

• *Hylocurus medius* Wood, 1956a: 144.

• *Hylocurus micaceus* Wood, 1984a: 116.

• *Hylocurus microcornis* Wood, 1969a: 45.

• *Hylocurus nodifer* Wood, 2007: 323.

• *Hylocurus nodulus* Wood, 1956a: 141.

• *Hylocurus obtusipennis* Schedl, 1976: 72.

• *Hylocurus parkinsoniae* Blackman, 1922d: 142.

• *Hylocurus parvus* (Wood, 1969a: 46) (*Phloeocleptus*).

• *Hylocurus pilosus* Schedl, 1950c: 151.

• *Hylocurus plaumanni* Wood, 2007: 320.

• *Hylocurus prolatus* Wood, 1982: 228.

• *Hylocurus punctatorugosus* (Schedl, 1948c: 575) (*Micracis*).

• *Hylocurus quadrispinosus* Blackman, 1928b: 191.

• *Hylocurus retusipennis* Blandford, 1898a: 223.

= *Hylocurus dubius* Schedl, 1959b: 547.

• *Hylocurus rivalis* Wood, 1974a: 16.

• *Hylocurus robustus* Schedl, 1952a: 456.

= *Hylocurus stachi* Nunberg, 1958: 487.

• *Hylocurus ruber* Wood, 1956a: 142.

• *Hylocurus rudis* (LeConte, 1876: 369) (*Micracis*).

= *Micracis biorbis* Blackman, 1920a: 22.

= *Hylocurus torosus* Wood, 1971a: 28.

= *Hylocurus binodatus* Wood, 1974a: 17.

• *Hylocurus schwarzi* Blackman, 1928b: 189.

• *Hylocurus scitulus* Wood, 1984a: 116.

• *Hylocurus secus* Wood, 1984a: 116.

• *Hylocurus simplex* Blandford, 1898a: 222.

• *Hylocurus singularis* Wood, 1971a: 31.

• *Hylocurus spadix* Blackman, 1928b: 188.

• *Hylocurus spinifex* Blandford, 1904: 225.

• *Hylocurus subgranulatus* Schedl, 1954b: 31.

• *Hylocurus torresi* Bright, 2019: 77.

• *Hylocurus touroulti* Bright, 2019: 77.

• *Hylocurus trispinatus* Schedl, 1978a: 301.

• *Hylocurus tumidosus* Bright, 2019: 78.

• *Hylocurus vagabundus* Blandford, 1898a: 224.

• *Hylocurus verrucosus* Wood, 1971a: 28.

• *Hylocurus vianai* Schedl, 1952a: 457.

= *Hylocurus intermedius* Schedl, 1952a: 458.

• *Hylocurus villifrons* Wood, 1971a: 30.

• *Hylocurus woytkowskii* Wood, 2007: 324.

#### *Lanurgus* Eggers

16 spp.

• *Lanurgus barbatus* Eggers, 1920c: 37.

• *Lanurgus beaveri* Jordal, 2021f: 96.

• *Lanurgus capensis* Schedl, 1965d: 115.

• *Lanurgus carinatus* Jordal, 2021f: 100.

• *Lanurgus jubatus* Jordal, 2021f: 94.

• *Lanurgus longipilis* (Schedl, 1958b: 558) (*Traglostus*).

• *Lanurgus mattheei* Jordal, 2021f: 98.

• *Lanurgus oleae* Schedl, 1955b: 216.

• *Lanurgus podocarpi* Schedl, 1957b: 216.

= *Traglostus brevisetosus* Schedl, 1957c: 153.

= *Lanurgus podocarpi* Schedl, 1957c: 156.

= *Lanurgus bicolor* Schedl, 1961a: 350.

= *Pseudohylocurus caplandicus* Nunberg, 1961a: 613.

• *Lanurgus pubescens* (Schedl, 1941c: 395) (*Traglostus*).

• *Lanurgus rhusi* Schedl, 1963g: 42.

= *Traglostus spatulatus* Schedl, 1982: 282.

• *Lanurgus spathulatus* Schedl, 1948a: 666.

• *Lanurgus subsulcatus* Browne, 1970: 559.

• *Lanurgus tsitsikammae* Jordal, 2021f: 98.

• *Lanurgus xanthophloeae* Schedl, 1957b: 70.

• *Lanurgus xylographus* Schedl, 1963g: 46.

= *Lanurgus oleaeformis* Schedl, 1970d: 178.

#### *Laximicracis* Jordal

3 spp.

• *Laximicracis convexa* (Schedl, 1965g: 1082) (*Afromicracis*).

• *Laximicracis dubia* (Schedl, 1950b: 21) (*Miocryphalus dubius*).

= *Afromicracis angolensis* Schedl, 1965g: 1081.

• *Laximicracis latipes* Jordal, 2021c: 92.

#### *Leiomicracis* Jordal

1 sp.

• *Leiomicracis aurea* Jordal, 2021b: 254.

#### *Micracis* LeConte

27 spp.

• *Micracis acutipennis* Eichhoff, 1878b: 302.

• *Micracis amplinis* Wood, 1971a: 25.

• *Micracis burgosi* Wood, 1982: 228.

• *Micracis carinula* Wood, 1969a: 40 (E).

• *Micracis carinulata* Wood, 1960b: 62 (E).

• *Micracis cubensis* Blackman, 1928b: 193.

• *Micracis detenta* Wood, 1969a: 42 (E).

• *Micracis evanescens* Wood, 1956a: 152.

• *Micracis exilis* Wood, 1971a: 27.

• *Micracis festiva* Wood, 1969a: 43 (E).

• *Micracis grandis* Schedl, 1948c: 575.

= *Micracis costaricensis* Wood, 1969a: 39.

• *Micracis incerta* Wood, 1971a: 26 (E).

• *Micracis inimica* Wood, 1969a: 42 (E).

• *Micracis lepida* Wood, 1969a: 41 (E).

• *Micracis lignator* Blackman, 1928b: 195.

= *Micracis truncatus* Wood, 1956a: 152.

• *Micracis lignicola* Wood, 1969a: 41 (E).

• *Micracis minima* Wood, 2007: 331 (E).

• *Micracis ovata* Wood, 1956a: 150 (E).

• *Micracis senta* Wood, 1971a: 28 (E).

• *Micracis suturalis* LeConte, 1868: 165.

= *Micracis aculeatus* LeConte, 1868: 165.

= *Micracis meridianus* Blackman, 1920a: 29.

• *Micracis swainei* Blackman, 1920a: 32.

= *Micracis populi* Swaine in: Blackman 1920a: 31.

= *Micracis pygmaeus* Schedl, 1948c: 577.

= *Micracis robustus* Schedl, 1948c: 576.

= *Micracis photophilus* Wood, 1956a: 149.

• *Micracis torus* Wood, 1971a: 26.

• *Micracis tribulata* Wood, 1969a: 40 (E).

• *Micracis tropica* Wood, 2007: 331 (E).

• *Micracis unicornis* Wood, 1969a: 42.

• *Micracis vitula* Wood, 1971a: 27 (E).

• *Micracis wataensis* Petrov, 2016: 2.

#### *Micracisella* Blackman

20 spp.

• *Micracisella adnata* Wood, 1971a: 24.

• *Micracisella divaricata* Wood, 1969a: 39.

• *Micracisella hondurensis* Wood, 1956b: 233.

• *Micracisella knulli* (Blackman, 1943c: 348) (*Micracis*).

• *Micracisella mimetica* Wood, 1974a: 15.

• *Micracisella monadis* Wood, 1969a: 36.

• *Micracisella nanula* (LeConte, 1876: 368) (*Micracis*).

• *Micracisella nigra* Wood, 1956b: 232.

• *Micracisella nigrella* Wood, 1969a: 37.

• *Micracisella nitidula* Wood, 1969a: 37.

• *Micracisella ocellata* Wood, 1974a: 16.

• *Micracisella opacicollis* (LeConte, 1878b: 625) (*Micracis*).

= *Micracis asperulus* LeConte, 1878b: 626.

• *Micracisella opacithorax* (Schedl, 1940a: 340) (*Micracis*).

• *Micracisella scitula* Wood, 1969a: 37.

• *Micracisella serjaniae* Wood, 1971a: 25.

• *Micracisella similis* Wood, 1969a: 38.

• *Micracisella squamatula* Wood, 1969a: 38.

• *Micracisella striata* Wood, 1956b: 231.

• *Micracisella subnitida* Blackman, 1943c: 350.

• *Micracisella vescula* Wood, 1969a: 38.

#### *Microlanurgus* Jordal

2 spp.

• *Microlanurgus ater* Jordal, 2021b: 267.

• *Microlanurgus bicolor* Jordal, 2021b: 266.

#### *Neomicracis* Jordal

1 sp.

• *Neomicracis squamigera* Jordal, 2021b: 260.

#### *Parathysanoes* Bright

1 sp.

• *Parathysanoes absonus* Bright, 2019: 82.

#### *Phloeocleptus* Wood

8 spp.

• *Phloeocleptus ardis* Wood, 1981: 124.

• *Phloeocleptus atkinsoni* Wood, 1981: 124.

= *Phloeocleptus cristatus* Wood, 1981: 125.

• *Phloeocleptus caudatus* Wood, 1956a: 147.

• *Phloeocleptus lorenzfischeri* Atkinson, 2023: 9.

• *Phloeocleptus nanulus* Wood, 1969a: 45.

• *Phloeocleptus obscurus* Wood, 1956a: 147.

• *Phloeocleptus plagiatus* Wood, 1969a: 45.

• *Phloeocleptus spicatus* Wood, 1981: 125.

#### *Phloeocurus* Wood

1 sp.

• *Phloeocurus africanus* (Schedl, 1958a: 875) (*Hylocurus*).

#### *Pseudolanurgus* Jordal

5 spp.

• *Pseudolanurgus asperatus* Jordal, 2021h: 594.

• *Pseudolanurgus bugekeae* (Schedl, 1957b: 69) (*Hylocurus*).

• *Pseudolanurgus harunganae* (Schedl, 1961g: 140) (*Micracis*).

= *Lanurgus cribrellus* Schedl, 1965f: 67.

• *Pseudolanurgus minutissimus* (Schedl, 1961e: 139) (*Lanurgus*).

• *Pseudolanurgus mystax* Jordal, 2021h: 597.

#### *Pseudomicracis* Eggers

12 spp.

• *Pseudomicracis atra* Jordal, 2022: 336.

• *Pseudomicracis coronata* Jordal, 2022: 330.

• *Pseudomicracis difficilis* (Schedl, 1965f: 64) (*Micracis*).

• *Pseudomicracis dispar* (Schedl, 1961g: 142) (*Micracidendron*).

• *Pseudomicracis elsae* Eggers, 1920c: 36.

• *Pseudomicracis lauricola* Jordal, 2022: 335.

• *Pseudomicracis madagascariensis* (Schedl, 1961g: 139) (*Micracis*).

• *Pseudomicracis pennata* (Schedl, 1965f: 63) (*Micracis pennatus*).

• *Pseudomicracis pilosa* Jordal, 2022: 333.

• *Pseudomicracis tomicoides* (Schedl, 1961g: 141) (*Micracidendron*).

• *Pseudomicracis verrucosa* Jordal, 2022: 330.

• *Pseudomicracis vitrioculata* Jordal, 2022: 333.

#### *Pseudothysanoes* Blackman

111 spp.

• *Pseudothysanoes abbreviatus* (Schedl, 1954b: 26) (*Cryptocleptes*).

= *Neoglostatus squamosus* Schedl, 1978a: 300.

• *Pseudothysanoes acaciae* (Blackman, 1943c: 362) (*Cryptocleptes*).

• *Pseudothysanoes acacicolens* Wood, 1969a: 23.

• *Pseudothysanoes acares* (Wood, 1969a: 32) (*Cryptulocleptus*).

• *Pseudothysanoes acutus* Bright, 2019: 86.

• *Pseudothysanoes amassius* Wood, 1969a: 25.

• *Pseudothysanoes amoenus* Bright, 2019: 87.

• *Pseudothysanoes aquilus* (Wood, 1969a: 32) (*Cryptulocleptus*).

• *Pseudothysanoes arbuti* (Wood, 1969a: 30) (*Cryptulocleptus*).

• *Pseudothysanoes argentiniae* (Schedl, 1958j: 43) (*Bostrichips*).

• *Pseudothysanoes atomus* Wood, 1980b: 355.

• *Pseudothysanoes bartoni* Bruck, 1936: 32.

• *Pseudothysanoes bellus* (Schedl, 1954b: 28) (*Cryptocleptes*).

• *Pseudothysanoes brunneus* Wood, 1971b: 72.

• *Pseudothysanoes bullatus* Wood, 1969a: 28.

• *Pseudothysanoes caribbeanensis* Bright, 2019: 87.

• *Pseudothysanoes caritus* (Wood, 1969a: 30) (*Cryptulocleptus*).

• *Pseudothysanoes colombianus* (Blackman, 1943c: 361) (*Cryptocleptes*).

• *Pseudothysanoes concentralis* Wood, 1975a: 27.

• *Pseudothysanoes coniferae* (Wood, 1960b: 64) (*Aphanocleptus*).

• *Pseudothysanoes contrarius* Wood, 1974a: 14.

• *Pseudothysanoes coracinus* Wood, 1969a: 29.

• *Pseudothysanoes costatus* Wood, 1956b: 236.

• *Pseudothysanoes cracentis* Bright, 2019: 88.

• *Pseudothysanoes crassinus* Wood, 1969a: 29.

• *Pseudothysanoes cuspidis* Wood, 1971a: 22.

• *Pseudothysanoes dimorphus* (Schedl, 1954b: 25) (*Cryptocleptes*).

• *Pseudothysanoes dislocatus* (Blackman, 1920a: 51) (*Cryptocleptes*).

• *Pseudothysanoes excavatus* (Wood, 1969a: 33) (*Cryptulocleptus*).

• *Pseudothysanoes fimbriatus* Wood, 1982: 229.

• *Pseudothysanoes frondicolens* Wood, 1971b: 73.

• *Pseudothysanoes funebris* Wood, 1969a: 27.

• *Pseudothysanoes funereus* Wood, 1971a: 21.

• *Pseudothysanoes furvatus* Wood, 1969a: 26.

• *Pseudothysanoes furvescens* Wood, 1971a: 21.

• *Pseudothysanoes furvus* Wood, 1969a: 26.

• *Pseudothysanoes graniticus* Wood, 1971a: 21.

• *Pseudothysanoes granulatus* Bright, 2019: 89.

• *Pseudothysanoes guadeloupensis* Bright, 2019: 89.

• *Pseudothysanoes guevinae* (Schedl, 1966a: 45) (*Bostrichips*).

• *Pseudothysanoes heliura* Wood, 1956b: 237.

• *Pseudothysanoes hopkinsi* Blackman, 1928b: 200.

• *Pseudothysanoes huachucae* Blackman, 1943c: 355.

• *Pseudothysanoes incertissimus* Bright, 2019: 90.

• *Pseudothysanoes insularis* (Blackman, 1943c: 359) (*Cryptocleptes*).

• *Pseudothysanoes isolatus* Bright & Peck, 1998: 238.

• *Pseudothysanoes kashmiricus* Buhroo & Knížek, 2020: 142 (E).

• *Pseudothysanoes lautus* Bright, 2019: 92.

• *Pseudothysanoes lecontei* Blackman, 1920a: 4.

• *Pseudothysanoes leechi* Wood, 1980b: 356.

• *Pseudothysanoes leptus* Bright, 2019: 92.

• *Pseudothysanoes magnispinatus* Bright & Torres, 2006: 399.

• *Pseudothysanoes mancus* Wood, 1969a: 24.

• *Pseudothysanoes mandibularis* Wood, 1984a: 118.

• *Pseudothysanoes marginatus* Bright, 2019: 94.

• *Pseudothysanoes masneri* Bright, 2019: 95.

• *Pseudothysanoes mendicus* (Wood, 1969a: 31) (*Cryptulocleptus*).

• *Pseudothysanoes minor* (Blackman, 1928b: 207) (*Cryptocleptes*).

• *Pseudothysanoes minulus* (Wood, 1956b: 238) (*Cryptocleptes*).

• *Pseudothysanoes minutissimus* Bright, 2019: 96.

• *Pseudothysanoes mirus* (Wood, 1969a: 32) (*Cryptulocleptus*).

• *Pseudothysanoes modestus* (Murayama, 1940: 236) (*Cryphalus*).

= *Gretschkinia mongolica* Sokanovskiy, 1959b: 277.

• *Pseudothysanoes mucronatus* (Wood, 1969a: 34) (*Cryptulocleptus*).

• *Pseudothysanoes multispinatus* Wood, 1957: 109.

• *Pseudothysanoes multispinosus* Wood, 2007: 307.

• *Pseudothysanoes muricatus* Bright, 2019: 97.

• *Pseudothysanoes murilloi* (Blackman, 1943c: 360) (*Cryptocleptes*).

• *Pseudothysanoes obesus* (Wood, 1969a: 33) (*Cryptulocleptus*).

• *Pseudothysanoes peniculus* Wood, 1969a: 28.

• *Pseudothysanoes perexiguus* Bright, 2019: 97.

• *Pseudothysanoes perseae* Wood, 1981: 125.

• *Pseudothysanoes phoradendri* Blackman, 1928b: 202.

• *Pseudothysanoes pini* Wood, 1982: 229.

• *Pseudothysanoes plaumanni* (Schedl, 1951c: 106) (*Cryptocleptes*).

• *Pseudothysanoes plumalis* Wood, 1969a: 29.

• *Pseudothysanoes pumilus* (Wood, 1969a: 31) (*Cryptulocleptus*).

• *Pseudothysanoes punctatus* (Wood, 1980b: 355) (*Phloeocleptus*).

• *Pseudothysanoes quercicolens* Wood, 1956b: 235.

• *Pseudothysanoes quercinus* (Wood, 1969a: 30) (*Cryptulocleptus*).

• *Pseudothysanoes querneus* Wood, 1971a: 20.

• *Pseudothysanoes recavus* Wood, 1974a: 14.

• *Pseudothysanoes rigidus* (LeConte, 1876: 362) (*Cryphalus*).

= *Pseudothysanoes drakei* Blackman, 1920a: 48.

• *Pseudothysanoes securigerus* (Blackman, 1943c: 364) (*Chalcohyus*).

• *Pseudothysanoes securus* Wood, 1977a: 216.

• *Pseudothysanoes sedulus* Blackman, 1928b: 204.

= *Pseudothysanoes barberi* Blackman, 1928b: 206.

= *Pseudothysanoes gambetti* Blackman, 1928b: 205.

• *Pseudothysanoes simplex* Wood, 1984a: 118.

• *Pseudothysanoes smithi* Bright, 2019: 99.

• *Pseudothysanoes spicatus* (Wood, 1969a: 33) (*Cryptulocleptus*).

• *Pseudothysanoes spinatulus* Wood, 1984b: 228 (RN).

= *Pseudothysanoes spinatus* Wood, 1956a: 154.

= *Pseudothysanoes spinatifer* Wood, 1988a: 32 (RN).

• *Pseudothysanoes spinatus* (Schedl, 1952i: 21) (*Bostrichips*).

• *Pseudothysanoes spinura* Wood, 1959b: 58.

• *Pseudothysanoes squameus* Wood, 1984a: 118.

• *Pseudothysanoes subpilosus* (Wood, 1956b: 238) (*Cryptocleptes*).

• *Pseudothysanoes subulatus* (Wood, 1969a: 34) (*Cryptulocleptus*).

• *Pseudothysanoes tecomi* Petrov, 2016: 5.

• *Pseudothysanoes tenellus* Wood, 1971a: 23.

• *Pseudothysanoes thomasi* Wood, 1967a: 38.

• *Pseudothysanoes tresmariae* (Schedl, 1956a: 32) (*Hylocurus*).

• *Pseudothysanoes truncatus* Wood, 1984a: 119.

• *Pseudothysanoes trunculus* Bright, 2019: 100.

• *Pseudothysanoes tumidulus* Wood, 1975a: 28.

• *Pseudothysanoes turnbowi* Wood, 1977a: 217.

• *Pseudothysanoes unimodus* (Schedl, 1959b: 549) (*Cryptocleptes*).

• *Pseudothysanoes vallatus* Wood, 1969a: 24.

• *Pseudothysanoes verdicus* Wood, 1969a: 27.

• *Pseudothysanoes verticillus* Wood, 1971a: 22.

• *Pseudothysanoes vesculus* Wood, 1969a: 24.

• *Pseudothysanoes viscicolens* Wood, 1969a: 25.

• *Pseudothysanoes viscivorus* Wood, 1969a: 25.

• *Pseudothysanoes vorontsovi* Petrov, 2009: 129.

• *Pseudothysanoes yuccae* (Wood, 1956b: 239) (*Cryptocleptes*).

= *Pseudothysanoes yuccavorus* Wood, 1971a: 23.

#### *Stenoclyptus* Blackman

2 spp.

• *Stenoclyptus ruficollis* Wood, 1956b: 239.

• *Stenoclyptus sulcatus* (Bruck, 1936: 33) (*Pseudothysanoes*).

= *Stenoclyptus ceanothi* Blackman, 1943c: 358.

= *Stenoclyptus rhois* Blackman, 1943c: 357.

#### *Stevewoodia* Bright

2 spp.

• *Stevewoodia atomus* Bright, 2019: 102.

• *Stevewoodia minutum* Bright, 2010: 45.

#### *Thysanoes* LeConte

14 spp.

• *Thysanoes adonis* Wood, 1969a: 36.

• *Thysanoes berbericolens* Wood, 1971b: 73.

• *Thysanoes berchemiae* Blackman, 1920a: 44 (E).

• *Thysanoes epicaris* Wood, 1969a: 35.

• *Thysanoes fimbricornis* LeConte, 1876: 370.

• *Thysanoes granulifer* Wood, 1974a: 15.

• *Thysanoes inornatus* Wood, 1971a: 24.

• *Thysanoes lobdelli* Blackman, 1920a: 43.

• *Thysanoes neotropicalis* Wood, 1969a: 35.

• *Thysanoes pallens* Wood, 1956b: 234.

• *Thysanoes subsulcatus* Wood, 1969a: 35.

• *Thysanoes texanus* Blackman, 1943c: 352.

= *Thysanoes retamae* Blackman, 1943c: 354.

= *Thysanoes vachelliae* Blackman, 1943c: 353.

= *Thysanoes mexicanus* Wood, 1956b: 234.

• *Thysanoes tuberculatus* Wood, 1975a: 29.

• *Thysanoes xylophagus* Blackman, 1928b: 198.

#### *Traglostus* Schedl

1 sp.

• *Traglostus exornatus* Schedl, 1938a: 454.

### PHLOEOSININI Nüsslin

15 genera, 241 spp. (2 spp. with 4 sspp.).

#### *Asiophilus* Jordal

3 spp.

• *Asiophilus bukiticola* Smith, Beaver & Cognato in: Smith et al. 2025: 30.

• *Asiophilus macropunctatus* Jordal, 2010: 149.

• *Asiophilus phloeosinoides* (Browne, 1966: 243) (*Phloeoditica*).

#### *Carphotoreus* Wood

1 sp.

• *Carphotoreus alni* (Bright, 1972c: 1492) (*Chaetophloeus*).

#### *Catenophorus* Nunberg

1 sp.

• *Catenophorus congonus* Nunberg, 1956b: 197.

#### *Chramesus* LeConte

100 spp.

• *Chramesus aberrans* Schedl, 1951c: 90.

• *Chramesus acacicolens* Wood, 1969a: 3.

• *Chramesus advena* Schedl, 1951c: 91.

• *Chramesus annectens* (Wood, 1956c: 254) (*Prochramesus*).

• *Chramesus aquilus* Wood, 1974a: 10.

• *Chramesus argentinae* Wood, 2007: 173.

• *Chramesus argentinensis* Schedl, 1952a: 456.

• *Chramesus asperatus* Schaeffer, 1908: 220.

= *Chramesus gibber* Blackman, 1938a: 541.

• *Chramesus aspericollis* Schedl, 1938f: 23.

• *Chramesus asperulus* Schedl, 1978a: 294.

• *Chramesus atkinsoni* Wood, 1981: 123.

• *Chramesus atlanticus* Bright & Torres, 2006: 395.

• *Chramesus badius* Schedl, 1951c: 88.

• *Chramesus bicolor* Wood, 1967b: 91.

• *Chramesus bispinus* Wood, 1982: 225.

• *Chramesus bolivianus* Schedl, 1973a: 370.

• *Chramesus brasiliensis* Nunberg, 1962: 224.

• *Chramesus cecropiae* Wood, 1969a: 5.

• *Chramesus chapuisi* LeConte, 1876: 375.

= *Chramesus wisteriae* Wood, 1974a: 11.

• *Chramesus corniger* Wood, 1974a: 8.

• *Chramesus corumbensis* Eggers, 1951: 145.

• *Chramesus crenatus* Wood, 1956c: 257.

• *Chramesus cylindricus* Schedl, 1952a: 455.

• *Chramesus demissus* Wood, 1967b: 93.

• *Chramesus dentatus* Schaeffer, 1908: 221.

= *Chramesus barbatus* Eggers, 1930a: 169.

• *Chramesus dentellus* Wood, 2007: 178.

• *Chramesus denticulatus* Wood, 1971a: 6.

• *Chramesus deplanatus* Eggers, 1940a: 124.

• *Chramesus disparilis* Wood, 1974a: 9.

• *Chramesus editus* (Bright, 1972c: 1495) (*Prochramesus*).

• *Chramesus erinaceus* Schedl, 1967d: 7.

• *Chramesus exilis* Wood, 1983: 653.

• *Chramesus exul* Wood, 1983: 653.

• *Chramesus flechtmanni* Petrov & Mandelshtam, 2011: 273.

• *Chramesus globosus* Hagedorn, 1909: 742.

= *Chramesus eurypterus* Schedl, 1963f: 214.

• *Chramesus gracilis* Wood, 1969a: 2.

• *Chramesus granulatus* (Eggers, 1928a: 94) (*Pagiocerus*).

• *Chramesus granulipennis* Schedl, 1959b: 547.

• *Chramesus hicoriae* LeConte, 1868: 168.

= *Rhopalopleurus lecontei* Chapuis, 1869: 47.

• *Chramesus hylurgoides* Schedl, 1963f: 214.

• *Chramesus impolitus* Wood, 1971a: 6.

• *Chramesus imporcatus* Wood, 1971a: 5.

• *Chramesus incomptus* Wood, 1967b: 90.

• *Chramesus ingens* Wood, 1969a: 3.

• *Chramesus karavaevi* Petrov & Flechtmann, 2020: 332.

• *Chramesus lepidotus* Bright, 2019: 38.

• *Chramesus longus* Petrov & Flechtmann, 2020: 333.

• *Chramesus luteus* Wood, 2007: 172.

• *Chramesus macrocornis* Wood, 1971a: 3.

• *Chramesus maieri* Bright, 2019: 39.

• *Chramesus marginatus* Wood, 1974a: 11.

• *Chramesus microporosus* Wood, 1974a: 10.

• *Chramesus mimosae* Blackman, 1938a: 544.

• *Chramesus minor* Eggers, 1951: 144.

• *Chramesus minulus* Wood, 1969c: 126.

• *Chramesus neglectus* Schedl, 1978a: 296.

• *Chramesus nobilis* Petrov & Flechtmann, 2020: 335.

• *Chramesus opacicollis* Eggers, 1940a: 124.

= *Chramesus opacicollis nitidus* Eggers, 1940a: 125.

= *Chramesus brevisetosus* Bright, 1972a: 40.

• *Chramesus orinocensis* Wood, 1971a: 4.

• *Chramesus ovalis* Schedl, 1952a: 454.

• *Chramesus palearis* Bright, 2019: 41.

• *Chramesus parcus* Wood, 1971a: 6.

• *Chramesus peniculus* Wood, 1971a: 8.

• *Chramesus periosus* Wood, 1969a: 5.

• *Chramesus peruanus* Schedl, 1961h: 223.

• *Chramesus phloeosinites* Schedl, 1951c: 89.

• *Chramesus phloeotriboides* Schedl, 1958j: 41.

• *Chramesus priscus* Wood, 1971a: 7.

• *Chramesus pumilus* (Chapuis, 1869: 47) (*Rhopalopleurus*).

= *Chramesus tumidulus* Blandford, 1897: 170.

= *Chramesus panamensis* Blackman, 1943d: 391.

= *Chramesus mexicanus* Schedl, 1949b: 264.

• *Chramesus punctatus* Wood, 1967b: 94.

• *Chramesus quadridens* Wood, 1956c: 256.

• *Chramesus robustus* Schedl, 1949b: 264.

• *Chramesus rotundatus* (Chapuis, 1869: 47) (*Rhopalopleurus*).

= *Dendrosinus bonnairei* Reitter, 1895: 45.

• *Chramesus scabiosus* Bright, 2019: 43.

• *Chramesus schoenmanni* Petrov & Mandelshtam, 2011: 274.

• *Chramesus securus* Wood, 1983: 653.

• *Chramesus secus* Wood, 1969a: 4.

• *Chramesus setiger* Schedl, 1951c: 92.

• *Chramesus setosus* Wood, 1960b: 61.

• *Chramesus signatipennis* Schedl, 1962d: 97.

• *Chramesus simplicis* Wood, 1971a: 3.

• *Chramesus solicitatus* Wood, 1971a: 8.

• *Chramesus spinosus* Brèthes, 1921: 167.

= *Chramesus cristatus* Schedl, 1963f: 213.

= *Chramesus dentipes* Schedl, 1978a: 295.

• *Chramesus squamosus* Bright, 2019: 44.

• *Chramesus striatus* Eggers, 1943d: 344.

• *Chramesus strigatus* Wood, 1960b: 62 (RN).

= *Chramesus striatus* Wood, 1956c: 256.

• *Chramesus strigilis* Wood, 1971a: 4.

• *Chramesus subopacus* Schaeffer, 1908: 221.

= *Chramesus canus* Blackman, 1938a: 541.

• *Chramesus subtuberculatus* Eggers, 1951: 146.

• *Chramesus tibialis* Wood, 1983: 654.

• *Chramesus tuberculatus* (Chapuis, 1869: 47) (*Rhopalopleurus*).

• *Chramesus unespi* Petrov & Flechtmann, 2020: 337.

• *Chramesus unicornis* Wood, 1969a: 4.

• *Chramesus variabilis* Wood, 1974a: 9.

• *Chramesus variegatus* Eggers, 1943d: 345.

• *Chramesus vastus* Wood, 1967b: 92.

• *Chramesus vinealis* Wood, 1971a: 7.

• *Chramesus vitiosus* Wood, 1969c: 125.

• *Chramesus xalapae* Atkinson, 1989a: 58.

• *Chramesus xylophagus* Wood, 1956c: 255.

#### *Cladoctonus* Strohmeyer

18 spp.

• *Cladoctonus affinis* Strohmeyer, 1911a: 17.

= *Cladoctonus eggersi* Wichmann, 1911c: 174.

• *Cladoctonus amanicus* Eggers, 1920b: 118.

• *Cladoctonus atrocis* Wood, 1974a: 12.

• *Cladoctonus banosus* (Eggers, 1923b: 141) (*Hoplites*).

= *Phloeosinus nonseptis* Schedl, 1942b: 173.

• *Cladoctonus brevisetosus* Bright, 1972a: 46.

• *Cladoctonus contractus* Schedl, 1975a: 340.

• *Cladoctonus corumbensis* (Eggers, 1951: 149) (*Hoplites*).

= *Hoplitophthorus boliviae* Wood, 1961c: 106.

• *Cladoctonus cubensis* (Wood, 1961c: 107) (*Hoplitophthorus*).

• *Cladoctonus elongatus* Schedl, 1967c: 222.

• *Cladoctonus interruptus* (Eggers, 1940a: 126) (*Hoplites*).

= *Hoplitophthorus sentus* Wood, 1961a: 3.

• *Cladoctonus major* (Eggers, 1940a: 125) (*Hoplites*).

• *Cladoctonus minor* Bright, 2019: 48.

• *Cladoctonus natalensis* Eggers, 1936c: 34.

• *Cladoctonus peckorum* Bright, 2019: 49.

• *Cladoctonus similis* Nunberg, 1969: 391.

• *Cladoctonus torosus* Bright, 2019: 49.

• *Cladoctonus tuberculatus* Schedl, 1973a: 369.

• *Cladoctonus tuberosus* Bright, 2019: 51.

#### *Cortisinus* Wood

1 sp.

• *Cortisinus lobatus* (Eggers, 1943d: 374) (*Sternobothrus*).

#### *Dendrosinus* Chapuis

7 spp.

• *Dendrosinus ater* Eggers, 1930a: 167.

= *Dendrosinus paraguayensis* Eggers, 1930a: 168.

= *Dendrosinus hirsutus* Schedl, 1958j: 38.

• *Dendrosinus bourreriae* Schwarz in: Schwarz and Barber 1920: 225.

= *Dendrosinus lima* Eggers, 1930a: 166.

• *Dendrosinus globosus* (Eichhoff, 1868b: 149) (*Hylesinus*).

• *Dendrosinus mexicanus* Wood, 1983: 656.

• *Dendrosinus puncticollis* Blandford, 1897: 156.

• *Dendrosinus transversalis* Blandford, 1897: 156.

• *Dendrosinus vittifrons* Blandford, 1897: 156.

#### *Hyledius* Sampson

23 spp.

• *Hyledius armatus* (Schedl, 1936c: 23) (*Phloeosinopsis*).

= *Phloeosinus spinifer* Browne, 1963b: 53 (RN).

• *Hyledius birmanus* (Eggers, 1923b: 138) (*Phloeosinus*).

• *Hyledius brunneus* (Browne, 1980a: 373) (*Phloeosinus*).

• *Hyledius canus* (Browne, 1972: 20) (*Phloeosinus*).

• *Hyledius ceylonicus* (Schedl, 1959i: 473) (*Phloeosinus*).

• *Hyledius concinnulus* (Walker, 1859: 261) (*Hylurgus*).

= *Olonthogaster nudifrons* Motschulsky, 1866: 402.

• *Hyledius cribratus* (Blandford, 1896e: 198) (*Phloeosinus*).

= *Phloeosinus malayensis* Schedl, 1936c: 22.

= *Phloeosinus australis* Schedl, 1938g: 36.

= *Phloeosinus tuberculosus* Browne, 1970: 544.

• *Hyledius detersus* (Chapuis, 1869: 38) (*Phloeosinus*).

• *Hyledius imitans* (Eggers, 1927b: 75) (*Phloeosinus*).

• *Hyledius jiri* (Wood, 1988c: 198) (*Olonthogaster*).

• *Hyledius kesiyae* (Browne in: Beaver and Browne 1975: 292) (*Phloeosinus*).

• *Hyledius kinabaluensis* (Bright, 1989: 80) (*Phloeosinus*).

• *Hyledius nitidicollis* (Motschulsky, 1866: 401) (*Olonthogaster*).

= *Hyledius asper* Sampson, 1921: 35.

= *Phloeosinus latus* Eggers, 1923b: 138.

= *Phloeosinus vagans* Eggers, 1923b: 139.

= *Phloeosinus philippinensis* Schedl, 1934a: 91.

• *Hyledius papuanus* (Schedl, 1936e: 521) (*Phloeosinus*).

• *Hyledius penangensis* (Schedl, 1975i: 295) (*Phloeosinus*).

• *Hyledius phyllocladus* (Bright, 1989: 81) (*Phloeosinus*).

• *Hyledius regalis* (Wood, 1988c: 198) (*Olonthogaster*).

• *Hyledius solomonicus* (Browne, 1980d: 494) (*Phloeosinus*).

• *Hyledius subcarinatus* (Schedl, 1942b: 173) (*Phloeosinus*).

• *Hyledius sumatranus* (Eggers, 1923b: 140) (*Phloeosinus*).

• *Hyledius takahashii* (Browne, 1981b: 600) (*Phloeosinus*).

• *Hyledius vilis* (Blandford, 1896e: 199) (*Phloeosinus*).

= *Hylurgulus sumatranus* Eggers, 1927e: 393.

= *Phloeosinus hylurgulus* Eggers, 1940c: 62 (RN).

= *Phloeosinus borneensis* Schedl, 1942b: 172.

= *Phloeosinus nanus* Browne in: Beaver and Browne 1975: 293.

• *Hyledius xanthophylli* (Browne, 1949: 896) (*Phloeosinus*).

#### *Hyleops* Schedl

1 sp.

• *Hyleops glabratus* Schedl, 1938g: 36.

#### *Microditica* Jordal

1 sp.

• *Microditica uniseriata* Jordal, 2010: 153.

#### *Phloeocranus* Schedl

1 sp.

• *Phloeocranus bruchoides* Schedl, 1942d: 8.

= *Diamerides litseae* Browne, 1949: 894.

#### *Phloeoditica* Schedl

2 spp.

• *Phloeoditica curta* (Eggers, 1925: 155) (*Kissophagus curtus*).

• *Phloeoditica elegans* (Schedl, 1962f: 190) (Phloeoditica*).

#### *Phloeosinopsioides* Schedl

3 spp.

• *Phloeosinopsioides formosanus* (Schedl, 1935e: 479) (*Xylechinus*).

= *Phloeosinopsis triseriatus* Schedl, 1964k: 297.

= *Xylechinus papuanus* Schedl, 1970g: 128.

• *Phloeosinopsioides leai* (Schedl, 1936e: 524) (*Xylechinus*).

• *Phloeosinopsioides pumilus* Wood, 1985: 274 (*Phloeosinopsoides*) (E).

#### *Phloeosinus* Chapuis

68 spp. (2 spp. with 4 sspp.).

• *Phloeosinus abietis* Tsai & Yin, 1964: 92.

• *Phloeosinus acatayi* Schedl, 1958g: 33.

= *Phloeosinus cedri libani* Balachowsky, 1969: 649.

• *Phloeosinus antennatus* Swaine, 1924b: 146.

= *Phloeosinus pseudotsugae* Chamberlin, 1955: 117.

• *Phloeosinus arisanus* Niisima, 1942: 73.

• *Phloeosinus arizonicus* Blackman, 1942a: 424.

• *Phloeosinus armatus* Reitter, 1887: 192.

= *Phloeosinus andresi* Eggers, 1927a: 120.

• *Phloeosinus aubei* (Perris, 1855: lxxviii) (*Hylesinus*).

= *Phloeophthorus praenotatus* Gredler, 1866: 370.

= *Phloeosinus transcaspicus* Semenov, 1903: 79.

= *Phloeosinus hercegovinensis* Eggers, 1922b: 120.

= *Phloeosinus schumensis* Eggers, 1922c: 166.

• *Phloeosinus baumanni* Hopkins, 1906: 79.

• *Phloeosinus camphoratus* Tsai & Yin, 1964: 93.

• *Phloeosinus canadensis* Swaine, 1917: 8.

• *Phloeosinus cedri* Brisout de Barneville, 1883: 146.

= *Phloeosinus cedri maura* Balachowsky, 1969: 649.

• *Phloeosinus corneyanus* Knížek & Tshering, 2024: 590.

• *Phloeosinus cristatus* (LeConte, 1868: 169) (*Hylesinus*).

= *Phloeosinus chiricahua* Blackman, 1942a: 444.

• *Phloeosinus cupressi* Hopkins, 1903a: 135.

= *Phloeosinus nitidus* Swaine, 1924b: 145.

= *Phloeosinus blackwelderi* Blackman, 1943d: 397.

• *Phloeosinus deleoni* Blackman, 1942a: 454.

• *Phloeosinus dentatus* (Say, 1826: 258) (*Hylurgus*).

= *Dendroctonus graniger* Eichhoff, 1868b: 147.

= *Dendroctonus haagi* Eichhoff, 1868b: 148.

= *Phloeosinus enixus* Blackman, 1921: 5.

• *Phloeosinus dubiosus* Schedl, 1972e: 145.

• *Phloeosinus frontalis* Bruck, 1933a: 55.

= *Phloeosinus granulatus* Bruck, 1936: 33.

• *Phloeosinus fulgens* Swaine, 1924b: 146.

= *Phloeosinus splendens* Blackman, 1942a: 428.

• *Phloeosinus furnissi* Blackman, 1942a: 469.

• *Phloeosinus gifuensis* Murayama, 1954: 190.

• *Phloeosinus gillerforsi* Bright, 1987: 3.

• *Phloeosinus granosus* Schedl, 1962f: 191.

• *Phloeosinus granulipennis* Schedl, 1975h: 218.

• *Phloeosinus henschi* Reitter, 1901c: 201.

= *Phloeosinus stoeckleini* Schedl, 1935b: 241.

= *Phloeosinus krimaeus* Eggers, 1937a: 334.

= *Phloeosinus inermis* Nunberg, 1948: 14.

• *Phloeosinus hoferi* Blackman, 1942a: 412.

• *Phloeosinus hopehi* Schedl, 1953a: 23.

• *Phloeosinus hoppingi* Swaine, 1915b: 364.

= *Phloeosinus woodi* Bright, 1966: 296.

• *Phloeosinus jubatus* Sampson, 1919: 112.

• *Phloeosinus keeni* Blackman, 1942a: 414.

• *Phloeosinus kiushuensis* Murayama, 1955: 93.

• *Phloeosinus kumamotoensis* Murayama, 1955: 91.

• *Phloeosinus laricionis* Faccoli & Sidoti, 2013: 93.

• *Phloeosinus lewisi* Chapuis in: Chapuis and Eichhoff 1876: 198.

= *Phloeosinus minutus* Blandford, 1894c: 71.

• *Phloeosinus machilus* (Schedl, 1959e: 173) (*Hylesinus*).

= *Phloeosinus cinnamomi* Tsai & Yin, 1964: 94.

• *Phloeosinus metasequoiae* Ning in: Ning et al. 2025: 163.

• *Phloeosinus neotropicus* Schedl, 1939g: 12.

• *Phloeosinus osumiensis* Murayama, 1955: 90.

• *Phloeosinus pacificus* Wood, 1960a: 15.

• *Phloeosinus palearis* Wood, 1969a: 2.

• *Phloeosinus perlatus* Chapuis in: Chapuis and Eichhoff 1876: 198.

• *Phloeosinus pertuberculatus* Eggers, 1939a: 115.

• *Phloeosinus pfefferi* Knížek, 1994: 124.

• *Phloeosinus phoebe* Wood, 1988c: 199.

• *Phloeosinus pini* Swaine, 1915b: 362.

= *Phloeosinus piceae* Swaine, 1934: 205.

= *Phloeosinus alaskanus* Blackman, 1942a: 409.

• *Phloeosinus podocarpi* Browne, 1986b: 663.

• *Phloeosinus pulchellus* Blandford, 1894c: 69.

= *Phloeosinus dubius* Blandford, 1894c: 70.

= *Phloeosinus izuensis* Nobuchi, 1959a: 9.

• *Phloeosinus punctatus* LeConte, 1876: 382.

= *Phloeosinus rubicundulus* Swaine, 1924b: 144.

= *Phloeosinus buckhorni* Blackman, 1942a: 432.

= *Phloeosinus chamberlini* Blackman, 1942a: 470.

= *Phloeosinus kaniksu* Blackman, 1942a: 434.

= *Phloeosinus rusti* Blackman, 1942a: 435.

• *Phloeosinus rudis* Blandford, 1894c: 73.

= *Phloeosinus shotoensis* Murayama, 1955: 88.

• *Phloeosinus sannohensis* Murayama, 1954: 190.

• *Phloeosinus scopulorum neomexicanus* Blackman, 1942a: 460 (*Phloeosinus neomexicanus*).

= *Phloeosinus texanus* Blackman, 1942a: 462.

• *Phloeosinus scopulorum scopulorum* Swaine, 1924b: 148 (*Phloeosinus scopulorum*).

• *Phloeosinus sequoiae* Hopkins, 1903b: 33.

= *Phloeosinus squamosus* Blackman, 1942a: 448.

= *Phloeosinus blackmani* Schedl, 1950e: 36 (RN).

• *Phloeosinus seriatus* Blandford, 1894c: 72.

• *Phloeosinus serratus* (LeConte, 1868: 170) (*Hylesinus*).

= *Phloeosinus utahensis* Swaine, 1915b: 363.

= *Phloeosinus juniperi* Swaine, 1917: 10.

= *Phloeosinus rugosus* Swaine, 1917: 9.

= *Phloeosinus aciculatus* Bruck, 1931: 127.

= *Phloeosinus neotropicus* Schedl, 1939g: 12.

• *Phloeosinus setosus* Bruck, 1933a: 54.

• *Phloeosinus shensi* Tsai & Yin, 1964: 90.

• *Phloeosinus similis* Eggers, 1925: 155.

• *Phloeosinus sinensis* Schedl, 1953a: 23.

• *Phloeosinus spinosus* Blackman, 1942a: 447.

• *Phloeosinus squamulatus* Chapuis, 1869: 39.

• *Phloeosinus swainei* Bruck, 1933a: 56 (RN).

= *Phloeosinus minutus* Swaine, 1917: 9.

• *Phloeosinus tacubayae* Hopkins, 1906: 78.

• *Phloeosinus taxodii taxodii* Blackman, 1922a: 61 (*Phloeosinus taxodii*).

• *Phloeosinus taxodii taxodiicolens* Wood, 1956c: 251 (*Phloeosinus taxodiicolens*).

• *Phloeosinus thujae* (Perris, 1855: lxxvii) (*Hylesinus*).

= *Scolytus impressus* Olivier, 1800a: 12.

= *Dendroctonus juniperi* Döbner in: Döbner 1860: 261.

= *Phloeosinus serrifer* Wichmann, 1916: 12.

= *Phloeosinus prostratus* Peyerimhoff, 1918: 259.

• *Phloeosinus transversarius* Schedl, 1936e: 522.

• *Phloeosinus turkestanicus* Semenov, 1902: 269.

• *Phloeosinus vandykei* Swaine, 1915b: 366.

= *Phloeosinus russus* Swaine, 1924b: 148.

• *Phloeosinus variolatus* Bruck, 1931: 126.

#### *Pseudochramesus* Blackman

11 spp.

• *Pseudochramesus acuteclavatus* (Hagedorn, 1909: 742) (*Chramesus*).

= *Chramesus semibrunneus* Eggers, 1951: 145.

• *Pseudochramesus brasiliensis* Schedl, 1949b: 265.

• *Pseudochramesus costulatus* Blackman, 1939: 91.

• *Pseudochramesus duplosquamosus* Schedl, 1963f: 215.

• *Pseudochramesus golbachi* Wood, 2007: 155.

• *Pseudochramesus harringtoni* Blackman, 1939: 93.

= *Pseudochramesus multiseriatus* Schedl, 1978a: 296.

• *Pseudochramesus jaliscoensis* Wood, 1987: 548.

• *Pseudochramesus manni* Blackman, 1939: 88.

• *Pseudochramesus opacus* Schedl, 1963f: 216.

• *Pseudochramesus setifer* Schedl, 1951c: 93.

= *Pseudochramesus abbreviatus* Schedl, 1951c: 94.

• *Pseudochramesus vianai* Schedl, 1958j: 39.

### PHLOEOTRIBINI Chapuis

3 genera, 109 spp.

#### *Aricerus* Blandford

3 spp.

• *Aricerus analis* Schedl, 1975h: 216.

• *Aricerus chapuisi* Blandford, 1894a: 134.

• *Aricerus eichhoffi* Blandford, 1894a: 135.

= *Hylesinus fici* Lea, 1904: 103.

#### *Dryotomicus* Wood

4 spp.

• *Dryotomicus oenophilis* Cognato & Smith, 2010: 54.

• *Dryotomicus puberulus* (Chapuis, 1869: 46) (*Dryotomus*).

• *Dryotomicus tuberculatus* (Eggers, 1943d: 348) (*Dryotomus*).

• *Dryotomicus woodrex* Cognato & Smith, 2010: 58.

#### *Phloeotribus* Latreille

102 spp.

• *Phloeotribus acaciae* (Lea, 1910: 146) (*Phloeophthorus*).

• *Phloeotribus amplus* Wood, 1977b: 390.

• *Phloeotribus argentinensis* (Schedl, 1952a: 447) (*Phthorophloeus*).

• *Phloeotribus asperulus* Eggers, 1943d: 353.

• *Phloeotribus atavus* Wood, 1969a: 6.

• *Phloeotribus atlanticus* Schedl, 1951c: 81.

• *Phloeotribus biguttatus* Blandford, 1897: 169.

• *Phloeotribus brasiliensis* (Schedl, 1951c: 85) (*Phthorophloeus*).

• *Phloeotribus brevicollis* (Kolenati, 1846: 38) (*Hylesinus*).

• *Phloeotribus carinatus* Burgos & Equihua in: Burgos et al. 2003: 342.

= *Phloeotribus ebeneus* Wood, 2007: 141.

• *Phloeotribus caucasicus* Reitter, 1891a: 32.

• *Phloeotribus caymanensis* Bright, 2019: 33.

• *Phloeotribus championi* (Blandford, 1897: 161) (*Eulytocerus*).

= *Eulytocerus substriatus* Schedl, 1935f: 344.

• *Phloeotribus collaris* Chapuis, 1869: 46.

= *Phloeotribus despectus* Schedl, 1966d: 96.

• *Phloeotribus contortus* Schedl, 1973b: 167.

• *Phloeotribus cristatus* (Fauvel, 1889: 71) (*Phloeophthorus*).

= *Phloeophthorus cristatus lineigera* Guillebeau, 1893: 60.

= *Phloeophthorus cristatus mayeti* Guillebeau, 1893: 62.

= *Phloeophthorus cristatus pubifrons* Guillebeau, 1893: 59.

= *Phloeophthorus cristatus sharpi* Guillebeau, 1893: 62.

= *Phloeophthorus helveticus* Guillebeau, 1893: 60.

= *Phloeophthorus latus* Wichmann, 1916: 14.

= *Phloeophthorus geschwindi* Seitner, 1920: 283.

= *Phloeophthorus ovalis* Eggers, 1941d: 120.

= *Phloeotribus pectinicornis* Balachowsky, 1949b: 116.

• *Phloeotribus cylindricus* Schedl, 1951c: 82.

• *Phloeotribus demessus* Blandford, 1897: 165.

= *Phloeotribus tuberculatus* Eggers, 1951: 147.

= *Phloeotribus eggersi* Schedl, 1962a: 487 (RN).

• *Phloeotribus dentifrons* (Blackman, 1921: 3) (*Phthorophloeus*).

• *Phloeotribus destructor* Wood, 1969c: 123.

• *Phloeotribus discrepans* Blandford, 1897: 163.

• *Phloeotribus erinaceus* Schedl, 1973b: 168.

• *Phloeotribus erosus* Schedl, 1951c: 83.

• *Phloeotribus fici* Wood, 1977b: 393.

• *Phloeotribus fraxini* (Eggers, 1920a: 239) (*Phloeophthorus*).

• *Phloeotribus frontalis* (Olivier, 1800a: 13) (*Scolytus*).

= *Phloeophthorus granicollis* Eichhoff, 1868b: 149.

= *Phloeophthorus moriperda* Hopkins, 1906: 77.

• *Phloeotribus furvus* Wood, 1969c: 124.

• *Phloeotribus geminus* Wood, 1983: 657.

• *Phloeotribus harringtoni* Blackman, 1943d: 388.

• *Phloeotribus hebes* Schedl, 1978a: 294.

• *Phloeotribus hercegovinensis* (Seitner, 1920: 284) (*Phloeophthorus*).

• *Phloeotribus hirtellus* Schedl, 1966d: 99.

• *Phloeotribus hirticulus* Wood, 2007: 123.

• *Phloeotribus hirtus* Wood, 1977b: 393.

• *Phloeotribus hispidulus* Eggers, 1934a: 78.

• *Phloeotribus hylurgulus* Schedl, 1959c: 405.

• *Phloeotribus hystrix* Wood, 1969a: 7.

• *Phloeotribus incanus* Wood, 2007: 137.

• *Phloeotribus ingae* Wood, 1977b: 389.

• *Phloeotribus insularis* Eggers, 1940a: 123.

• *Phloeotribus jujuya* Blackman, 1943d: 389.

• *Phloeotribus lecontei* Schedl, 1962a: 487 (RN).

= *Phloeotribus puberulus* LeConte, 1879: 519.

• *Phloeotribus liminaris* (Harris, 1852: 79) (*Tomicus*).

= *Phthorophloeus mississippiensis* Blackman, 1921: 4.

• *Phloeotribus longipilus* Eggers, 1943d: 355.

• *Phloeotribus maroccanus* (Guillebeau, 1896: 152) (*Phloeophthorus*).

• *Phloeotribus maurus* Wood, 1969a: 6.

• *Phloeotribus mayeti* (Guillebeau, 1893: 62) (*Phloeophthorus*).

• *Phloeotribus minor* Wood, 1977b: 391.

• *Phloeotribus muricatus* (Eggers, 1929c: 9) (*Phloeophthorus*).

• *Phloeotribus nanus* Wood, 1974a: 8.

• *Phloeotribus nitidicollis* (Eggers, 1943d: 347) (*Phthorophloeus*).

= *Phloeotribus striatus* Eggers, 1943d: 351.

= *Phloeotribus eggersi* Schedl, 1963d: 60 (RN).

= *Phloeotribus neglectus* Schedl, 1964g: 303 (RN).

= *Phloeotribus levis* Wood, 1977b: 392.

= *Phloeotribus nebulosus* Wood, 1977b: 390.

• *Phloeotribus novateutonicus* (Schedl, 1951c: 84) (*Phthorophloeus*).

• *Phloeotribus nubilus* Blandford, 1897: 163.

• *Phloeotribus opacicollis* Eggers, 1943d: 353.

• *Phloeotribus opimus* Wood, 1969a: 7.

• *Phloeotribus ovatus* (Eggers, 1943d: 347) (*Dryotomus*).

• *Phloeotribus pacificus* Bright, 1982b: 128.

• *Phloeotribus perfoliatus* (Wollaston, 1854: 301) (*Phloeophthorus*).

= *Phloeophthorus abeillei* Guillebeau, 1893: 58.

• *Phloeotribus perniciosus* Wood, 1982: 229.

• *Phloeotribus peyerimhoffi* (Eggers, 1913: 239) (*Phloeophthorus*).

• *Phloeotribus piceae* Swaine, 1911: 220.

• *Phloeotribus picipennis* Eggers, 1943d: 352.

= *Phloeotribus boliviae* Blackman, 1943d: 387.

• *Phloeotribus pilifer* Wood, 2007: 123.

• *Phloeotribus pilula* (Erichson, 1847: 138) (*Hylesinus*).

= *Phloeotribus obliquus* Chapuis, 1869: 45.

= *Phloeotribus obesus* Kirsch, 1875: 283.

= *Phloeotribus manni* Blackman, 1943d: 385.

= *Phloeotribus australis* Schedl, 1953b: 80.

• *Phloeotribus porteri* Bruch, 1914: 25.

• *Phloeotribus profanus* Schedl, 1963f: 212.

• *Phloeotribus pruni* Wood, 1956c: 253.

• *Phloeotribus pseudocristatus* (Pfeffer, 1972: 40) (*Phloeophthorus*).

• *Phloeotribus pubifrons* (Guillebeau, 1893: 59) (*Phloeophthorus*).

= *Phloeophthorus corsicus* Guillebeau, 1893: 60.

= *Phloeophthorus lineigera* Guillebeau, 1893: 60.

= *Phloeophthorus guillebeaui* Reitter, 1913: 35.

= *Comesiella sicula* Del Guercio, 1925b: 210.

• *Phloeotribus quercinus* Wood, 1969c: 123.

• *Phloeotribus remorsus* Wood, 1977b: 391.

• *Phloeotribus rhododactylus* (Marsham, 1802: 58) (*Ips*).

= *Hylesinus spartii* Nördlinger, 1847: 217.

= *Polygraphus tarsalis* Förster, 1849: 383.

= *Hylesinus retamae* Perris, 1864: 300.

= *Phloeophthorus rhododactylus austriacus* Guillebeau, 1893: 58.

= *Phloeophthorus rhododactylus vinogradovi* Semenov, 1902: 269.

• *Phloeotribus rudis* Eichhoff, 1868b: 149.

= *Phloeotribus contractus* Chapuis, 1869: 46.

= *Phloeotribus puncticollis* Chapuis, 1869: 45.

= *Phthorophloeus striatus* Eggers, 1943d: 346.

= *Phloeotribus lineatus* Eggers, 1951: 148.

= *Phloeotribus peruensis* Schedl, 1952c: 69.

• *Phloeotribus rugulosus* Eggers, 1951: 147.

• *Phloeotribus scabratus* Blandford, 1897: 164.

• *Phloeotribus scabricollis* (Hopkins, 1916: 656) (*Phloeophthorus*).

= *Phloeotribus pseudoscabricollis* Atkinson, 1989b: 329.

• *Phloeotribus scarabaeoides* (Bernard, 1788: 271) (*Scolytus*).

= *Bostrichus oleae* Fabricius, 1792: 366.

= *Phloeotribus occidentalis* Sainte-Claire Deville, 1924: 148.

= *Phloeotribus oleiphilus* Del Guercio, 1925a: 196.

• *Phloeotribus schedli* Wood, 2007: 124.

• *Phloeotribus schoenbachi* Kirsch, 1866: 214.

• *Phloeotribus serratus* Eggers, 1943d: 354.

• *Phloeotribus setulosus* Eichhoff, 1868b: 149.

= *Phloeotribus dubius* Eichhoff, 1868b: 150.

= *Phloeotribus armatus* Blandford, 1897: 166.

= *Phloeotribus asperatus* Blandford, 1897: 166.

= *Phloeotribus sodalis* Blandford, 1897: 168.

= *Phloeotribus spinipennis* Eggers, 1930a: 168.

= *Phloeotribus bolivianus* Eggers, 1933f: 5.

= *Phloeotribus atlanticus* Schedl, 1951c: 81.

= *Phloeotribus mixtecus* Bright, 1972c: 1494.

• *Phloeotribus sharpi* (Guillebeau, 1893: 62) (*Phloeophthorus*).

• *Phloeotribus simplex* Wood, 1967b: 95.

• *Phloeotribus simplicidens* Wood, 1977b: 389.

• *Phloeotribus spinulosus* (Rey in: Eichhoff 1885: 127) (*Phthorophloeus*).

= *Hylesinus rhododactylus* Ratzeburg, 1837: 178.

= *Phloeophthorus chapuisii* Blandford, 1891: 213 (RN).

= *Elzearius crenatus* Guillebeau, 1893: 64.

= *Phloeophthorus perrisi* Guillebeau, 1893: 62.

• *Phloeotribus squamatus* Wood, 1969a: 7.

• *Phloeotribus squamiger* Wood, 1977b: 392.

• *Phloeotribus subcostatus* Eggers, 1943d: 346.

• *Phloeotribus subovatus* Blandford, 1897: 167.

= *Phloeotribus argentiniae* Blackman, 1943d: 386.

• *Phloeotribus sulcifrons* Chapuis, 1869: 45.

• *Phloeotribus suturalis* Eggers, 1943d: 349.

• *Phloeotribus tetricus* Wood, 1977b: 389.

• *Phloeotribus texanus* Schaeffer, 1908: 222.

• *Phloeotribus transversus* Chapuis, 1869: 44.

= *Phloeotribus marginatus* Eggers, 1933f: 4.

• *Phloeotribus truncatus* Wood, 2007: 129.

• *Phloeotribus uniseriatus* Eggers, 1943d: 350.

• *Phloeotribus venezuelensis* (Schedl, 1936a: 105) (*Phthorophloeus*).

• *Phloeotribus vesculus* Wood, 1977b: 392.

• *Phloeotribus vestitus* Eggers, 1943d: 349.

• *Phloeotribus vinogradowi* Semenov, 1902: 269.

• *Phloeotribus willei* Schedl, 1937a: 66.

= *Phloeotribus chiliensis* Eggers, 1942c: 16.

• *Phloeotribus woytkowskii* Wood, 2007: 126.

### PHRIXOSOMATINI Wood

1 genus, 26 spp.

#### *Phrixosoma* Blandford

26 spp.

• *Phrixosoma antillicum* Bright, 2019: 16.

• *Phrixosoma barinense* Wood, 2007: 69 (E).

• *Phrixosoma brasiliense* Schedl, 1959b: 546 (E).

• *Phrixosoma caraibicum* Schedl, 1966d: 101 (E).

• *Phrixosoma clusiae* Wood, 1969a: 1.

• *Phrixosoma concavifrons* Jordal, 2012: 53.

• *Phrixosoma costatum* (Schedl, 1941c: 383) (*Bothryperus costatus*).

• *Phrixosoma crebrum* Wood, 1971a: 2 (E).

• *Phrixosoma frustratum* Wood, 1971a: 3 (E).

• *Phrixosoma fuscovillosum* (Schedl, 1957b: 29) (*Bothryperus fuscovillosus*).

• *Phrixosoma garciniae* (Schedl, 1957b: 30) (*Bothryperus*).

• *Phrixosoma magnum* Blackman, 1943d: 392 (E).

• *Phrixosoma majus* (Eggers, 1940e: 229) (*Bothryperus major*).

• *Phrixosoma minus* Wood, 1956c: 248 (E).

• *Phrixosoma nigrum* (Eggers, 1933e: 21) (*Bothryperus niger*).

= *Hapalogenius niger* Schedl, 1952d: 7.

• *Phrixosoma obesum* Blackman, 1943d: 393 (E).

• *Phrixosoma parvum* Blackman, 1943d: 393 (E).

• *Phrixosoma peruvianum* Eggers, 1951: 148 (E).

• *Phrixosoma psaltes* (Hagedorn, 1909: 742) (*Bothryperus*).

• *Phrixosoma quadrioculatum* (Eggers, 1920b: 119) (*Neohylesinus quadrioculatus*).

• *Phrixosoma rubrum* Wood, 2007: 69 (E).

• *Phrixosoma rude* Blandford, 1897: 148.

• *Phrixosoma sinuosum* (Schedl, 1941c: 392) (*Bothryperus sinuosus*).

• *Phrixosoma striatum* (Eggers, 1929d: 41) (*Sphaerosinus striatus*).

• *Phrixosoma uniseriatum* (Eggers, 1940e: 230) (*Bothryperus uniseriatus*).

• *Phrixosoma viriosum* Wood, 1971a: 2 (E).

### POLYGRAPHINI Chapuis

9 genera, 155 spp. (1 sp. with 2 sspp.).

#### *Bothinodroctonus* Schedl

4 spp.

• *Bothinodroctonus bicinctus* Schedl, 1969a: 209.

• *Bothinodroctonus indicus* Wood, 1988b: 191.

• *Bothinodroctonus longicornis* Knížek & Mandelshtam, 2015: 289.

• *Bothinodroctonus setosus* Wood, 1988b: 192.

#### *Cardroctonus* Schedl

3 spp.

• *Cardroctonus africanus* (Nunberg, 1964: 433) (*Carphoborus*).

• *Cardroctonus congonus* Schedl, 1966f: 362.

• *Cardroctonus orientalis* Schedl, 1966f: 361.

#### *Carphobius* Blackman

4 spp.

• *Carphobius arizonicus* Blackman, 1943d: 398.

• *Carphobius caterinoi* Cognato & Smith, 2023: 259.

• *Carphobius cupressi* Wood, 1974a: 12.

• *Carphobius pilifer* Wood, 1983: 652.

#### *Carphoborus* Eichhoff

36 spp.

• *Carphoborus andersoni* Swaine, 1919: 6.

• *Carphoborus bicornis* Wood, 1986a: 269.

• *Carphoborus bifurcus* Eichhoff, 1868b: 147.

= *Carphoborus bicristatus* Chapuis, 1869: 41.

• *Carphoborus blaisdelli* Swaine, 1924c: 234.

= *Carphoborus cressatyi* Bruck, 1936: 36.

• *Carphoborus bonnairei* Brisout de Barneville, 1884: lii.

• *Carphoborus boswelliae* (Stebbing, 1903: 261) (*Cryphalus*).

• *Carphoborus brevisetosus* Wood, 1954a: 514.

• *Carphoborus carri* Swaine, 1917: 16.

• *Carphoborus cholodkovskyi* Spessivtsev, 1916: 64.

= *Carphoborus borealis* Karpiński, 1933: 28.

• *Carphoborus convexifrons* Wood, 1954a: 516.

• *Carphoborus costatus* Wichmann, 1915a: 106.

• *Carphoborus declivis* Wood, 1954a: 522.

• *Carphoborus dunni* Swaine, 1924c: 235.

• *Carphoborus frontalis* Wood, 1954a: 515.

• *Carphoborus henscheli* Reitter, 1887: 192.

• *Carphoborus intermedius* Wood, 1954a: 523.

• *Carphoborus jurinskii* Eggers, 1910b: 36.

• *Carphoborus lautus* Wood, 1988b: 192.

• *Carphoborus marani* Pfeffer, 1941a: 177.

• *Carphoborus mexicanus* Bright, 1972c: 1491.

• *Carphoborus minimus* (Fabricius, 1798: 158) (*Bostrichus*).

= *Hylesinus minimus* Fabricius, 1801: 395.

= *Hylesinus squamulatus* Redtenbacher, 1858: 369.

= *Carphoborus balgensis* Murayama, 1943: 99.

• *Carphoborus perplexus* Wood, 1960b: 59.

• *Carphoborus perrisi* (Chapuis, 1869: 31) (*Hylesinus*).

= *Carphoborus abachidsei* Stark, 1952: 232.

= *Carphoborus kushkensis* Sokanovskiy, 1954: 16.

• *Carphoborus piceae* Wood, 1974a: 11.

• *Carphoborus pini* Eichhoff, 1881: 131.

= *Carphoborus atritus* Peyerimhoff, 1932: 274.

• *Carphoborus pinicolens* Wood, 1954a: 512.

= *Carphoborus tuberculatus* Bright, 1964: 165.

• *Carphoborus ponderosae* Swaine, 1924c: 236.

• *Carphoborus pseudotsugae* Wood, 1954a: 522.

• *Carphoborus radiatae* Swaine, 1918: 57.

• *Carphoborus rossicus* Semenov, 1902: 272.

• *Carphoborus sansoni* Swaine, 1924c: 235.

= *Carphoborus engelmanni* Wood, 1951: 31.

• *Carphoborus simplex* LeConte, 1876: 383.

= *Carphoborus swainei* Bruck, 1933b: 105.

• *Carphoborus taireiensis* Murayama, 1943: 98.

• *Carphoborus teplouchovi* Spessivtsev, 1916: 65.

• *Carphoborus vandykei* Bruck, 1933b: 104.

• *Carphoborus zhobi* (Stebbing, 1909: 18) (*Phloeosinus*).

#### *Chortastus* Schaufuss

5 spp.

• *Chortastus camerunus* Schaufuss, 1905c: 15.

• *Chortastus minimus* Hagedorn, 1909: 738.

= *Chortastus medius* Eggers, 1924: 100.

= *Chortastus agnatus* Eggers, 1935e: 301.

= *Chortastus sulcatus* Eggers, 1935e: 301.

= *Chortastus brunneus* Nunberg, 1967: 317.

= *Afrochramesus baguenai* Schedl, 1971c: 197.

• *Chortastus orientalis* Schedl, 1958a: 868.

• *Chortastus schenklingi* Hagedorn, 1909: 737.

• *Chortastus verrucosus* Jordal, 2025b: 82.

#### *Dolurgocleptes* Schedl

2 spp.

• *Dolurgocleptes malgassicus* Schedl, 1965f: 62.

• *Dolurgocleptes punctifer* Jordal, 2009b: 44.

#### *Halystus* Schedl

2 spp.

• *Halystus bimaculatus* Knížek, 2012: 512.

• *Halystus namibiae* Schedl, 1982: 283.

= *Phloeographus mamibiae* Wood, 1984b: 229.

#### *Polygraphus* Erichson

97 spp. (1 sp. with 2 sspp.).

• *Polygraphus abietis* Kurentsov, 1941: 131.

• *Polygraphus aequalis* Schedl, 1961g: 129.

• *Polygraphus aequatus* Schedl, 1961g: 129.

• *Polygraphus afzeliae* Schedl, 1954a: 880.

• *Polygraphus amoenus* (Schaufuss, 1891: 10) (*Hylurgus*).

• *Polygraphus amplifolius* Yin & Huang, 1996: 347.

• *Polygraphus angusticollis* Schedl, 1965f: 52.

• *Polygraphus angustus* Tsai & Yin, 1965: 335.

• *Polygraphus anogeissi* Wood, 1988b: 194.

• *Polygraphus apicalis* Schedl, 1957b: 33.

• *Polygraphus aterrimus* Strohmeyer, 1908a: 69.

= *Polygraphus niger* Stebbing, 1914: 520.

• *Polygraphus basutoae* Schedl, 1957b: 32.

• *Polygraphus bicolor* Eggers, 1935e: 302.

= *Polygraphus latus* Eggers, 1935e: 303.

• *Polygraphus binotatus* Schedl, 1957b: 41.

• *Polygraphus brunneus* Eggers, 1920a: 236.

• *Polygraphus carphoboroides* Eggers, 1920a: 237.

• *Polygraphus confusus* Eggers, 1940d: 99.

• *Polygraphus congonus* Eggers, 1940d: 100.

• *Polygraphus convexifrons* Wood, 1951: 32.

• *Polygraphus coronatus* Eggers, 1920a: 237.

= *Polygraphus pygmaeus* Eggers, 1920a: 239.

= *Polygraphus granulifer* Eggers, 1935e: 303.

• *Polygraphus creber* Schedl, 1961g: 128.

• *Polygraphus difficilis* Wood, 1988b: 194.

• *Polygraphus dimorphus* Schedl, 1957b: 39.

• *Polygraphus formosanus* Nobuchi, 1967: 16.

• *Polygraphus fulvipennis* Niisima, 1941: 131.

• *Polygraphus gracilis* Niisima, 1909: 132.

• *Polygraphus grandiclava* Thomson, 1886b: lxxii.

= *Polygraphus cembrae* Seitner, 1911: 109.

• *Polygraphus grandis* Nunberg, 1967: 318.

• *Polygraphus granulatus* Eggers, 1932b: 29.

= *Polygraphus tanzanicus* Browne, 1970: 549.

• *Polygraphus granulicauda* Schedl, 1939c: 171.

• *Polygraphus granulifer* Eggers, 1935e: 303.

• *Polygraphus hoppingi* Swaine, 1925a: 51.

• *Polygraphus horyurensis* Murayama, 1937: 368.

• *Polygraphus japonicus* Nunberg, 1956c: 208.

= *Polygraphus granulatus* Niisima, 1941: 125.

= *Polygraphus uchimappensis* Murayama, 1956: 279 (RN).

• *Polygraphus jezoensis* Niisima, 1909: 135.

• *Polygraphus junnanicus* Sokanovskiy, 1959a: 93.

• *Polygraphus kaimochii* (Nobuchi, 1981b: 13) (*Nipponopolygraphus*).

= *Polygraphus querci* Wood, 1988b: 195.

• *Polygraphus kasukumbii* Schedl, 1957b: 40.

• *Polygraphus kisoensis* Niisima, 1941: 131.

• *Polygraphus kivuensis* Schedl, 1957b: 37.

• *Polygraphus knochei* Eggers, 1920a: 238.

• *Polygraphus longifolia* Stebbing, 1903: 255.

= *Polygraphus himalayensis* Stebbing, 1908: 8.

• *Polygraphus longipilis* Schedl, 1957b: 35.

• *Polygraphus major* Stebbing, 1903: 234.

• *Polygraphus majusculus* Schedl, 1957b: 34.

= *Polygraphus symphoniae* Browne, 1970: 549.

• *Polygraphus meakanensis* Niisima, 1935: 2.

• *Polygraphus militaris* (Eggers, 1920a: 235) (*Ozophagus*).

• *Polygraphus minutissimus* Schedl, 1972a: 286.

• *Polygraphus montanus* Schedl, 1957b: 42.

• *Polygraphus muluensis* Bright, 2000: 49.

• *Polygraphus musangae* (Schedl, 1957b: 28) (*Chortastus*).

• *Polygraphus natalensis* Eggers, 1920a: 238.

• *Polygraphus nigrielytris* Niisima, 1913: 2.

• *Polygraphus nobuchii* Choo & Woo, 1989: 57.

• *Polygraphus oblongus* Blandford, 1894c: 75.

• *Polygraphus occidentalis* Schedl, 1954a: 879.

= *Polygraphus occidentalis minusculus* Schedl, 1966b: 236.

• *Polygraphus orientalis* (Eggers, 1922c: 166) (*Ozophagus*).

• *Polygraphus parvulus* Murayama, 1956: 279.

• *Polygraphus perlaetus* Schedl, 1953c: 84.

• *Polygraphus pini* Stebbing, 1914: 522 (RN).

= *Polygraphus minor* Stebbing, 1903: 234.

• *Polygraphus poligraphus* (Linnaeus, 1758: 355) (*Dermestes*).

= *Bostrichus pubescens* Fabricius, 1792: 368.

= *Polygraphus griseus* Eggers, 1923a: 136.

• *Polygraphus potens* Schedl, 1965f: 53.

• *Polygraphus primus* Wichmann, 1915b: 217.

= *Ozophagus camerunus* Eggers, 1920a: 235.

= *Polygraphus opacicollis* Eggers, 1940d: 100.

• *Polygraphus proximus* Blandford, 1894c: 75.

= *Polygraphus miser* Blandford, 1894c: 76.

= *Polygraphus oblongus* Blandford, 1894c: 75.

= *Polygraphus laticollis* Eggers, 1926a: 135.

= *Polygraphus proximus nigricans* Kurentsov, 1948: 50.

= *Polygraphus ussuriensis* Schedl, 1955g: 11.

= *Polygraphus magnus* Murayama, 1956: 279.

• *Polygraphus pseudobrunneus* Schedl, 1971d: 7.

• *Polygraphus pterocaryi* Yin & Huang, 1996: 345.

• *Polygraphus punctifrons* Thomson, 1886a: xi.

= *Polygraphus seriatus* Reitter, 1913: 56.

= *Polygraphus krivolutzkianus* Stark, 1956: 830.

= *Polygraphus bicolor* Sokanovskiy, 1960: 675.

• *Polygraphus quadrioculatus* (Hagedorn, 1908: 373) (*Spongotarsus*).

• *Polygraphus ruandae* Schedl, 1957b: 34.

• *Polygraphus rudis hexiensis* Yin & Huang, 1996: 348.

• *Polygraphus rudis rudis* Eggers, 1933d: 99 (*Polygraphus rudis*).

= *Polygraphus rudis likiangensis* Tsai & Yin, 1965: 334.

= *Polygraphus rudis retriventriculus* Tsai & Yin, 1965: 328.

= *Polygraphus zhungdianensis* Tsai & Yin, 1965: 334.

= *Polygraphus squameus* Yin & Huang, 1981: 558.

• *Polygraphus rufipennis* (Kirby, 1837: 193) (*Apate*).

= *Apate brevicomis* Kirby, 1837: 194.

= *Apate nigriceps* Kirby, 1837: 194.

= *Polygraphus saginatus* Mannerheim, 1853: 237.

• *Polygraphus rufus* (Eggers, 1936e: 83) (*Spongotarsus*).

• *Polygraphus sachalinensis* Eggers, 1926a: 135.

= *Polygraphus sachalinensis fontinalis* Kurentsov, 1941: 136.

• *Polygraphus scalptor* Schedl, 1965f: 54.

• *Polygraphus setosus* Schedl, 1979c: 128.

• *Polygraphus shariensis* Niisima, 1941: 127.

• *Polygraphus sinensis* Eggers, 1933d: 100.

• *Polygraphus squamosus* (Schedl, 1975c: 384) (*Blastophagus*).

• *Polygraphus squamulatus* Niisima, 1941: 129.

• *Polygraphus ssiori* Niisima, 1909: 132.

• *Polygraphus subopacus* Thomson, 1871: 393.

= *Polygraphus subopacus minor* Lindemann, 1875b: 242.

= *Polygraphus subopacus xaverti* Reitter, 1911: 47.

= *Polygraphus nanus* Schedl, 1955g: 22.

• *Polygraphus subsulcatus* Schedl, 1957b: 38.

• *Polygraphus sulcatus* Schedl, 1952b: 10.

• *Polygraphus sumatranus* Schedl, 1961f: 72.

• *Polygraphus szemaoensis* Tsai & Yin, 1965: 335.

• *Polygraphus taiwanensis* Schedl, 1967b: 128.

• *Polygraphus tenuipennis* Schedl, 1957b: 43.

• *Polygraphus tenuis* Schedl, 1952b: 10.

• *Polygraphus thitsi* Wood, 1992: 86.

• *Polygraphus trenchi* Stebbing, 1905: 6.

• *Polygraphus tropicus* Eggers, 1932b: 29.

• *Polygraphus ugandae* Schedl, 1965e: 7.

• *Polygraphus verrucifrons* Tsai & Yin, 1965: 335.

• *Polygraphus vexator* (Browne, 1970: 551) (*Spongotarsus*).

• *Polygraphus vietnamensis* Schedl, 1965a: 340.

• *Polygraphus yunnanensis* Bright in: Bright and Skidmore 2002: 4 (RN).

= *Polygraphus querci* Yin & Huang, 1996: 346.

• *Polygraphus zambianus* Beaver in: Beaver and Löyttyniemi 1985: 66.

#### *Serrastus* Nunberg

2 spp.

• *Serrastus serrifer* (Hagedorn, 1909: 739) (*Chortastus*).

• *Serrastus similis* (Eggers, 1924: 100) (*Chortastus*).

= *Serrastus ivoriensis* Nunberg, 1969: 393.

### SCOLYTINI Latreille

6 genera, 214 spp. (2 spp. with 4 sspp.).

#### *Camptocerus* Dejean

32 spp.

• *Camptocerus aeneipennis* (Fabricius, 1801: 392) (*Hylesinus*).

= *Hylesinus gibbus* Fabricius, 1801: 392.

• *Camptocerus angustior* Eggers, 1928a: 91.

• *Camptocerus annectens* Wood, 2007: 210.

• *Camptocerus aterrimus* Eggers, 1933f: 12.

• *Camptocerus auricomus* Blandford, 1896d: 125.

= *Camptocerus striatulus* Hagedorn, 1905a: 547.

• *Camptocerus charpentierae* Schedl, 1970c: 582.

• *Camptocerus coccoformus* Smith & Cognato, 2010: 43.

• *Camptocerus costatus* Chapuis, 1869: 51.

= *Camptocerus seriatus* Eggers, 1933f: 12.

• *Camptocerus distinctus* Smith & Cognato, 2010: 68.

• *Camptocerus doleae* Smith & Cognato, 2010: 45.

• *Camptocerus hirtipennis* Schedl, 1973b: 165.

• *Camptocerus igniculus* Smith & Cognato, 2010: 47.

• *Camptocerus inoblitus* (Schedl, 1939h: 722) (*Loganius*).

= *Loganius morio* Schedl, 1952e: 348.

• *Camptocerus latipilis* Schedl, 1973b: 166.

• *Camptocerus lucwildi* Smith & Cognato, 2017: 445.

• *Camptocerus major* (Eggers, 1929e: 60) (*Loganius*).

• *Camptocerus mallopterus* Smith & Cognato, 2010: 50.

• *Camptocerus mandelshtami* Petrov, 2007: 101.

• *Camptocerus niger* (Fabricius, 1801: 393) (*Hylesinus*).

= *Camptocerus squamiger* Chapuis, 1869: 51.

= *Camptocerus tectus* Eggers, 1943a: 244.

• *Camptocerus noel* Smith & Cognato, 2010: 73.

• *Camptocerus occidentalis* Eggers, 1928a: 91.

• *Camptocerus opacicollis* (Eggers, 1929e: 61) (*Loganius*).

= *Camptocerus infidelis* Wood, 1969a: 11.

= *Camptocerus aquilus* Wood, 1972a: 244.

= *Camptocerus uniseriatus* Schedl, 1972f: 54.

• *Camptocerus orientalis* Eggers, 1943a: 244.

• *Camptocerus petrovi* Smith & Cognato, 2010: 52.

• *Camptocerus pilifrons* Smith & Cognato, 2010: 56.

• *Camptocerus pseudoangustior* Smith & Cognato, 2010: 76.

• *Camptocerus quadridens* Blackman, 1943d: 379.

• *Camptocerus rectus* Wood, 1972a: 245.

• *Camptocerus satyrus* Smith & Cognato, 2010: 53.

• *Camptocerus suturalis* (Fabricius, 1801: 393) (*Hylesinus*).

= *Hylesinus fasciatus* Fabricius, 1801: 392.

= *Camptocerus cinctus* Chapuis, 1869: 15.

• *Camptocerus unicornus* Smith & Cognato, 2010: 31.

• *Camptocerus zucca* Smith & Cognato, 2010: 60.

#### *Ceratolepis* Chapuis

7 spp.

• *Ceratolepis amazonica* (Eggers, 1929e: 60) (*Loganius amazonicus*).

= *Ceratolepsis amazonicus* Schedl, 1952e: 349.

• *Ceratolepis boliviae* (Blackman, 1943d: 378) (*Camptocerus*).

• *Ceratolepis hylurgoides* (Schedl, 1949b: 262) (*Coptodryas*).

• *Ceratolepis insignis* (Wood, 1969a: 9) (*Cnemonyx*).

• *Ceratolepis jucunda* Chapuis, 1869: 52.

• *Ceratolepis maculicornis* Blandford, 1896d: 127.

• *Ceratolepis nigra* Eggers, 1933f: 13 (*Ceratolepis niger*).

= *Camptocerus nigricans* Schedl, 1962a: 485 (RN).

#### *Cnemonyx* Eichhoff

23 spp.

• *Cnemonyx atratus* (Blandford, 1896d: 129) (*Loganius*).

= *Cnemonyx nitens* Wood, 1969a: 9.

• *Cnemonyx brevisetosus* Schedl, 1939f: 407.

• *Cnemonyx creber* Schedl, 1951c: 76.

• *Cnemonyx cupreus* Petrov, 2017: 328.

• *Cnemonyx difformis* (Schedl, 1951c: 74) (*Loganius*).

= *Loganius cirratus* Nunberg, 1958: 482.

• *Cnemonyx errans* (Blandford, 1896d: 127) (*Ceratolepis*).

= *Ceratolepis brasiliensis* Schedl, 1936a: 104.

= *Ceratolepis barbatus* Schedl, 1954b: 24.

= *Cnemonyx schedli* Wood, 1972a: 244 (RN).

• *Cnemonyx exiguus* (Blandford, 1896d: 130) (*Loganius*).

= *Loganius pumilus* Eggers, 1929e: 65.

• *Cnemonyx furvescens* Wood, 1979: 137.

• *Cnemonyx galeritus* Eichhoff, 1868b: 150.

= *Minulus barbatus* Eggers, 1912c: 207.

• *Cnemonyx glaber* (Eggers, 1929e: 64) (*Loganius*).

• *Cnemonyx glabratus* (Schedl, 1940a: 329) (*Loganius*).

• *Cnemonyx incanus* Petrov, 2017: 329.

• *Cnemonyx insidiosus* (Schedl, 1939e: 171) (*Loganius*).

• *Cnemonyx longicollis* (Blandford, 1896d: 128) (*Loganius*).

• *Cnemonyx minor* Schedl, 1951c: 75.

• *Cnemonyx minusculus* (Blandford, 1896d: 130) (*Loganius*).

= *Loganius vismiae* Eggers, 1929e: 63.

• *Cnemonyx panamensis* (Blandford, 1896d: 129) (*Loganius*).

• *Cnemonyx parvus* (Nunberg, 1972: 195) (*Loganius*).

• *Cnemonyx protivorus* Wood, 1979: 137.

• *Cnemonyx rugulosus* (Eggers, 1929e: 64) (*Loganius*).

• *Cnemonyx setulosus* (Eggers, 1929e: 62) (*Loganius*).

= *Loganius similis* Eggers, 1929e: 63.

• *Cnemonyx vestitus* (Eggers, 1929e: 59) (*Loganius*).

• *Cnemonyx vismiacolens* Wood, 1979: 138 (E).

#### *Loganius* Chapuis

18 spp.

• *Loganius acuminatus* (Schedl, 1976: 61) (*Cnemonyx*).

• *Loganius confinis* Wood, 1961c: 94.

• *Loganius equihuai* (Wood, 1983: 654) (*Cnemonyx*).

• *Loganius euphorbiae* (Wood, 1986a: 270) (*Cnemonyx*).

• *Loganius evidens* (Wood, 1983: 654) (*Cnemonyx*).

• *Loganius exilis* Wood, 1967c: 119.

• *Loganius fastigius* Wood, 1961c: 93.

• *Loganius ficus* Schwarz, 1894b: 44.

= *Ceratolepis nubilus* Blackman, 1943d: 380.

• *Loganius flavicornis* Chapuis, 1869: 52.

= *Loganius scaliger* Hagedorn, 1910: 5.

= *Cnemonyx vianai* Schedl, 1951a: 289.

= *Cnemonyx opacus* Wood, 1969a: 10.

• *Loganius gracilens* (Wood, 1969a: 10) (*Cnemonyx*).

• *Loganius impressus* Wood, 1961c: 90.

• *Loganius liratus* Wood, 1961c: 92.

• *Loganius niger* Wood, 1961c: 95.

= *Cnemonyx nigrellus* Wood, 1974d: 278 (RN).

• *Loganius prociduus* Wood, 1961c: 91.

• *Loganius recavus* (Wood, 1969a: 11) (*Cnemonyx*).

• *Loganius splendens* Wood, 1961c: 88.

• *Loganius squamifer* (Wood, 1979: 138) (*Cnemonyx*).

• *Loganius vagabundus* Wood, 1961c: 89.

#### *Scolytopsis* Blandford

7 spp.

• *Scolytopsis brasiliensis* Eggers, 1931a: 16.

• *Scolytopsis laticollis* Wood, 1968a: 14.

• *Scolytopsis orinocanus* Wood, 1971a: 18.

• *Scolytopsis peruanus* Eggers, 1937b: 83.

• *Scolytopsis puncticollis* Blandford, 1896d: 123.

= *Scolytopsis cubensis* Wood, 1961c: 87.

• *Scolytopsis sokolovi* Petrov, 2020a: 430.

• *Scolytopsis toba* Wichmann, 1914a: 136.

= *Scolytopsis argentinensis* Eggers, 1937b: 84.

= *Scolytopsis bruchi* Schedl, 1939e: 170.

#### *Scolytus* Geoffroy

127 spp. (2 spp. with 4 sspp.).

• *Scolytus abaensis* Tsai & Yin in: Tsai et al. 1962: 10.

• *Scolytus amazonicus* Schedl, 1972f: 52.

• *Scolytus amygdali* Guérin-Méneville, 1847: xlvii.

= *Scolytus amygdali rufipennis* Brancsik, 1874: 135.

= *Eccoptogaster anatolicus* Eggers, 1911a: 74.

= *Scolytus mailleri* Eggers, 1912b: 49.

= *Scolytus aegyptiacus* Pic, 1920: 55.

• *Scolytus angustatus* Browne, 1970: 575.

= *Scolytus facialis* Schedl, 1973b: 164.

• *Scolytus antennatus* Schedl, 1935g: 272.

• *Scolytus aomoriensis* Nobuchi, 1973: 16.

• *Scolytus aratus* Blandford, 1894c: 79.

= *Scolytus aequipunctatus* Niisima, 1905: 71.

= *Scolytus brevipennis* Kurentsov, 1941: 100.

= *Scolytus intermedius* Kurentsov, 1941: 82.

• *Scolytus aztecus* Wood, 1967c: 120.

• *Scolytus barbatus* Schedl, 1976: 59.

• *Scolytus barinensis* Wood, 1971a: 18.

• *Scolytus betulae* Niisima, 1943: 141.

• *Scolytus bicinctus* Schedl, 1972f: 52.

• *Scolytus bicolor* Eggers, 1931a: 15.

• *Scolytus binodus* Wood, 1982: 230.

• *Scolytus bispinatus* Wood, 2007: 225.

• *Scolytus bolivianus* Schedl, 1966d: 97.

• *Scolytus butovitschi* Stark, 1936: 153.

= *Scolytus butovitschi* Eggers, 1942a: 35.

• *Scolytus canellae* Wood, 2007: 230.

• *Scolytus carinatus* Chapuis, 1869: 55.

= *Scolytus atratus* Chapuis, 1869: 56.

• *Scolytus carpini* (Ratzeburg, 1837: 187) (*Eccoptogaster*).

= *Eccoptogaster peregrinus* Eggers, 1908a: 215.

= *Eccoptogaster balcanicus* Eggers, 1911a: 75.

• *Scolytus carveli* Petrov & Mandelshtam, 2010: 76.

• *Scolytus caudatus* Eggers, 1931b: 35.

= *Scolytus pseudocaudatus* Eggers, 1931b: 37.

= *Scolytus conidens* Browne, 1970: 574.

• *Scolytus chelogaster* Schedl, 1958d: 168.

• *Scolytus chikisanii* Niisima, 1905: 69.

= *Scolytus curviventralis* Niisima, 1905: 70.

= *Scolytus mandschuricus* Schedl, 1941a: 42.

• *Scolytus claviger* Blandford, 1894c: 80.

= *Eccoptogaster platystylus* Wichmann, 1915b: 213.

• *Scolytus confusus* (Eggers, 1922a: 13) (*Eccoptogaster*).

• *Scolytus convexus* Schedl, 1972f: 53.

• *Scolytus costellatus* Chapuis, 1869: 58.

= *Scolytus pseudocostellatus* Schedl, 1937b: 159.

= *Scolytus strigipennis* Schedl, 1976: 60.

• *Scolytus cristatus* Wood, 1969a: 12.

• *Scolytus dahuricus* Chapuis, 1869: 60.

= *Scolytus agnatus* Blandford, 1894c: 78.

= *Scolytus possyeti* Stark, 1938: 129.

• *Scolytus dentatus* Bright, 1964: 167.

• *Scolytus dimidiatus* Chapuis, 1869: 57.

• *Scolytus ecksteini* Butovitsch, 1929: 26.

• *Scolytus eichhoffi* Reitter, 1895: 40.

= *Scolytus iranicus* Eggers, 1941d: 123.

• *Scolytus ellipticus* Murayama, 1958: 929.

• *Scolytus elongatus* Schedl, 1972f: 54.

• *Scolytus emarginatus* (Wichmann, 1915b: 215) (*Eccoptogaster*).

= *Scolytus seulensis* Murayama, 1930: 5.

• *Scolytus ensifer* Eichhoff, 1881: 163.

• *Scolytus esuriens* Blandford, 1894c: 77.

= *Scolytus sachalinensis* Michalski, 1964: 666.

• *Scolytus excavatus* Wood, 2007: 229.

• *Scolytus fagi* (Walsh, 1867: 58) (*Eccoptogaster*).

• *Scolytus fiskei* Blackman, 1934: 25.

• *Scolytus frontalis* Blandford, 1894c: 79.

= *Scolytus formosanus* Eggers, 1939a: 115.

• *Scolytus golbachi* Schedl, 1951a: 288.

• *Scolytus gretschkini* Sokanovskiy, 1956: 43.

• *Scolytus hermosus* Wood, 1968a: 12.

• *Scolytus incognitus* Eggers, 1951: 154.

• *Scolytus intricatus* (Ratzeburg, 1837: 186) (*Eccoptogaster*).

= *Scolytus picicolor* Stephens, 1830: 362.

= *Scolytus penicillatus* Reitter, 1913: 21.

= *Eccoptogaster simmeli* Eggers, 1923a: 133.

= *Scolytus lenkoranus* Eggers, 1942a: 34.

• *Scolytus jacobsoni* (Spessivtsev, 1919: 246) (*Eccoptogaster*).

= *Scolytus jacobsoni montana* Kurentsov, 1935: 22.

= *Scolytus rimskii* Kurentsov, 1941: 103.

= *Scolytus nunbergi* Michalski, 1964: 665.

• *Scolytus japonicus* Chapuis in: Chapuis and Eichhoff 1876: 199.

= *Eccoptogaster mandli* Eggers, 1922a: 13.

= *Scolytus starki* Kurentsov, 1941: 101.

= *Scolytus subconfusus* Eggers, 1941d: 123.

= *Scolytus ussuriensis* Kurentsov, 1941: 102.

• *Scolytus jaroschewskii jaroschewskii* Schevyrew, 1893b: 31 (*Scolytus jaroschewskii*) (RN).

= *Scolytus unispinosus* Schevyrew, 1890: 470.

= *Scolytus jaroschewskii* Schevyrew, 1893a: 90 (RN).

= *Scolytus granulifer* Reitter, 1913: 98.

= *Eccoptogaster tauricus* Eggers, 1914b: 185.

• *Scolytus jaroschewskii kostini* Sokanovskiy, 1954: 15 (*Scolytus kostini*).

• *Scolytus jiulianshanensis* Zhang, Li & Smith in: Zhang et al. 2021: 296.

• *Scolytus kashmirensis* Schedl, 1958d: 168.

• *Scolytus kirschii fasciatus* Reitter, 1890: 395 (*Scolytus fasciatus*).

= *Eccoptogaster demaisoni* Eggers, 1912b: 47.

• *Scolytus kirschii kirschii* Skalitzky, 1876: 110 (*Scolytus kirschii*).

= *Scolytus kirschii ruguloides* Sokanovskiy, 1954: 14.

• *Scolytus koenigi* Schevyrew, 1890: 471.

= *Scolytus aceris* Knotek, 1892: 235.

= *Scolytus siculus* Eggers, 1908c: 193.

= *Scolytus belokani* Stark, 1941: 302.

• *Scolytus koltzei* Reitter, 1894b: 128.

= *Scolytus vexator* Reitter, 1913: 23.

= *Scolytus yablonianus* Murayama, 1943: 96.

= *Scolytus shanhaiensis* Yin & Huang, 1980: 50.

• *Scolytus kononovi* Kurentsov, 1941: 98.

• *Scolytus laetus* Wood, 1975a: 25.

• *Scolytus laevis* Chapuis, 1869: 54.

= *Eccoptogaster loevendali* Eggers, 1912c: 203.

= *Scolytus pomacearum* Butovitsch, 1929: 44.

= *Scolytus azerbaidzhanicus* Michalski, 1964: 662.

• *Scolytus laricis* Blackman, 1934: 24.

• *Scolytus lindemani* Petrov & Mandelshtam, 2010: 81.

• *Scolytus major* Stebbing, 1903: 203.

= *Scolytus deodara* Stebbing, 1903: 220.

= *Scolytus minor* Stebbing, 1903: 207.

• *Scolytus mali* (Bechstein, 1805: 882) (*Bostrichus*).

= *Bostrichus castaneus* Illiger, 1808: 304.

= *Eccoptogaster castaneus* Ratzeburg, 1837: 187.

= *Eccoptogaster pruni* Ratzeburg, 1837: 187.

= *Eccoptogaster pyri* Ratzeburg, 1837: 186.

= *Scolytus sulcatus* LeConte, 1868: 167.

= *Scolytus nitidulus* Chapuis, 1869: 59.

= *Scolytus pruni strigilatus* Reitter, 1908b: 23.

= *Scolytus bicallosus* Eggers, 1933c: 75.

• *Scolytus marginatus* Chapuis, 1869: 56.

= *Scolytus productus* Hagedorn, 1905a: 547.

• *Scolytus monticolae* (Swaine, 1917: 32) (*Eccoptogaster*).

• *Scolytus morawitzi* Semenov, 1902: 267.

= *Scolytus pini* Eggers, 1942a: 33.

• *Scolytus mozolevskae* Petrov & Mandelshtam, 2010: 85.

• *Scolytus multistriatus* (Marsham, 1802: 54) (*Ips*).

= *Scolytus flavicornis* Chevrolat in: Guérin-Méneville, 1835: pl. 40.

= *Scolytus ulmi* Redtenbacher, 1849: 361.

= *Scolytus javanus* Chapuis, 1869: 56.

= *Scolytus multistriatus triornatus* Eichhoff, 1881: 160.

= *Eccoptogaster abhorrens* Wichmann, 1913d: 210.

= *Scolytus nodifer* Reitter, 1913: 24.

= *Scolytus papuanus* Schedl, 1936b: 8.

= *Scolytus multistriatus therondi* Hoffmann, 1939: 36.

= *Scolytus kozikowskii* Michalski, 1964: 663.

• *Scolytus mundus* Wood, 1968a: 13.

• *Scolytus muticus* Say, 1824: 323.

• *Scolytus nakanei* Nobuchi, 1967: 16.

• *Scolytus neofacialis* Schedl, 1976: 60.

• *Scolytus nevermanni* Schedl, 1935g: 272.

• *Scolytus nitidus* Schedl, 1936b: 8.

• *Scolytus nodatus* Wood, 1969a: 12.

• *Scolytus nodulus* (Wichmann, 1915a: 102) (*Eccoptogaster*).

= *Eccoptogaster nodicornis* Wichmann, 1915b: 216.

• *Scolytus novateutonicus* Schedl, 1937b: 162.

• *Scolytus numidicus* Brisout de Barneville, 1883: 147.

• *Scolytus obelus* Wood, 1962: 81.

• *Scolytus obscuriceps* Wood, 2007: 235.

• *Scolytus oregoni* Blackman, 1934: 18.

• *Scolytus orientalis* Eggers, 1910a: 557 (*Eccoptogaster*).

= *Eccoptogaster affinis* Eggers, 1914a: 108.

• *Scolytus parviclaviger* Yin & Huang, 1980: 49.

• *Scolytus peruensis* Schedl, 1937b: 160.

• *Scolytus piceae* (Swaine, 1910a: 34) (*Eccoptogaster*).

• *Scolytus pilosus* Yin & Huang, 1980: 49.

• *Scolytus pinnatus* Eggers, 1928a: 94.

• *Scolytus pomi* Yin & Huang, 1980: 50.

• *Scolytus praeceps* LeConte, 1876: 373.

= *Scolytus abietis* Blackman, 1934: 21.

= *Scolytus opacus* Blackman, 1934: 20.

• *Scolytus propinquus* Blandford, 1896d: 121.

= *Scolytus penicillus* Schedl, 1973b: 165.

• *Scolytus proximus* Chapuis, 1869: 57.

= *Eccoptogaster brevicauda* Wichmann, 1915a: 104.

• *Scolytus pubescens* Stark, 1936: 153.

= *Scolytus pubescens* Eggers, 1942a: 34.

• *Scolytus pygmaeus* (Fabricius, 1787: 37) (*Bostrichus*).

= *Scolytus armatus* Comolli, 1837: 37.

= *Eccoptogaster noxius* Ratzeburg, 1837: 187.

• *Scolytus quadrispinosus* Say, 1824: 323.

= *Scolytus carya* Riley, 1867: 68.

= *Scolytus caryae* Walsh, 1867: 58.

• *Scolytus querci* Yin & Huang, 1980: 50.

• *Scolytus rabaglii* Smith & Cognato, 2013: 553.

• *Scolytus ratzeburgi* Janson, 1856: 87.

= *Eccoptogaster amurensis* Eggers, 1908d: 144.

= *Eccoptogaster sahlbergi* Eggers, 1912c: 204.

= *Eccoptogaster sibiricus* Eggers, 1922a: 14.

= *Scolytus lineatus* Kurentsov, 1941: 104.

= *Scolytus bituberculatus* Puzyr, 1951: 46.

• *Scolytus reflexus* Blackman, 1934: 13.

= *Scolytus wickhami* Blackman, 1934: 13.

= *Scolytus virgatus* Bright, 1972c: 1490.

• *Scolytus robustus* Blackman, 1934: 19.

• *Scolytus rugulosus* (Müller, 1818: 247) (*Bostrichus*).

= *Scolytus haemorrhous* Schmidberger, 1837: 27.

= *Eccoptogaster punctatus* Ratzeburg, 1837: 187.

= *Eccoptogaster assimilis* Boheman, 1858: 88.

= *Scolytus rugulosus fauveli* Reitter, 1895: 43.

= *Eccoptogaster mediterraneus* Eggers, 1922b: 121.

= *Scolytus rugulosus caucasicus* Butovitsch, 1929: 54.

= *Scolytus rugulosus samarkandicus* Butovitsch, 1929: 56.

= *Scolytus rugulosus similis* Butovitsch, 1929: 52.

= *Scolytus rugulosus sanctaluciae* Hoffmann, 1935: 82.

= *Scolytus manglissiensis* Lezhava, 1940: 71.

= *Scolytus taxicola* Lezhava, 1943: 193.

= *Scolytus rugulosus baluchistani* Schedl, 1958d: 165.

= *Scolytus rugulosus intermedius* Sokanovskiy, 1960: 674.

• *Scolytus schevyrewi* Semenov, 1902: 265.

= *Scolytus sinensis* Eggers, 1910b: 35.

= *Eccoptogaster frankei* Wichmann, 1915b: 214.

• *Scolytus scolytus* (Fabricius, 1775: 59) (*Bostrichus*).

= *Scolytus niger* Geoffroy, 1762: 139.

= *Scolytus punctatus* Müller, 1776: 57.

= *Dermestes scolythus* Sulzer, 1776: 21.

= *Scolytus destructor* Olivier, 1800a: 5.

= *Scolytus bostrichus* Tigny, 1801: 79.

= *Ips inermis* Marsham, 1802: 54.

= *Scolytus californicus* LeConte, 1868: 166.

= *Scolytus destructor ciliaris* Rey, 1892: 30.

= *Scolytus fuchsi* Reitter, 1913: 15.

= *Scolytus variabilis* Sokanovskiy, 1958: 37.

• *Scolytus semenovi* (Spessivtsev, 1919: 247) (*Eccoptogaster*).

• *Scolytus silvaticus* Bright, 1972c: 1489.

• *Scolytus sinopiceus* Tsai in: Tsai et al. 1962: 9.

• *Scolytus spinidens* Schedl, 1966d: 96.

• *Scolytus squamosus* Yin & Huang, 1980: 49.

• *Scolytus stepheni* Mandelshtam & Petrov, 2010b: 172.

• *Scolytus submarginatus* Schedl, 1937b: 163.

= *Scolytus submarginatus artestriatus* Schedl, 1937b: 163.

• *Scolytus subscaber* LeConte, 1876: 373.

• *Scolytus sulcifrons* Rey, 1892: 30 (*Scolytus destructor sulcifrons*).

= *Eccoptogaster leonii* Eggers, 1908c: 194.

• *Scolytus tadzhikistanicus* Stark, 1941: 302.

• *Scolytus thoracicus* Chapuis, 1869: 55.

= *Scolytus plaumanni* Wood, 2007: 229.

• *Scolytus torulus* Wood, 1975a: 25.

• *Scolytus transcaspicus* (Eggers, 1922b: 116) (*Eccoptogaster*).

• *Scolytus transversalis* Eggers, 1943d: 375.

• *Scolytus triarmatus* (Eggers, 1912c: 205) (*Eccoptogaster*).

• *Scolytus trispinosus* Strohmeyer, 1908a: 69.

= *Scolytus ventrosus* Schevyrew, 1890: 470.

= *Scolytus grandis* Kurentsov, 1941: 104.

• *Scolytus tsugae* (Swaine, 1917: 32) (*Eccoptogaster*).

• *Scolytus unicornis* Cao, Petrov & Wang in: Cao et al. 2023: 186.

• *Scolytus unispinosus* LeConte, 1876: 372.

= *Scolytus sobrinus* Blackman, 1934: 23.

• *Scolytus vagabundus* Petrov & Mandelshtam, 2010: 94.

• *Scolytus varshalovitchi* Michalski, 1973: 55.

• *Scolytus venezuelensis* Wood, 2007: 231.

• *Scolytus ventralis* LeConte, 1868: 167.

• *Scolytus woodi* Petrov & Mandelshtam, 2010: 95.

• *Scolytus zaitzevi* Butovitsch, 1929: 31.

### SCOLYTOPLATYPODINI Blandford

2 genera, 61 spp.

#### *Remansus* Jordal

4 spp.

• *Remansus mutabilis* (Schedl, 1965f: 78) (*Scolytoplatypus*).

• *Remansus pygmaeus* Jordal, 2013a: 24.

• *Remansus sahondrae* Jordal, 2013a: 22.

• *Remansus serratus* Jordal, 2013a: 26.

#### *Scolytoplatypus* Schaufuss

57 spp.

• *Scolytoplatypus africanus* Eggers, 1920b: 122.

• *Scolytoplatypus armatus* Eggers, 1924: 109.

• *Scolytoplatypus blandfordi* Gebhardt in: Beaver and Gebhardt 2006: 162.

• *Scolytoplatypus bombycinus* Browne, 1955: 361.

• *Scolytoplatypus brahma* Blandford, 1898b: 425.

= *Scolytoplatypus hamatus* Hagedorn, 1904c: 260.

= *Scolytoplatypus paucegranulatus* Eggers, 1935a: 242.

= *Scolytoplatypus hirsutus* Blackman, 1943b: 124.

• *Scolytoplatypus calvus* Beaver & Liu, 2007: 227.

• *Scolytoplatypus carinatus* Bright, 1994: 269.

• *Scolytoplatypus cirratus* Gebhardt in: Beaver and Gebhardt 2006: 165.

• *Scolytoplatypus congonus* Schedl, 1952b: 21.

• *Scolytoplatypus costatus* Gebhardt & Beaver in: Gebhardt et al. 2021: 486.

• *Scolytoplatypus curviciliosus* Gebhardt in: Beaver and Gebhardt 2006: 165.

• *Scolytoplatypus daimio* Blandford, 1893b: 433.

= *Scolytoplatypus siomio* Blandford, 1893b: 436.

= *Scolytoplatypus muticus* Hagedorn, 1904b: 124.

• *Scolytoplatypus darjeelingi* Stebbing, 1914: 607.

• *Scolytoplatypus denticauda* Buhroo & Zubair, 2024: 59.

• *Scolytoplatypus eichelbaumi* Hagedorn, 1905c: 64.

= *Scolytoplatypus acuminatus* Schedl, 1957b: 116.

= *Scolytoplatypus octospinosus* Nunberg, 1960: 295.

• *Scolytoplatypus eutomoides* Blandford, 1896e: 196.

= *Scolytoplatypus papuanus* Eggers, 1923b: 165.

= *Scolytoplatypus setosus* Schedl, 1942c: 192.

• *Scolytoplatypus exiguus* Beaver in: Beaver and Gebhardt 2006: 166.

• *Scolytoplatypus fasciatus* Hagedorn, 1904e: 405.

= *Scolytoplatypus maculatus* Nunberg, 1960: 299.

= *Scolytoplatypus strohmeyeri* Eggers, 1920c: 123

• *Scolytoplatypus gardneri* Maiti & Saha, 2009: 87.

• *Scolytoplatypus geminus* Gebhardt & Beaver in: Gebhardt et al. 2021: 489.

• *Scolytoplatypus glaber* Eggers, 1935a: 240.

• *Scolytoplatypus hova* Schaufuss, 1905b: 12.

• *Scolytoplatypus javanus* Eggers, 1923b: 164.

= *Scolytoplatypus piceus* Blackman, 1943b: 122.

• *Scolytoplatypus kivuensis* Schedl, 1957b: 117.

• *Scolytoplatypus kunala* Strohmeyer, 1908c: 161.

• *Scolytoplatypus lopchuensis* Maiti & Saha, 2009: 90.

• *Scolytoplatypus luzonicus* Eggers, 1935a: 244.

= *Scolytoplatypus benguetus* Blackman, 1943b: 123.

• *Scolytoplatypus macgregori* Blackman, 1943b: 121.

• *Scolytoplatypus mikado* Blandford, 1893b: 437.

• *Scolytoplatypus minimus* Hagedorn, 1904b: 125.

• *Scolytoplatypus nanus* Schedl, 1931b: 118.

• *Scolytoplatypus neglectus* Schedl, 1975d: 235.

• *Scolytoplatypus nitidicollis* Eggers, 1935a: 241.

• *Scolytoplatypus nitidus* Eggers, 1923b: 166.

• *Scolytoplatypus obtectus* Schedl, 1975d: 226.

• *Scolytoplatypus occidentalis* Browne, 1971: 118.

• *Scolytoplatypus opacicollis* Eggers, 1936c: 38.

• *Scolytoplatypus parvus* Sampson, 1921: 36.

• *Scolytoplatypus peniculatus* Gebhardt & Beaver in: Gebhardt et al. 2021: 490.

• *Scolytoplatypus permirus* Schaufuss, 1891: 31.

= *Scolytoplatypus madagascarensis* Schedl, 1953c: 71.

• *Scolytoplatypus pubescens* Hagedorn, 1904b: 123.

= *Scolytoplatypus pubescens kabakovi* Aksent’ev, 1992: 192.

• *Scolytoplatypus pusillus* Eggers, 1935a: 243.

• *Scolytoplatypus raja* Blandford, 1893b: 440.

= *Scolytoplatypus himalayensis* Stebbing, 1914: 604.

• *Scolytoplatypus reticulatus* Bright, 1994: 270.

• *Scolytoplatypus ruficauda* Eggers, 1939b: 9.

• *Scolytoplatypus rugosus* Jordal, 2013a: 17.

• *Scolytoplatypus samsinghensis* Maiti & Saha, 2009: 101.

• *Scolytoplatypus shogun* Blandford, 1894c: 126.

• *Scolytoplatypus sinensis* Tsai & Huang, 1965: 121.

• *Scolytoplatypus skyliuae* Liao, Lai & Beaver in: Liao et al. 2022: 33.

• *Scolytoplatypus superciliosus* Tsai & Huang, 1965: 121.

• *Scolytoplatypus truncatus* Browne, 1971: 120.

• *Scolytoplatypus tycon* Blandford, 1893b: 432.

= *Scolytoplatypus ussuriensis* Berger & Kholodkovskiy, 1916: 4.

• *Scolytoplatypus unipilus* Jordal, 2018a: 126.

• *Scolytoplatypus uter* Schedl, 1938a: 457.

• *Scolytoplatypus wugongshanensis* Liao, Lai & Beaver in: Liao et al. 2022: 30.

• *Scolytoplatypus zahradniki* Knížek, 2008: 120.

### TRYPOPHLOEINI Nüsslin

10 genera, 268 spp. (1 sp. with 2 sspp.).

#### *Afrocosmoderes* Johnson & Jordal

7 spp.

• *Afrocosmoderes caplandicus* (Schedl, 1965d: 115) (*Hypocryphalus*).

• *Afrocosmoderes grobleri* (Schedl, 1961a: 349) (*Miocryphalus*).

• *Afrocosmoderes madagascariensis* (Schedl, 1961g: 133) (*Ptilopodius*).

• *Afrocosmoderes niger* (Schedl, 1961g: 131) (*Erischidias*).

• *Afrocosmoderes pellitus* (Schedl, 1953c: 79) (*Erischidias*).

• *Afrocosmoderes pennatus* (Schedl, 1953c: 79) (*Miocryphalus*).

• *Afrocosmoderes schedli* Johnson in: Johnson et al. 2020a: 27 (RN).

= *Euptilius madagascariensis* Schedl, 1963d: 65.

#### *Atomothenemus* Bright

1 sp.

• *Atomothenemus unicus* Bright, 2019: 107.

#### *Cosmoderes* Eichhoff

15 spp.

• *Cosmoderes anticus* (Schedl, 1975a: 343) (*Erioschidias*).

• *Cosmoderes attenuatus* (Nobuchi, 1981b: 17) (*Pseudocosmoderes*).

• *Cosmoderes consobrinus* Blandford, 1894c: 86.

• *Cosmoderes corpulentus* (Schedl, 1971f: 151) (*Erioschidias*).

• *Cosmoderes cylindricus* (Schedl, 1962d: 103) (*Erioschidias*).

• *Cosmoderes elegans* Schedl, 1975a: 342.

• *Cosmoderes elongatus* (Eggers, 1939b: 4) (*Erioschidias*).

• *Cosmoderes euonymi* (Kurentsov, 1941: 159) (*Allernoporus*).

• *Cosmoderes frontalis* (Wood, 1960a: 21) (*Erischidias*).

• *Cosmoderes monilicollis* Eichhoff, 1878a: 387.

= *Cosmoderes monilicollis* Eichhoff, 1878b: 496.

= *Dendriops granulicollis* Schedl, 1953d: 125.

= *Erioschidias coriaceus* Schedl, 1959i: 474.

• *Cosmoderes papuanus* Schedl, 1975a: 341.

• *Cosmoderes queenslandi* (Schedl, 1938g: 43) (*Erioschidias*).

• *Cosmoderes ruandae* (Schedl, 1962d: 104) (*Erioschidias*).

• *Cosmoderes setistriatus* (Lea, 1910: 141) (*Cryphalus*).

• *Cosmoderes solitarius* (Schedl, 1957b: 46) (*Erioschidias*).

#### *Hypothenemus* Westwood

219 spp.

• *Hypothenemus aberrans* Browne, 1973: 287.

• *Hypothenemus abhorrens* Wood, 2007: 504.

• *Hypothenemus abruptus* (Schedl, 1961g: 135) (*Stephanoderes*).

• *Hypothenemus acaciae* (Eggers, 1920b: 120) (*Cryphalus*).

• *Hypothenemus adscitus* (Schedl, 1950e: 46) (*Stephanoderes*).

• *Hypothenemus adustus* Bright, 2019: 122.

• *Hypothenemus aethiops* (Schedl, 1965b: 26) (*Lepiceroides*).

• *Hypothenemus africanus* (Hopkins, 1915d: 30) (*Stephanoderes*).

= *Hypothenemus concavifrons* Bright & Torres, 2006: 405.

• *Hypothenemus agnatus* (Eggers, 1924: 103) (*Stephanoderes*).

= *Stephanoderes tungamwansolus* Schedl, 1939i: 385.

= *Stephanoderes confusus* Eggers, 1940e: 235.

• *Hypothenemus alternatus* (Eggers, 1943c: 73) (*Stephanoderes*).

• *Hypothenemus amakusanus* (Murayama, 1934a: 287) (*Stephanoderes*).

• *Hypothenemus amplissimus* Bright & Torres, 2006: 404.

• *Hypothenemus apicalis* Wood, 1974a: 19.

• *Hypothenemus areccae* (Hornung, 1842: 117) (*Bostrichus*).

= *Stephanoderes obscurus* Eichhoff, 1872a: 133.

= *Stephanoderes depressus* Eichhoff, 1878b: 155 (RN).

= *Hypothenemus vafer* Blandford, 1898a: 214.

= *Stephanoderes fungicola* Eggers, 1908a: 216.

= *Stephanoderes polyphagus* Eggers, 1924: 104.

= *Stephanoderes hispidus* Eggers, 1925: 156.

= *Hypothenemus heterolepsis* Costa Lima, 1928: 117.

= *Hypothenemus capitalis* Beeson, 1935a: 102.

= *Stephanoderes bambesanus* Eggers, 1940e: 232.

= *Hypothenemus eupolyphagus* Beeson, 1940: 193.

= *Stephanoderes subvestitus* Eggers, 1940e: 232.

= *Stephanoderes martiniquensis* Eggers, 1941a: 99.

= *Hypothenemus oahuensis* Schedl, 1941b: 110.

= *Hypothenemus subglabratus* Schedl, 1942c: 174.

= *Hypothenemus bauhaniae* Schedl, 1950b: 19.

= *Stephanoderes occidentalis* Schedl, 1954d: 76.

• *Hypothenemus artocarpi* Browne in: Beaver and Browne 1979: 588.

• *Hypothenemus arundinis* (Eichhoff, 1878a: 386) (*Stephanoderes*).

= *Stephanoderes arundinis* Eichhoff, 1878b: 157.

• *Hypothenemus ascitus* Wood, 1971a: 35.

• *Hypothenemus ater* (Eggers, 1932b: 31) (*Stephanoderes*).

• *Hypothenemus aterrimulus* Wood, 1989: 178 (RN).

= *Lepiceroides aterrimus* Schedl, 1957b: 59.

• *Hypothenemus aterrimus* (Schedl, 1951c: 104) (*Stephanoderes*).

• *Hypothenemus atomus* Hopkins, 1915d: 15.

= *Stephanoderes buscki* Hopkins, 1915d: 30.

= *Hypothenemus impressifrons* Hopkins, 1915d: 15.

= *Hypothenemus marylandicae* Hopkins, 1915d: 15.

= *Hypothenemus robiniae* Hopkins, 1915d: 15.

= *Hypothenemus toxicodendri* Hopkins, 1915d: 15.

= *Ernoporus nigrinus* Schedl, 1967d: 7.

• *Hypothenemus atratus* (Schedl, 1964h: 45) (*Stephanoderes*).

• *Hypothenemus aulmanni* (Hagedorn, 1912b: 41) (*Cryphalus*).

= *Hypothenemus tonsus* Eggers, 1920a: 242.

• *Hypothenemus balachowskyi* Menier, 1971: 141.

• *Hypothenemus baloghi* (Schedl, 1967c: 226) (*Stephanoderes*).

• *Hypothenemus barinensis* Wood, 2007: 505.

• *Hypothenemus bauhaniae* (Schedl, 1950b: 20) (*Stylolentus*).

• *Hypothenemus bezaziani* Peyerimhoff, 1935: 192.

• *Hypothenemus bicinctus* Schedl, 1959i: 479.

• *Hypothenemus bidens* Browne, 1973: 288.

• *Hypothenemus bifurcatus* Bright, 2019: 127.

• *Hypothenemus birmanus* (Eichhoff, 1878b: 384) (*Triarmocerus*).

= *Hypothenemus maculicollis* Sharp, 1879: 101.

= *Hypothenemus peritus* Blandford, 1894c: 84.

= *Hypothenemus farinosus* Blandford, 1904: 241.

= *Hypothenemus validus valens* Sampson, 1914: 385.

= *Stephanoderes perkinsi* Hopkins, 1915d: 31.

= *Stephanoderes psidii* Hopkins, 1915d: 32.

= *Stephanoderes sterculiae* Hopkins, 1915d: 32.

= *Stephanoderes alter* Eggers, 1923b: 219.

= *Stephanoderes uter* Eggers, 1923b: 219.

= *Stephanoderes nibarani* Beeson, 1933: 10.

= *Stephanoderes ampliatus* Eggers, 1936a: 627.

= *Stephanoderes pacificus* Beeson, 1940: 197.

= *Stephanoderes castaneus* Wood, 1954b: 1027.

= *Stylolentus dubius* Schedl, 1971e: 372.

• *Hypothenemus biseriatus* (Eggers, 1920a: 240) (*Stephanoderes*).

• *Hypothenemus bolivianus* (Eggers, 1931b: 29) (*Stephanoderes*).

• *Hypothenemus brevicollis* (Eggers, 1927c: 177) (*Stephanoderes*).

= *Stephanoderes lamuensis* Eggers, 1935e: 304.

• *Hypothenemus brevis* (Eggers, 1932b: 30) (*Stephanoderes*).

• *Hypothenemus brunneus* (Hopkins, 1915d: 31) (*Stephanoderes*).

= *Stephanoderes frontalis* Hopkins, 1915d: 31.

= *Hypothenemus cryphalomorphus* Schedl, 1939g: 14.

= *Stephanoderes bituberculatus* Eggers, 1940a: 126.

• *Hypothenemus californicus* Hopkins, 1915d: 19.

= *Stephanoderes gossypii* Hopkins, 1915d: 30.

= *Hypothenemus tritici* Hopkins, 1915d: 19.

= *Hypothenemus thoracicus* Hopkins, 1916: 598.

= *Hypothenemus beameri* Wood, 1954b: 1056.

= *Stephanoderes zeae* Schedl, 1973b: 169.

• *Hypothenemus camerunus* (Eggers, 1922c: 167) (*Stephanoderes*).

= *Stephanoderes brunneipes* Nunberg, 1960: 289.

• *Hypothenemus carbonarius* (Eggers, 1943c: 73) (*Stephanoderes*).

• *Hypothenemus carinafrons* Bright, 2019: 128.

• *Hypothenemus colae* (Schedl, 1957b: 54) (*Stephanoderes*).

• *Hypothenemus collinus* Bright, 2019: 129.

• *Hypothenemus columbi* Hopkins, 1915d: 18.

= *Hypothenemus abdominalis* Hopkins, 1915d: 18.

= *Hypothenemus amplipennis* Hopkins, 1915d: 19.

= *Hypothenemus brunneipennis* Hopkins, 1915d: 18.

= *Hypothenemus rufopalliatus* Hopkins, 1915d: 18.

• *Hypothenemus concavodeclivis* Atkinson & Flechtmann, 2021: 19.

• *Hypothenemus concolor* Hagedorn, 1909: 744.

• *Hypothenemus confusus* (Eggers, 1940e: 235) (*Stephanoderes*).

• *Hypothenemus cordeiroi* Atkinson & Flechtmann, 2021: 16.

• *Hypothenemus costatus* Eichhoff, 1878a: 386 (*Stephanoderes*).

• *Hypothenemus crinatus* Bright, 2019: 131.

• *Hypothenemus criticus* (Schedl, 1937e: 398) (*Cryphalus*).

= *Hypothenemus alternans* Browne, 1970: 554.

• *Hypothenemus crudiae* (Panzer, 1791: 35) (*Bostrichus*).

= *Cryphalus hispidulus* LeConte, 1868: 156.

= *Cryphalus mucronifer* Wollaston, 1868: 116.

= *Hypothenemus nanus* Hagedorn, 1909: 744.

= *Stephanoderes brasiliensis* Hopkins, 1915d: 26.

= *Stephanoderes differens* Hopkins, 1915d: 25.

= *Stephanoderes guatemalensis* Hopkins, 1915d: 26.

= *Stephanoderes lecontei* Hopkins, 1915d: 27.

= *Stephanoderes paraguayensis* Hopkins, 1915d: 26.

= *Stephanoderes trinitatis* Hopkins, 1915d: 28.

= *Stephanoderes fallax* Costa Lima, 1924b: 414 (RN).

= *Stephanoderes largipennis* Toledo Piza, 1924: 354.

= *Stephanoderes polyphagus* Costa Lima, 1924a: 316.

= *Stephanoderes uniseriatus* Eggers, 1924: 103.

= *Stephanoderes obscurus* Eggers, 1929f: 50.

= *Stephanoderes hivaoea* Beeson, 1935a: 105.

= *Stephanoderes lebronneci* Beeson, 1935a: 104.

= *Stephanoderes hawaiiensis* Schedl, 1941b: 112.

= *Hypothenemus ebenus* Wood, 2007: 520.

• *Hypothenemus cryphaloides* (Eichhoff, 1878a: 384) (*Triarmocerus*).

= *Triarmocerus cryphaloides* Eichhoff, 1878b: 119.

• *Hypothenemus cuneolus* (Schedl, 1936c: 24) (*Stephanoderes*).

• *Hypothenemus curtipennis* (Schedl, 1950e: 45) (*Stephanoderes*).

= *Cryphalomorphus samoanus* Browne, 1977a: 61.

• *Hypothenemus cylindraceus* (Schedl, 1972a: 287) (*Stephanoderes*).

• *Hypothenemus cynometrae* Schedl, 1957b: 50.

• *Hypothenemus delicatus* Schedl, 1964h: 44.

• *Hypothenemus deprecator* (Schedl, 1941c: 393) (*Pachynoderes*).

• *Hypothenemus dexter* (Sampson, 1922a: 141) (*Cryphalus*).

• *Hypothenemus dimorphus* (Schedl, 1959g: 168) (*Stephanoderes*).

• *Hypothenemus dipterocarpi* Hopkins, 1915d: 17.

= *Hypothenemus mangarevanus* Beeson, 1940: 196.

• *Hypothenemus discordis* Bright, 2019: 133.

• *Hypothenemus dissimilis* (Zimmermann, 1868: 144) (*Crypturgus*).

= *Stephanoderes chapuisii* Eichhoff, 1872a: 132.

• *Hypothenemus distinctus* Wood, 1954b: 1065.

• *Hypothenemus dolichocola* Hopkins, 1915d: 19.

• *Hypothenemus dolosus* Wood, 1974a: 21.

• *Hypothenemus donisi* (Schedl, 1957b: 46) (*Ericryphalus*).

= *Ericryphalus madagascarensis* Schedl, 1961g: 131.

• *Hypothenemus dorsosignatus* (Schedl, 1950e: 46) (*Stephanoderes*).

= *Stephanopodius fijianus* Schedl, 1955e: 289.

• *Hypothenemus dubitalis* Bright, 2019: 134.

• *Hypothenemus elephas* (Eichhoff, 1872a: 132) (*Stephanoderes*).

= *Adiaeretus spinosus* Hagedorn, 1909: 745.

• *Hypothenemus erectus* LeConte, 1876: 356.

= *Stephanoderes sculpturatus* Eichhoff, 1878b: 146.

= *Hypothenemus validus* Blandford, 1904: 228.

= *Stephanoderes brunneicollis* Hopkins, 1915d: 33.

= *Stephanoderes cubensis* Hopkins, 1915d: 32.

= *Stephanoderes puncticollis* Hopkins, 1915d: 32.

= *Stephanoderes discedens* Schedl, 1950b: 23.

• *Hypothenemus eruditus* (Westwood, 1834: 34) (*Tomicus*).

= *Cryphalus aspericollis* Wollaston, 1860b: 365.

= *Bostrichus boieldieui* Perroud in: Perroud and Montrouzier 1864: 188.

= *Cryphalus obscurus* Ferrari, 1867: 17.

= *Homoeocryphalus ehlersii* Lindemann, 1877a: 168.

= *Stephanoderes germari* Eichhoff, 1878b: 159.

= *Stephanoderes myrmedon* Eichhoff, 1878b: 159.

= *Stephanopodius communis* Schaufuss, 1891: 11.

= *Hypothenemus insularis* Perkins, 1900: 181.

= *Cryphalus tectonae* Stebbing, 1903: 263.

= *Cryphalus basjoo* Niisima, 1910: 9.

= *Cryphalus striatopunctatus* Lea, 1910: 142.

= *Cryphalus tantillus* Lea, 1910: 142.

= *Hypothenemus tuberculosus* Hagedorn, 1912c: 339.

= *Hypothenemus asiminae* Hopkins, 1915d: 16.

= *Hypothenemus bradfordi* Hopkins, 1915d: 15.

= *Stephanoderes elongatus* Hopkins, 1915d: 25.

= *Stephanoderes evonymi* Hopkins, 1915d: 26.

= *Hypothenemus ferrugineus* Hopkins, 1915d: 20.

= *Stephanoderes flavicollis* Hopkins, 1915d: 24.

= *Hypothenemus flavipes* Hopkins, 1915d: 18.

= *Hypothenemus flavosquamosus* Hopkins, 1915d: 15.

= *Hypothenemus hamamelidis* Hopkins, 1915d: 16.

= *Hypothenemus heathi* Hopkins, 1915d: 20.

= *Hypothenemus koebelei* Hopkins, 1915d: 17.

= *Hypothenemus lineatifrons* Hopkins, 1915d: 17.

= *Hypothenemus mali* Hopkins, 1915d: 17.

= *Hypothenemus myristicae* Hopkins, 1915d: 16.

= *Hypothenemus nigricollis* Hopkins, 1915d: 16.

= *Hypothenemus nigripennis* Hopkins, 1915d: 19.

= *Hypothenemus parvus* Hopkins, 1915d: 17.

= *Hypothenemus pruni* Hopkins, 1915d: 16.

= *Hypothenemus punctifrons* Hopkins, 1915d: 18.

= *Hypothenemus punctipennis* Hopkins, 1915d: 20.

= *Stephanoderes pygmaeus* Hopkins, 1915d: 24.

= *Hypothenemus rumseyi* Hopkins, 1915d: 16.

= *Hypothenemus sacchari* Hopkins, 1915d: 17.

= *Cosmoderes schwarzii* Hopkins, 1915d: 11.

= *Stephanoderes subconcentralis* Hopkins, 1915d: 25.

= *Hypothenemus tenuis* Hopkins, 1915d: 16.

= *Stephanoderes unicolor* Hopkins, 1915d: 25.

= *Hypothenemus webbi* Hopkins, 1915d: 17.

= *Hypothenemus bicolor* Eggers, 1920a: 241.

= *Hypothenemus ehlersi rotroui* Peyerimhoff, 1919: 255.

= *Hypothenemus juglandis* Blackman, 1922a: 88.

= *Hypothenemus pusillus* Eggers, 1927c: 173.

= *Stephanoderes intersetosus* Eggers, 1928a: 85.

= *Stephanoderes gracilis* Eggers, 1929f: 51 (RN).

= *Hypothenemus lezhavai* Pyatnitskiy, 1929: 15.

= *Hypothenemus citri* Ebeling, 1935: 21.

= *Hypothenemus erythrinae* Eggers, 1936a: 628.

= *Hypothenemus argentinensis* Schedl, 1939f: 408.

= *Hypothenemus bicolor* Schedl, 1939j: 32.

= *Hypothenemus cylindricus* Schedl, 1939f: 409.

= *Hypothenemus asaroriensis* Beeson, 1940: 195.

= *Hypothenemus dubiosus* Schedl, 1940c: 207.

= *Stephanoderes subcylindricus* Eggers, 1940e: 233.

= *Hypothenemus mauiensis* Schedl, 1941b: 110.

= *Hypothenemus glabratus* Schedl, 1942b: 175.

= *Archeophalus ealensis* Eggers, 1944c: 94.

= *Stephanopodius nanulus* Schedl, 1949b: 263.

= *Hypothenemus parilis* Schedl, 1951c: 100.

= *Hypothenemus hirtipennis* Schedl, 1952a: 450.

= *Hypothenemus longipilis* Schedl, 1952a: 451.

= *Hypothenemus obscuriceps* Schedl, 1952a: 449.

= *Stephanoderes tigrensis* Schedl, 1952a: 452.

= *Hypothenemus glabratellus* Schedl, 1953f: 292.

= *Hypothenemus cylindripennis* Schedl, 1957b: 51.

= *Hypothenemus parcilus* Schedl, 1957b: 49.

= *Hypothenemus vianai* Schedl, 1958j: 42.

= *Hypothenemus mesoleius* Schedl, 1959i: 480.

= *Hypothenemus minutulus* Schedl, 1972h: 225.

= *Cryphalus minutus* Schedl, 1978a: 299.

• *Hypothenemus euphorbiae* (Schedl, 1961g: 135) (*Stephanoderes*).

• *Hypothenemus exceptus* Bright, 2019: 139.

• *Hypothenemus exiguus* (Wood, 1986a: 273) (*Trischidias*).

• *Hypothenemus eximius* Schedl, 1951c: 99.

• *Hypothenemus externedentatus* Schedl, 1959i: 480.

• *Hypothenemus ficivorus* Buhroo & Zubair, 2024: 69.

• *Hypothenemus flavus* Hopkins, 1915d: 17.

• *Hypothenemus foelkelae* Atkinson & Flechtmann, 2021: 19.

• *Hypothenemus fuscicollis* (Eichhoff, 1878b: 148) (*Stephanoderes*).

= *Stephanoderes sundaensis* Eggers, 1927e: 396.

= *Hypothenemus aequaliclavatus* Schedl, 1939j: 33.

= *Ernophloeus costalimai* Nunberg, 1958: 484.

= *Hypothenemus ghanensis* Schedl, 1962g: 67.

= *Hypothenemus comosus* Bright, 1972a: 50.

• *Hypothenemus georgiae* (Hopkins, 1915d: 12) (*Trischidias*).

• *Hypothenemus glabratulus* (Schedl, 1957d: 192) (*Stephanoderes*).

= *Hypothenemus parvistriatus* Wood, 2007: 517.

• *Hypothenemus glabripennis* (Hopkins, 1915d: 32) (*Stephanoderes*).

• *Hypothenemus grandis* Schedl, 1939i: 384.

• *Hypothenemus granulatus* Bright, 2019: 144.

• *Hypothenemus hampei* (Ferrari, 1867: 11) (*Cryphalus*).

= *Stephanoderes coffeae* Hagedorn, 1910: 1.

= *Xyleborus coffeivorus* Van der Weele, 1910: 308.

= *Stephanoderes cooki* Hopkins, 1915d: 27.

= *Xyleborus cofeicola* Campos Novaes, 1922: 67.

= *Stephanoderes punctatus* Eggers, 1924: 101.

= *Stephanoderes glabellus* Schedl, 1952a: 452.

• *Hypothenemus hirsutus* (Wood, 1954b: 1020) (*Stephanoderes*).

• *Hypothenemus hystrix* (Eggers, 1920a: 242) (*Adiaeretus*).

• *Hypothenemus ignotus* Bright, 2019: 146.

• *Hypothenemus improvidus* Bright, 2019: 147.

• *Hypothenemus incognitus* (Schedl, 1967c: 227) (*Stephanoderes*).

• *Hypothenemus indigens* Wood, 1974a: 20.

• *Hypothenemus indigenus* Bright & Peck, 1998: 245.

• *Hypothenemus indistinctus* Bright, 2019: 147.

• *Hypothenemus ingens* (Schedl, 1942d: 18) (*Stephanoderes*).

= *Cryphalomorphus grandis* Schedl, 1969d: 49.

• *Hypothenemus inordinatus* Bright, 2019: 148.

• *Hypothenemus insulanus* Bright in: Bright and Skidmore 2002: 146 (RN).

= *Hypothenemus pacificus* Bright & Peck, 1998: 246.

• *Hypothenemus interstitialis* (Hopkins, 1915d: 28) (*Stephanoderes*).

= *Stephanoderes approximatus* Hopkins, 1915d: 29.

= *Hypothenemus ceibae* Hopkins, 1915d: 20.

= *Stephanoderes flavescens* Hopkins, 1915d: 29.

= *Stephanoderes interpunctus* Hopkins, 1915d: 28.

= *Stephanoderes obliquus* Hopkins, 1915d: 30.

= *Stephanoderes opacipennis* Hopkins, 1915d: 30.

= *Stephanoderes quadridentatus* Hopkins, 1915d: 30.

• *Hypothenemus intricatus* (Schedl, 1950b: 25) (*Stephanoderes*).

• *Hypothenemus japonicus* (Niisima, 1910: 10) (*Cryphalus*).

• *Hypothenemus javanus* (Eggers, 1908a: 215) (*Stephanoderes*).

= *Stephanoderes obesus* Hopkins, 1915d: 30.

= *Stephanoderes philippinensis* Hopkins, 1915d: 31.

= *Stephanoderes bananensis* Eggers, 1922c: 167.

= *Stephanoderes kalshoveni* Schedl, 1939j: 35.

= *Stephanoderes subagnatus* Eggers, 1940d: 101.

= *Stephanoderes pistor* Schedl, 1951c: 102.

= *Stephanoderes prosper* Schedl, 1951c: 103.

• *Hypothenemus kamathi* Beaver, 1995: 16.

• *Hypothenemus kraunhiae* (Murayama, 1950b: 50) (*Cryphalus*).

• *Hypothenemus lefevrei* (Schedl, 1952d: 8) (*Stephanoderes*).

• *Hypothenemus leprieuri* (Perris, 1866: 194) (*Dryocoetes*).

= *Stephanoderes albipilis* Reitter, 1887: 195.

= *Hypothenemus kraussei* Wichmann, 1911b: 210.

• *Hypothenemus leptosquamus* Bright, 2019: 152.

• *Hypothenemus liberiensis* (Hopkins, 1915d: 31) (*Stephanoderes*).

= *Stephanoderes theobromae* Eggers, 1932b: 32.

• *Hypothenemus liliputianus* Bright, 2019: 152.

• *Hypothenemus lineatus* (Eggers, 1927c: 175) (*Stephanoderes*).

• *Hypothenemus longipennis* (Eggers, 1935e: 305) (*Stephanoderes*).

• *Hypothenemus loranthus* (Schedl, 1942d: 8) (*Margadillius*).

• *Hypothenemus lunzi* Atkinson & Flechtmann, 2021: 11.

• *Hypothenemus macrolobii* (Eggers, 1940e: 234) (*Stephanoderes*).

• *Hypothenemus madagascariensis* Schedl, 1953c: 82.

• *Hypothenemus magnus* (Eggers, 1924: 102) (*Stephanoderes*).

• *Hypothenemus major* Browne, 1970: 555.

• *Hypothenemus malayensis* (Schedl, 1977c: 499) (*Lepiceroides*).

• *Hypothenemus mallyi* (Hopkins, 1915d: 32) (*Stephanoderes*).

= *Stephanoderes soussouensis* Eggers, 1943c: 74.

• *Hypothenemus malus* (Schedl, 1957b: 56) (*Stephanoderes*).

• *Hypothenemus mangovorus* Schedl, 1961g: 134.

• *Hypothenemus margaritae* Petrov & Shmaev, 2020: 84.

• *Hypothenemus marginatus* Johnson in: Johnson et al. 2020a: 37 (RN).

= *Periocryphalus sobrinus* Wood, 1974a: 37.

• *Hypothenemus mateui* (Schedl, 1965c: 198) (*Stephanoderes*).

• *Hypothenemus melanarius* (Schedl, 1953c: 80) (*Cosmoderes*).

• *Hypothenemus melasomus* (Lea, 1910: 140) (*Cryphalus*).

• *Hypothenemus meridensis* Wood, 2007: 506.

• *Hypothenemus miles* (LeConte, 1878a: 433) (*Cryphalus*).

• *Hypothenemus minor* (Eggers, 1927c: 178) (*Adiaeretus*).

• *Hypothenemus morigerus* (Schedl, 1957b: 57) (*Stephanoderes*).

• *Hypothenemus morio* (Eggers, 1940d: 101) (*Stephanoderes*).

• *Hypothenemus morosus* Schedl, 1965f: 57.

• *Hypothenemus mozambiquensis* Eggers, 1943c: 75.

• *Hypothenemus mulongensis* (Eggers, 1940e: 235) (*Stephanoderes*).

• *Hypothenemus multidentatulus* (Schedl, 1962d: 95) (*Stephanoderes*) (RN).

= *Stephanoderes multidentatus* Schedl, 1959a: 707.

• *Hypothenemus multipunctatus* (Schedl, 1939j: 36) (*Stephanoderes*).

• *Hypothenemus murariae* Atkinson & Flechtmann, 2021: 9.

• *Hypothenemus muticus* (Schedl, 1961g: 136) (*Stephanoderes*).

• *Hypothenemus namosianus* Browne, 1984d: 78.

• *Hypothenemus nanellus* Wood, 1971a: 34.

• *Hypothenemus nanoparvus* Bright, 2019: 154.

• *Hypothenemus natalensis* (Schedl, 1941c: 392) (*Archeophalus*).

• *Hypothenemus nesiotus* Bright, 2019: 154.

• *Hypothenemus nigropiceus* (Schedl, 1951e: 20) (*Stephanoderes*).

• *Hypothenemus novateutonicus* (Schedl, 1951c: 105) (*Ptilopodius*).

• *Hypothenemus obscurifrons* Bright, 2019: 155.

• *Hypothenemus obscurus* (Fabricius, 1801: 395) (*Hylesinus*).

= *Stephanoderes asperulus* Eichhoff, 1872a: 133.

= *Stephanoderes cassiae* Eichhoff, 1878b: 152.

= *Hypothenemus kunnemanni* Reitter, 1902: 140.

= *Stephanoderes moschatae* Schaufuss, 1905a: 8.

= *Stephanoderes rufescens* Hopkins, 1915d: 29.

= *Stephanoderes amazonicus* Eggers, 1934a: 78.

= *Hypothenemus emarginatus* Schedl, 1942d: 11.

• *Hypothenemus olzenoi* Atkinson & Flechtmann, 2021: 22.

• *Hypothenemus opacus* (Eichhoff, 1872a: 132) (*Stephanoderes*).

• *Hypothenemus paradoxus* Schedl, 1964h: 45.

• *Hypothenemus parasquamosus* Bright, 2019: 160.

• *Hypothenemus parvulosus* Bright, 2019: 160.

• *Hypothenemus parvulus* Browne, 1984e: 93.

• *Hypothenemus paulus* Bright, 2019: 162.

• *Hypothenemus perappositus* (Schedl, 1934a: 91) (*Stephanoderes*).

• *Hypothenemus perexiguus* Bright, 2019: 162.

• *Hypothenemus perhispidus* (Eggers, 1927c: 177) (*Stephanoderes*).

• *Hypothenemus perpunctatus* (Eggers, 1940e: 233) (*Stephanoderes*).

• *Hypothenemus piaparolinae* Johnson, Atkinson & Hulcr, 2016: 418.

• *Hypothenemus pilosus* Hopkins, 1915d: 20.

• *Hypothenemus plumeriae* (Nördlinger, 1856: 74) (*Bostrichus*).

= *Stephanoderes cylindricus* Hopkins, 1915d: 25.

= *Hypothenemus pallidus* Hopkins, 1915d: 18.

= *Stephanoderes parallelus* Hopkins, 1915d: 25.

= *Stephanoderes transatlanticus* Eggers, 1941a: 99.

= *Hypothenemus guadeloupensis* Schedl, 1951c: 98.

= *Stephanoderes ituriensis* Schedl, 1957b: 55.

• *Hypothenemus ponticus* Bright, 2019: 164.

• *Hypothenemus praecellens* (Schedl, 1972a: 288) (*Stephanoderes*).

• *Hypothenemus pubescens* Hopkins, 1915d: 19.

= *Stephanoderes opacifrons* Hopkins, 1915d: 25.

= *Hypothenemus similis* Hopkins, 1915d: 20.

= *Hypothenemus sparsus* Hopkins, 1915d: 20.

= *Hypothenemus subelongatus* Hopkins, 1915d: 19.

= *Stephanoderes tridentatus* Hopkins, 1915d: 31.

= *Hypothenemus minutissimus* Schedl, 1952a: 450.

• *Hypothenemus pubipennis* (Eggers, 1935e: 305) (*Stephanoderes*).

• *Hypothenemus puertoricensis* (Bright & Torres, 2006: 409) (*Trischidias*).

• *Hypothenemus pullus* (Wood, 1971a: 33) (*Periocryphalus*).

• *Hypothenemus pygmaeomorphus* Bright, 2019: 166.

• *Hypothenemus rotundicollis* (Eichhoff, 1878b: 145) (*Stephanoderes*).

= *Stephanoderes quercus* Hopkins, 1915d: 32.

• *Hypothenemus rubrithorax* Bright, 2019: 168.

• *Hypothenemus ruficeps* Perkins, 1900: 181.

• *Hypothenemus rugifer* (Schedl, 1965e: 9) (*Stephanoderes*).

• *Hypothenemus ruginosus* Wood, 1989: 178 (RN).

= *Pachynoderes rugifer* Schedl, 1977a: 395.

• *Hypothenemus rugosicollis* Park, 2025: 52.

• *Hypothenemus rugosipes* Wood, 2007: 515.

• *Hypothenemus sambesianus* Eggers, 1943c: 74.

• *Hypothenemus sapporoensis* (Niisima, 1910: 3) (*Cryphalus*).

• *Hypothenemus sassaensis* (Eggers, 1924: 102) (*Stephanoderes*).

• *Hypothenemus schedli* Browne, 1963b: 54 (RN).

= *Stephanoderes pubescens* Schedl, 1942d: 18.

• *Hypothenemus seriatus* (Eichhoff, 1872a: 133) (*Stephanoderes*).

= *Stephanoderes pulverulentus* Eichhoff, 1872a: 133.

= *Stephanoderes vulgaris* Schaufuss, 1897b: 209.

= *Stephanoderes ferrugineus* Hopkins, 1915d: 29.

= *Stephanoderes ficus* Hopkins, 1915d: 28.

= *Stephanoderes fiebrigi* Hopkins, 1915d: 27.

= *Stephanoderes floridensis* Hopkins, 1915d: 27.

= *Stephanoderes georgiae* Hopkins, 1915d: 26.

= *Stephanoderes lucasi* Hopkins, 1915d: 28.

= *Stephanoderes minutus* Hopkins, 1915d: 26.

= *Stephanoderes multidentatus* Hopkins, 1915d: 28.

= *Stephanoderes niger* Hopkins, 1915d: 31.

= *Stephanoderes nitidifrons* Hopkins, 1915d: 31.

= *Stephanoderes nitidipennis* Hopkins, 1915d: 29.

= *Stephanoderes nitidulus* Hopkins, 1915d: 29.

= *Stephanoderes pecanis* Hopkins, 1915d: 29.

= *Stephanoderes pini* Hopkins, 1915d: 27.

= *Stephanoderes salicis* Hopkins, 1915d: 27.

= *Stephanoderes soltaui* Hopkins, 1915d: 28.

= *Stephanoderes subopacicollis* Hopkins, 1915d: 30.

= *Stephanoderes tamarindi* Hopkins, 1915d: 27.

= *Stephanoderes texanus* Hopkins, 1915d: 26.

= *Stephanoderes virentis* Hopkins, 1915d: 28.

= *Hypothenemus robustus* Blackman, 1922a: 88.

= *Hypothenemus cassavaensis* Schedl, 1938a: 453.

= *Stephanoderes hawaiensis* Schedl, 1941b: 112.

= *Stephanoderes darwinensis* Schedl, 1942c: 178.

= *Stephanoderes striatulus* Schedl, 1942d: 12.

= *Hypothenemus marovoayi* Schedl, 1953c: 81.

= *Stephanoderes andersoni* Wood, 1954b: 1045.

= *Stephanoderes liquidambarae* Wood, 1954b: 1046.

= *Hypothenemus hopkinsi* Browne, 1963b: 53 (RN).

= *Stephanoderes asperatus* Schedl, 1967c: 226.

• *Hypothenemus setiferous* Bright, 2019: 169.

• *Hypothenemus setosus* (Eichhoff, 1867: 391) (*Hypoborus*).

= *Stephanoderes congonus* Hagedorn, 1912c: 337.

• *Hypothenemus simoni* (Reitter, 1887: 194) (*Stephanoderes*).

• *Hypothenemus sobrinus* (Schedl, 1965f: 58) (*Stephanoderes*).

• *Hypothenemus socialis* (Schedl, 1957b: 57) (*Stephanoderes*).

• *Hypothenemus solitarius* (Schedl, 1950b: 24) (*Stephanoderes*).

• *Hypothenemus solivagus* Bright, 2019: 171.

• *Hypothenemus solocis* Wood, 1974a: 21.

• *Hypothenemus sparsedentatus* (Schedl, 1942c: 178) (*Stephanoderes*).

• *Hypothenemus spinatus* (Schedl, 1977b: 282) (*Miocryphalus*).

• *Hypothenemus spinicollis* (Schedl, 1965f: 59) (*Stephanoderes*).

• *Hypothenemus spinosus* (Schedl, 1979e: 98) (*Lepiceroides*).

• *Hypothenemus squamosulus* Johnson in: Johnson et al. 2020a: 38 (RN).

= *Ptilopodius squamosus* Schedl, 1953f: 294.

• *Hypothenemus squamosus* (Hopkins, 1915d: 26) (*Stephanoderes*).

• *Hypothenemus stigmosus* (Schedl, 1951c: 101) (*Stephanoderes*).

= *Stephanoderes garciae* Schedl, 1958j: 42.

• *Hypothenemus striatus* (Atkinson, 1993a: 422) (*Trischidias*).

• *Hypothenemus styrax* (Schedl, 1942b: 177) (*Stephanoderes*).

• *Hypothenemus subacuminatus* (Schedl, 1942b: 177) (*Stephanoderes*).

• *Hypothenemus subsulcatus* Atkinson & Flechtmann, 2021: 5.

• *Hypothenemus subterrestris* Johnson, Atkinson & Hulcr, 2016: 421.

• *Hypothenemus suspectus* Wood, 1974a: 22.

• *Hypothenemus tectus* Bright, 2019: 173.

• *Hypothenemus teretis* Wood, 1971a: 35.

• *Hypothenemus teteforti* (Menier, 1971: 141) (*Stephanoderes*).

• *Hypothenemus tristis* (Eichhoff in: Chapuis and Eichhoff 1876: 200) (*Stephanoderes*).

= *Dryocoetes apatoides* Eichhoff in: Chapuis and Eichhoff 1876: 200.

= *Hypothenemus expers* Blandford, 1894c: 85.

= *Stephanoderes taihokuensis* Schedl, 1952h: 63.

= *Hypothenemus cosmoderoides* Murayama, 1961: 30.

= *Hypothenemus seoulensis* Choo & Woo, 1989: 58.

• *Hypothenemus trivialis* Wood, 1974a: 20.

• *Hypothenemus tuberosus* (Schedl, 1942d: 19) (*Stephanoderes*).

• *Hypothenemus turnbowi* Bright, 2019: 173.

• *Hypothenemus ustulatus* Bright, 2019: 174.

• *Hypothenemus vernaculus* Bright, 2019: 174.

• *Hypothenemus versicolor* Bright, 2019: 176.

• *Hypothenemus vesculus* Wood, 1974a: 21.

• *Hypothenemus villosus* Bright, 2019: 176.

• *Hypothenemus virolae* Wood, 2007: 509.

• *Hypothenemus vitis* Browne, 1970: 554.

• *Hypothenemus wilsoni* Atkinson & Flechtmann, 2021: 7.

• *Hypothenemus winkleri* (Reitter, 1907: 192) (*Stephanoderes*).

• *Hypothenemus woodi* Bright, 2019: 153 (RN).

= *Trischidias minutissima* Wood, 1954b: 1069.

#### *Karlsenius* Jordal

5 spp.

• *Karlsenius attenuatum* (Eggers, 1935e: 306) (*Stephanoderes attenuatus*).

• *Karlsenius ghanaense* (Schedl, 1977b: 281) (*Cryphalomorphus ghanaensis*).

• *Karlsenius klainedoxae* (Schedl, 1957b: 52) (*Miocryphalus*).

• *Karlsenius nigrinum* (Schedl, 1957b: 53) (*Miocryphalus nigrinus*).

• *Karlsenius nitidum* (Schedl, 1965b: 26) (*Eidophelus nitidus*).

#### *Macrocryphalus* Nobuchi

3 spp.

• *Macrocryphalus elongatus* (Schedl, 1966f: 366) (*Miocryphalus*).

• *Macrocryphalus oblongus* Nobuchi, 1981: 86 (*Hypothenemus nobuchii*).

• *Macrocryphalus punctipennis* (Schedl, 1966f: 367) (*Miocryphalus*).

#### *Microcosmoderes* Johnson & Jordal

1 sp.

• *Microcosmoderes shoreae* (Schedl, 1953f: 293) (*Ptilopodius*).

#### *Microsomus* Bright

1 sp.

• *Microsomus atomus* Bright, 2019: 178.

#### *Pygmaeoborus* Bright

1 sp.

• *Pygmaeoborus cubensis* Bright, 2019: 179.

#### *Trypophloeus* Fairmaire

15 spp. (1 sp. with 2 sspp.).

• *Trypophloeus alni* (Lindemann, 1875a: 136) (*Cryphalus*).

= *Trypophloeus holdhausi* Wichmann, 1912: 186.

• *Trypophloeus binodulus* (Ratzeburg, 1837: 163) (*Bostrichus*).

= *Cryphalus grothii* Hagedorn, 1904d: 232.

= *Trypophloeus spiculatus* Eggers, 1927a: 122.

= *Trypophloeus populi* Kurentsov, 1941: 164.

= *Trypophloeus berezinae* Stark, 1952: 285.

= *Trypophloeus kurenzovi* Nunberg, 1956c: 208 (RN).

= *Trypophloeus kurenzowi* Schedl, 1959d: 42.

• *Trypophloeus bispinulus* Eggers, 1927a: 121.

• *Trypophloeus borealis* Kvamme, Mandelshtam, Salnitska, Ojeda & Lindelöw, 2021: 54.

• *Trypophloeus dejevi* Stark, 1936: 152.

= *Trypophloeus niger* Stark, 1936: 152.

= *Trypophloeus dejevi* Eggers, 1942a: 31.

• *Trypophloeus discedens* Palm, 1950: 142.

= *Trypophloeus palmi* Hansen, 1956: 183.

• *Trypophloeus grandis* Schedl, 1964c: 99.

• *Trypophloeus granulatus* (Ratzeburg, 1837: 164) (*Bostrichus*).

• *Trypophloeus klimeschi* Eggers, 1915c: 188.

• *Trypophloeus nitidus* (Swaine, 1912: 349) (*Cryphalus*).

= *Trypophloeus punctipennis* Hopkins, 1915d: 37.

• *Trypophloeus populi* Hopkins, 1915d: 37.

• *Trypophloeus rybinskii corsicus* Eggers, 1912a: 113.

• *Trypophloeus rybinskii rybinskii* (Reitter, 1895: 72) (*Cryphalus rybinskii*).

• *Trypophloeus salicis* Hopkins, 1915d: 36.

= *Trypophloeus concentralis* Hopkins, 1915d: 36.

• *Trypophloeus thatcheri* (Wood, 1954b: 994) (*Cryphalus*).

• *Trypophloeus tremulae* Stark, 1952: 287.

### XYLEBORINI LeConte

43 genera, 1349 spp.

#### *Amasa* Lea

52 spp.

• *Amasa aglaiae* (Browne, 1984a: 156) (*Xyleborus*).

• *Amasa anomala* (Schedl, 1955e: 298) (*Xyleborus anomalus*).

= *Xyleborus nakazawai* Browne, 1984a: 156.

• *Amasa aspersa* Sampson, 1921: 31 (*Xyleborus*) (E).

• *Amasa banksiae* (Schedl, 1964d: 213) (*Xyleborus*).

• *Amasa batoerradensis* (Schedl, 1939j: 39) (*Pseudoxyleborus*).

• *Amasa beaveri* Sittichaya & Smith, 2022: 198.

• *Amasa beesoni* (Eggers, 1930b: 207) (*Pseudoxyleborus*).

• *Amasa bicostata* (Sampson, 1921: 28) (*Xyleborus bicostatus*).

• *Amasa brevipennis* (Schedl, 1971e: 378) (*Xyleborus*).

• *Amasa circumcisula* (Schedl, 1954c: 140) (*Xyleborus circumcisulus*).

• *Amasa cognatoi* Sittichaya & Smith, 2022: 199.

• *Amasa concitata* Schedl, 1969a: 214 (*Xyleborus*) (E).

• *Amasa consularis* (Schedl, 1955e: 299) (*Xyleborus*).

• *Amasa cycloxyster* Smith, Beaver & Cognato, 2020: 33.

• *Amasa cylindrotomica* (Schedl, 1939j: 40) (*Pseudoxyleborus cylindrotomicus*).

= *Xyleborus semitruncatus* Schedl, 1942d: 35.

= *Xyleborus truncatellus* Schedl, 1951i: 79.

= *Xyleborus jucundus* Schedl, 1954c: 138 (RN).

• *Amasa darwini* (Schedl, 1972e: 148) (*Xyleborus*).

• *Amasa dasyura* (Browne, 1950: 645) (*Xyleborus dasyurus*).

• *Amasa doliaris* (Schedl, 1959i: 511) (*Xyleborus*).

• *Amasa eugeniae* (Eggers, 1930b: 183) (*Xyleborus*).

• *Amasa exacta* (Schedl, 1964e: 246) (*Xyleborus exactus*).

• *Amasa foveicollis* (Browne, 1950: 646) (*Xyleborus*).

• *Amasa fulgens* (Schedl, 1975a: 365) (*Xyleborus*).

• *Amasa fuscipilosa* (Eggers, 1940f: 137) (*Xyleborus fuscipilosus*).

• *Amasa galeoderma* Smith, Beaver & Cognato, 2020: 36.

• *Amasa gibbosa* Smith, Beaver & Cognato, 2020: 38.

• *Amasa glauca* (Sampson, 1921: 30) (*Xyleborus glaucus*).

• *Amasa kenchingtoni* Beaver, 1995: 13.

• *Amasa knizeki* Hulcr & Cognato, 2013: 30.

• *Amasa latetruncata* (Schedl, 1942b: 190) (*Xyleborus latetruncatus*).

• *Amasa laticaudata* (Eggers, 1923b: 168) (*Xyleborus laticaudatus*).

• *Amasa lini* Smith, Beaver & Cognato, 2020: 40.

• *Amasa macarthorum* Sittichaya & Smith, 2022: 200.

• *Amasa nitidior* (Eggers, 1940f: 136) (*Xyleborus*).

• *Amasa nobilis* (Eggers, 1940f: 134) (*Pseudoxyleborus*).

• *Amasa omissa* (Schedl, 1961g: 153) (*Xylosandrus omissus*).

• *Amasa opalescens* (Schedl, 1937f: 550) (*Xyleborus*).

• *Amasa oralis* (Schedl, 1961g: 164) (*Xyleborus*).

• *Amasa orbicaudata* (Eggers, 1940f: 135) (*Xyleborus orbicaudatus*).

• *Amasa parviseta* Knížek & Smith, 2024: 386.

• *Amasa resecta* Eggers, 1927e: 391 (*Xyleborus*) (E).

= *Xyleborus abruptus* Eggers, 1923b: 169.

= *Xyleborus opacicauda* Eggers, 1940f: 136.

• *Amasa schlichii* (Stebbing, 1914: 592) (*Xyleborus*).

= *Acanthotomicus truncatus* Stebbing, 1907: 40.

= *Xyleborus glaber* Eggers, 1930b: 185.

= *Xyleborus uniseriatus* Eggers, 1936e: 89.

= *Xyleborus verax* Schedl, 1939j: 43.

• *Amasa setosa* Lin, Li, Smith & Meng, 2024a: 187.

• *Amasa similis* (Eggers, 1923b: 213) (*Xyleboricus*).

= *Xyleborus cylindriformis* Schedl, 1942c: 190.

= *Xyleborus tereticollis* Schedl, 1951i: 82.

= *Xyleborus obscurus* Schedl, 1972c: 259 (RN).

= *Xyleborus circulicaudus* Browne, 1974a: 68.

• *Amasa sirambeana* (Eggers, 1923b: 169) (*Xyleborus sirambeanus*).

• *Amasa striatotruncata* (Schedl, 1936c: 29) (*Xyleborus striatotruncatus*).

• *Amasa tropidacron* Smith, Beaver & Cognato, 2020: 44.

• *Amasa truncata* (Erichson, 1842: 212) (*Tomicus truncatus*).

= *Amasa thoracica* Lea, 1894: 322.

• *Amasa truncatifera* (Schedl, 1955e: 309) (*Xyleborus truncatiferus*).

• *Amasa umbratula* (Schedl, 1975h: 221) (*Xyleborus umbratulus*).

• *Amasa versicolor* (Sampson, 1921: 29) (*Xyleborus*).

• *Amasa youlii* Smith, Beaver & Cognato, 2020: 47.

• *Amasa yunnanensis* Lin, Li, Smith & Meng, 2024a: 186.

#### *Ambrosiodmus* Hopkins

60 spp.

• *Ambrosiodmus addendus* (Schedl, 1964a: 70) (*Xyleborus*).

• *Ambrosiodmus adustus* (Eggers, 1932c: 295) (*Xyleborus*).

• *Ambrosiodmus aegir* (Eggers, 1922c: 171) (*Xyleborus*).

= *Xyleborus holtzi* Eggers, 1922c: 171.

= *Xyleborus scabridus* Schedl, 1952b: 16.

• *Ambrosiodmus albizzianus* Schedl, 1950b: 30.

• *Ambrosiodmus alexae* Wood, 2007: 406.

• *Ambrosiodmus alsapanicus* (Schedl, 1951i: 59) (*Xyleborus*).

• *Ambrosiodmus anepotulus* (Eggers, 1940f: 138) (*Xyleborus*).

• *Ambrosiodmus artegranulatus* (Schedl, 1937e: 400) (*Xyleborus*).

• *Ambrosiodmus asperatus* (Blandford, 1895: 321) (*Xyleborus*).

= *Xyleborus nepotulus* Eggers, 1923b: 179.

= *Xyleborus citri* Beeson, 1930: 215.

= *Xyleborus nepotulomorphus* Eggers, 1936e: 88.

• *Ambrosiodmus bostrichoides* (Schedl, 1956b: 33) (*Xyleborus*).

• *Ambrosiodmus brunneipes* (Eggers, 1940f: 138) (*Xyleborus*).

• *Ambrosiodmus camphorae* (Hagedorn, 1908: 378) (*Xyleborus*).

• *Ambrosiodmus coffeiceus* (Schedl, 1951d: 376) (*Xyleborus*).

• *Ambrosiodmus conspectus* (Schedl, 1964i: 247) (*Xyleborus*).

• *Ambrosiodmus devexulus* (Wood, 1978: 398) (*Xyleborus*) (RN).

= *Xyleborus devexus* Wood, 1977a: 219.

= *Xyleborus woodi* Schedl, 1979c: 127 (RN).

• *Ambrosiodmus devexus* (Schedl, 1977d: 45) (*Xyleborus*).

• *Ambrosiodmus diversipennis* (Schedl, 1951e: 23) (*Xyleborus*).

• *Ambrosiodmus eichhoffi* (Schreiner, 1882: 248) (*Xyleborus*).

= *Xyleborus congonus* Hagedorn, 1908: 379.

• *Ambrosiodmus facetus* (Schedl, 1965e: 12) (*Xyleborus*).

• *Ambrosiodmus ferus* Wood, 1986a: 269.

• *Ambrosiodmus fraterculus* (Schaufuss, 1905d: 19) (*Xyleborus*).

• *Ambrosiodmus funebris* (Schedl, 1976: 73) (*Xyleborus*).

• *Ambrosiodmus hagedorni* (Iglesias, 1914: 128) (*Xyleborus*).

= *Ambrosiodmus guatemalensis* Hopkins, 1915d: 56.

= *Ambrosiodmus lecontei* Hopkins, 1915d: 56.

= *Xyleborus gundlachi* Eggers, 1931b: 30.

= *Xyleborus anisandrus* Schedl, 1954b: 44.

• *Ambrosiodmus infidelis* Bright, 2019: 254.

• *Ambrosiodmus innominatus* (Schedl, 1970b: 236) (*Xyleborus*).

• *Ambrosiodmus inoblitus* (Schedl, 1939c: 173) (*Xyleborus*).

• *Ambrosiodmus lewisi* (Blandford, 1894c: 104) (*Xyleborus*).

= *Ozopemon tuberculatus* Strohmeyer, 1912b: 38.

= *Xyleborus lewekianus* Eggers, 1923b: 181.

= *Xyleborus tegalensis* Eggers, 1923b: 181.

• *Ambrosiodmus loebli* (Schedl, 1979h: 413) (*Xyleborus*).

• *Ambrosiodmus mahafali* (Schedl, 1953c: 96) (*Xyleborus*).

• *Ambrosiodmus minor* (Stebbing, 1907: 37) (*Phloeosinus*).

= *Phloeosinus minor* Stebbing, 1909: 20.

= *Xyleborus crassus* Hagedorn, 1910: 8.

• *Ambrosiodmus natalensis* (Schaufuss, 1891: 20) (*Xyleborus*).

• *Ambrosiodmus neglectus* (Schedl, 1957b: 16) (*Xyleborus*) (RN).

= *Xyleborus scabrior* Schedl, 1954d: 79.

• *Ambrosiodmus nepocranus* (Schedl, 1939j: 45) (*Xyleborus*).

• *Ambrosiodmus nuperus* (Bright, 1972a: 76) (*Xyleborus*).

• *Ambrosiodmus obliquus* (LeConte, 1878a: 432) (*Pityophthorus*).

= *Xyleborus gilvipes* Blandford, 1898a: 205.

= *Ambrosiodmus linderae* Hopkins, 1915d: 56.

= *Xyleborus brasiliensis* Eggers, 1928a: 96.

= *Xyleborus mexicanus* Eggers, 1931a: 19.

= *Xyleborus illepidus* Schedl, 1941c: 402.

= *Xyleborus pseudobrasiliensis* Eggers, 1941a: 101.

= *Xyleborus melanarius* Schedl, 1978a: 307.

= *Ambrosiodmus klapperichi* Bright, 1985a: 178.

• *Ambrosiodmus obscuripennis* Park, 2025: 122.

• *Ambrosiodmus ocellatus* (Wood, 1974a: 27) (*Xyleborus*).

• *Ambrosiodmus opacithorax* (Schedl, 1937e: 402) (*Xyleborus*).

• *Ambrosiodmus opimus* (Wood, 1974a: 37) (*Xyleborus*).

• *Ambrosiodmus optatus* (Schedl, 1973d: 92) (*Xyleborus*).

• *Ambrosiodmus ovatus* (Eggers, 1932c: 298) (*Xyleborus*).

= *Xyleborus tenebrosus* Schedl, 1937e: 402.

• *Ambrosiodmus pardous* (Eggers, 1943a: 247) (*Xyleborus*).

• *Ambrosiodmus paucus* Wood, 1986a: 269.

• *Ambrosiodmus pithecolobius* (Schedl, 1937d: 13) (*Xyleborus*).

• *Ambrosiodmus pseudocitri* (Schedl, 1959i: 494) (*Xyleboricus*).

• *Ambrosiodmus raucus* (Schedl, 1950g: 209) (*Xyleborus*).

• *Ambrosiodmus rubricollis* (Eichhoff in: Chapuis and Eichhoff 1876: 202) (*Xyleborus*).

= *Xyleborus taboensis* Schedl, 1952h: 65.

= *Xyleborus strohmeyeri* Schedl, 1975j: 457.

• *Ambrosiodmus rugicollis* (Blandford, 1898a: 207) (*Xyleborus*).

• *Ambrosiodmus rusticus* (Wood, 1974a: 36) (*Xyleborus*).

• *Ambrosiodmus sakoae* (Schedl, 1961g: 144) (*Xyleborus*).

• *Ambrosiodmus sandragotoensis* (Schedl, 1961g: 148) (*Xyleborus*).

• *Ambrosiodmus scalaris* (Schedl, 1935c: 95) (*Xyleborus*).

• *Ambrosiodmus signiceps* (Schedl, 1963g: 185) (*Xyleborus*).

• *Ambrosiodmus signifer* (Schedl, 1968b: 146) (*Xyleborus*).

• *Ambrosiodmus spinosus* Pérez, Pérez de la Cruz & Equihua in: Pérez et al. 2020: 445.

• *Ambrosiodmus tachygraphus* (Zimmermann, 1868: 144) (*Xyleborus*).

• *Ambrosiodmus triton* (Schaufuss, 1905b: 12) (*Xyleborus*).

• *Ambrosiodmus tropicus* (Hagedorn, 1910: 12) (*Xyleborus*).

• *Ambrosiodmus trux* (Schedl, 1937e: 400) (*Xyleborus*).

• *Ambrosiodmus turgidus* (Schedl, 1963g: 188) (*Xyleborus*).

#### *Ambrosiophilus* Hulcr & Cognato

27 spp.

• *Ambrosiophilus atratus* (Eichhoff in: Chapuis and Eichhoff 1876: 201) (*Xyleborus*).

= *Xyleborus collis* Niisima, 1910: 12.

• *Ambrosiophilus bispinosulus* (Schedl, 1961f: 73) (*Xyleborus*).

• *Ambrosiophilus caliginestris* Smith, Beaver & Cognato, 2020: 58.

• *Ambrosiophilus compressus* (Lea, 1894: 321) (*Xylopertha compressa*).

• *Ambrosiophilus consimilis* (Eggers, 1923b: 180) (*Xyleborus*).

• *Ambrosiophilus cristatulus* (Schedl, 1953f: 300) (*Xyleborus*).

• *Ambrosiophilus immitatrix* (Schedl, 1975a: 367) (*Xyleborus*).

= *Xyleborus imitatrix* Schedl, 1980c: 124.

• *Ambrosiophilus indicus* Smith, Beaver & Cognato, 2020: 61.

• *Ambrosiophilus lannaensis* Smith, Beaver & Cognato, 2020: 62.

• *Ambrosiophilus latecompressus* (Schedl, 1936e: 532) (*Xyleborus*).

• *Ambrosiophilus latisulcatus* (Eggers, 1940f: 142) (*Xyleborus*).

• *Ambrosiophilus mogia* Hulcr & Cognato, 2013: 37.

• *Ambrosiophilus osumiensis* (Murayama, 1934a: 292) (*Xyleborus*).

= *Xyleborus metanepotulus* Eggers, 1939a: 119.

= *Xyleborus nodulosus* Eggers, 1941c: 223.

= *Xyleborus pernodulus* Schedl, 1957b: 85 (RN).

= *Xyleborus hunanensis* Browne, 1983a: 33.

= *Ambrosiophilus peregrinus* Smith & Cognato, 2015: 216.

• *Ambrosiophilus papilliferus* Smith, Beaver & Cognato, 2020: 67.

• *Ambrosiophilus pertortuosus* (Schedl, 1942b: 186) (*Xyleborus*).

• *Ambrosiophilus pityogenes* (Schedl, 1936e: 534) (*Xyleborus*).

• *Ambrosiophilus restrictus* (Schedl, 1939j: 46) (*Xyleborus*).

= *Xyleborus incertus* Schedl, 1970h: 217.

= *Xyleborus devius* Schedl, 1979e: 106.

= *Xyleborus australis* Schedl, 1980b: 185.

• *Ambrosiophilus satoi* (Schedl, 1966e: 39) (*Xyleborus*).

• *Ambrosiophilus semicarinatus* (Schedl, 1942c: 191) (*Xyleborus*).

= *Xyleborus nitens* Browne, 1984e: 97.

• *Ambrosiophilus semirufus* (Schedl, 1959i: 499) (*Xyleborus*).

• *Ambrosiophilus sexdentatus* (Eggers, 1940f: 148) (*Xyleborus*).

• *Ambrosiophilus subnepotulus* (Eggers, 1930b: 178) (*Xyleborus*).

= *Xyleborus cristatuloides* Schedl, 1971b: 284.

• *Ambrosiophilus sulcatus* (Eggers, 1930b: 180) (*Xyleborus*).

= *Xyleborus sulcatulus* Eggers, 1939b: 13.

= *Xyleborus sinensis* Eggers, 1941c: 224.

• *Ambrosiophilus tomicoides* (Eggers, 1923b: 205) (*Xyleborus*).

• *Ambrosiophilus tortuosus* (Schedl, 1942b: 186) (*Xyleborus*).

• *Ambrosiophilus wantaneeae* Smith, Beaver & Cognato, 2020: 72.

• *Ambrosiophilus wilderi* (Beeson, 1929: 235) (*Xyleborus*).

= *Xyleborus upoluensis* Schedl, 1951f: 152.

#### *Ancipitis* Hulcr & Cognato

3 spp.

• *Ancipitis puer* (Eggers, 1923b: 191) (*Xyleborus*).

= *Xyleborus ceramensis* Schedl, 1937f: 549.

• *Ancipitis punctatissimus* (Eichhoff, 1880: 189) (*Xyleborus*).

= *Xyleborus spatulatus* Blandford, 1896e: 218.

• *Ancipitis scabrior* (Schedl, 1953f: 304) (*Xyleborus*).

#### *Anisandrus* Ferrari

43 spp.

• *Anisandrus achaete* Smith, Beaver & Cognato, 2020: 80.

• *Anisandrus apicalis* (Blandford, 1894c: 105) (*Xyleborus*).

• *Anisandrus auco* Smith, Beaver & Cognato, 2020: 83.

• *Anisandrus auratipilus* Smith, Beaver & Cognato, 2020: 84.

• *Anisandrus bolavenensis* Sittichaya & Smith, 2024: 2.

• *Anisandrus butamali* (Beeson, 1930: 216) (*Xyleborus*).

• *Anisandrus carinensis* (Eggers, 1923b: 180) (*Xyleborus*).

• *Anisandrus congruens* Smith, Beaver & Cognato, 2020: 86.

• *Anisandrus cornutus* (Schaufuss, 1891: 17) (*Xyleborus*).

• *Anisandrus cristatus* (Hagedorn, 1908: 377) (*Xyleborus*).

= *Xyleborus fabricii* Schedl, 1964f: 217 (RN).

• *Anisandrus cryphaloides* Smith, Beaver & Cognato, 2020: 89.

• *Anisandrus dispar* (Fabricius, 1792: 363) (*Apate*).

= *Bostrichus dispar* Herbst, 1793: 113.

= *Bostrichus brevis* Panzer, 1795: 288.

= *Bostrichus thoracicus* Panzer, 1795: 288.

= *Bostrichus rufipes* Olivier, 1800b: 17.

= *Scolytus pyri* Peck, 1817: 207.

= *Bostrichus tachygraphus* Sahlberg, 1836a: 152.

= *Bostrichus ratzeburgii* Kolenati, 1846: 39.

= *Xyleborus ishidai* Niisima, 1909: 156.

= *Anisandrus aequalis* Reitter, 1913: 81.

= *Anisandrus swainei* Drake, 1921: 203.

= *Xyleborus dispar rugulosus* Eggers, 1922a: 17.

= *Xyleborus cerasi* Eggers, 1937a: 335.

= *Xyleborus khinganensis* Murayama, 1943: 100.

• *Anisandrus eggersi* (Beeson, 1930: 215) (*Xyleborus*).

• *Anisandrus feronia* Smith, Beaver & Cognato, 2020: 92.

• *Anisandrus geminatus* (Hagedorn, 1904b: 126) (*Xyleborus*).

• *Anisandrus hera* Smith, Beaver & Cognato, 2020: 95.

• *Anisandrus hirtus* (Hagedorn, 1904b: 126) (*Xyleborus*).

= *Xyleborus hagedorni* Stebbing, 1914: 596.

= *Xyleborus hirtuosus* Beeson, 1930: 217.

= *Xyleborus hagedornianus* Schedl, 1952f: 164 (RN).

= *Xyleborus tectonae* Nunberg, 1956c: 209 (RN).

= *Xyleborus hirtipes* Schedl, 1969d: 53.

= *Xyleborus taiwanensis* Browne, 1980b: 386.

• *Anisandrus improbus* (Sampson, 1913: 444) (*Xyleborus*).

• *Anisandrus klapperichi* (Schedl, 1955f: 46) (*Xyleborus*).

• *Anisandrus lineatus* (Eggers, 1930b: 177) (*Xyleborus*).

= *Xyleborus melancranis* Beeson, 1930: 218.

• *Anisandrus longidens* (Eggers, 1930b: 181) (*Xyleborus*).

• *Anisandrus maiche* (Kurentsov, 1941: 142) (Xyleborus).

= *Xyleborus maiche* Eggers, 1942a: 36.

• *Anisandrus montanus* Sittichaya, Smith & Beaver, 2023a: 291.

• *Anisandrus mussooriensis* (Eggers, 1930b: 179) (*Xyleborus*).

• *Anisandrus niger* (Sampson, 1912: 247) (*Xyleborus*).

• *Anisandrus obesus* (LeConte, 1868: 159) (*Xyleborus*).

= *Xyleborus serratus* Swaine, 1910b: 162.

= *Anisandrus populi* Swaine, 1917: 22.

• *Anisandrus ovalis* Park, 2025: 130.

• *Anisandrus paragogus* Smith, Beaver & Cognato, 2020: 104.

• *Anisandrus percristatus* (Eggers, 1939b: 12) (*Xyleborus*).

• *Anisandrus phithakpa* Sittichaya, Smith & Beaver, 2023a: 293.

• *Anisandrus proscissus* Smith, Beaver, Pham & Cognato, 2022: 2.

• *Anisandrus sayi* Hopkins, 1915d: 68.

= *Xyleborus obesus minor* Swaine, 1910b: 164.

= *Xyleborus neardus* Schedl, 1950a: 893 (RN).

• *Anisandrus simplex* Smith, Beaver & Cognato in: Smith et al. 2022: 3.

• *Anisandrus sinivali* Smith, Beaver & Cognato, 2020: 107.

• *Anisandrus tanaosi* Sittichaya, Smith & Beaver, 2023a: 295.

• *Anisandrus triton* Sittichaya, Smith & Beaver, 2023a: 297.

• *Anisandrus tuberculatus* Lin, Smith, Xu & Li, 2025: 196.

• *Anisandrus uniseriatus* Sittichaya, Smith & Beaver, 2023a: 299.

• *Anisandrus ursa* (Eggers, 1923b: 172) (*Xyleborus*).

• *Anisandrus ursinus* (Hagedorn, 1908: 381) (*Xyleborus*).

• *Anisandrus ursulus* (Eggers, 1923b: 173) (*Xyleborus*).

= *Xyleborus lativentris* Schedl, 1942b: 191.

• *Anisandrus venustus* Smith, Beaver & Cognato, 2020: 109.

• *Anisandrus xuannu* Smith, Beaver & Cognato, 2020: 111.

#### *Arixyleborus* Hopkins

46 spp.

• *Arixyleborus abruptus* Schedl, 1975a: 358.

• *Arixyleborus belalongi* Smith, Beaver & Cognato in: Smith et al. 2022: 5.

• *Arixyleborus canaliculatus* (Eggers, 1923b: 216) (*Xyleboricus*).

= *Amasa subsimilis* Schedl, 1970f: 362.

= *Arixyleborus cariniceps* Schedl, 1975a: 358.

• *Arixyleborus castaneae* Schedl, 1958i: 146.

• *Arixyleborus confinis* (Eggers, 1927b: 106) (*Webbia*).

• *Arixyleborus crassior* Smith, Beaver & Cognato, 2020: 115.

• *Arixyleborus crenulatus* (Eggers, 1920b: 117) (*Xyleborus*).

= *Xyleborus mpangae* Browne, 1965: 202.

• *Arixyleborus fuliginosus* (Eggers, 1923b: 205) (*Xyleborus*).

• *Arixyleborus gedeanus* (Schedl, 1942d: 26) (*Xyleboricus*).

• *Arixyleborus grandis* (Schedl, 1942d: 27) (*Xyleboricus*).

• *Arixyleborus granifer* (Eichhoff, 1878a: 391) (*Xyleborus*).

= *Xyleborus granifer* Eichhoff, 1878b: 502.

= *Xyleborus granifer borneensis* Schedl, 1965b: 27.

• *Arixyleborus granulicauda* Schedl, 1975a: 359.

• *Arixyleborus granulifer* (Eggers, 1923b: 206) (*Xyleborus*).

• *Arixyleborus guttifer* (Schedl, 1955e: 297) (*Xyleboricus*).

• *Arixyleborus halabala* Sittichaya, Smith & Beaver in: Sittichaya et al. 2024a: 65.

• *Arixyleborus hirsutulus* Schedl, 1969a: 212.

• *Arixyleborus imitator* (Eggers, 1927b: 105) (*Webbia*).

= *Xyleborus granistriatus* Eggers, 1940f: 147.

= *Arixyleborus dipterocarpi* Browne, 1981a: 133.

• *Arixyleborus iriani* Browne, 1983b: 560.

• *Arixyleborus leprosulus* Schedl, 1953f: 300.

= *Arixyleborus aralidii* Nunberg, 1961a: 618.

• *Arixyleborus liratus* Sittichaya, Smith & Beaver in: Sittichaya et al. 2024a: 68.

• *Arixyleborus longicauda* Sittichaya, Smith & Beaver in: Sittichaya et al. 2024a: 70.

• *Arixyleborus malayensis* (Schedl, 1954c: 150) (*Xyleboricus*).

• *Arixyleborus mediosectus* (Eggers, 1923b: 215) (*Xyleboricus*).

= *Xyleboricus angulatus* Schedl, 1942b: 183.

• *Arixyleborus minor* (Eggers, 1940f: 134) (*Xyleboricus*).

= *Arixyleborus trux* Schedl, 1975a: 359.

• *Arixyleborus moestus* (Eggers, 1930b: 189) (*Xyleborus*).

• *Arixyleborus nudulus* Smith, Rabaglia & Cognato in: Smith et al. 2018: 841.

• *Arixyleborus okadai* Browne, 1985b: 293.

• *Arixyleborus perbrevis* Sittichaya & Smith in: Sittichaya et al. 2025: 5.

• *Arixyleborus phiaoacensis* Smith, Beaver & Cognato, 2020: 124.

• *Arixyleborus pilosus* (Eggers, 1923b: 207) (*Xyleborus*).

• *Arixyleborus puberulus* (Blandford, 1896e: 215) (*Xyleborus*).

= *Xyleborus morio* Eggers, 1923b: 207.

= *Xyleborus hirtipennis* Eggers, 1940f: 146.

• *Arixyleborus resecans* (Eggers, 1930b: 184) (*Xyleborus*).

• *Arixyleborus rugosipes* Hopkins, 1915d: 59.

= *Webbia medius* Eggers, 1927b: 104.

= *Webbia camphorae* Eggers, 1936a: 634.

• *Arixyleborus scabripennis* (Blandford, 1896e: 216) (*Xyleborus*).

• *Arixyleborus scapularis* (Schedl, 1942b: 193) (*Xyleborus*).

= *Arixyleborus magnus* Browne, 1985a: 192.

• *Arixyleborus seminitens* (Blandford, 1895: 322) (*Xyleborus*).

• *Arixyleborus setosus* Smith, Beaver & Cognato, 2020: 130.

• *Arixyleborus silvanus* Smith, Beaver & Cognato, 2020: 132.

• *Arixyleborus simplicaudus* Hulcr & Cognato, 2013: 49.

• *Arixyleborus sittichayai* Smith, Beaver & Cognato, 2020: 133.

• *Arixyleborus strombosiopsis* (Schedl, 1957b: 91) (*Xyleborus*).

• *Arixyleborus suturalis* (Eggers, 1936e: 91) (*Xyleboricus*).

• *Arixyleborus titanus* Smith, Beaver & Cognato, 2020: 135.

• *Arixyleborus tuberculatus* (Eggers, 1940f: 133) (*Xyleboricus*).

• *Arixyleborus vellus* Sittichaya, Beaver & Smith, 2024a: 72.

• *Arixyleborus yakushimanus* (Murayama, 1955: 83) (*Xyleborus*).

#### *Beaverium* Hulcr & Cognato

16 spp.

• *Beaverium batoensis* (Eggers, 1923b: 178) (*Xyleborus*).

• *Beaverium brevicaudatus* Smith, Beaver & Cognato in: Smith et al. 2022: 6.

• *Beaverium calvus* (Schedl, 1942b: 187) (*Xyleborus*).

• *Beaverium dihingicus* (Wood, 1992: 79) (*Cyclorhipidion dihingicum*) (RN).

= *Xyleborus dihingensis* Schedl, 1951i: 75.

• *Beaverium insulindicus* (Eggers, 1923b: 177) (*Xyleborus*).

= *Xyleborus annexus* Schedl, 1973d: 89.

= *Xyleborus depressurus* Browne, 1985a: 192.

• *Beaverium lantanae* (Eggers, 1930b: 180) (*Xyleborus*).

• *Beaverium latus* (Eggers, 1923b: 177) (*Xyleborus*).

• *Beaverium magnus* (Niisima, 1910: 11) (*Xyleborus*).

= *Xyleborus rufobrunneus dihingensis* Eggers, 1930b: 189.

= *Xyleborus chujoi* Schedl, 1951i: 73.

• *Beaverium obstipus* (Schedl, 1935g: 270) (*Xyleborus*).

• *Beaverium perplexus* (Schedl, 1970h: 218) (*Xyleborus*).

= *Xyleborus platyurus* Browne, 1983c: 74.

• *Beaverium rufonitidus* (Schedl, 1951i: 74) (*Xyleborus*).

• *Beaverium rufus* (Schedl, 1951i: 74) (*Xyleborus*).

• *Beaverium rugipunctus* Hulcr & Cognato, 2013: 54.

• *Beaverium sundaensis* (Eggers, 1923b: 175) (*Xyleborus*).

= *Xyleborus aplanatideclivis* Schedl, 1942c: 191.

• *Beaverium swezeyi* (Beeson, 1929: 234) (*Xyleborus*).

• *Beaverium venustulus* (Schedl, 1970h: 219) (*Xyleborus*).

#### *Callibora* Cognato

1 sp.

• *Callibora sarahsmithae* Cognato, 2018: 801.

#### *Cnestus* Sampson

30 spp.

• *Cnestus ater* (Eggers, 1923b: 210) (*Xyleborus*).

= *Xyleborus retusiformis* Schedl, 1936c: 31.

• *Cnestus aterrimus* (Eggers, 1927e: 400) (*Xyleborus*).

= *Xyleborus pallidipennis* Eggers, 1940f: 143.

= *Xyleborus glaberrimus* Schedl, 1942c: 189.

= *Xyleborus glabripennis* Schedl, 1942b: 189.

= *Tosaxyleborus pallidipennis* Murayama, 1950b: 49.

= *Xyleborus nitens* Browne, 1955: 358.

= *Cnestus murayamai* Schedl, 1962c: 207 (RN).

= *Cnestus murayamai* Browne, 1963b: 54 (RN).

= *Cnestus pseudosuturalis* Schedl, 1964g: 315.

= *Cnestus maculatus* Browne, 1983a: 33.

• *Cnestus atkinsoni* Petrov, 2019: 371.

• *Cnestus bicornioides* (Schedl, 1952g: 368) (*Xyleborus*).

• *Cnestus bicornis* (Eggers, 1923b: 194) (*Xyleborus*).

• *Cnestus bimaculatus* (Eggers, 1927b: 88) (*Xyleborus*).

• *Cnestus fijianus* (Schedl, 1938g: 50) (*Xyleborus*).

• *Cnestus gravidus* (Blandford, 1898b: 427) (*Xyleborus*).

• *Cnestus improcerus* (Sampson, 1921: 33) (*Xyleborus*).

• *Cnestus laticeps* (Wood, 1977a: 219) (*Xyleborus*).

• *Cnestus lepidus* (Bright, 1972a: 74) (*Xyleborus*).

• *Cnestus luculentus* Smith, Beaver & Cognato in: Smith et al. 2022: 7.

• *Cnestus magnus* Sampson, 1911: 383.

• *Cnestus mutilatus* (Blandford, 1894c: 103) (*Xyleborus*).

= *Xyleborus sampsoni* Eggers, 1930b: 184.

= *Xyleborus banjoewangi* Schedl, 1939j: 41.

= *Xyleborus taitonus* Eggers, 1939a: 118.

• *Cnestus nitidiloides* (Schedl, 1951i: 89) (*Xyleborus*).

• *Cnestus nitidipennis* (Schedl, 1951i: 88) (*Xyleborus*).

• *Cnestus nitidus* (Schedl, 1951i: 87) (*Xyleborus*).

• *Cnestus nudus* (Nunberg, 1956c: 209) (*Xyleborus*) (RN).

= *Xyleborus puntulatus* Schedl, 1951i: 61.

• *Cnestus orbiculatus* (Schedl, 1942b: 186) (*Xyleborus*).

• *Cnestus peruanus* (Wood, 2007: 468) (*Xylosandrus*).

• *Cnestus protensus* (Eggers, 1930b: 201) (*Xyleborus*).

= *Cnestus rostratus* Schedl, 1977c: 502.

• *Cnestus quadrispinosus* Sittichaya & Beaver, 2018: 32.

• *Cnestus retifer* (Wood, 2007: 468) (*Xylosandrus*).

• *Cnestus retusus* (Eichhoff, 1868b: 151) (*Xyleborus*).

• *Cnestus rotundatus* Schedl, 1975a: 357.

• *Cnestus schoenmanni* Petrov & Mandelshtam, 2018: 269.

• *Cnestus solidus* (Eichhoff, 1868b: 151) (*Xyleborus*).

= *Xyleborus pseudosolidus* Schedl, 1936e: 530.

• *Cnestus suturalis* (Eggers, 1930b: 200) (*Xyleborus*).

• *Cnestus testudo* (Eggers, 1939a: 116) (*Xyleborus*).

• *Cnestus triangularis* (Schedl, 1975a: 370) (*Xyleborus*).

#### *Coptoborus* Hopkins

77 spp.

• *Coptoborus amazonicus* (Petrov, 2020b: 406) (*Theoborus*).

• *Coptoborus amplissimus* Smith & Cognato, 2021: 629.

• *Coptoborus artetenuis* (Schedl, 1973a: 372) (*Xyleborus*).

• *Coptoborus asperatus* Smith & Cognato, 2021: 631.

• *Coptoborus atlanticus* (Bright & Torres, 2006: 417) (*Xyleborus*).

• *Coptoborus attenuatus* Wood, 2007: 400.

• *Coptoborus barbicauda* Smith & Cognato, 2021: 633.

• *Coptoborus bellus* Bright & Torres, 2006: 415.

• *Coptoborus bettysmithae* Smith & Cognato, 2021: 636.

• *Coptoborus brevicauda* Smith & Cognato, 2021: 637.

• *Coptoborus brigman* Smith & Cognato, 2021: 638.

• *Coptoborus busoror* Smith & Cognato, 2021: 640.

• *Coptoborus capillisoror* Smith & Cognato, 2021: 641.

• *Coptoborus carumbensis* Wood, 2007: 399.

• *Coptoborus catulus* (Blandford, 1898a: 215) (*Xyleborus*).

= *Xyleborus intricatus* Schedl, 1949b: 274.

• *Coptoborus chica* Smith & Cognato, 2021: 644.

• *Coptoborus coartatus* (Sampson, 1921: 32) (*Xyleborus*).

= *Xyleborus artecuneolus* Schedl, 1939g: 14.

• *Coptoborus cracens* Wood, 2007: 400.

• *Coptoborus crassisorocula* Smith & Cognato, 2021: 647.

• *Coptoborus crinitulus* (Wood, 1974a: 34) (*Xyleborus*).

• *Coptoborus cuneatus* (Eichhoff, 1878b: 380) (*Xyleborus*).

• *Coptoborus doliolum* Smith & Cognato, 2021: 650.

• *Coptoborus erwini* Smith & Cognato, 2021: 651.

• *Coptoborus exilis* (Schedl, 1934c: 209) (*Xyleborus*).

• *Coptoborus exutus* (Wood, 1974a: 36) (*Xyleborus*).

• *Coptoborus furiosa* Smith & Cognato, 2021: 654.

• *Coptoborus galacatosae* Smith & Cognato, 2021: 656.

• *Coptoborus gentilis* (Schedl, 1972f: 70) (*Xyleborus*).

• *Coptoborus gracilens* Wood, 2007: 401.

• *Coptoborus hansen* Smith & Cognato, 2021: 659.

• *Coptoborus incomptus* Smith & Cognato, 2021: 660.

• *Coptoborus incultus* (Wood, 1975b: 400) (*Xyleborus*).

• *Coptoborus inornatus* Wood, 2007: 399.

• *Coptoborus janeway* Smith & Cognato, 2021: 663.

• *Coptoborus katniss* Smith & Cognato, 2021: 664.

• *Coptoborus leeloo* Smith & Cognato, 2021: 666.

• *Coptoborus leia* Smith & Cognato, 2021: 667.

• *Coptoborus leporinus* Smith & Cognato, 2021: 668.

• *Coptoborus magnus* (Petrov, 2020b: 408) (*Theoborus*).

• *Coptoborus martinezae* Smith & Cognato, 2021: 671.

• *Coptoborus micarius* (Wood, 1974a: 33) (*Xyleborus*).

• *Coptoborus murinus* Smith & Cognato, 2021: 672.

• *Coptoborus newt* Smith & Cognato, 2021: 672.

• *Coptoborus nudulus* Wood, 2007: 394.

• *Coptoborus obtusicornis* (Schedl, 1976: 78) (*Sampsonius*).

• *Coptoborus ochromactonus* Smith & Cognato in: Stillwell et al. 2014: 677.

• *Coptoborus osbornae* Smith & Cognato, 2021: 677.

• *Coptoborus panosus* Smith & Cognato, 2021: 679.

• *Coptoborus papillicauda* Smith & Cognato, 2021: 680.

• *Coptoborus paurus* (Wood, 2007: 388) (*Theoborus*).

• *Coptoborus pilisoror* Smith & Cognato, 2021: 682.

• *Coptoborus pristis* (Wood, 1974a: 31) (*Xyleborus*).

• *Coptoborus pseudotenuis* (Schedl, 1936a: 109) (*Xyleborus*).

= *Xyleborus tenuis* Schedl, 1949b: 269.

• *Coptoborus puertoricensis* (Bright & Torres, 2006: 414) (*Theoborus*).

• *Coptoborus ricini* (Eggers, 1932c: 296) (*Xyleborus*).

= *Xyleborus solitariceps* Schedl, 1954b: 45.

• *Coptoborus ripley* Smith & Cognato, 2021: 687.

• *Coptoborus sagitticauda* Smith & Cognato, 2021: 689.

• *Coptoborus sarahconnor* Smith & Cognato, 2021: 690.

• *Coptoborus schulzi* Wood, 2007: 394.

• *Coptoborus scully* Smith & Cognato, 2021: 692.

• *Coptoborus semicostatus* (Schedl, 1949b: 268) (*Xyleborus*).

• *Coptoborus sicula* Smith & Cognato, 2021: 694.

• *Coptoborus silviasalasi* Atkinson, 2018: 345.

• *Coptoborus solitariformis* (Schedl, 1976: 77) (*Xyleborus*).

• *Coptoborus sororcula* Smith & Cognato, 2021: 696.

• *Coptoborus spicatus* Wood, 2007: 394.

• *Coptoborus starbuck* Smith & Cognato, 2021: 698.

• *Coptoborus subtilis* (Schedl, 1970e: 96) (*Xyleborus*).

• *Coptoborus tolimanus* (Eggers, 1928a: 97) (*Xyleborus*).

• *Coptoborus trinity* Smith & Cognato, 2021: 701.

• *Coptoborus tristiculus* (Wood, 1975b: 401) (*Xyleborus*).

• *Coptoborus uhura* Smith & Cognato, 2021: 703.

• *Coptoborus vasquez* Smith & Cognato, 2021: 705.

• *Coptoborus vespatorius* (Schedl, 1931a: 339) (*Xyleborus*).

= *Coptoborus emarginatus* Hopkins, 1915d: 53.

= *Xyleborus corniculatulus* Schedl, 1949b: 275.

= *Xyleborus corniculatus* Schedl, 1949b: 275.

• *Coptoborus villosulus* (Blandford, 1898a: 204) (*Xyleborus*).

= *Theoborus theobromae* Hopkins, 1915d: 57.

= *Xyleborus pseudococcotrypes* Eggers, 1941a: 105.

= *Xyleborus coccotrypoides* Eggers, 1943d: 388.

= *Xyleborus hirtellus* Schedl, 1949b: 271.

= *Xyleborus villosus* Schedl, 1949b: 270.

• *Coptoborus vrataski* Smith & Cognato, 2021: 709.

• *Coptoborus yar* Smith & Cognato, 2021: 710.

#### *Coptodryas* Hopkins

25 spp.

• *Coptodryas amydra* Smith, Beaver & Cognato, 2020: 157.

• *Coptodryas bella* (Sampson, 1921: 31) (*Xyleborus bellus*).

• *Coptodryas brevior* (Eggers, 1923b: 183) (*Xyleborus*).

• *Coptodryas brunnea* (Browne, 1981b: 601) (*Xyleborus brunneus*).

• *Coptodryas camela* (Eggers, 1940f: 152) (*Xyleborus*).

• *Coptodryas carinata* Smith, Beaver & Cognato, 2020: 159.

• *Coptodryas chimbui* (Schedl, 1973c: 75) (*Xyleborus*).

= *Xyleborus rosseli* Schedl, 1975l: 34 (RN).

• *Coptodryas concinna* (Beeson, 1930: 214) (*Xyleborus concinnus*).

= *Xyleboricus flexiocostatus* Schedl, 1942d: 31.

• *Coptodryas confusa* Hopkins, 1915d: 54.

= *Xyleborus cryphaloides* Schedl, 1942b: 191.

• *Coptodryas costipennis* (Schedl, 1959i: 501) (*Xyleborus*).

• *Coptodryas cuneolus* (Eggers, 1927b: 92) (*Xyleborus*).

• *Coptodryas curvidentis* (Schedl, 1958e: 104) (*Xyleborus*).

• *Coptodryas decepta* (Schedl, 1979e: 103) (*Arixyleborus deceptus*).

• *Coptodryas destricta* (Schedl, 1939b: 352) (*Xyleborus destrictus*).

• *Coptodryas elegans* (Sampson, 1923: 288) (*Xyleborus*).

• *Coptodryas erinacea* (Eggers, 1927b: 103) (*Xyleborus erinaceus*).

• *Coptodryas inornata* Smith, Beaver & Cognato, 2020: 162.

• *Coptodryas muasi* (Browne, 1962b: 306) (*Xyleborus*).

• *Coptodryas mus* (Eggers, 1930b: 203) (*Xyleborus*).

• *Coptodryas nudipennis* (Schedl, 1951i: 63) (*Xyleborus*).

• *Coptodryas obtusicollis* (Schedl, 1938e: 427) (*Xyleborus*).

• *Coptodryas pseudopunctula* (Schedl, 1942b: 194) (*Xyleborus pseudopunctulus*).

• *Coptodryas pubifer* (Schedl, 1972d: 200) (*Xyleborus*).

• *Coptodryas punctipennis* (Schedl, 1953f: 302) (*Xyleborus*).

• *Coptodryas quadricostata* (Schedl, 1942d: 30) (*Xyleborus*).

#### *Cryptoxyleborus* Wood & Bright

18 spp.

• *Cryptoxyleborus acutus* (Schedl, 1975a: 361) (*Xyleborus*).

• *Cryptoxyleborus barbieri* (Schedl, 1953d: 128) (Cryptoxyleborus*).

• *Cryptoxyleborus brevicauda* Sittichaya & Beaver, 2024: 398.

• *Cryptoxyleborus confusus* (Browne, 1950: 644) (Cryptoxyleborus*).

• *Cryptoxyleborus cuneatus* Beaver & Hulcr, 2008: 138.

• *Cryptoxyleborus eggersi* (Schedl, 1936d: 60) (Cryptoxyleborus*).

= *Cryptoxyleborus dryobalanopsis* Schedl, 1942b: 184.

= *Xyleborus eggersianus* Schedl, 1960: 110 (RN).

• *Cryptoxyleborus major* (Browne, 1986b: 665) (Cryptoxyleborus*).

• *Cryptoxyleborus mawdsleyi* Beaver & Hulcr, 2008: 140.

• *Cryptoxyleborus micrographus* (Schedl, 1942b: 185) (Cryptoxyleborus*).

• *Cryptoxyleborus naevus* (Schedl, 1937f: 551) (Cryptoxyleborus*).

• *Cryptoxyleborus opacicaudulus* (Schedl, 1942b: 185) (Cryptoxyleborus*).

• *Cryptoxyleborus oxyurus* (Schedl, 1942b: 184) (Cryptoxyleborus*).

= *Cryptoxyleborus shoreae* Browne, 1981b: 602.

• *Cryptoxyleborus percuneolus* (Schedl, 1951i: 85) (*Xyleborus*).

• *Cryptoxyleborus quadriporus* (Beaver, 1990a: 281) (Cryptoxyleborus*).

• *Cryptoxyleborus stenographus* (Schedl, 1971e: 383) (*Xyleborus*).

• *Cryptoxyleborus subnaevus* (Schedl, 1937f: 552) (Cryptoxyleborus*).

• *Cryptoxyleborus turbineus* (Sampson, 1923: 288) (*Xyleborus*).

• *Cryptoxyleborus vestigator* (Schedl, 1973d: 93) (*Xyleborus*).

= *Platypus goilalae* Schedl, 1975a: 387.

#### *Cyclorhipidion* Hagedorn

109 spp.

• *Cyclorhipidion achlys* Smith, Beaver, Pham & Cognato, 2022: 8.

• *Cyclorhipidion agnaticeps* (Schedl, 1957b: 100) (*Xyleborus*).

• *Cyclorhipidion amanicum* (Hagedorn, 1910: 11) (*Xyleborus amanicus*).

= *Xyleborus tanganjikaensis* Schedl, 1937e: 403.

= *Xyleborus jongaensis* Schedl, 1941c: 404.

= *Xyleborus amanicus ominosus* Schedl, 1963g: 301.

• *Cyclorhipidion amarantum* (Schedl, 1942b: 198) (*Xyleborus amarantus*).

• *Cyclorhipidion amasoides* Smith, Beaver & Cognato, 2020: 177.

• *Cyclorhipidion amputatum* Smith, Beaver & Cognato, 2020: 178.

• *Cyclorhipidion andriani* (Schedl, 1965f: 73) (*Xyleborus*).

• *Cyclorhipidion apicipenne* (Schedl, 1974a: 462) (*Xyleborus*).

= *Xyleborus anoplum* Schedl, 1975a: 362.

• *Cyclorhipidion armiger* (Schedl, 1953a: 28) (*Xyleborus*).

• *Cyclorhipidion artedilatatum* Hulcr & Cognato, 2013: 66.

• *Cyclorhipidion artifex* (Schedl, 1942d: 45) (*Xyleborus*).

• *Cyclorhipidion beaveri* Lin & Smith, 2022: 286.

• *Cyclorhipidion bituberculatum* (Eggers, 1923b: 183) (*Xyleborus bituberculatus*).

= *Xyleborus flavopilosus* Schedl, 1936e: 533.

= *Xyleborus revocabile* Schedl, 1942c: 186.

= *Xyleborus abbreviatipennis* Schedl, 1973d: 88.

= *Xyleborus flavipennis* Schedl, 1979e: 107.

= *Xyleborus funestus* Schedl, 1979e: 107.

= *Xyleborus canarii* Browne, 1984a: 155.

• *Cyclorhipidion bodoanum* (Reitter, 1913: 82) (*Xyleborus bodoanus*).

= *Xyleborus punctulatus* Kurentsov, 1948: 52.

= *Xyleborus takinoyensis* Murayama, 1953a: 109.

= *Xyleborus misatoensis* Nobuchi, 1981c: 143.

• *Cyclorhipidion brownei* Smith, Beaver & Cognato in: Smith et al. 2025: 53.

• *Cyclorhipidion cachani* (Schedl, 1958c: 241) (*Xyleborus*).

• *Cyclorhipidion californicum* (Wood, 1975b: 399) (*Xyleborus californicus*).

• *Cyclorhipidion callosum* (Schedl, 1941c: 405) (*Xyleborus callosus*).

• *Cyclorhipidion capense* (Eggers, 1944c: 98) (*Xyleborus capensis*).

• *Cyclorhipidion circumcisum* (Sampson, 1921: 30) (*Xyleborus circumcisus*).

= *Xyleborus obtusus* Eggers, 1923b: 172.

= *Xyleborus subobtusus* Schedl, 1942b: 192.

• *Cyclorhipidion cognatoi* Lin & Smith, 2022: 288.

• *Cyclorhipidion conidentatum* Smith, Beaver & Cognato in: Smith et al. 2022: 9 (E).

• *Cyclorhipidion corrugatum* (Schedl, 1951i: 93) (*Xyleborus corrugatus*).

• *Cyclorhipidion crucifer* (Hagedorn, 1908: 381) (*Xyleborus*).

= *Xyleborus crucifer cruciferinus* Schedl, 1957b: 98.

• *Cyclorhipidion cruciforme* (Schedl, 1957b: 99) (*Xyleborus cruciformis*).

• *Cyclorhipidion crucipenne* (Schedl, 1963g: 277) (*Xyleborus crucipennis*).

= *Xyleborus metacrucifer* Browne, 1965: 201.

• *Cyclorhipidion curvipenne* (Schedl, 1951i: 82) (*Xyleborus curvipennis*).

• *Cyclorhipidion daosi* (Schedl, 1958c: 242) (*Xyleborus*).

• *Cyclorhipidion denticauda* Smith, Beaver & Cognato, 2020: 183.

• *Cyclorhipidion distinguendum* (Eggers, 1930b: 205) (*Xyleborus distinguendus*).

= *Xyleborus fukiensis* Eggers, 1941c: 225.

= *Xyleborus ganshoensis* Murayama, 1952: 16.

• *Cyclorhipidion druk* Smith & Beaver in: Beaver and Smith 2022: 3.

• *Cyclorhipidion foersteri* (Hagedorn, 1910: 7) (*Xyleborus*).

• *Cyclorhipidion fouqueti* (Schedl, 1937g: 15) (*Xyleborus*).

• *Cyclorhipidion gladigerum* Smith, Beaver & Cognato in: Smith et al. 2022: 10.

• *Cyclorhipidion guineense* (Eggers, 1941b: 179) (*Xyleborus*).

• *Cyclorhipidion hastatum* (Schedl, 1942d: 39) (*Xyleborus hastatus*).

• *Cyclorhipidion hsuae* Lin & Smith, 2022: 289.

• *Cyclorhipidion impar* (Eggers, 1927b: 89) (*Xyleborus*).

• *Cyclorhipidion inaequale* (Schedl, 1934a: 87) (*Xyleborus inaequalis*).

• *Cyclorhipidion inarmatum* (Eggers, 1923b: 209) (*Xyleborus inarmatus*).

= *Xyleborus vagans* Schedl, 1977c: 504.

• *Cyclorhipidion japonicum* (Nobuchi, 1981c: 150) (*Xyleborus japonicus*).

• *Cyclorhipidion kajangense* (Schedl, 1942b: 193) (*Xyleborus kajangensis*).

• *Cyclorhipidion kalshoveni* (Schedl, 1934a: 85) (*Notoxyleborus*).

• *Cyclorhipidion kelantanum* (Schedl, 1953f: 302) (*Xyleborus kelantanus*).

• *Cyclorhipidion kumanoense* (Nobuchi, 1985: 25) (*Xyleborus kumanoensis*) (RN).

= *Xyleborus bispinus* Nobuchi, 1981c: 147.

• *Cyclorhipidion laciniosum* Park & Smith in: Park et al. 2020: 214.

• *Cyclorhipidion laetum* (Niisima, 1909: 159) (*Xyleborus laetus*).

• *Cyclorhipidion lapilliferum* Smith, Beaver, Pham & Cognato, 2022: 11.

• *Cyclorhipidion malayense* (Browne, 1981a: 132) (*Xyleborus malayensis*).

• *Cyclorhipidion miyazakiense* (Murayama, 1936: 144) (*Xyleborus miyazakiensis*).

= *Xyleborus armipennis* Schedl, 1953a: 27.

= *Xyleborus wakayamensis* Nobuchi, 1981c: 144.

• *Cyclorhipidion multigranosum* (Schedl, 1964b: 622) (*Xyleborus multigranosus*).

• *Cyclorhipidion multipunctatum* (Browne, 1980b: 386) (*Xyleborus multipunctatus*).

= *Xyleborus multipunctulus* Browne, 1984e: 98.

• *Cyclorhipidion muticum* Smith, Beaver & Cognato, 2020: 191.

• *Cyclorhipidion nemesis* Smith & Cognato, 2022: 9.

• *Cyclorhipidion neocavipenne* (Schedl, 1977c: 503) (*Xyleborus neocavipennis*).

• *Cyclorhipidion neocrucifer* (Schedl, 1955b: 219) (*Xyleborus*).

• *Cyclorhipidion nepalense* Smith, Beaver & Cognato in: Smith et al. 2022: 12.

• *Cyclorhipidion nodosum* Beaver, 2005a: 113.

• *Cyclorhipidion nsafukalae* (Schedl, 1957b: 105) (*Xyleborus*).

• *Cyclorhipidion obesulum* Smith, Beaver & Cognato, 2020: 192.

• *Cyclorhipidion obiense* (Browne, 1980a: 375) (*Xyleborus obiensis*).

• *Cyclorhipidion obliquesectum* (Eggers, 1927b: 99) (*Xyleborus obliquesectus*).

• *Cyclorhipidion obtusatum* (Schedl, 1972d: 200) (*Xyleborus obtusatus*).

• *Cyclorhipidion ohnoi* (Browne, 1980a: 375) (*Xyleborus*).

• *Cyclorhipidion opimatum* (Schedl, 1975g: 459) (*Xyleborus opimatus*).

• *Cyclorhipidion pelliculosum* (Eichhoff, 1878b: 336) (*Xyleborus pelliculosus*).

= *Xyleborus seiryorense* Murayama, 1930: 21.

= *Xyleborus quercus* Kurentsov, 1948: 51.

= *Xyleborus starki* Nunberg, 1956c: 209 (RN).

= *Xyleborus okinosenensis* Murayama, 1961: 31.

• *Cyclorhipidion perlaetum* (Schedl, 1960: 107) (*Xyleborus perlaetus*) (RN).

= *Cyclorhipidion pelliculosum* Hagedorn, 1912a: 356.

• *Cyclorhipidion perpilosellum* (Schedl, 1935d: 402) (*Xyleborus perpilosellus*).

= *Xyleborus punctatopilosus* Schedl, 1936e: 532.

• *Cyclorhipidion perpunctatum* (Schedl, 1971e: 382) (*Xyleborus perpunctatus*).

• *Cyclorhipidion petrosum* Smith, Beaver & Cognato, 2020: 196.

• *Cyclorhipidion pilipenne* (Eggers, 1940f: 140) (*Xyleborus pilipennis*).

• *Cyclorhipidion pilosulum* (Eggers, 1927b: 100) (*Xyleborus pilosulus*).

• *Cyclorhipidion planotruncatum* (Schedl, 1942b: 197) (*Xyleborus planotruncatus*).

• *Cyclorhipidion posticespinatum* (Eggers, 1940e: 238) (*Xyleborus posticespinatus*).

• *Cyclorhipidion praecursor* (Schedl, 1963g: 527) (*Xyleborus*).

• *Cyclorhipidion pruinosum* (Blandford, 1896e: 214) (*Xyleborus pruinosus*).

= *Xyleborus arcticollis* Blandford, 1896e: 217.

= *Xyleborus foersteri decipiens* Eggers, 1923b: 182.

• *Cyclorhipidion psaltes* (Schedl, 1954d: 81) (*Xyleborus*).

• *Cyclorhipidion pseudofoersteri* (Schedl, 1942b: 191) (*Xyleborus*).

• *Cyclorhipidion punctatum* (Eggers, 1923b: 182) (*Xyleborus punctatus*).

• *Cyclorhipidion punctilicolle* (Schedl, 1942b: 191) (*Xyleborus punctilicollis*).

• *Cyclorhipidion quadricuspe* (Schedl, 1942d: 34) (*Xyleborus quadricuspis*).

• *Cyclorhipidion quasimodo* (Browne, 1980e: 776) (*Xyleborus*).

• *Cyclorhipidion repandum* (Schedl, 1942c: 186) (*Xyleborus repandus*).

• *Cyclorhipidion repositum* (Schedl, 1942b: 197) (*Xyleborus repositus*).

= *Xyleborus pruinosulus* Browne in: Beaver and Browne 1979: 611.

• *Cyclorhipidion scalptor* (Schedl, 1961g: 153) (*Xyleborus*).

• *Cyclorhipidion scorpium* (Schedl, 1942b: 187) (*Xyleborus scorpius*).

• *Cyclorhipidion separandum* (Schedl, 1971e: 383) (*Xyleborus separandus*).

• *Cyclorhipidion sisyrnophorum* (Hagedorn, 1910: 7) (*Xyleborus sisyrnophorus*).

• *Cyclorhipidion spinibarbe* (Schedl, 1955b: 219) (*Xyleborus spinibarbis*).

• *Cyclorhipidion spinidens* (Eggers, 1920b: 116) (*Xyleborus*).

• *Cyclorhipidion spurlinum* Hulcr & Cognato, 2013: 69.

• *Cyclorhipidion squamulatum* (Beaver in: Beaver and Löyttyniemi 1985: 69) (*Apoxyleborus squamulatus*).

• *Cyclorhipidion subpruinosum* (Browne, 1986a: 93) (*Xyleborus subpruinosus*).

• *Cyclorhipidion sulcipenne* (Eggers, 1932c: 299) (*Xyleborus sulcipennis*).

• *Cyclorhipidion superbum* (Schedl, 1942c: 188) (*Xyleborus superbus*).

• *Cyclorhipidion sus* (Schedl, 1973d: 93) (*Xyleborus*).

= *Xyleborus varicus* Schedl, 1975a: 360.

• *Cyclorhipidion taedulum* Smith, Beaver, Pham & Cognato, 2022: 13.

• *Cyclorhipidion tanibe* (Schedl, 1965f: 77) (*Xyleborus tanibis*).

• *Cyclorhipidion tecleae* (Schedl, 1958a: 883) (*Xyleborus*).

• *Cyclorhipidion tenuigraphum* (Schedl, 1953a: 29) (*Xyleborus tenuigraphus*).

• *Cyclorhipidion titorum* Smith, Beaver, Pham & Cognato, 2022: 14.

• *Cyclorhipidion triste* Park & Smith in: Park et al. 2020: 215.

• *Cyclorhipidion truncaudinum* Smith, Beaver & Cognato, 2020: 202.

• *Cyclorhipidion tuberculosissimum* (Eggers, 1940f: 152) (*Xyleborus tuberculosissimus*).

• *Cyclorhipidion umbratum* (Eggers, 1941c: 223) (*Xyleborus umbratus*).

• *Cyclorhipidion vigilans* (Schedl, 1939j: 43) (*Xyleborus*).

• *Cyclorhipidion wui* Lin & Smith, 2022: 291.

• *Cyclorhipidion xeniolum* Smith, Beaver & Cognato, 2020: 205.

• *Cyclorhipidion xyloteroides* (Eggers, 1939a: 120) (*Xyleborus*).

#### *Debus* Hulcr & Cognato

34 spp.

• *Debus abscissus* (Browne, 1974b: 537) (*Xyleborus*).

• *Debus adusticollis* (Motschulsky, 1863: 514) (*Tomicus*).

= *Xyleborus vestitus* Schedl, 1931a: 341.

• *Debus amphicranoides* (Hagedorn, 1908: 379) (*Xyleborus*).

= *Xyleborus amphicranoides latecavatus* Eggers, 1927b: 95.

= *Xyleborus amphicranoides parvior* Browne, 1981b: 601.

• *Debus amplexicauda* (Hagedorn, 1910: 9) (*Xyleborus*).

= *Xyleborus borneensis* Eggers, 1927b: 97.

• *Debus armillatus* (Schedl, 1933c: 199) (*Xyleborus*).

• *Debus balbalanus* (Eggers, 1927b: 95) (*Xyleborus*).

• *Debus birmanus* (Eggers, 1930b: 200) (*Xyleborus*).

• *Debus blandus* (Schedl, 1954e: 160) (*Xyleborus*).

• *Debus cavatus* (Browne, 1980c: 487) (*Xyleborus*).

• *Debus cavulus* (Browne, 1974b: 538) (*Xyleborus*).

= *Xyleborus cavuloides* Browne, 1984c: 451.

• *Debus cyclopus* (Schedl, 1940b: 440) (*Xyleborus*).

• *Debus cylindromorphus* (Eggers, 1927b: 96) (*Xyleborus*).

• *Debus defensus* (Blandford, 1894c: 118) (*Xyleborus*).

• *Debus dentatus* (Blandford, 1895: 323) (*Xyleborus*).

• *Debus detritus* (Eggers, 1927e: 402) (*Xyleborus*).

= *Xyleborus maniensis* Browne, 1981a: 130.

• *Debus dolosus* (Blandford, 1896e: 225) (*Xyleborus*).

• *Debus emarginatus* (Eichhoff, 1878a: 392) (*Xyleborus*).

= *Xyleborus emarginatus* Eichhoff, 1878b: 510.

= *Xyleborus exesus* Blandford, 1894c: 119.

= *Tomicus cinchonae* Veen, 1897: 135.

= *Xyleborus cordatus* Hagedorn, 1910: 12.

= *Coptoborus palmeri* Hopkins, 1915d: 54.

= *Coptoborus terminaliae* Hopkins, 1915d: 54.

= *Xyleborus emarginatus semicircularis* Schedl, 1973d: 92.

• *Debus excavus* (Schedl, 1964i: 249) (*Xyleborus*).

• *Debus eximius* (Schedl, 1970f: 362) (*Xyleborus*).

= *Xyleborus apicenotatus* Schedl, 1971e: 377.

• *Debus fallaxoides* (Schedl, 1955e: 302) (*Xyleborus*).

• *Debus fischeri* (Hagedorn, 1908: 380) (*Xyleborus*).

• *Debus hatanakai* (Browne, 1983b: 559) (*Xyleborus*).

• *Debus insitivus* (Schedl, 1959i: 509) (*Xyleborus*).

• *Debus latecornis* (Schedl, 1969a: 212) (*Xyleborus*).

= *Xyleborus opulentus* Schedl, 1975a: 369.

• *Debus persimilis* (Eggers, 1927b: 97) (*Xyleborus*).

= *Xyleborus subdolosus* Schedl, 1942d: 44.

= *Xyleborus balanocarpi* Nunberg, 1961a: 619.

• *Debus pseudocylindricus* (Eggers, 1927e: 402) (*Xyleborus*).

• *Debus pumilus* (Eggers, 1923b: 209) (*Xyleborus*).

= *Xyleborus cylindricus* Eggers, 1927b: 94.

= *Xyleborus neocylindricus* Schedl, 1942b: 196.

= *Ips kelantanensis* Browne, 1955: 345.

= *Xyleborus ipidia* Schedl, 1972k: 63.

= *Xyleborus planodeclivis* Browne, 1974a: 70.

• *Debus quadrispinus* (Motschulsky, 1863: 514) (*Tomicus*).

= *Xyleborus fallax* Eichhoff, 1878a: 392.

= *Xyleborus fallax* Eichhoff, 1878b: 508.

= *Xyleborus fallax* Stebbing, 1914: 582.

= *Xyleborus amphicranulus* Eggers, 1923b: 204.

= *Xyleborus fastigatus* Schedl, 1935d: 402.

• *Debus robustipennis* (Schedl, 1954e: 159) (*Xyleborus*).

= *Xyleborus interponens* Schedl, 1954e: 160.

• *Debus shoreae* (Stebbing, 1909: 28) (*Tomicus*).

= *Tomicus assamensis* Stebbing, 1909: 29.

• *Debus spinatus* (Eggers, 1923b: 203) (*Xyleborus*).

• *Debus spinicornis* (Schedl, 1975a: 370) (*Xyleborus*).

• *Debus subdentatus* (Browne, 1986a: 97) (*Xyleborus*).

= *Xyleborus katoi* Browne, 1986b: 666.

• *Debus trispinatus* (Browne, 1981a: 130) (*Xyleborus*).

#### *Dinoxyleborus* Smith

3 spp.

• *Dinoxyleborus cognatoi* Smith, 2017: 132.

• *Dinoxyleborus infernus* Smith, 2017: 135.

• *Dinoxyleborus sexnotatus* (Schedl, 1970e: 95) (*Xyleborus*).

#### *Diuncus* Hulcr & Cognato

20 spp.

• *Diuncus adossuarius* (Schedl, 1952g: 367) (*Xyleborus*).

• *Diuncus ciliatoformis* (Schedl, 1953b: 81) (*Xyleborus*).

• *Diuncus clerodendronae* (Schedl, 1952b: 17) (*Xyleborus*).

• *Diuncus conidens* (Eggers, 1936a: 632) (*Xyleborus*).

• *Diuncus corpulentus* (Eggers, 1930b: 198) (*Xyleborus*).

• *Diuncus dossuarius* (Eggers, 1923b: 187) (*Xyleborus*).

• *Diuncus duodecimspinatus* (Schedl, 1936e: 531) (*Xyleborus*).

• *Diuncus gorggae* (Schedl, 1973d: 91) (*Xyleborus*).

• *Diuncus haberkorni* (Eggers, 1920c: 43) (*Xyleborus*).

= *Xyleborus approximatus* Schedl, 1951i: 77.

= *Xyleborus taichuensis* Schedl, 1952h: 64.

= *Xyleborus potens* Schedl, 1964k: 298.

• *Diuncus haddeni* (Schedl, 1933b: 101) (*Xyleborus*).

• *Diuncus javanus* (Eggers, 1923b: 188) (*Xyleborus*).

= *Xyleborus perdix* Schedl, 1939b: 351.

• *Diuncus justus* (Schedl, 1931a: 339) (*Xyleborus*).

= *Xyleborus marginicollis* Schedl, 1936d: 64.

= *Xyleborus ciliatus* Eggers, 1940f: 141.

= *Xyleborus apiculatus* Schedl, 1942b: 190.

• *Diuncus mesoleiulus* (Schedl, 1979e: 108) (*Xyleborus*).

= *Xyleborus brevicollis* Browne, 1984e: 97.

• *Diuncus mollis* Beaver & Liu, 2016: 22.

• *Diuncus mucronatulus* (Eggers, 1930b: 199) (*Xyleborus*).

• *Diuncus mucronatus* (Eggers, 1923b: 191) (*Xyleborus*).

• *Diuncus niger* Hulcr & Cognato, 2013: 82.

• *Diuncus papatrae* (Schedl, 1972j: 53) (*Xyleborus*).

= *Xyleborus mucronatoides* Schedl, 1975a: 368.

= *Xyleborus biuncus* Browne, 1984e: 96.

• *Diuncus quadrispinosulus* (Eggers, 1923b: 189) (*Xyleborus*).

= *Xyleborus parvispinosus palembangensis* Schedl, 1939j: 43.

= *Xyleborus parvispinosus* Schedl, 1951i: 78.

• *Diuncus taxicornis* (Schedl, 1971e: 385) (*Xyleborus*).

#### *Dryocoetoides* Hopkins

22 spp.

• *Dryocoetoides alter* (Eggers, 1931a: 22) (*Xyleborus*).

• *Dryocoetoides asperulus* (Eggers, 1931a: 21) (*Xyleborus*).

= *Xyleborus imitator* Schedl, 1976: 75.

• *Dryocoetoides capucinus* (Eichhoff, 1869: 281) (*Xyleborus*).

= *Xyleborus rufithorax* Eichhoff, 1869: 281.

= *Dryocoetoides guatemalensis* Hopkins, 1915d: 52.

= *Xyleborus capucinoides* Eggers, 1941a: 104.

• *Dryocoetoides cristatus* (Fabricius, 1801: 389) (*Bostrichus*).

= *Xyleborus solitarius* Hagedorn, 1905b: 415.

= *Xyleborus urichi* Sampson, 1912: 245.

= *Dryocoetoides caracicolai* Hopkins, 1915d: 53.

= *Xyleborus crenatus* Eggers, 1920c: 42.

• *Dryocoetoides flavus* (Fabricius, 1801: 394) (*Hylesinus*).

• *Dryocoetoides granulicauda* (Eggers, 1931a: 22) (*Xyleborus*).

= *Xyleborus rufithorax nigricollis* Hagedorn, 1905b: 413.

• *Dryocoetoides inaffectatus* (Schedl, 1972f: 71) (*Xyleborus*).

• *Dryocoetoides indolatus* Wood, 1974a: 31.

• *Dryocoetoides insculptus* Wood, 1974a: 30.

• *Dryocoetoides obtusitruncatus* (Schedl, 1949b: 271) (*Xyleborus*).

• *Dryocoetoides paradoxus* (Schedl, 1972f: 71) (*Xyleborus*).

= *Xyleborus solitaripennis* Schedl, 1976: 77.

• *Dryocoetoides pileatus* Wood, 1974a: 29.

• *Dryocoetoides pseudosolitarius* (Eggers, 1933f: 28) (*Xyleborus*).

= *Xyleborus pseudosolitarius schizolobus* Schedl, 1950c: 179.

• *Dryocoetoides rusticus* Wood, 1974a: 30.

= *Xyleborus haesitus* Schedl, 1976: 74.

• *Dryocoetoides severus* Wood, 1974a: 30.

• *Dryocoetoides solitarinus* (Schedl, 1950c: 178) (*Xyleborus*).

• *Dryocoetoides truncatellus* (Schedl, 1949b: 272) (*Xyleborus*).

• *Dryocoetoides tuberculatus* Pérez & Atkinson in: Pérez et al. 2020: 447.

• *Dryocoetoides velutinus* Wood, 1974a: 29.

• *Dryocoetoides verrucosus* Wood, 1974a: 28.

• *Dryocoetoides versutus* Wood, 2007: 379.

• *Dryocoetoides vexans* (Schedl, 1972f: 72) (*Xyleborus*).

= *Dryocoetoides reticulatus* Atkinson, 2009: 66.

#### *Dryoxylon* Bright & Rabaglia

1 sp.

• *Dryoxylon onoharaense* (Murayama, 1934a: 293) (*Xyleborus onoharaensis*).

#### *Eccoptopterus* Motschulsky

6 spp.

• *Eccoptopterus drescheri* Eggers, 1940f: 154.

• *Eccoptopterus formosanus* Lin, Sittichaya & Smith in: Sittichaya et al. 2024b: 253.

• *Eccoptopterus intermedius* Sittichaya, Lin & Smith in: Sittichaya et al. 2024b: 256.

• *Eccoptopterus limbus* Sampson, 1911: 381.

= *Xyleborus squamulosus* Eggers, 1923b: 193.

= *Xyleborus squamulosus auratus* Eggers, 1923b: 193.

= *Xyleborus squamulosus duplicatus* Eggers, 1923b: 193.

• *Eccoptopterus spinosus* (Olivier, 1800a: 9) (*Scolytus*).

= *Eccoptopterus sexspinosus* Motschulsky, 1863: 515.

= *Xyleborus abnormis* Eichhoff, 1869: 282.

= *Platydactylus gracilipes* Eichhoff, 1886: 25.

= *Xyleborus sexspinosus multispinosus* Hagedorn, 1908: 377.

= *Xyleborus collaris* Eggers, 1923b: 194.

= *Eccoptopterus sagittarius* Schedl, 1939j: 41.

= *Xyleborus sexspinosus pluridentatus* Schedl, 1942d: 49.

= *Xyleborus eccoptopterus* Schedl, 1951f: 154.

• *Eccoptopterus tarsalis* Schedl, 1936c: 26.

#### *Eggersanthus* Sittichaya & Smith

1 sp.

• *Eggersanthus sublaevis* (Eggers, 1927b: 104) (*Webbia*).

#### *Euwallacea* Hopkins

87 spp.

• *Euwallacea agathis* (Browne, 1984b: 291) (*Xyleborus*).

• *Euwallacea alastos* Smith, Beaver & Cognato in: Smith et al. 2022: 16.

• *Euwallacea andamanensis* (Blandford, 1896e: 222) (*Xyleborus*).

= *Xyleborus noxius* Sampson, 1913: 445.

= *Xyleborus siobanus* Eggers, 1923b: 186.

= *Xyleborus burmanicus* Beeson, 1930: 210.

= *Xyleborus granulipennis* Eggers, 1930b: 194.

= *Xyleborus intextus* Beeson, 1930: 211.

= *Xyleborus senchalensis* Beeson, 1930: 212.

= *Xyleborus talumalai* Browne, 1966: 248.

• *Euwallacea andreae* Hulcr & Cognato, 2013: 91.

• *Euwallacea aplanatus* (Wichmann, 1914b: 412) (*Xyleborus*).

• *Euwallacea assimilis* (Eggers, 1927e: 405) (*Xyleborus*).

• *Euwallacea barbatus* (Hagedorn, 1910: 11) (*Xyleborus*).

• *Euwallacea beckeri* (Bright, 1972a: 84) (*Xyleborus*).

• *Euwallacea benguetensis* (Schedl, 1951i: 71) (*Xyleborus*).

• *Euwallacea bryanti* (Sampson, 1919: 110) (*Xyleborus*).

• *Euwallacea caraibicus* (Eggers, 1941a: 103) (*Xyleborus*).

= *Xyleborus variabilis* Schedl, 1949b: 281.

= *Xyleborus trinidadensis* Schedl, 1961b: 530.

• *Euwallacea chiangdao* Sittichaya, Smith & Beaver in: Sittichaya et al. 2024c: 398.

• *Euwallacea congruens* Sittichaya, Smith & Beaver in: Sittichaya et al. 2024c: 400.

• *Euwallacea declivispinatus* (Schedl, 1970h: 216) (*Xyleborus*).

= *Xyleborus tectus* Schedl, 1972k: 63.

• *Euwallacea densatus* (Schedl, 1979e: 106) (*Xyleborus*).

• *Euwallacea deplanatulus* (Schedl, 1950e: 53) (*Xyleborus*).

= *Xyleborus duplex* Browne, 1974a: 68.

• *Euwallacea destruens* (Blandford, 1896e: 221) (*Xyleborus*).

= *Xyleborus barbatulus* Schedl, 1934a: 86.

= *Xyleborus procerior* Schedl, 1942c: 189.

= *Xyleborus nandarivatus* Schedl, 1950e: 52.

= *Xyleborus procerrimus* Schedl, 1969a: 214.

• *Euwallacea dilatatiformis* (Schedl, 1971b: 285) (*Xyleborus*).

• *Euwallacea discretus* (Eggers, 1933f: 29) (*Xyleborus*).

= *Xyleborus usticus* Wood, 1968a: 3.

• *Euwallacea elevatus* (Eggers, 1931a: 21) (*Xyleborus*).

• *Euwallacea fornicatior* (Eggers, 1923b: 184) (*Xyleborus*).

= *Xyleborus schultzei* Schedl, 1951i: 68.

• *Euwallacea fornicatus* (Eichhoff, 1868b: 151) (*Xyleborus*).

= *Xyleborus whitfordiodendrus* Schedl, 1942b: 189.

= *Xyleborus tapatapaoensis* Schedl, 1951f: 152.

• *Euwallacea frimensis* Smith, Beaver & Cognato in: Smith et al. 2025: 65.

• *Euwallacea fulgidus* Bright, 2019: 266.

• *Euwallacea fulvus* (Murayama, 1936: 142) (*Xyleborus*).

• *Euwallacea funereus* (Lea, 1910: 139) (*Xyleborus*).

= *Xyleborus nepos* Eggers, 1923b: 199.

= *Xyleborus nepos robustus* Schedl, 1933c: 199.

= *Xyleborus signatus* Schedl, 1949b: 278.

• *Euwallacea galeatus* (Blandford, 1894c: 123) (*Xyleborus*).

• *Euwallacea galoanus* Browne, 1974a: 69 (*Xyleborus*).

• *Euwallacea geminus* Smith, Beaver & Cognato, 2020: 239.

• *Euwallacea granosus* (Schedl, 1957b: 101) (*Xyleborus*).

• *Euwallacea gravelyi* (Wichmann, 1914b: 411) (*Xyleborus*).

= *Xyleborus ovalicollis* Eggers, 1930b: 193.

• *Euwallacea illustrior* (Schedl, 1939j: 51) (*Xyleborus illustrius*).

• *Euwallacea innovatus* Bright, 2019: 267.

• *Euwallacea insolitus* Smith & Beaver in: Beaver and Smith 2022: 5.

• *Euwallacea interjectus* (Blandford, 1894d: 576) (*Xyleborus*).

= *Xyleborus pseudovalidus* Eggers, 1925: 159.

• *Euwallacea jamaicensis* (Bright, 1972a: 79) (*Xyleborus*).

• *Euwallacea kersianus* (Browne, 1981a: 132) (*Xyleborus*).

• *Euwallacea khayae* (Schedl, 1958a: 880) (*Xyleborus*).

• *Euwallacea kuroshio* Gomez & Hulcr in: Gomez et al. 2018: 9.

• *Euwallacea latecarinatus* (Schedl, 1936b: 10) (*Xyleborus*).

• *Euwallacea luctuosus* (Eggers, 1939b: 13) (*Xyleborus*).

• *Euwallacea lugubris* (Eggers, 1927b: 98) (*Xyleborus*).

• *Euwallacea malloti* (Eggers, 1930b: 192) (*Xyleborus*).

• *Euwallacea minutus* (Blandford, 1894c: 116) (*Xyleborus*).

= *Xyleborus breviusculus* Schedl, 1942b: 196.

= *Xyleborus pernitidus* Schedl, 1954c: 152.

• *Euwallacea neptis* Smith, Beaver & Cognato, 2020: 247.

• *Euwallacea nigrosetosus* (Schedl, 1939j: 49) (*Xyleborus*).

= *Xyleborus nigripennis* Schedl, 1951i: 68.

• *Euwallacea obliquecauda* (Motschulsky, 1863: 513) (*Phloeotrogus*).

= *Xyleborus carinipennis* Eichhoff, 1868b: 152.

• *Euwallacea oparunus* (Beeson, 1940: 201) (*Xyleborus*).

• *Euwallacea pandae* (Schedl, 1957b: 86) (*Xyleborus*).

• *Euwallacea perakensis* (Schedl, 1942b: 194) (*Xyleborus*).

• *Euwallacea perbrevis* (Schedl, 1951i: 59) (*Xyleborus*).

= *Xyleborus molestulus* Wood, 1975b: 400.

• *Euwallacea phrixosoma* Smith, Beaver & Cognato in: Smith et al. 2025: 68.

• *Euwallacea piceus* (Motschulsky, 1863: 512) (*Anodius*).

= *Xyleborus indicus* Eichhoff, 1878b: 354.

= *Xyleborus indicus* Eichhoff, 1878a: 392.

= *Xyleborus imitans* Eggers, 1927e: 404.

= *Xyleborus indicus subcoriaceus* Eggers, 1927b: 92.

= *Xyleborus samoensis* Beeson, 1929: 237.

• *Euwallacea planipennis* (Schedl, 1955e: 305) (*Xyleborus*).

• *Euwallacea plenilunium* Sittichaya, Smith & Beaver in: Sittichaya et al. 2024c: 401.

• *Euwallacea posticus* (Eichhoff, 1869: 281) (*Xyleborus*).

= *Xyleborus posticoides* Schedl, 1949b: 281.

= *Xyleborus novateutonicus* Schedl, 1954b: 47.

• *Euwallacea praevius* (Blandford, 1894c: 110) (*Xyleborus*).

• *Euwallacea procerrissimus* (Schedl, 1942b: 194) (*Xyleborus*).

• *Euwallacea pseudobarbatus* (Schedl, 1942b: 193) (*Xyleborus*).

• *Euwallacea pseudorudis* (Schedl, 1951i: 62) (*Xyleborus*).

• *Euwallacea quadraticollis* (Eggers, 1923b: 197) (*Xyleborus*).

= *Xyleborus duplicatus* Schedl, 1933b: 102.

• *Euwallacea rudis* (Eggers, 1932c: 192) (*Xyleborus*).

• *Euwallacea rufoniger* (Schedl, 1934a: 89) (*Xyleborus*).

• *Euwallacea semiermis* (Schedl, 1934a: 89) (*Xyleborus*).

• *Euwallacea semipolitus* (Schedl, 1951i: 70) (*Xyleborus*).

• *Euwallacea semirudis* (Blandford, 1896e: 210) (*Xyleborus*).

= *Xyleborus dubius* Eggers, 1923b: 199.

= *Xyleborus sereinuus* Eggers, 1923b: 187.

= *Xyleborus hybridus* Eggers, 1927b: 90.

= *Xyleborus interruptus* Eggers, 1940f: 139.

= *Xyleborus neohybridus* Schedl, 1942b: 188.

= *Xyleborus longehirtus* Nunberg, 1956c: 209.

• *Euwallacea sibsagaricus* (Eggers, 1930b: 196) (*Xyleborus*).

= *Xyleborus dalbergiae* Eggers, 1930b: 196.

= *Xyleborus tonkinensis* Schedl, 1934b: 39.

• *Euwallacea similis* (Ferrari, 1867: 23) (*Xyleborus*).

= *Bostrichus ferrugineus* Boheman, 1858: 88.

= *Xyleborus parvulus* Eichhoff, 1868b: 152.

= *Xyleborus dilatatus* Eichhoff, 1878b: 393.

= *Xyleborus submarginatus* Blandford, 1896e: 223.

= *Xyleborus bucco* Schaufuss, 1897b: 212.

= *Xyleborus capito* Schaufuss, 1897b: 215.

= *Xyleborus novaguineanus* Schedl, 1936e: 530.

= *Xyleborus dilatatulus* Schedl, 1953d: 127.

• *Euwallacea simulatus* (Bright, 1972a: 80) (*Xyleborus*).

• *Euwallacea siporanus* (Hagedorn, 1910: 11) (*Xyleborus*).

• *Euwallacea solomonicus* (Schedl, 1970f: 363) (*Xyleborus*).

• *Euwallacea streblicola* Hopkins, 1915d: 55.

• *Euwallacea striatulus* (Browne, 1980c: 487) (*Xyleborus*).

• *Euwallacea strombiformis* (Schedl, 1971e: 384) (*Xyleborus*).

• *Euwallacea subalpinus* Smith, Beaver & Cognato, 2020: 256.

• *Euwallacea sulcatus* Sittichaya, Smith & Beaver in: Sittichaya et al. 2024c: 403.

• *Euwallacea temetiuicus* (Beeson, 1935a: 112) (*Xyleborus*).

• *Euwallacea testudinatus* Smith, Beaver & Cognato, 2020: 258.

• *Euwallacea timidus* (Schedl, 1973c: 76) (*Xyleborus*).

= *Xyleborus granulipes* Schedl, 1973d: 91.

• *Euwallacea trapezicollis* (Schedl, 1971e: 385) (*Xyleborus*).

• *Euwallacea validus* (Eichhoff in: Chapuis and Eichhoff 1876: 202) (*Xyleborus*).

• *Euwallacea velatus* (Sampson, 1913: 443) (*Xyleborus*).

= *Xyleborus assamensis* Eggers, 1930b: 195.

= *Xyleborus asperipennis* Eggers, 1934b: 27 (RN).

• *Euwallacea viruensis* (Browne, 1984a: 157) (*Xyleborus*).

• *Euwallacea voarotrae* (Schedl, 1961g: 146) (*Xyleborus*).

• *Euwallacea wallacei* (Blandford, 1896e: 220) (*Xyleborus*).

= *Xyleborus confinis* Eggers, 1923b: 200.

= *Xyleborus wallacei indecorus* Schedl, 1979d: 162.

• *Euwallacea xanthopus* (Eichhoff, 1868b: 151) (*Xyleborus*).

= *Xyleborus fraternus* Blandford, 1896e: 212.

= *Xyleborus kivuensis* Eggers, 1935e: 309.

= *Xyleborus artehybridus* Schedl, 1951i: 66.

• *Euwallacea zicsii* (Schedl, 1967c: 231) (*Xyleborus*).

#### *Fraudatrix* Cognato, Smith & Beaver

4 spp.

• *Fraudatrix cuneiformis* (Schedl, 1958e: 104) (*Xyleborus*).

• *Fraudatrix melas* (Eggers, 1927b: 93) (*Xyleborus*).

• *Fraudatrix pileatula* (Schedl, 1975a: 369) (*Xyleborus pileatulus*).

• *Fraudatrix simplex* (Browne, 1949: 902) (*Cryptoxyleborus*).

#### *Hadrodemius* Wood

3 spp.

• *Hadrodemius comans* (Sampson, 1919: 109) (*Xyleborus*).

= *Xyleborus amorphus* Eggers, 1926b: 147.

= *Xyleborus metacomans* Eggers, 1930b: 188.

• *Hadrodemius globus* (Blandford, 1896e: 208) (*Xyleborus*).

= *Xyleborus ursus* Eggers, 1923b: 173.

= *Xyleborus ursus fuscus* Eggers, 1923b: 174.

= *Xyleborus tomentosus* Eggers, 1939b: 10.

• *Hadrodemius pseudocomans* (Eggers, 1930b: 187) (*Xyleborus*).

= *Xyleborus artecomans* Schedl, 1953a: 24.

#### *Heteroborips* Reitter

5 spp.

• *Heteroborips cryptographus* (Ratzeburg, 1837: 160) (*Bostrichus*).

= *Bostrichus villosus* Ratzeburg, 1837: 160.

• *Heteroborips fastigatus* Smith, Beaver & Cognato, 2020: 272.

• *Heteroborips indicus* Smith, Beaver & Cognato, 2020: 273.

• *Heteroborips seriatus* (Blandford, 1894c: 111) (*Xyleborus*).

= *Xyleborus orientalis* Eggers, 1933b: 54.

= *Xyleborus todo* Kôno, 1938: 71.

= *Xyleborus orientalis aceris* Kurentsov, 1941: 188.

= *Xyleborus orientalis kalopanacis* Kurentsov, 1941: 187.

• *Heteroborips tristis* (Eggers, 1930b: 194) (*Xyleborus*).

#### *Immanus* Hulcr & Cognato

11 spp.

• *Immanus acanthurus* (Lea, 1910: 137) (*Tomicus*).

• *Immanus colossus* (Blandford, 1896e: 207) (*Xyleborus*).

= *Xyleborus szentivanyi* Schedl, 1968c: 267.

• *Immanus desectus* (Eggers, 1923b: 167) (*Xyleborus*).

= *Xyleborus desectus arduus* Schedl, 1942b: 188.

= *Xyleborus insignis* Browne, 1984c: 452.

• *Immanus duploarmatus* (Browne, 1962a: 204) (*Xyleborus*).

• *Immanus papuanus* Beaver, Sittichaya & Liu, 2019a: 380.

• *Immanus permarginatus* (Schedl, 1933c: 200) (*Xyleborus*).

• *Immanus pseudocolossus* (Schedl, 1942d: 28) (*Xyleborus*).

• *Immanus sarawakensis* (Eggers, 1923b: 176) (*Xyleborus*).

• *Immanus songi* Lin, Li & Meng, 2023: 434.

• *Immanus trolaki* (Schedl, 1939b: 350) (*Xyleborus*).

• *Immanus virago* Wang, Smith & Cognato, 2020: 438.

#### *Leptoxyleborus* Wood

6 spp.

• *Leptoxyleborus machili* (Niisima, 1910: 14) (*Xyleborus*).

= *Xyleborus depressus* Eggers, 1923b: 190.

= *Xyleborus kojimai* Murayama, 1936: 143.

= *Xyleborus sejugatus* Schedl, 1942b: 188.

• *Leptoxyleborus novus* (Eggers, 1923b: 190) (*Xyleborus*).

• *Leptoxyleborus regina* Smith, Beaver & Cognato in: Smith et al. 2022: 17.

• *Leptoxyleborus semigranulatus* (Schedl, 1931a: 340) (*Xyleborus*).

= *Xyleborus artemarginatus* Schedl, 1975g: 456.

• *Leptoxyleborus sordicauda* (Motschulsky, 1863: 514) (*Phloeotrogus*).

= *Phloeotrogus attenuatus* Motschulsky, 1863: 514.

= *Xyleborus concisus* Blandford, 1894c: 107.

= *Xyleborus marginatus* Eggers, 1927b: 91.

= *Xyleborus sordicaudulus* Eggers, 1927b: 91.

= *Xyleborus incurvus* Eggers, 1930b: 197.

= *Xyleborus sordicaudulus peguensis* Eggers, 1930b: 198.

• *Leptoxyleborus sublinearis* (Eggers, 1940f: 148) (*Xyleborus*).

#### *Melanesicus* Beaver & Petrov

6 spp.

• *Melanesicus caledoniae* (Beaver & Liu, 2016: 26) (*Xyleborus*).

• *Melanesicus deformatus* (Browne, 1974a: 70) (*Xyleborus*).

• *Melanesicus granulosus* (Schedl, 1975a: 366) (*Xyleborus*).

• *Melanesicus nukuruanus* Beaver in: Beaver et al. 2021: 167.

• *Melanesicus partitus* (Browne, 1974a: 69) (*Xyleborus*).

• *Melanesicus tishechkini* Petrov in: Beaver et al. 2021: 165.

#### *Microperus* Wood

48 spp.

• *Microperus abbreviatus* (Schedl, 1942b: 195) (*Xyleborus*).

• *Microperus alpha* (Beeson, 1929: 239) (*Xyleborus*).

• *Microperus amphicauda* (Browne, 1986b: 666) (*Xyleborus*).

• *Microperus armaticeps* (Schedl, 1942b: 198) (*Xyleborus*).

• *Microperus bidentatus* Smith & Beaver in: Sittichaya et al. 2021: 195.

• *Microperus borneensis* (Browne, 1986a: 92) (*Xyleborus*).

• *Microperus bucolicus* Sittichaya, Smith & Beaver in: Sittichaya et al. 2021: 197.

• *Microperus calamoides* (Murayama, 1934a: 291) (*Xyleborus*).

• *Microperus chimbui* (Schedl, 1973d: 90) (*Xyleborus*).

• *Microperus chrysophylli* (Eggers, 1930b: 205) (*Xyleborus*).

• *Microperus comptus* (Sampson, 1919: 111) (*Xyleborus*).

• *Microperus corporaali* (Eggers, 1923b: 210) (*Xyleborus*).

• *Microperus cruralis* (Schedl, 1975j: 456) (*Xyleborus*).

• *Microperus diversicolor* (Eggers, 1923b: 202) (*Xyleborus*).

= *Xyleborus myristicae* Schedl, 1939j: 39.

= *Xyleborus brevipilosus* Eggers, 1940f: 145.

= *Xyleborus theae* Eggers, 1940f: 144.

= *Xyleborus cylindripennis* Schedl, 1954c: 152.

= *Xyleborus atavus* Schedl, 1979e: 104.

• *Microperus eucalypticus* (Schedl, 1938g: 51) (*Xyleborus*).

• *Microperus exsculptus* (Eggers, 1927b: 101) (*Xyleborus*).

= *Xyleborus dentipennis* Browne, 1983b: 558.

• *Microperus fragosus* (Schedl, 1942d: 41) (*Xyleborus*).

• *Microperus fulvulus* (Schedl, 1942d: 35) (*Xyleborus*) (RN).

= *Xyleborus fulvus* Schedl, 1939j: 48.

• *Microperus globodeclivis* Sittichaya, Smith & Beaver in: Sittichaya et al. 2021: 198.

• *Microperus gorontalosus* (Schedl, 1939j: 50) (*Xyleborus*).

• *Microperus intermedius* (Eggers, 1923b: 201) (*Xyleborus*).

= *Xyleborus nitellus* Browne, 1984e: 97.

• *Microperus kadoyamaensis* (Murayama, 1934a: 290) (*Xyleborus*).

= *Xyleborus denseseriatus* Eggers, 1941c: 225.

= *Xyleborus nameranus* Murayama, 1954: 194.

= *Xyleborus pubipennis* Schedl, 1974d: 263.

= *Xyleborus huangi* Browne, 1983a: 34.

• *Microperus kirishimanus* (Murayama, 1955: 85) (*Coptodryas*).

• *Microperus latesalebrinus* Smith, Beaver & Cognato, 2020: 292.

• *Microperus leprosulus* (Schedl, 1936c: 27) (*Xyleborus*).

• *Microperus micrographus* (Schedl, 1958e: 103) (*Xyleborus*).

= *Xyleborus decorus* Schedl, 1960: 110 (RN).

• *Microperus minax* Smith, Beaver & Cognato, 2020: 293.

• *Microperus molestus* Park & Smith in: Park et al. 2020: 218.

• *Microperus nanus* (Browne, 1949: 903) (*Cryptoxyleborus*).

= *Xyleborus caelator* Browne, 1955: 353 (RN).

• *Microperus nudibrevis* (Schedl, 1942b: 195) (*Xyleborus*).

• *Microperus nugax* (Schedl, 1939b: 353) (*Xyleborus*).

= *Xyleborus pertuberculatus* Eggers, 1940f: 144.

• *Microperus parvus* (Lea, 1894: 321) (*Xylopertha parva*).

= *Xyleborus liber* Eggers, 1923b: 202.

= *Xyleborus bismarcensis* Browne, 1966: 255.

• *Microperus pasohanthus* Smith, Beaver & Cognato in: Smith et al. 2025: 77.

• *Microperus pedellus* (Schedl, 1969a: 213) (*Xyleborus*).

• *Microperus perparvus* (Sampson, 1922b: 151) (*Xyleborus*).

= *Xyleborus tsukubanus* Murayama, 1954: 195.

• *Microperus pometianus* (Schedl, 1939b: 354) (*Xyleborus*).

• *Microperus popondettae* (Browne, 1970: 573) (*Xyleborus*).

= *Xyleborus doctus* Schedl, 1975a: 364.

• *Microperus pullus* (Schedl, 1952g: 370) (*Xyleborus*).

• *Microperus pusillus* (Eggers, 1927b: 108) (*Webbia*).

• *Microperus quercicola* (Eggers, 1926b: 146) (*Xyleborus*).

= *Xyleborus izuensis* Murayama, 1952: 16.

= *Xyleborus semistriatus* Schedl, 1971e: 382.

• *Microperus recidens* (Sampson, 1923: 287) (*Xyleborus*).

= *Xyleborus minusculus* Eggers, 1923b: 212.

= *Xyleborus minutissimus* Eggers, 1930b: 204.

= *Xyleborus crassitarsus* Schedl, 1936c: 28.

= *Xyleborus artegraphus* Schedl, 1942d: 44.

= *Xyleborus extensus* Schedl, 1955e: 301.

= *Xyleborus tuberculosus* Browne, 1981b: 602.

• *Microperus sagmatus* Smith, Beaver & Cognato, 2020: 300.

• *Microperus serratus* Sittichaya, Smith & Beaver in: Sittichaya et al. 2021: 200.

• *Microperus spicatulus* (Browne, 1986b: 667) (*Xyleborus*).

• *Microperus tenellus* (Schedl, 1959i: 501) (*Xyleborus*).

• *Microperus truncatipennis* (Schedl, 1962e: 92) (*Xyleborus*).

• *Microperus undulatus* (Sampson, 1919: 111) (*Xyleborus*).

• *Microperus vafer* (Schedl, 1958a: 880) (*Xyleborus*).

#### *Planiculus* Hulcr & Cognato

10 spp.

• *Planiculus angulatus* Sittichaya & Cognato, 2024: 483.

• *Planiculus aries* (Schedl, 1970h: 215) (*Xyleborus*).

• *Planiculus bicolor* (Blandford, 1894c: 113) (*Xyleborus*).

= *Xyleborus laevis* Eggers, 1923b: 201.

= *Xyleborus bicolor unimodus* Beeson, 1929: 238.

= *Xyleborus rodgeri* Beeson, 1930: 213.

= *Xyleborus rodgeri privatus* Beeson, 1930: 213.

= *Xyleborus rameus* Schedl, 1940b: 441.

= *Xyleborus artelaevis* Schedl, 1942b: 196.

= *Xyleborus ashuensis* Murayama, 1954: 193.

= *Xyleborus filiformis* Schedl, 1975a: 364.

= *Xyleborus tumidus* Schedl, 1975a: 371.

= *Xyleborus glabratulus* Browne, 1983b: 560.

• *Planiculus immersus* (Schedl, 1972j: 52) (*Xyleborus*).

= *Xyleborus hashimotoi* Browne, 1986a: 95.

• *Planiculus kororensis* (Wood, 1960a: 63) (*Xyleborus*).

• *Planiculus limatus* (Schedl, 1936d: 65) (*Xyleborus*).

= *Xyleborus subemarginatus* Eggers, 1940f: 150.

= *Xyleborus subparallelus* Eggers, 1940f: 151.

• *Planiculus loricatus* (Schedl, 1933c: 200) (*Xyleborus*).

• *Planiculus murudensis* (Browne, 1965: 203) (*Xyleboricus*).

• *Planiculus rodmanculus* Hulcr & Cognato, 2013: 117.

• *Planiculus shiva* (Maiti & Saha, 1986: 140) (*Xyleborus*).

#### *Pseudowebbia* Browne

6 spp.

• *Pseudowebbia armifer* (Schedl, 1942c: 185) (*Xyleborus*).

= *Xyleborus spinachius* Schedl, 1955e: 306.

• *Pseudowebbia curvata* (Browne, 1986a: 98) (*Xyleborus curvatus*).

• *Pseudowebbia percorthyla* (Schedl, 1935g: 270) (*Xyleborus percorthylus*).

• *Pseudowebbia seriata* Browne, 1963b: 56.

= *Xyleborus brownei* Schedl, 1964f: 214 (RN).

• *Pseudowebbia squamatilis* (Schedl, 1955e: 307) (*Xyleborus*).

= *Webbia denticulatus* Browne, 1983b: 561.

• *Pseudowebbia trepanicauda* (Eggers, 1923b: 170) (*Xyleborus*).

#### *Sampsonius* Eggers

21 spp.

• *Sampsonius alvarengai* Bright, 1991: 15.

• *Sampsonius buculus* Schedl, 1937b: 170.

• *Sampsonius conifer* (Hagedorn, 1905a: 549) (*Xyleborus*).

• *Sampsonius dampfi* Schedl, 1940a: 359.

= *Sampsonius costaricencis* Nunberg, 1963: 104.

• *Sampsonius detractus* Wood, 1974a: 31.

• *Sampsonius expulsus* Wood, 1974a: 31.

• *Sampsonius giganteus* Schönherr, 1994: 65.

= *Sampsonius ensifer* Wood, 2007: 373.

• *Sampsonius kuaizi* Petrov & Mandelshtam, 2009: 315.

• *Sampsonius lapidosus* Petrov & Flechtmann, 2013: 178.

• *Sampsonius lepusculus* Petrov & Flechtmann, 2013: 179.

• *Sampsonius mexicanus* Bright, 1991: 21.

• *Sampsonius militaris* Petrov & Flechtmann, 2013: 180.

• *Sampsonius pedrosai* Schönherr, 1994: 67.

• *Sampsonius pennatus* Schedl, 1973b: 171.

• *Sampsonius prolongatus* Schönherr, 1994: 66.

• *Sampsonius quadrispinosus* Eggers, 1935c: 158.

• *Sampsonius reticulatus* Bright, 1972b: 1369.

• *Sampsonius sagittarius* Petrov & Mandelshtam, 2009: 316.

• *Sampsonius sexdentatus* (Eggers, 1933f: 23) (Sampsonius*).

• *Sampsonius sulcatus* Bright, 1981b: 163.

• *Sampsonius usurpatus* Wood, 1974a: 32.

#### *Schedlia* Browne

6 spp.

• *Schedlia allecta* (Schedl, 1942d: 33) (*Xyleborus*).

• *Schedlia brownei* Bright, 1980: 369.

• *Schedlia praeusta* (Eggers, 1923b: 167) (*Xyleborus praeustus*).

• *Schedlia sumatrana* (Hagedorn, 1908: 381) (*Xyleborus sumatranus*).

• *Schedlia usitata* (Schedl, 1942c: 188) (*Xyleborus usitatus*).

= *Schedlia convexa* Bright, 1980: 370.

= *Schedlia paraconvexa* Bright, 1980: 370.

• *Schedlia vulpina* (Schedl, 1942b: 192) (*Xyleborus*).

#### *Stictodex* Hulcr & Cognato

4 spp.

• *Stictodex dimidiatus* (Eggers, 1927e: 404) (*Xyleborus*).

= *Xyleborus dorsosulcatus* Beeson, 1930: 219.

= *Xyleborus tunggali* Schedl, 1936c: 32.

= *Xyleborus decumans* Schedl, 1953f: 301.

= *Xyleborus cruciatus* Schedl, 1973d: 90.

= *Xyleborus spicatus* Browne, 1986a: 95.

• *Stictodex halli* (Schedl, 1954e: 161) (*Xyleborus*).

= *Xyleborus cuspidus* Schedl, 1975a: 363.

• *Stictodex rimulosus* (Schedl, 1959i: 508) (*Xyleborus*).

• *Stictodex sulcicauda* (Schedl, 1972i: 271) (*Xyleborus*).

= *Xyleborus tenuipennis* Browne, 1974a: 71.

#### *Streptocranus* Schedl

13 spp.

• *Streptocranus bicolor* Browne, 1949: 900.

• *Streptocranus bicuspis* (Eggers, 1940f: 153) (*Xyleborus*).

= *Streptocranus recurvus* Browne, 1949: 898.

• *Streptocranus bispinus* (Schedl, 1979e: 104) (*Xyleborus*).

• *Streptocranus capucinulus* Schedl, 1942d: 27.

= *Streptocranus penangensis* Browne, 1950: 645.

• *Streptocranus forficatus* (Schedl, 1957b: 108) (*Xyleborus*).

• *Streptocranus fragilis* Browne, 1949: 901.

• *Streptocranus longicauda* Browne, 1962a: 206.

• *Streptocranus longicollis* (Browne, 1977a: 61) (*Xyleborus*).

• *Streptocranus longispinis* Browne, 1986a: 91.

• *Streptocranus mirabilis* Schedl, 1939j: 53.

• *Streptocranus petilus* Smith, Beaver & Cognato, 2020: 315.

• *Streptocranus superbus* (Schedl, 1951i: 95) (*Xyleborus*).

= *Xyleborus superbulus* Schedl, 1958i: 148 (RN).

• *Streptocranus usagaricus* (Eggers, 1922c: 172) (*Xyleborus*).

= *Streptocranus hendricksi* Schedl, 1953e: 245.

= *Xyleborus fallaciousus* Schedl, 1957b: 114.

= *Xyleborus monticolus* Schedl, 1957b: 113.

= *Xyleborus usagaricus hembebitakei* Schedl, 1963g: 567.

#### *Taurodemus* Wood

15 spp.

• *Taurodemus bicornutus* (Wood, 1974a: 33) (*Xyleborus*).

• *Taurodemus colombianus* Wood, 2007: 463.

• *Taurodemus ebenus* (Wood, 1971a: 39) (*Xyleborus*).

• *Taurodemus flavipes* (Fabricius, 1801: 388) (*Bostrichus*).

= *Amphicranus perebeae* Ferrari, 1869: 252.

• *Taurodemus godmani* (Blandford, 1898a: 197) (*Xyleborus*).

= *Xyleborus caelebs* Blandford, 1898a: 198.

• *Taurodemus militaris* Petrov, 2020b: 402.

• *Taurodemus pandulus* (Wood, 1974a: 34) (*Xyleborus*).

• *Taurodemus peruanus* Petrov, 2020b: 404.

• *Taurodemus ruber* (Eichhoff, 1868b: 146) (*Xyleborus*).

• *Taurodemus salvini* (Blandford, 1898a: 200) (*Xyleborus*).

• *Taurodemus sanguinicollis* (Blandford, 1898a: 198) (*Xyleborus*).

• *Taurodemus sharpi* (Blandford, 1898a: 199) (*Xyleborus*).

= *Xyleborus sharpi lenis* Wood, 1974a: 35.

• *Taurodemus splendidus* (Schaufuss, 1897a: 111) (*Xyleborus*).

= *Xyleborus camopinus* Hagedorn, 1905a: 549.

• *Taurodemus varians* (Fabricius, 1801: 386) (*Bostrichus*).

= *Bostrichus serratus* Fabricius, 1801: 388.

= *Bostrichus unidentatus* Fabricius, 1801: 386.

= *Xyleborus insignis* Eichhoff, 1869: 282.

= *Xyleborus perversus* Hagedorn, 1905b: 414.

• *Taurodemus varulus* (Wood, 1974a: 35) (*Xyleborus*).

#### *Terminalinus* Hopkins

18 spp.

• *Terminalinus anisopterae* (Browne, 1983b: 558) (*Xyleborus*).

• *Terminalinus dipterocarpi* Hopkins, 1915d: 58.

• *Terminalinus granurus* (Browne, 1980a: 376) (*Xyleborus*).

• *Terminalinus indigens* (Schedl, 1955e: 303) (*Xyleborus*).

• *Terminalinus indonesianus* (Browne, 1984b: 290) (*Xyleborus*).

• *Terminalinus macropterus* (Schedl, 1935g: 271) (*Xyleborus*).

• *Terminalinus major* (Stebbing, 1909: 19) (*Phloeosinus*).

= *Xyleborus siclus* Schedl, 1936c: 26.

• *Terminalinus moluccanus* (Browne, 1985b: 292) (*Xyleborus*).

= *Xyleborus teminabani* Browne, 1986b: 668.

• *Terminalinus pilifer* (Eggers, 1923b: 178) (*Xyleborus*).

• *Terminalinus posticepilosus* (Schedl, 1951i: 92) (*Xyleborus*).

= *Ozopemon major* Strohmeyer, 1911b: 23.

• *Terminalinus pseudomajor* (Schedl, 1951i: 93) (*Xyleborus*).

• *Terminalinus pseudopilifer* (Schedl, 1936b: 11) (*Xyleborus*).

• *Terminalinus sexspinatus* (Schedl, 1935g: 271) (*Xyleborus*).

• *Terminalinus sublongus* (Eggers, 1927b: 99) (*Xyleborus*).

• *Terminalinus sulcinoides* (Schedl, 1974a: 463) (*Xyleborus*).

• *Terminalinus takeharai* (Browne, 1983b: 557) (*Xyleborus*).

• *Terminalinus terminaliae* Hopkins, 1915d: 58.

• *Terminalinus xanthophyllus* (Schedl, 1942b: 195) (*Xyleborus*).

#### *Tricosa* Cognato, Smith & Beaver

9 spp.

• *Tricosa abberrans* (Schedl, 1959i: 502) (*Xyleborus*).

• *Tricosa cattienensis* Cognato, Smith & Beaver, 2020: 548.

• *Tricosa hipparion* Smith, Beaver & Cognato in: Smith et al. 2022: 18.

• *Tricosa indochinensis* Cognato, Smith & Beaver, 2020: 549.

• *Tricosa jacula* Cognato, Smith & Beaver, 2020: 549.

• *Tricosa mangoensis* (Schedl, 1942b: 189) (*Xyleborus*).

• *Tricosa metacuneolus* (Eggers, 1940f: 150) (*Xyleborus*).

= *Xyleborus kaimochii* Nobuchi, 1981c: 143.

• *Tricosa sokanovskii* Petrov, 2023: 525.

• *Tricosa uniseriata* Sittichaya & Cognato, 2023: 99.

#### *Truncaudum* Hulcr & Cognato

10 spp.

• *Truncaudum agnatum* (Eggers, 1923b: 197) (*Xyleborus agnatus*).

= *Xyleborus polyodon* Eggers, 1923b: 196.

= *Xyleborus nutans* Schedl, 1942b: 199.

= *Xyleborus delicatus* Schedl, 1955e: 300.

= *Xyleborus gratiosus* Schedl, 1975a: 366.

= *Cyclorhipidion subagnatum* Wood, 1992: 85 (RN).

• *Truncaudum baculum* (Beeson, 1929: 247) (*Xyleborus*).

• *Truncaudum bullatum* Smith, Beaver & Cognato, 2020: 321.

• *Truncaudum circumcinctum* (Schedl, 1941c: 401) (*Premnobius circumcinctus*).

• *Truncaudum impexum* (Schedl, 1942c: 184) (*Xyleborus impexus*).

= *Xyleborus falcarius* Schedl, 1942c: 183.

= *Xyleborus circumspinosus* Schedl, 1972j: 52.

= *Xyleborus vernaculus* Schedl, 1975a: 372.

= *Xyleborus dentatulus* Browne, 1981a: 131.

= *Xyleborus putputensis* Browne, 1986a: 97.

= *Xyleborus subdentatulus* Browne, 1986a: 97.

• *Truncaudum leverense* (Browne, 1986b: 667) (*Xyleborus leverensis*).

• *Truncaudum longius* (Eggers, 1923b: 171) (*Xyleborus longior*).

= *Xyleborus viaticus* Schedl, 1974a: 464.

= *Xyleborus protii* Browne, 1984e: 101.

= *Xyleborus canarivorus* Browne, 1986a: 96.

• *Truncaudum truncaticauda* (Browne, 1984e: 100) (*Xyleborus*).

• *Truncaudum truncatiformis* (Eggers, 1923b: 170) (*Xyleborus*).

• *Truncaudum tuberculifer* (Eggers, 1923b: 195) (*Xyleborus*).

= *Xyleborus hopeae* Browne, 1986a: 96.

#### *Webbia* Hopkins

41 spp.

• *Webbia aculeata* Sittichaya, Smith & Beaver, 2023b: 49.

• *Webbia bakoensis* Browne, 1962b: 310.

• *Webbia bicornis* (Schedl, 1939b: 349) (*Xelyborus*).

• *Webbia biformis* Browne, 1958: 496.

• *Webbia bituberculata* Browne, 1977b: 369 (E).

• *Webbia ceylonae* Schedl, 1959i: 493.

• *Webbia circumcisa* Schedl, 1975a: 373 (E).

• *Webbia cornuta* Schedl, 1942b: 183 (E).

• *Webbia cylindrica* Schedl, 1942b: 182 (E).

• *Webbia dasyura* Browne, 1981a: 133 (E).

• *Webbia dentata* Eggers, 1927b: 108 (E).

• *Webbia dipterocarpi* Hopkins, 1915c: 223.

= *Webbia octodecimspinatus* Sampson, 1921: 34.

• *Webbia dissimilis* (Eggers, 1923b: 214) (*Xyleboricus*).

= *Webbia costulatulus* Schedl, 1953f: 299.

• *Webbia diversicauda* Browne, 1972: 26.

• *Webbia divisa* Browne, 1972: 25 (E).

• *Webbia duodecimspinata* Schedl, 1942b: 182 (E).

• *Webbia gracilis* Browne, 1972: 27.

• *Webbia granulosa* Sittichaya, Smith & Beaver, 2023b: 51.

• *Webbia hatanakai* Browne, 1986a: 99.

= *Webbia turbinata* Maiti & Saha, 1986: 104 (E).

• *Webbia kuchingensis* (Browne, 1955: 348) (*Xyleborus*).

• *Webbia multidentata* Browne, 1984c: 453 (E).

• *Webbia obtusipennis* Schedl, 1966e: 38.

• *Webbia obtusispinosa* Schedl, 1939b: 348 (E).

• *Webbia orbicularis* Schedl, 1970f: 361.

• *Webbia orbiculata* (Eggers, 1923b: 213) (*Xyleboricus orbiculatus*).

• *Webbia pabo* Sampson, 1922b: 150.

• *Webbia penicillata* (Hagedorn, 1910: 7) (*Xyleborus penicillatus*).

• *Webbia philippinensis* Schedl, 1971e: 374.

• *Webbia picicauda* Schedl, 1979c: 129.

• *Webbia piscecauda* Browne, 1962a: 210.

• *Webbia planicauda* Beaver, Sittichaya & Smith in: Sittichaya et al. 2023b: 53.

• *Webbia platypoides* Eggers, 1927b: 105.

• *Webbia quadricincta* Schedl, 1972k: 62 (E).

• *Webbia quattuordecimspinata* Sampson, 1921: 34 (E).

= *Webbia quattuordecimspinatus* Schedl, 1942b: 182.

= *Webbia quattuordecimcostatus* Schedl, 1952h: 61 (RN).

= *Webbia sampsoni* Nunberg, 1956c: 209 (RN).

• *Webbia sarawakensis* Schedl, 1964i: 245.

• *Webbia spinipennis* Schedl, 1970f: 360.

• *Webbia spinosulcata* Sittichaya, Smith & Beaver, 2023b: 54.

• *Webbia subuculae* (Browne, 1962a: 209) (*Prowebbia*).

• *Webbia suturalis* Browne, 1955: 349.

• *Webbia talautica* (Eggers, 1923b: 214) (*Xyleboricus talauticus*).

• *Webbia trigintispinata* Sampson, 1922b: 149 (E).

= *Webbia 26-spinatus* Sampson, 1922b: 149.

= *Webbia vigintisexspinata* Sampson, 1922b: 149.

= *Webbia mucronata* Eggers, 1927b: 107.

= *Webbia mucronatus* Eggers, 1927b: 107.

#### *Xenoxylebora* Osborn, Smith & Cognato

14 spp.

• *Xenoxylebora addenda* Osborn, Smith & Cognato, 2022: 10.

• *Xenoxylebora calculosa* Osborn, Smith & Cognato, 2022: 10.

• *Xenoxylebora caudata* (Schedl, 1957b: 110) (*Xyleborus caudatus*).

• *Xenoxylebora collarti* (Eggers, 1932c: 300) (*Xyleborus*).

= *Xyleborus semipilosus* Eggers, 1932c: 300.

• *Xenoxylebora hystricosa* Osborn, Smith & Cognato, 2022: 12.

• *Xenoxylebora neosphenos* (Schedl, 1976: 76) (*Xyleborus*).

• *Xenoxylebora perdiligens* (Schedl, 1937e: 399) (*Xyleborus*).

• *Xenoxylebora pilosa* Osborn, Smith & Cognato, 2022: 14.

• *Xenoxylebora serrata* Osborn, Smith & Cognato, 2022: 14.

• *Xenoxylebora sphenos* (Sampson, 1912: 247) (*Xyleborus*).

= *Xyleborus perdiligens diligens* Schedl, 1954d: 79.

= *Xyleborus montanus tenellus* Schedl, 1957b: 107.

= *Xyleborus tenellus* Schedl, 1957b: 107.

• *Xenoxylebora subcrenulata* (Eggers, 1932c: 301) (*Xyleborus subcrenulatus*).

• *Xenoxylebora sulcata* Osborn, Smith & Cognato, 2022: 15.

• *Xenoxylebora syzygii* (Nunberg, 1959b: 167) (*Xyleborus*) (RN).

= *Xyleborus montanus* Schedl, 1957b: 106.

= *Xyleborus submontanus* Schedl, 1960: 106 (RN).

• *Xenoxylebora truncatula* (Schedl, 1957b: 94) (*Xyleborus truncatulus*).

#### *Xyleborinus* Reitter

95 spp.

• *Xyleborinus acanthopteron* Smith, Beaver & Cognato in: Smith et al. 2022: 19.

• *Xyleborinus aduncus* (Schedl, 1961g: 148) (*Xyleborus*).

= *Xyleborus adunculus* Schedl, 1961g: 150.

• *Xyleborinus aemulus* (Wollaston, 1869: 321) (*Tomicus*).

= *Xyleborus spinifer* Eggers, 1920b: 116.

• *Xyleborinus alienus* (Schedl, 1977a: 398) (*Xyleborus*).

• *Xyleborinus andrewesi* (Blandford, 1896e: 227) (*Xyleborus*).

= *Xyleborus mimosae* Schedl, 1957b: 109.

= *Xyleborus persephenos* Schedl, 1970g: 129.

= *Xyleborus insolitus* Bright, 1972a: 77.

= *Cryptoxyleborus gracilior* Browne, 1984e: 101.

• *Xyleborinus angustatus* (Eichhoff, 1866: 278) (*Xyleborus*).

• *Xyleborinus armatus* (Schaufuss, 1891: 30) (*Xyleborus*).

• *Xyleborinus artelineatus* (Beeson, 1929: 239) (*Xyleborus*).

= *Xyleborus validicornis* Schedl, 1950e: 52.

= *Xyleborus cinctipennis* Schedl, 1980b: 186.

• *Xyleborinus artestriatus* (Eichhoff, 1878b: 507) (*Xyleborus*).

= *Xyleborus laticollis* Blandford, 1896e: 226.

= *Xyleborus angustior* Eggers, 1925: 158.

= *Xyleborus rugipennis* Schedl, 1953f: 303.

= *Xyleborus undatus* Schedl, 1974d: 264.

= *Xyleborinus beaveri* Browne in: Beaver and Browne 1979: 603.

• *Xyleborinus attenuatus* (Blandford, 1894c: 114) (*Xyleborus*).

= *Xyleborus alni* Niisima, 1909: 160.

= *Xyleborus canus* Niisima, 1909: 161.

• *Xyleborinus bicinctus* (Schedl, 1965f: 76) (*Xyleborus*).

• *Xyleborinus bicornatulus* (Wood, 1967c: 137) (*Xyleborus*).

• *Xyleborinus buscki* (Hopkins, 1915d: 63) (*Xyleborus*).

= *Xyleborus longulus* Schedl, 1966d: 117.

• *Xyleborinus castriformis* Eliassen & Jordal, 2021: 25.

• *Xyleborinus celatus* Wood, 1974a: 43.

• *Xyleborinus clivus* Eliassen & Jordal, 2021: 23.

• *Xyleborinus cocoensis* Kirkendall & Jordal, 2006: 732.

• *Xyleborinus concavus* Eliassen & Jordal, 2021: 14.

• *Xyleborinus coronatus* Eliassen & Jordal, 2021: 17.

• *Xyleborinus cuneatus* Smith, Beaver & Cognato, 2020: 338.

• *Xyleborinus cuneidentis* (Schedl, 1961g: 151) (*Xylechinosomus*).

• *Xyleborinus cuneolosus* (Schedl, 1959i: 510) (*Xyleborus*).

• *Xyleborinus cupulatus* (Schedl, 1961g: 150) (*Xyleborus*).

• *Xyleborinus dentellus* (Schedl, 1953c: 102) (*Xyleborus*).

= *Xyleborinus forcipatus* Schedl, 1957b: 104.

• *Xyleborinus diadematus* Eliassen & Jordal, 2021: 9.

• *Xyleborinus diapiformis* (Schedl, 1961g: 155) (*Xyleborus*).

• *Xyleborinus dirus* Wood, 1974a: 41.

• *Xyleborinus disgregus* Smith, Beaver & Cognato, 2020: 340.

• *Xyleborinus dumosus* Smith, Beaver, Pham & Cognato, 2022: 20.

• *Xyleborinus echinatus* Bright, 2019: 280.

• *Xyleborinus echinopterus* Smith, Beaver & Cognato, 2020: 342.

• *Xyleborinus elongatus* Beaver & Liu, 2016: 23.

• *Xyleborinus ephialtodes* Smith, Beaver & Cognato, 2020: 344.

• *Xyleborinus exiguus* (Walker, 1859: 260) (*Bostrichus*).

= *Xyleborus muriceus* Eichhoff, 1878a: 392.

= *Xyleborus muriceus* Eichhoff, 1878b: 506.

= *Xyleborus diversus* Schedl, 1954d: 80.

= *Xyleborus perexiguus* Schedl, 1971e: 381.

= *Xyleborinus ankius* Schedl, 1975a: 361.

• *Xyleborinus forficuloides* (Schedl, 1951e: 24) (*Xelyborus*).

= *Xelyborus forficuloides dentibaris* Schedl, 1961g: 150.

= *Xelyborus forficuloides pinguis* Schedl, 1961g: 150.

• *Xyleborinus forficulus* (Eggers, 1922c: 171) (*Xyleborus*).

• *Xyleborinus gracilicornis* (Schedl, 1977d: 45) (*Xyleborus*).

• *Xyleborinus gracilis* (Eichhoff, 1868b: 149) (*Xyleborus*).

= *Xyleborus quercus* Hopkins, 1915d: 63.

= *Xyleborus aspericauda* Eggers, 1941a: 106.

= *Xyleborus neogracilis* Schedl, 1954b: 46.

= *Xyleborus schoenherri* Schedl, 1981: 5.

• *Xyleborinus heveae* (Schedl, 1957b: 97) (*Xyleborus*).

• *Xyleborinus horridulus* (Browne, 1962b: 307) (*Xyleborus*).

• *Xyleborinus howdenae* (Bright, 1973: 18) (*Xyleborus*) (RN).

= *Xyleborus novus* Bright, 1972a: 78.

= *Xyleborus brighti* Schedl, 1974b: 335 (RN).

• *Xyleborinus huifenyinae* Smith, Beaver & Cognato, 2020: 346.

• *Xyleborinus insulosus* Bright & Torres, 2006: 420.

• *Xyleborinus intersetosus* (Blandford, 1898a: 211) (*Xyleborus*).

= *Xyleborus analogus* Schedl, 1949b: 277.

• *Xyleborinus jianghuasuni* Smith, Beaver & Cognato, 2020: 348.

• *Xyleborinus kwangreungensis* Park & Smith in: Park et al. 2020: 219.

• *Xyleborinus laevipennis* Eliassen & Jordal, 2021: 19.

• *Xyleborinus linearicollis* (Schedl, 1937b: 169) (*Xyleborus*).

• *Xyleborinus longus* (Eggers, 1927e: 403) (*Xyleborus*).

• *Xyleborinus magnispinosus* Eliassen & Jordal, 2021: 26.

• *Xyleborinus marcidus* (Schedl, 1965f: 72) (*Xyleborus*).

• *Xyleborinus margo* Eliassen & Jordal, 2021: 21.

• *Xyleborinus mitosomiformis* (Schedl, 1953c: 104) (*Xyleborus*).

• *Xyleborinus mitosomus* (Schedl, 1965f: 57) (*Xyleborus*).

• *Xyleborinus namibiae* (Schedl, 1982: 285) (*Xyleborus*).

• *Xyleborinus nobuchii* Smith, Beaver & Cognato in: Smith et al. 2022: 21.

• *Xyleborinus ntsoui* Eliassen & Jordal, 2021: 19.

• *Xyleborinus octiesdentatus* (Murayama, 1931: 46) (*Xyleborus*).

• *Xyleborinus octospinosus* (Eggers, 1920c: 44) (*Xyleborus*).

= *Xyleborus mitosomipennis* Schedl, 1953c: 103.

• *Xyleborinus perpusillus* (Eggers, 1927e: 404) (*Xyleborus*).

= *Xyleborus perminutissimus* Schedl, 1934a: 90.

= *Xyleborus angustatulus* Schedl, 1942d: 43.

• *Xyleborinus pilosellus* (Schedl, 1957b: 86) (*Xyleborus*).

• *Xyleborinus polyalthiae* Schedl, 1952b: 19.

• *Xyleborinus profundus* (Schedl, 1961g: 149) (*Xyleborus aduncus profundus*).

• *Xyleborinus protinus* (Wood, 1974a: 42) (*Xyleborus*).

• *Xyleborinus pseudopityogenes* (Eggers, 1943c: 76) (*Xyleborus*).

• *Xyleborinus quadrispinis* (Schedl, 1953c: 102) (*Xyleborus*).

• *Xyleborinus quadrispinosus* (Eichhoff, 1878b: 396) (*Xyleborus*).

• *Xyleborinus reconditus* (Schedl, 1963e: 60) (*Xyleborus*).

• *Xyleborinus saginatus* Wood, 2007: 474.

• *Xyleborinus saxesenii* (Ratzeburg, 1837: 164) (*Bostrichus*).

= *Tomicus dohrnii* Wollaston, 1854: 290.

= *Tomicus decolor* Boieldieu, 1859: 473.

= *Xyleborus aesculi* Ferrari, 1867: 22.

= *Xyleborus sobrinus* Eichhoff in: Chapuis and Eichhoff 1876: 202.

= *Xyleborus frigidus* Blackburn in: Blackburn and Sharp 1885: 193.

= *Xyleborus subdepressus* Rey in: Eichhoff 1885: 142.

= *Xyleborus arbuti* Hopkins, 1915d: 64.

= *Xyleborus floridensis* Hopkins, 1915d: 63.

= *Xyleborus pecanis* Hopkins, 1915d: 63.

= *Xyleborinus libocedri* Swaine, 1934: 205.

= *Xyleborinus tsugae* Swaine, 1934: 204.

= *Xyleborus pseudogracilis* Schedl, 1937b: 169.

= *Xyleborus retrusus* Schedl, 1940c: 208.

= *Xyleborus peregrinus* Eggers, 1944a: 142.

= *Xyleborus pseudoangustatus* Schedl, 1948b: 28.

= *Xyleborus paraguayensis* Schedl, 1949b: 276.

= *Xyleborus opimulus* Schedl, 1976: 77.

• *Xyleborinus schaufussi* (Blandford, 1894c: 117) (*Xyleborus*).

= *Xyleborus kraunhiae* Niisima, 1910: 14.

• *Xyleborinus sculptilis* (Schedl, 1964i: 247) (*Xyleborus*).

• *Xyleborinus sentosus* (Eichhoff, 1868b: 146) (*Xyleborus*).

• *Xyleborinus sharpae* (Hopkins, 1915d: 63) (*Xyleborus*).

= *Xyleborus schreineri* Eggers, 1920b: 115.

• *Xyleborinus signatipennis* (Schedl, 1961g: 152) (*Xyleborus*).

• *Xyleborinus similans* (Eggers, 1940e: 237) (*Xyleborus*).

= *Xyleborus sclerocaryae* Schedl, 1962g: 71.

• *Xyleborinus singularis* Eliassen & Jordal, 2021: 14.

• *Xyleborinus speciosus* (Schedl, 1975j: 457) (*Xyleborus*).

• *Xyleborinus spiculatulus* (Schedl, 1965f: 70) (*Xyleborus*).

• *Xyleborinus spiculatus* (Schaufuss, 1891: 28) (*Xyleborus*).

• *Xyleborinus spiniger* (Schedl, 1954b: 45) (*Xyleborus*).

• *Xyleborinus spinipennis* (Eggers, 1930b: 202) (*Xyleborus*).

• *Xyleborinus spinipes* (Schedl, 1957b: 103) (*Xyleborus*).

• *Xyleborinus spiniposticus* Wood, 1992: 80 (RN).

= *Eidophelus spinipennis* Schedl, 1979g: 106.

• *Xyleborinus spinosus* (Schaufuss, 1891: 27) (*Xyleborus*).

• *Xyleborinus subgranulatus* (Eggers, 1930b: 202) (*Xyleborus*).

• *Xyleborinus subspinosus* (Eggers, 1930b: 203) (*Xyleborus*).

• *Xyleborinus subsulcatus* (Eggers, 1927c: 190) (*Xyleborus*).

• *Xyleborinus syzygii* (Schedl, 1959a: 709) (*Xyleborus*).

• *Xyleborinus thaiphami* Smith, Beaver & Cognato, 2020: 357.

• *Xyleborinus tribuloides* Wood, 1977a: 218.

• *Xyleborinus tribulosus* Wood, 1974a: 42.

• *Xyleborinus tritus* Smith, Beaver & Cognato, 2020: 360.

• *Xyleborinus tuberculatus* Eliassen & Jordal, 2021: 21.

• *Xyleborinus turritus* Eliassen & Jordal, 2021: 23.

#### *Xyleborus* Eichhoff

270 spp.

• *Xyleborus acuminatus* Schedl, 1970e: 94.

• *Xyleborus adamsoni* Beeson, 1935b: 120.

= *Xyleborus nigroaffinis* Beeson, 1940: 199.

= *Xyleborus rapanus* Beeson, 1940: 200.

• *Xyleborus adelographus* Eichhoff, 1868c: 400.

= *Xyleborus vitiosus* Schedl, 1940a: 367.

= *Xyleborus accomodatus* Schedl, 1966d: 112.

• *Xyleborus advena* Bright, 2019: 288.

• *Xyleborus aequatorensis* Eggers, 1940d: 108.

• *Xyleborus affinis* Eichhoff, 1868c: 401.

= *Xyleborus affinis fuscobrunneus* Eichhoff, 1878b: 372.

= *Xyleborus affinis mascarensis* Eichhoff, 1878b: 372.

= *Xyleborus affinis parvus* Eichhoff, 1878b: 372.

= *Xyleborus sacchari* Hopkins, 1915d: 64.

= *Xyleborus subaffinis* Eggers, 1933f: 36.

= *Xyleborus societatis* Beeson, 1935b: 120.

= *Xyleborus proximus* Eggers, 1943e: 66.

• *Xyleborus africanus* Eggers, 1927c: 194.

= *Xyleborus africanus picinus* Schedl, 1957b: 96.

• *Xyleborus agamus* Perkins, 1900: 178.

= *Xyleborus immaturus* Blackburn in: Blackburn and Sharp 1885: 193.

• *Xyleborus agraphus* Schedl, 1977c: 503.

• *Xyleborus algidus* Schedl, 1979h: 414.

• *Xyleborus altilis* Schedl, 1966d: 115.

• *Xyleborus ambasinotatus* Schedl, 1978a: 306.

• *Xyleborus ambasipennis* Schedl, 1957b: 89.

• *Xyleborus ambasiusculus* Eggers, 1920c: 41.

• *Xyleborus analis* Schedl, 1973d: 89.

• *Xyleborus angolensis* Schedl, 1959h: 25.

• *Xyleborus antaisaka* Schedl, 1953c: 100.

• *Xyleborus antanala* Schedl, 1953c: 98.

= *Xyleborus boeni* Schedl, 1953c: 99.

• *Xyleborus anthracinus* Bright, 2019: 290.

• *Xyleborus aquilus* Blandford, 1894c: 109.

• *Xyleborus araguensis* Wood, 2007: 441.

• *Xyleborus arcturus* Samuelson, 1981: 69.

• *Xyleborus artecylindrus* Schedl, 1942b: 197.

• *Xyleborus asper* Eggers, 1933f: 30.

= *Xyleborus amoenus* Schedl, 1949b: 282.

• *Xyleborus asperipunctatus* Eggers, 1933f: 35.

• *Xyleborus asperrimus* Schedl, 1975g: 457.

• *Xyleborus associatus* Schedl, 1976: 73.

• *Xyleborus astutus* Schedl, 1954c: 154.

• *Xyleborus aurilegulus* Schaufuss, 1897a: 112.

• *Xyleborus bambesanus* Eggers, 1940e: 238.

• *Xyleborus barbatoides* Schedl, 1971e: 377.

• *Xyleborus barumbuensis* Eggers, 1924: 109.

= *Xyleborus robertsi* Browne, 1963a: 247.

= *Xyleborus barumbuensis mendosus* Schedl, 1965f: 74.

• *Xyleborus basalis* Schedl, 1972a: 292.

• *Xyleborus betsileo* Schedl, 1965f: 74.

• *Xyleborus bezanozano* Schedl, 1961g: 152.

• *Xyleborus biconicus* Eggers, 1928a: 97.

= *Xyleborus bicinctus* Schedl, 1972f: 69.

= *Xyleborus bicinctulus* Schedl, 1974b: 338 (RN).

• *Xyleborus bidentatus* (Motschulsky, 1863: 514) (*Phloeotrogus*).

= *Xyleborus subcostatus* Eichhoff, 1869: 281.

= *Xyleborus riehli* Eichhoff, 1878b: 346.

= *Progenius fleutiauxi* Blandford, 1896b: 21.

= *Xyleborus laeviusculus* Blandford, 1896b: 21.

= *Boroxylon stephegynis* Hopkins, 1915d: 58.

= *Boroxylon webbi* Hopkins, 1915d: 59.

= *Xyleborus subcostatus dearmatus* Eggers, 1923b: 205.

= *Xyleborus brevidentatus* Eggers, 1930b: 190.

= *Xyleborus quadridens* Eggers, 1930b: 191.

• *Xyleborus bispinatus* Eichhoff, 1868b: 146.

• *Xyleborus bolivianus* Eggers, 1943d: 385.

= *Xyleborus parvipunctatus* Eggers, 1943d: 385.

= *Xyleborus rugosipennis* Schedl, 1963e: 61.

= *Xyleborus rugosipennis incertus* Schedl, 1963e: 63.

• *Xyleborus buxtoni* Beeson, 1929: 243.

• *Xyleborus cacuminatus* Eggers, 1928a: 98.

• *Xyleborus caldensis* Wood, 2007: 456.

• *Xyleborus carinulatus* Eggers, 1920c: 41.

• *Xyleborus catharinensis* Eggers, 1928a: 98.

• *Xyleborus celsoides* Hagedorn, 1908: 379.

• *Xyleborus celsus* Eichhoff, 1868c: 400.

= *Xyleborus biographus* LeConte, 1868: 160.

• *Xyleborus cinctipes* Schedl, 1979e: 105.

• *Xyleborus cognatus* Blandford, 1896b: 19.

• *Xyleborus commixtus* Blandford, 1898a: 208.

• *Xyleborus comparabilis* Schedl, 1957b: 91.

• *Xyleborus concentus* Wood, 1974a: 39.

• *Xyleborus confluens* Schedl, 1966d: 113.

• *Xyleborus congruens* Schedl, 1966d: 114.

• *Xyleborus conradti* Hagedorn, 1910: 8.

= *Xyleborus ambasius* Hagedorn, 1912c: 341.

= *Xyleborus carbonarius* Eggers, 1927c: 190.

• *Xyleborus convexicauda* Eggers, 1932c: 303.

• *Xyleborus costaricensis* Blandford, 1898a: 210.

= *Xyleborus nevermanni* Schedl, 1935c: 93.

• *Xyleborus costatomorphus* Schedl, 1950a: 897.

• *Xyleborus costulatus* Eggers, 1940f: 147.

• *Xyleborus cribratus* Eggers, 1940f: 145.

• *Xyleborus cribripennis* Eggers, 1927c: 191.

• *Xyleborus crinitus* Schedl, 1963g: 301.

= *Xyleborus nigericus* Browne, 1970: 552.

• *Xyleborus cuneipennis* Schedl, 1950b: 31.

• *Xyleborus curtidentis* Schedl, 1961g: 156.

• *Xyleborus declivis* Eichhoff, 1869: 280.

= *Xyleborus pseudoprocer* Schedl, 1949b: 279.

• *Xyleborus demissus* Wood, 1974a: 40.

• *Xyleborus densicornis* Schedl, 1957b: 95.

• *Xyleborus deplanatus* Eggers, 1933f: 32.

= *Xyleborus longideclivis* Wood, 1968a: 1.

• *Xyleborus derelictus* Hagedorn, 1910: 12.

• *Xyleborus desertus* Schedl, 1971e: 379.

• *Xyleborus devexipennis* Hulcr & Cognato, 2013: 146.

• *Xyleborus dichrous* (Eichhoff, 1868b: 145).

• *Xyleborus diglyptus* Schedl, 1956b: 33.

• *Xyleborus disjunctus* Bright, 2019: 292.

• *Xyleborus dissimulatus* Wood, 1974a: 38.

• *Xyleborus dorsalis* Schedl, 1965b: 27.

• *Xyleborus dryographus* (Ratzeburg, 1837: 167) (*Bostrichus*).

= *Tomicus flavus* Stephens, 1830: 356.

= *Xyleborus sampsoni* Donisthorpe, 1940: 6.

= *Xyleborus linearis* Schedl, 1949b: 273.

= *Xyleborus donisthorpi* Schedl, 1951i: 51 (RN).

• *Xyleborus dubiosus* Perkins, 1900: 177.

• *Xyleborus eichhoffianus* Schedl, 1950d: 110 (RN).

= *Xyleborus eichhoffi* Schaufuss, 1891: 25.

• *Xyleborus elongatus* Eggers, 1920c: 43.

• *Xyleborus eurygraphus* (Ratzeburg, 1837: 168) (*Bostrichus*).

• *Xyleborus exaratus* Blandford, 1898a: 206.

• *Xyleborus exsectus* Perkins, 1900: 179.

• *Xyleborus falsus* Schedl, 1966d: 116.

• *Xyleborus ferox* Blandford, 1898a: 201.

• *Xyleborus ferrugineus* (Fabricius, 1801: 388) (*Bostrichus*).

= *Xyleborus confusus* Eichhoff, 1868c: 401.

= *Xyleborus fuscatus* Eichhoff, 1868c: 400.

= *Xyleborus retusicollis* Zimmermann, 1868: 146.

= *Tomicus trypanaeoides* Wollaston, 1868: 114.

= *Xyleborus amplicollis* Eichhoff, 1869: 280.

= *Xyleborus insularis* Sharp in: Blackburn and Sharp 1885: 193.

= *Xyleborus tanganus* Hagedorn, 1910: 8.

= *Xyleborus nyssae* Hopkins, 1915d: 66.

= *Xyleborus soltaui* Hopkins, 1915d: 66.

= *Xyleborus hopkinsi* Beeson, 1929: 246.

= *Xyleborus argentinensis* Schedl, 1931a: 345.

= *Xyleborus rufopiceus* Eggers, 1932c: 303.

= *Xyleborus schedli* Eggers, 1934a: 83.

= *Xyleborus nesianus* Beeson, 1940: 200.

= *Xyleborus notatus* Eggers, 1941a: 107.

= *Xyleborus subitus* Schedl, 1949b: 280.

• *Xyleborus festivus* Eichhoff in: Chapuis and Eichhoff 1876: 202.

= *Xyleborus pinicola* Eggers, 1930b: 206.

= *Xyleborus detectus* Schedl, 1975g: 458.

= *Xyleborus pinivorus* Browne, 1980a: 374.

• *Xyleborus ficus* Eggers, 1927c: 193.

• *Xyleborus figuratus* Schedl, 1959i: 508.

• *Xyleborus foederatus* Schedl, 1963e: 58.

• *Xyleborus fuyugei* Schedl, 1973c: 74.

• *Xyleborus geayi* Hagedorn, 1905b: 413.

• *Xyleborus gezei* Lepesme, 1942a: 120.

• *Xyleborus gibber* Schedl, 1961g: 145.

• *Xyleborus glabratus* Eichhoff, 1877a: 127.

= *Xyleborus kumamotoensis* Murayama, 1934a: 288.

• *Xyleborus gracilipennis* Schedl, 1957b: 106.

• *Xyleborus granatus* Schedl, 1979d: 161.

• *Xyleborus grandis* Eichhoff, 1869: 281.

• *Xyleborus graniger* Schedl, 1955e: 303.

• *Xyleborus gratus* Schedl, 1964i: 248.

• *Xyleborus grosmanni* Schedl, 1952e: 362.

• *Xyleborus hawaiiensis* Perkins, 1900: 175.

• *Xyleborus hiiaka* Samuelson, 1981: 75.

• *Xyleborus horridatus* Wood, 1967c: 135.

• *Xyleborus horridicus* Wood, 1967c: 136.

• *Xyleborus horridus* Eichhoff, 1869: 282.

= *Xyleborus flohri* Schedl, 1972f: 69.

• *Xyleborus hova* Schedl, 1953c: 95.

• *Xyleborus hystricoides* Browne, 1973: 293.

• *Xyleborus ignobilis* Perkins, 1900: 180.

• *Xyleborus iheringi* (Iglesias, 1914: 129).

• *Xyleborus imbellis* Blandford, 1898a: 211.

• *Xyleborus impressus* Eichhoff, 1868c: 400.

• *Xyleborus improvidus* Schedl, 1935c: 92.

= *Xyleborus aclinis* Wood, 1974a: 38.

• *Xyleborus incertus* Schedl, 1963e: 63 (*Xyleborus rugosipennis incertus*).

= *Xyleborus oneratus* Schedl, 1976: 76.

• *Xyleborus inconstans* Schedl, 1963g: 271.

• *Xyleborus inferior* Schedl, 1976: 74.

• *Xyleborus inopinatus* Schedl, 1970e: 94.

• *Xyleborus insidiosus* Cognato & Smith in: Cognato et al. 2019: 1280.

• *Xyleborus insignis* Browne, 1984c: 452.

• *Xyleborus integer* Schedl, 1957b: 93.

• *Xyleborus interpunctatus* Blandford, 1898a: 206.

• *Xyleborus intrusus* Blandford, 1898a: 213.

= *Xyleborus fitchi* Hopkins, 1915d: 66.

= *Xyleborus howardi* Hopkins, 1915d: 65.

= *Xyleborus scopulorum* Hopkins, 1915d: 66.

• *Xyleborus inurbanus* (Broun, 1880: 346) (*Apate*).

• *Xyleborus irregularis* Eggers, 1923b: 211.

• *Xyleborus jambolanaensis* Schedl, 1951e: 22.

• *Xyleborus judenkoi* Schedl, 1959i: 507.

• *Xyleborus katangensis* Eggers, 1932c: 297.

= *Xyleborus rhodesianus* Eggers, 1936c: 40.

= *Xyleborus luteus* Schedl, 1937e: 403.

• *Xyleborus kauaiensis* Perkins, 1900: 174.

• *Xyleborus laciniatus* Hagedorn, 1910: 7.

• *Xyleborus lacunatus* Wood, 1974a: 37.

• *Xyleborus lanaiensis* Perkins, 1900: 176.

• *Xyleborus latipennis* Schedl, 1976: 75.

• *Xyleborus littoralis* Perkins, 1900: 179.

• *Xyleborus longipennis* Eggers, 1933f: 25.

• *Xyleborus lubricus* Schedl, 1941c: 404.

• *Xyleborus macer* Blandford, 1898a: 218.

• *Xyleborus madagascariensis* Schaufuss, 1891: 23.

• *Xyleborus magnificus* Wood, 1992: 87.

• *Xyleborus magnispinatus* Beaver in: Beaver and Löyttyniemi 1985: 75.

• *Xyleborus majusculus* Schedl, 1951c: 124.

= *Xyleborus cachoeirinhae* Schedl, 1951c: 125.

• *Xyleborus malgasicus* Schedl, 1961g: 148 (*Xyleborus subtuberculatus malgasicus*).

= *Xyleborus similaris* Schedl, 1965f: 71.

• *Xyleborus mambillae* Browne, 1970: 573.

• *Xyleborus maronicus* Eggers, 1933f: 35.

• *Xyleborus mauiensis* Perkins, 1900: 175.

• *Xyleborus meritus* Wood, 1974a: 40.

• *Xyleborus mimicus* Wood, 2007: 440.

• *Xyleborus mindanaensis* Eggers, 1927b: 93.

• *Xyleborus molokaiensis* Perkins, 1900: 174.

• *Xyleborus monographus* (Fabricius, 1792: 365) (*Bostrichus*).

= *Bostrichus tuberculosus* Herbst, 1793: 113.

= *Xyleborus monographus nitidipennis* Roubal, 1937: 67.

• *Xyleborus morulus* Blandford, 1898a: 212.

• *Xyleborus multispinatus* Eggers, 1920b: 125.

= *Xyleborus acanthus* Schedl, 1952b: 15.

= *Xyleborus acanthus acanthodes* Schedl, 1954d: 80.

= *Xyleborus acanthus acuticornis* Schedl, 1957b: 97.

= *Xyleborus acanthus minimus* Schedl, 1971d: 13.

• *Xyleborus mumfordi* Beeson, 1935a: 110.

• *Xyleborus mustus* Schedl, 1972i: 269.

• *Xyleborus mutabilis* Schedl, 1935c: 92.

= *Xyleborus itatiayaensis* Schedl, 1936a: 109.

= *Xyleborus meridensis* Wood, 1974a: 38.

• *Xyleborus muticus* Blandford, 1894c: 112.

= *Xyleborus lignographus* Schedl, 1953a: 28.

= *Xyleborus conditus* Schedl, 1971e: 379.

• *Xyleborus mysticulus* Cognato & Smith in: Cognato et al. 2019: 1281.

• *Xyleborus nagaoensis* Murayama, 1934a: 294.

• *Xyleborus neivai* Eggers, 1928a: 96.

• *Xyleborus neoscabridus* Schedl, 1972i: 269.

• *Xyleborus neotruncatus* Schedl, 1978a: 307.

• *Xyleborus neptunus* Schaufuss, 1891: 22.

• *Xyleborus nigrescens* Browne, 1980b: 387.

• *Xyleborus nigropilosus* Eggers, 1943e: 67.

• *Xyleborus nitidulus* Eggers, 1927c: 192.

• *Xyleborus norfolkensis* Schedl, 1972i: 270.

• *Xyleborus nossi* Schedl, 1961g: 146.

• *Xyleborus nubilus* Samuelson, 1981: 80.

• *Xyleborus oahuensis* Perkins, 1900: 177.

• *Xyleborus obliquus* Sharp in: Blackburn and Sharp 1885: 192.

= *Xyleborus tantalus* Schedl, 1941b: 114.

= *Xyleborus oblicatus* Schedl, 1980c: 122 (RN).

• *Xyleborus oblongus* Schedl, 1950b: 32.

• *Xyleborus ohtoensis* Nobuchi, 1981c: 149.

• *Xyleborus opacus* Smith, Beaver & Cognato, 2020: 374.

• *Xyleborus operosus* Schedl, 1973d: 91.

• *Xyleborus palatus* Wood, 1974a: 35.

• *Xyleborus papuanus* Blandford, 1896e: 209.

• *Xyleborus parallelocollis* Eggers, 1933f: 33.

• *Xyleborus parallelus* Eggers, 1936c: 39.

• *Xyleborus parcellus* Wood, 1968a: 2.

• *Xyleborus parinariae* Schedl, 1963g: 306.

• *Xyleborus pele* Samuelson, 1981: 82.

• *Xyleborus pentaclethrae* Schedl, 1957b: 87.

• *Xyleborus peramplus* Schedl, 1950b: 31.

• *Xyleborus perforans* (Wollaston, 1857: 96) (*Tomicus*).

= *Bostrichus testaceus* Walker, 1859: 260.

= *Bostrichus duponti* Montrouzier, 1861: 265.

= *Anodius denticulus* Motschulsky, 1863: 512.

= *Anodius tuberculatus* Motschulsky, 1863: 511.

= *Xyleborus kraatzi* Eichhoff, 1868b: 152.

= *Xyleborus kraatzii philippinensis* Eichhoff, 1878b: 374.

= *Xyleborus immaturus* Blackburn in: Blackburn and Sharp 1885: 193.

= *Xylopertha hirsuta* Lea, 1894: 321.

= *Xyleborus whitteni* Beeson, 1935a: 113.

= *Xyleborus apertus* Schedl, 1939b: 355.

= *Xyleborus criticus* Schedl, 1950a: 899.

= *Xyleborus cylindrus* Schedl, 1951i: 94.

= *Xyleborus shionomisakiensis* Murayama, 1951: 3.

= *Xyleborus minimus* Schedl, 1955e: 305.

• *Xyleborus perlongus* Eggers, 1943d: 386.

= *Xyleborus pulcnerrimus* Schedl, 1949a: 38.

= *Xyleborus pulchripes* Schedl, 1958j: 46.

• *Xyleborus pernotus* Schedl, 1934a: 88.

• *Xyleborus peruvianus* Schedl, 1951c: 123.

• *Xyleborus pfeilii* (Ratzeburg, 1837: 168) (*Bostrichus*).

= *Bostrichus alni* Mulsant & Rey, 1857: 111.

= *Xyleborus vicarius* Eichhoff in: Chapuis and Eichhoff 1876: 203.

= *Xyleborus adumbratus* Blandford, 1894c: 115.

= *Xyleborus septentrionalis* Niisima, 1909: 162.

• *Xyleborus pilipunctatus* Browne, 1966: 251.

• *Xyleborus planicollis* Zimmermann, 1868: 145.

• *Xyleborus pleiades* Samuelson, 1981: 84.

• *Xyleborus politus* Hagedorn, 1905b: 413.

• *Xyleborus posticegranulatus* Schedl, 1938d: 462.

• *Xyleborus pourriensis* Schedl, 1950d: 110.

• *Xyleborus praestans* Wood, 1980b: 358.

• *Xyleborus princeps* Blandford, 1898a: 208.

= *Xyleborus spathipennis ohausi* Hagedorn, 1912c: 345.

• *Xyleborus principalis* Eichhoff, 1878b: 357.

= *Xyleborus alluaudi* Schaufuss, 1897b: 210.

= *Xyleborus camerunus* Hagedorn, 1910: 9.

= *Xyleborus camerunus rugosus* Eggers, 1920c: 41.

= *Xyleborus consobrinus* Eggers, 1932c: 296.

= *Xyleborus discrepans* Schedl, 1950b: 29.

= *Xyleborus annectens* Schedl, 1957b: 88.

= *Xyleborus peramploides* Schedl, 1957b: 102.

• *Xyleborus procer* Eichhoff, 1878b: 402.

• *Xyleborus productus* Hagedorn, 1905b: 414.

• *Xyleborus prolatus* Wood, 1974a: 41.

• *Xyleborus prolixus* Schedl, 1963g: 307.

• *Xyleborus pseudoacuminatus* Wood, 2007: 448.

• *Xyleborus pseudoambasius* Schedl, 1954d: 82.

= *Xyleborus bobirae* Schedl, 1958a: 881.

• *Xyleborus pseudoferox* Wood, 2007: 426.

• *Xyleborus pubescens* Zimmermann, 1868: 145.

= *Xyleborus propinquus* Eichhoff, 1869: 281.

• *Xyleborus pusio* Eggers, 1941a: 105.

• *Xyleborus quadratus* Blandford, 1898a: 209.

• *Xyleborus quadrisignatus* Schedl, 1972d: 200.

• *Xyleborus reunionis* Schedl, 1961g: 147.

• *Xyleborus rothkirchi* Eggers, 1920c: 45.

• *Xyleborus ruandae* Schedl, 1957b: 92.

• *Xyleborus rufipes* Eggers, 1933f: 31.

• *Xyleborus rufobrunneus* Eggers, 1929f: 49 (RN).

= *Xyleborus similis* Eggers, 1927b: 101.

= *Xyleborus rufobrunneus dihingensis* Eggers, 1930b: 189.

• *Xyleborus rugatus* Blackburn in: Blackburn and Sharp 1885: 192.

= *Xyleborus nuuanus* Schedl, 1941b: 114.

• *Xyleborus rugulosipes* Wood, 2007: 446.

• *Xyleborus rusticanus* Beaver & Liu, 2016: 27.

• *Xyleborus sakalava* Schedl, 1953c: 97.

• *Xyleborus sartor* Schedl, 1961g: 156.

• *Xyleborus scaber* Schedl, 1949b: 273.

• *Xyleborus scabratus* Schedl, 1941b: 113 (*Xyleborus oahuensis scabratus*).

• *Xyleborus scabricollis* (Schedl, 1975h: 220) (*Ozopemon*).

• *Xyleborus schildi* Schedl, 1935c: 94.

• *Xyleborus schoutedeni* Eggers, 1927c: 189.

• *Xyleborus scobinatus* Hagedorn, 1910: 8.

• *Xyleborus semipunctatus* Eggers, 1933f: 30.

• *Xyleborus setulosus* Eggers, 1940f: 149.

= *Xyleborus barbatogranosus* Schedl, 1942d: 37.

• *Xyleborus sextuberculatus* Schedl, 1952a: 461.

• *Xyleborus simillimus* Perkins, 1900: 176.

• *Xyleborus singhi* Park & Smith in: Park et al. 2020: 222.

• *Xyleborus solutus* Schedl, 1963g: 516.

• *Xyleborus sparsegranulosus* Kirkendall & Jordal, 2006: 737.

• *Xyleborus sparsipilosus* Eggers, 1933f: 34.

= *Xyleborus inconveniens* Schedl, 1948c: 577.

• *Xyleborus spathipennis* Eichhoff, 1868b: 145.

= *Xyleborus coronatus* Eichhoff, 1878b: 348.

= *Boroxylon burgdorfi* Hopkins, 1915d: 59.

= *Xyleborus curtus* Eggers, 1928a: 94.

= *Xyleborus femoratus* Eggers, 1928a: 95.

• *Xyleborus spinulosus* Blandford, 1898a: 201.

= *Xyleborus fusciseriatus* Eggers, 1934a: 82.

= *Xyleborus spinosulus* Schedl, 1934d: 178.

= *Xyleborus artespinulosus* Schedl, 1935c: 93.

• *Xyleborus squamulatus* Eichhoff, 1869: 282.

= *Xyleborus squamulatus niger* Nunberg, 1971: 61.

• *Xyleborus suaui* Schedl, 1973c: 75.

• *Xyleborus subasperulus* Eggers, 1935e: 310.

• *Xyleborus subcarinulatus* Eggers, 1932c: 302.

• *Xyleborus subductus* Schedl, 1976: 78.

• *Xyleborus subgranosus* Schedl, 1963g: 309.

• *Xyleborus submolestus* Schedl, 1941c: 403.

• *Xyleborus subplanatus* Eggers, 1943d: 386.

• *Xyleborus subtruncatus* Schedl, 1972d: 201.

• *Xyleborus subtuberculatus* Eggers, 1927c: 194.

• *Xyleborus sulcaticeps* Schedl, 1963g: 318.

• *Xyleborus sunisae* Smith, Beaver & Cognato, 2020: 379.

• *Xyleborus tinnitus* Schedl, 1941c: 402.

• *Xyleborus titubanter* Schedl, 1948c: 578.

= *Xyleborus dissidens* Wood, 1974a: 41.

• *Xyleborus tonsus* (Hagedorn, 1905b: 412) (*Dryocoetes*).

• *Xyleborus torquatus* Eichhoff, 1868b: 146.

• *Xyleborus touotai* Beaver, 1995: 15.

• *Xyleborus transitus* Schedl, 1963g: 170.

• *Xyleborus triangi* Schedl, 1958e: 103.

• *Xyleborus tribulatus* Wood, 1974a: 39.

• *Xyleborus tumucensis* Hagedorn, 1905b: 414.

= *Xyleborus guayanensis* Eggers, 1933f: 26.

• *Xyleborus turraeanthus* Schedl, 1957b: 103.

• *Xyleborus ugandaensis* Schedl, 1958a: 882.

• *Xyleborus uncatus* Schedl, 1970e: 96.

• *Xyleborus ustus* Schedl, 1957b: 96.

• *Xyleborus vagabundus* Schedl, 1949b: 277.

• *Xyleborus vanrynae* Browne, 1973: 294.

• *Xyleborus vicinus* (Eichhoff, 1878b: 464).

• *Xyleborus viduus* Eichhoff, 1878b: 391.

• *Xyleborus vismiae* Wood, 1974a: 39.

• *Xyleborus vitiosus* Schedl, 1940a: 367.

• *Xyleborus vochysiae* Kirkendall, 2006: 211.

• *Xyleborus volutus* Wood, 2007: 452.

• *Xyleborus volvulus* (Fabricius, 1775: 454) (*Bostrichus*).

= *Xyleborus alternans* Eichhoff, 1869: 280.

= *Xyleborus badius* Eichhoff, 1869: 280.

= *Xyleborus interstitialis* Eichhoff, 1878b: 375.

= *Xyleborus guanajuatensis* Dugès, 1888: 140.

= *Xyleborus grenadensis* Hopkins, 1915d: 65.

= *Xyleborus hubbardi* Hopkins, 1915d: 65.

= *Xyleborus rileyi* Hopkins, 1915d: 65.

= *Xyleborus schwarzi* Hopkins, 1915d: 65.

= *Xyleborus continentalis* Eggers, 1920c: 42.

= *Xyleborus silvestris* Beeson, 1929: 241.

= *Xyleborus granularis* Schedl, 1950a: 898.

• *Xyleborus vulcanus* Perkins, 1900: 179.

= *Xyleborus truncatus* Sharp in: Blackburn and Sharp 1885: 192.

= *Xyleborus adspersus* Schedl, 1958i: 152 (RN).

= *Xyleborus pacificus* Nunberg, 1959a: 432.

• *Xyleborus xylographus* (Say, 1826: 256) (*Bostrichus*).

= *Xyleborus inermis* Eichhoff, 1868c: 401.

= *Xyleborus canadensis* Swaine, 1917: 24.

• *Xyleborus xylotrupes* Schedl, 1963g: 311.

• *Xyleborus yunnanensis* Smith, Beaver & Cognato, 2020: 382.

#### *Xylosandrus* Reitter

53 spp.

• *Xylosandrus abruptulus* (Schedl, 1953b: 81) (*Xyleborus*).

• *Xylosandrus adherescens* Schedl, 1971e: 375.

• *Xylosandrus amputatus* (Blandford, 1894d: 575) (*Xyleborus*).

= *Xyleborus melli* Schedl, 1938d: 463.

• *Xylosandrus arquatus* (Sampson, 1912: 246) (*Xyleborus*).

• *Xylosandrus assequens* Schedl, 1971e: 376.

• *Xylosandrus aurinegro* Gómez & Hulcr in: Gomez et al. 2020: 722.

• *Xylosandrus beesoni* Saha, Maiti & Chakraborti, 1993: 11.

• *Xylosandrus bellinsulanus* Smith, Beaver & Cognato, 2020: 390.

• *Xylosandrus borealis* Nobuchi, 1981a: 34.

• *Xylosandrus borneensis* Dole & Cognato, 2010: 477.

• *Xylosandrus brevis* (Eichhoff, 1877a: 121) (*Xyleborus*).

= *Xyleborus cucullatus* Blandford, 1894c: 121.

= *Xyleborus montanus* Niisima, 1910: 13.

• *Xylosandrus cancellatus* (Eggers, 1936e: 89) (*Xyleborus*).

= *Xyleborus cancellatus pronunciatus* Eggers, 1936e: 90.

• *Xylosandrus compactus* (Eichhoff in: Chapuis and Eichhoff 1876: 201) (*Xyleborus*).

= *Xyleborus morstatti* Hagedorn, 1912b: 37.

• *Xylosandrus corthyloides* (Schedl, 1934a: 86) (*Xyleborus*).

= *Xyleborus percorthyloides* Schedl, 1957b: 85 (RN).

• *Xylosandrus crassiusculus* (Motschulsky, 1866: 403) (*Phloeotrogus*).

= *Xyleborus semiopacus* Eichhoff, 1878b: 334.

= *Xyleborus semigranosus* Blandford, 1896e: 211.

= *Dryocoetes bengalensis* Stebbing, 1908: 12.

= *Xyleborus mascarenus* Hagedorn, 1908: 379.

= *Xyleborus ebriosus* Niisima, 1909: 154.

= *Xyleborus okoumeensis* Schedl, 1935g: 271.

• *Xylosandrus cubensis* Bright, 2019: 308.

• *Xylosandrus curtulus* (Eichhoff, 1869: 281) (*Xyleborus*).

= *Anisandrus zimmermanni* Hopkins, 1915d: 68.

= *Xyleborus curtuloides* Eggers, 1941a: 102.

= *Xyleborus biseriatus* Schedl, 1963f: 226.

= *Xyleborus strumosus* Schedl, 1972f: 73.

• *Xylosandrus declivigranulatus* (Schedl, 1936c: 30) (*Xyleborus*).

• *Xylosandrus dentipennis* Park & Smith in: Park et al. 2020: 224.

• *Xylosandrus derupteterminatus* (Schedl, 1951i: 64) (*Xyleborus*).

• *Xylosandrus deruptulus* (Schedl, 1942d: 37) (*Xyleborus*).

• *Xylosandrus discolor* (Blandford, 1898b: 429) (*Xyleborus*).

= *Xyleborus posticestriatus* Eggers, 1939a: 119.

• *Xylosandrus diversepilosus* (Eggers, 1941c: 224) (*Xyleborus*).

• *Xylosandrus eupatorii* (Eggers, 1940f: 140) (*Xyleborus*).

• *Xylosandrus ferinus* (Schedl, 1936c: 31) (*Xyleborus*).

• *Xylosandrus formosae* (Wood, 1992: 80) (*Xyleborus*) (RN).

= *Xyleborus formosanus* Browne, 1981a: 131.

• *Xylosandrus geduensis* Smith & Beaver in: Beaver and Smith 2022: 5.

• *Xylosandrus germanus* (Blandford, 1894c: 106) (*Xyleborus*).

= *Xyleborus orbatus* Blandford, 1894c: 123.

• *Xylosandrus hirsutipennis* (Schedl, 1961g: 144) (*Xyleborus*).

• *Xylosandrus hulcri* Dole & Cognato, 2010: 508.

• *Xylosandrus jaintianus* (Schedl, 1967a: 161) (*Xyleborus*).

• *Xylosandrus luokengensis* Lin & Gao in: Lin et al. 2024b: 634.

• *Xylosandrus mancus* (Blandford, 1898b: 428) (*Xyleborus*).

= *Xyleborus abruptus* Sampson, 1914: 388.

= *Xyleborus mancus formosanus* Eggers, 1930b: 186.

• *Xylosandrus mascareniformis* (Eggers, 1927e: 400) (*Xyleborus*).

= *Xyleborus onerosus* Schedl, 1942b: 185.

• *Xylosandrus mediocris* (Schedl, 1942b: 185) (*Xyleborus*).

• *Xylosandrus mesuae* (Eggers, 1930b: 182) (*Xyleborus*).

• *Xylosandrus metagermanus* (Schedl, 1951i: 58) (*Xyleborus*).

• *Xylosandrus mixtus* (Schedl, 1979e: 108) (*Xyleborus*).

• *Xylosandrus monteithi* Dole & Beaver, 2008: 483.

• *Xylosandrus morigerus* (Blandford, 1894b: 264) (*Xyleborus*).

= *Xyleborus coffeae* Wurth, 1908: 2.

= *Xyleborus difficilis* Eggers, 1923b: 174.

= *Xyleborus luzonicus* Eggers, 1923b: 174.

= *Xyleborus abruptoides* Schedl, 1955e: 298.

• *Xylosandrus nanlingensis* Lin & Gao in: Lin et al. 2024b: 637.

• *Xylosandrus pusillus* (Schedl, 1962e: 91) (*Xyleborus*).

• *Xylosandrus pygmaeus* (Eggers, 1940f: 142) (*Xyleborus*).

• *Xylosandrus queenslandi* Dole & Beaver, 2008: 486.

• *Xylosandrus ramulorum* (Schedl, 1957b: 115) (*Xyleborus*).

• *Xylosandrus rotundicollis* (Browne, 1983c: 73) (*Xyleborus*).

• *Xylosandrus russulus* (Schedl, 1942c: 187) (*Xyleborus*).

• *Xylosandrus spinifer* Smith, Beaver & Cognato, 2020: 408.

• *Xylosandrus subsimiliformis* (Eggers, 1939b: 11) (*Xyleborus*).

• *Xylosandrus subsimilis* (Eggers, 1930b: 186) (*Xyleborus*).

• *Xylosandrus terminatus* (Eggers, 1930b: 182) (*Xyleborus*).

• *Xylosandrus trunculus* Park & Smith in: Park et al. 2020: 225.

• *Xylosandrus woodi* Dole & Beaver, 2008: 488.

### XYLOCTONINI Eichhoff

5 genera, 85 spp.

### GLOSTATINA Jordal

1 genus, 29 spp.

#### *Glostatus* Schedl

29 spp.

• *Glostatus acaciae* (Schedl, 1957c: 155) (*Apoglostatus*).

= *Glostatus hirsutus* Schedl, 1965g: 1070.

• *Glostatus aculeus* Jordal, 2023: 229.

• *Glostatus acutidentes* Jordal, 2023: 214.

• *Glostatus bispinosus* Beaver in: Beaver and Löyttyniemi 1985: 77.

• *Glostatus carinifer* Schedl, 1958a: 871.

• *Glostatus declividepressus* Schedl, 1939i: 386.

= *Glostatus assimilis* Schedl, 1958a: 871.

• *Glostatus delicatus* (Schedl, 1965g: 1072) (*Paraglostatus*).

• *Glostatus dispar* (Eggers, 1936c: 35) (*Stephanoderes*).

= *Stephanopodius giganteus* Schedl, 1950b: 26.

• *Glostatus ghanaensis* (Schedl, 1962g: 66) (*Hypocryphalus*).

• *Glostatus giganteus* (Schedl, 1950b: 22) (*Cryphalus*).

• *Glostatus gracilior* (Schedl, 1958a: 872) (*Apoglostatus*).

• *Glostatus kenyae* Schedl, 1957c: 154.

• *Glostatus leprosus* Browne, 1973: 290.

• *Glostatus mkulumusius* (Eggers, 1920a: 241) (*Stephanoderes*).

= *Stephanopodius usambaricus* Schedl, 1941c: 396.

• *Glostatus multispinosus* (Schedl, 1958a: 873) (*Ctonocryphus*).

• *Glostatus nigrivestis* (Schedl, 1958a: 873) (*Ctonocryphus*).

• *Glostatus nunbergi* Jordal, 2023: 213 (RN).

= *Stephanopodius squamosus* Nunberg, 1973: 9.

• *Glostatus paraxyloctonus* Jordal, 2023: 227.

• *Glostatus pondoanus* Schedl, 1958b: 558.

= *Glostatus squamosus* Schedl, 1965g: 1071.

= *Hypocryphalus dubiosus* Schedl, 1970d: 177.

• *Glostatus procurvus* Jordal, 2023: 221.

• *Glostatus scutiae* (Schedl, 1959a: 708) (*Stephanoderes*).

• *Glostatus seydeli* (Nunberg, 1967: 321) (*Rhopalocryphus*).

• *Glostatus spinicarinatus* Beaver in: Beaver and Löyttyniemi 1985: 78.

• *Glostatus tenuis* Jordal, 2023: 207.

• *Glostatus tredli* (Reitter, 1908a: 55) (*Cryphalus*).

• *Glostatus tuberculatus* Jordal, 2023: 218.

• *Glostatus vrydaghi* (Nunberg, 1973: 10) (*Apoglostatus*).

• *Glostatus xanthophloeae* (Schedl, 1957b: 58) (*Stephanoderes*).

• *Glostatus xyloctonus* (Schedl, 1941c: 398) (*Ctonocryphus*).

### XYLOCTONINA Eichhoff

4 genera, 56 spp.

#### *Cryphalomimus* Eggers

3 spp.

• *Cryphalomimus ater* Nunberg, 1965: 23.

• *Cryphalomimus grandis* Schedl, 1971d: 10.

• *Cryphalomimus striatus* Eggers, 1927c: 174.

#### *Ctonoxylon* Hagedorn

24 spp.

• *Ctonoxylon acuminatum* Schedl, 1957b: 45 (E).

• *Ctonoxylon amanicum* Hagedorn, 1912b: 42.

= *Ctonoxylon intermedium* Schedl, 1971d: 10.

• *Ctonoxylon atrum* Browne, 1965: 190.

• *Ctonoxylon auratum* Hagedorn, 1910: 4.

• *Ctonoxylon bosqueiae* Schedl, 1962g: 66.

• *Ctonoxylon camerunum* Hagedorn, 1910: 4.

= *Ctonoxylon fuscum* Hagedorn, 1910: 5.

= *Ctonoxylon conradti* Schedl, 1939c: 171.

• *Ctonoxylon caudatum* Schedl, 1971d: 8.

• *Ctonoxylon cornutum* Eggers, 1943a: 246.

• *Ctonoxylon crenatum* Hagedorn, 1910: 5.

• *Ctonoxylon festivum* Schedl, 1941c: 389.

= *Ctonoxylon dentigerum* Schedl, 1941c: 388.

• *Ctonoxylon flavescens* Hagedorn, 1910: 4.

= *Ctonoxylon flavescens usambaricum* Eggers, 1920c: 38.

• *Ctonoxylon hirsutum* Hagedorn, 1910: 4 (*Ctonoxylon camerunum hirsutum*).

• *Ctonoxylon hirtellum* Schedl, 1971d: 9.

• *Ctonoxylon kivuense* Schedl, 1957b: 44 (E).

• *Ctonoxylon methneri* Eggers, 1922c: 170.

= *Ctonoxylon griseum* Schedl, 1941c: 389.

= *Ctonoxylon hamatum* Schedl, 1941c: 390.

• *Ctonoxylon montanum* Eggers, 1922c: 170.

= *Ctonoxylon longipilum* Eggers, 1935e: 308.

= *Ctonoxylon nodosum* Eggers, 1940e: 236.

• *Ctonoxylon pilosum* Jordal, 2024b: 115.

• *Ctonoxylon pygmaeum* Eggers, 1920c: 39.

• *Ctonoxylon quadrispinum* Jordal, 2024b: 118.

• *Ctonoxylon spathifer* Schedl, 1951g: 39.

• *Ctonoxylon spinifer* Eggers, 1920c: 39.

= *Ctonoxylon setifer* Eggers, 1920c: 39.

• *Ctonoxylon torquatum* Jordal, 2024b: 113.

• *Ctonoxylon tuberculatum* Jordal, 2024b: 108.

• *Ctonoxylon uniseriatum* Schedl, 1965d: 114.

= *Ctonoxylon capensis* Schedl, 1971d: 8.

#### *Scolytomimus* Blandford

14 spp.

• *Scolytomimus andamanensis* Wood, 1988c: 199.

• *Scolytomimus assamensis* Schedl, 1951i: 54.

= *Scolytomimus bassiae* Schedl, 1951i: 54.

= *Scolytomimus quadrioculatus* Browne, 1958: 490.

• *Scolytomimus baloghi* Schedl, 1969c: 157.

• *Scolytomimus bicolor* Wood, 1960a: 18.

• *Scolytomimus costipennis* Schedl, 1953f: 291.

• *Scolytomimus dilutus* Blandford, 1895: 320.

• *Scolytomimus maculatus* Beeson, 1929: 223.

• *Scolytomimus mimusopis* Wood, 1988c: 200.

• *Scolytomimus philippinensis* (Eggers, 1923b: 143) (*Neoxyloctonus*).

• *Scolytomimus pusillus* (Eggers, 1927b: 88) (*Neoxyloctonus*).

= *Scolytomimus kalshoveni* Eggers, 1940f: 132.

= *Scolytomimus brunigi* Browne, 1958: 488.

= *Scolytomimus menoni* Browne, 1958: 487.

= *Scolytocleptes insularis* Schedl, 1962a: 491.

• *Scolytomimus quadridens* Wood, 1988c: 200.

• *Scolytomimus rectus* Wood, 1988c: 201.

• *Scolytomimus tenuis* Browne, 1984d: 77.

• *Scolytomimus woodi* Browne, 1958: 489.

#### *Xyloctonus* Eichhoff

15 spp.

• *Xyloctonus aethiops* Schedl, 1953c: 77.

= *Xyloctonus stenographus* Schedl, 1961g: 130.

• *Xyloctonus bimarginatus* Eggers, 1939d: 17.

• *Xyloctonus biseriatus* Schedl, 1953c: 76.

• *Xyloctonus genieri* Jordal, 2024a: 77.

• *Xyloctonus maculatus* Schedl, 1965d: 113.

• *Xyloctonus magnus* Jordal, 2024a: 80.

• *Xyloctonus mauritianus* Menier, 1974: 662.

• *Xyloctonus niger* Schedl, 1938a: 452.

• *Xyloctonus opacus* Schedl, 1957b: 43.

• *Xyloctonus pubifer* Schedl, 1966f: 365.

• *Xyloctonus punctipennis* Eggers, 1939d: 16.

• *Xyloctonus quadricinctus* Schedl, 1941c: 387.

• *Xyloctonus quadridens* Schedl, 1953c: 77.

• *Xyloctonus scolytoides* Eichhoff, 1872a: 134.

= *Xyloctonus emarginatus* Eggers, 1939d: 16.

= *Xyloctonus latus* Eggers, 1939d: 14.

• *Xyloctonus subcostatus* Eggers, 1939d: 15.

= *Xyloctonus striatus* Eggers, 1939d: 18.

### XYLOTERINI LeConte

3 genera, 25 spp.

#### *Indocryphalus* Eggers

11 spp.

• *Indocryphalus aceris* (Niisima, 1910: 4) (*Xyloterus*).

= *Xyloterus dainichiensis* Murayama, 1954: 191.

• *Indocryphalus chiyui* Lin & Beaver, 2024: 258.

• *Indocryphalus intermedius* (Sampson, 1913: 445) (*Xyloterus*).

= *Indocryphalus malaisei* Eggers, 1939b: 7.

• *Indocryphalus machili* Wood, 1988c: 197.

• *Indocryphalus major* (Eggers, 1926b: 148) (*Trypodendron majus*).

= *Xyloterus ashuensis* Murayama, 1950b: 51.

• *Indocryphalus pasohensis* Beaver, 2000: 167.

• *Indocryphalus pubipennis* (Blandford, 1894c: 125) (*Trypodendron pubipenne*).

• *Indocryphalus sericeus* (Schedl, 1942d: 10) (*Erioschidias*).

= *Ptilopodius kalshoveni* Schedl, 1954c: 149.

• *Indocryphalus sordidus* (Blandford, 1894d: 577) (*Trypodendron sordidum*).

= *Trypodendron sinense* Eggers, 1941c: 225.

• *Indocryphalus suongmu* Cognato, Smith & Pham, 2015: 498.

• *Indocryphalus tropicus* (Browne, 1950: 648) (*Trypodendron tropicum*).

#### *Trypodendron* Stephens

13 spp.

• *Trypodendron betulae* Swaine, 1911: 216.

• *Trypodendron domesticum* (Linnaeus, 1758: 356) (*Dermestes domesticus*).

= *Bostrichus limbatus* Herbst, 1783: 26.

= *Apate limbatus* Fabricius, 1787: 33.

= *Xyloterus domesticus apicalis* Endrödi, 1957: 309.

= *Xyloterus domesticus toracalis* Endrödi, 1957: 309.

• *Trypodendron dorjitenzingi* Schmutzenhofer, 1988: 487.

• *Trypodendron gaimaense* (Murayama, 1937: 369) (*Xyloterus gaimaensis*).

• *Trypodendron laeve* Eggers, 1939a: 122.

= *Trypodendron piceum* Strand, 1946: 172.

• *Trypodendron lineatum* (Olivier, 1800b: 18) (*Bostrichus lineatus*).

= *Apate bivittata* Kirby, 1837: 192.

= *Bostrichus cavifrons* Mannerheim, 1843: 297.

= *Trypodendron vittiger* Eichhoff, 1881: 298.

= *Trypodendron borealis* Swaine, 1917: 21.

= *Trypodendron granulatum* Eggers, 1933b: 51.

= *Trypodendron meridionale* Eggers, 1940g: 38.

= *Xyloterus lineatus lineellus* Endrödi, 1957: 309.

= *Xyloterus lineatus pauper* Endrödi, 1957: 309.

• *Trypodendron niponicum* Blandford, 1894c: 125 (*Trypodendronquercum nipo­nicum*).

• *Trypodendron proximum* (Niisima, 1909: 165) (*Xyloterus proximus*).

• *Trypodendron pulchellum* (Murayama, 1957a: 585) (*Xyloterus pulchellus*).

• *Trypodendron retusum* (LeConte, 1868: 158) (*Xyloterus retusus*).

• *Trypodendron rufitarse* (Kirby, 1837: 193) (*Apate rufitarsis*).

= *Trypodendron ponderosae* Swaine, 1917: 22.

• *Trypodendron scabricolle* (LeConte, 1868: 158) (*Xyloterus scabricollis*).

• *Trypodendron signatum* (Fabricius, 1792: 363) (*Apate*).

= *Bostrichus quinquelineatus* Adams, 1817: 312.

= *Bostrichus waringii* Curtis, 1840: 279.

= *Xyloterus quercus* Eichhoff, 1864b: 381.

= *Trypodendron suturale* Eggers, 1933b: 52.

= *Trypodendron obtusum* Eggers, 1939a: 121.

#### *Xyloterinus* Swaine

1 sp.

• *Xyloterinus politus* (Say, 1826: 256) (*Bostrichus*).

= *Xyloterus unicolor* Eichhoff, 1872a: 136.

## Additional information

### Use of AI

No use of AI was reported.

### Author ORCIDs

Andrew J. Johnson https://orcid.org/0000-0003-3139-2257

Thomas H. Atkinson https://orcid.org/0000-0002-9675-8507

Roger A. Beaver https://orcid.org/0000-0003-1932-3208

Anthony I. Cognato https://orcid.org/0000-0001-6436-2447

Bjarte H. Jordal https://orcid.org/0000-0001-6082-443X

Miloš Knížek https://orcid.org/0000-0003-3221-9448

Michail Yu. Mandelshtam https://orcid.org/0000-0002-7135-3239

Robert J. Rabaglia https://orcid.org/0000-0001-8591-5338

Sarah M. Smith https://orcid.org/0000-0002-5173-3736

Jiri Hulcr https://orcid.org/0000-0002-8706-4618

### Data availability

All of the data that support the findings of this study are available in the main text or Supplementary Information.

## Supplementary materials

Supplementary material 1
Scolytinae described names and valid species or subspecies Data type tsv https://binary.pensoft.net/file/1545224This dataset is made available under the Open Database License (http://opendatacommons.org/licenses/odbl/1.0/). The Open Database License (ODbL) is a license agreement intended to allow users to freely share, modify, and use this Dataset while maintaining this same freedom for others, provided that the original source and author(s) are credited. Andrew J. Johnson, Thomas H. Atkinson, Roger A. Beaver, Anthony I. Cognato, Bjarte H. Jordal, Miloš Knížek, Michail Yu. Mandelshtam, Robert J. Rabaglia, Sarah M. Smith, Jiri Hulc
